# Stromal cyclin D1 promotes heterotypic immune signaling and breast cancer growth

**DOI:** 10.18632/oncotarget.19953

**Published:** 2017-08-04

**Authors:** Timothy G. Pestell, Xuanmao Jiao, Mukesh Kumar, Amy R. Peck, Marco Prisco, Shengqiong Deng, Zhiping Li, Adam Ertel, Mathew C. Casimiro, Xiaoming Ju, Agnese Di Rocco, Gabriele Di Sante, Sanjay Katiyar, Alison Shupp, Michael P. Lisanti, Pooja Jain, Kongming Wu, Hallgeir Rui, Douglas C. Hooper, Zuoren Yu, Aaron R. Goldman, David W. Speicher, Lisa Laury-Kleintop, Richard G. Pestell

**Affiliations:** ^1^ Pennsylvania Cancer and Regenerative Medicine Research Center, Baruch S. Blumberg Institute, Pennsylvania Biotechnology Center, Wynnewood, PA, USA; ^2^ Departments of Cancer Biology, Thomas Jefferson University, Bluemle Life Sciences Building, Philadelphia, PA, USA; ^3^ Department of Pathology, Medical College of Wisconsin, Milwaukee, WI 53226, USA; ^4^ Translational Medicine, School of Environment and Life Sciences, Biomedical Research Centre, University of Salford, Salford, Greater Manchester, England, UK; ^5^ Department of Microbiology and Immunology, Institute for Molecular Medicine & Infectious Disease, Drexel University College of Medicine, Philadelphia, PA, USA; ^6^ Department of Oncology, Tongji Hospital of Tongji Medical College, Huazhong University of Science and Technology, Wuhan, China; ^7^ Department of Microbiology, Thomas Jefferson University, Bluemle Life Sciences Building, Philadelphia, PA, USA; ^8^ Research Center for Translational Medicine, East Hospital, Tongji University School of Medicine, Shanghai, China; ^9^ Molecular and Cellular Oncogenesis Program, The Wistar Institute, Philadelphia, PA, USA; ^10^ Lankenau Institute for Medical Research, Wynnewood, PA, USA; ^11^ Lee Kong Chian School of Medicine, Nanyang Technological University, Singapore

**Keywords:** cyclin D1, OPN, CAFs, stem cell, breast cancer

## Abstract

The *cyclin D1* gene encodes the regulatory subunit of a holoenzyme that drives cell autonomous cell cycle progression and proliferation. Herein we show cyclin D1 abundance is increased >30-fold in the stromal fibroblasts of patients with invasive breast cancer, associated with poor outcome. Cyclin D1 transformed hTERT human fibroblast to a cancer-associated fibroblast phenotype. Stromal fibroblast expression of cyclin D1 (cyclin D1^Stroma^) *in vivo*, enhanced breast epithelial cancer tumor growth, restrained apoptosis, and increased autophagy. Cyclin D1^Stroma^ had profound effects on the breast tumor microenvironment increasing the recruitment of F4/80^+^ and CD11b^+^ macrophages and increasing angiogenesis. Cyclin D1^Stroma^ induced secretion of factors that promoted expansion of stem cells (breast stem-like cells, embryonic stem cells and bone marrow derived stem cells). Cyclin D1^Stroma^ resulted in increased secretion of proinflammatory cytokines (CCL2, CCL7, CCL11, CXCL1, CXCL5, CXCL9, CXCL12), CSF (CSF1, GM-CSF1) and osteopontin (OPN) (30-fold). OPN was induced by cyclin D1 in fibroblasts, breast epithelial cells and in the murine transgenic mammary gland and OPN was sufficient to induce stem cell expansion. These results demonstrate that cyclin D1^Stroma^ drives tumor microenvironment heterocellular signaling, promoting several key hallmarks of cancer.

## INTRODUCTION

The *cyclin D1* gene encodes the regulatory subunit of a holoenzyme that phosphorylates and inactivates the retinoblastoma (pRb) protein, promoting G_1_/S phase cell cycle entry. Cyclin D1 enhances breast cancer cellular proliferation *in vivo* and endogenous cyclin D1 maintains estradiol-mediated mammary epithelial cell gene expression *in vivo* [1]. The abundance of cyclin D1 is rate limiting in the growth of tumors *in vivo*, including ErbB2-induced breast cancer [2, 3] and gastrointestinal tumorigenesis [4]. In addition to canonical signaling governing the G_1_/S cell-cycle transition, cyclin D1 also participates in non canonical cell autonomous functions. Thus cyclin D1 promotes cellular migration [5] and DNA repair [6], governs the expression of specific miRNAs and determines the processing of miRNA through the induction of Dicer [7]. The ability of cyclin D1 to govern gene transcription correlates with the recruitment of cyclin D1 and cointegrator enzyme complexes into the promoter regulatory region of target genes in the context of chromatin [8, 9].

An historical view has focused on oncogenic signaling within tumor cells that drive the hallmarks of cancer [10]. However, solid tumors consist of both tumor cells and stromal cells. Modification of fibroblasts in the stroma immediately adjacent to transformed epithelial cells has been documented in several tumor systems [11-15]. Cancer-associated fibroblasts (CAFs) are a preponderant stromal population in many tumor types [16]. CAFs originate from different cell populations including bone-marrow mesenchymal stem cells [17], resident fibroblasts [18], cancer cells (following epithelial to mesenchymal transition (EMT)) or endothelial cells [19, 20]. The growth characteristics of breast cancer-associated fibroblasts are different from those of fibroblasts associated with normal breast epithelial cells [21]. CAFs associated with invasive breast carcinoma cells convey abnormal migratory behavior *in vitro* [22] and altered expression of growth factors such as platelet-derived growth factor, insulin-like growth factors I and II, transforming growth factor-β1, hepatocyte growth factor/epithelial scatter factor, and keratinocyte growth factor [21, 23-27] and increased expression of inflammatory genes [28].

Studies of fibroblasts in the vicinity of the malignant lesion support a role for stromal cells in tumorigenesis [29, 30]. In these circumstances genetic changes in the epithelial cell compartment are considered to be the independent drivers of the tumor inflammatory microenvironment. Evidence suggests that oncogenic and collaborative oncogenic signals within tumor epithelial cells recruit inflammatory cells which alter stromal fibroblasts to become cancer-associated fibroblasts (CAFs). Tumor cell derived inflammatory cytokines and growth factors including colony stimulating factor (CSF-1), GM-CSF, CCL2 and other factors which participate in tumor progression [31].

Currently, relatively little evidence supports an alternate model in which changes in the expression of a target gene within the CAFs drive tumorigenesis. Furthermore, the molecular genetic drivers governing the CAF phenotype are not well understood. It is however known that intratumoral hypoxia, which down regulates caveolin-1 via lysosomal degradation [32-35], augments the CAF phenotype [34]. Herein we show the expression of the *cyclin D1* gene is increased in human breast cancer stroma. We show that increased expression of cyclin D1 in stromal fibroblasts can transform it to a cancer-associated fibroblast phenotype and that stromal cyclin D1 is sufficient to augment breast tumor epithelial cell growth in mice. Analysis of heterotypic signals induced by stromal cyclin D1 identified activation of heterocellular signaling that promoted tumor inflammation, angiogenesis and stem cell expansion.

## RESULTS

### Cyclin D1 expression is increased in the stroma of human breast cancer associated with poor prognosis

**Figure 1:**
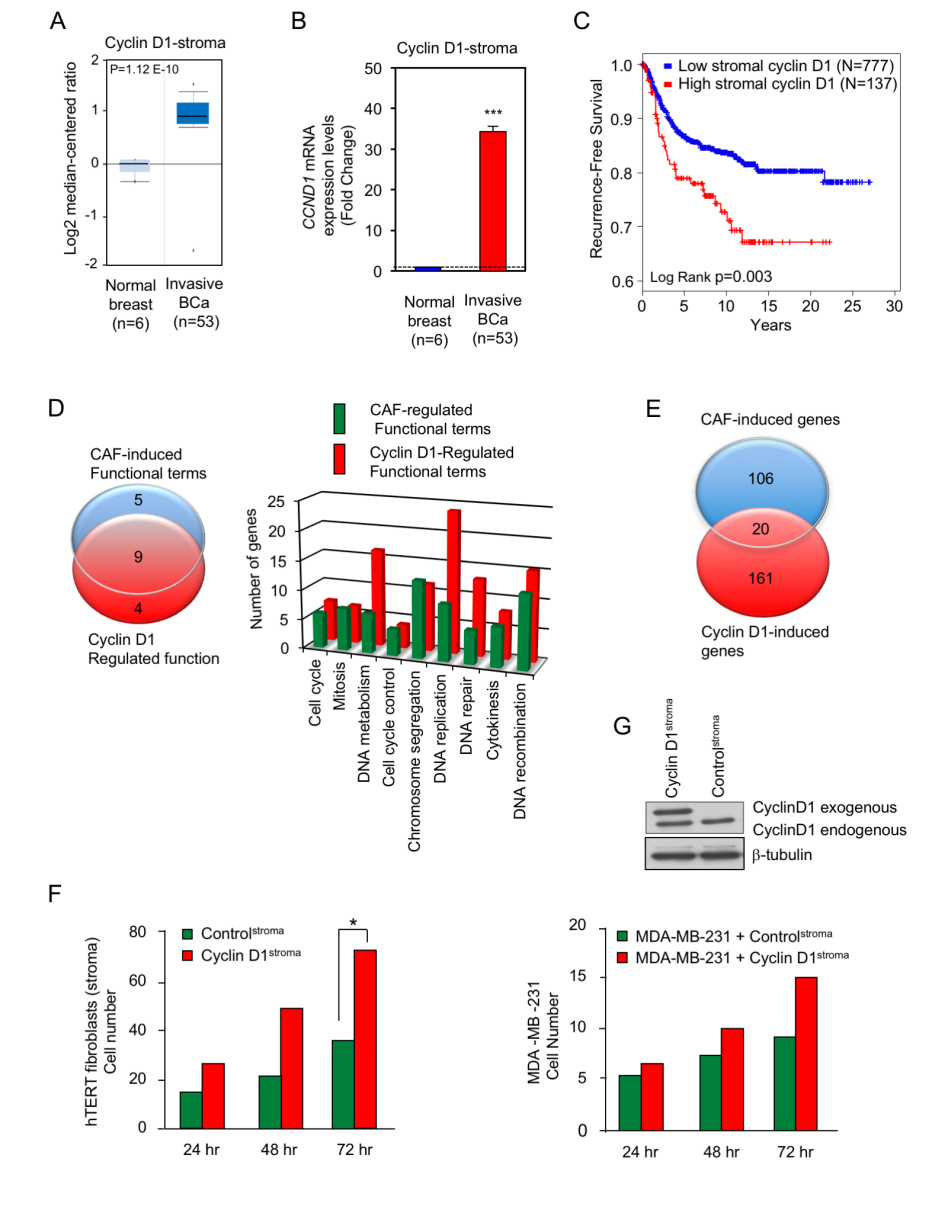
Cyclin D1 is increased in the stroma of human breast cancer associated with poor outcome **A.**. The relative abundance of cyclin D1 in either the normal breast (*N* = 6) or breast cancer stroma (N = 53) was quantitated as mean ± SEM and shown as either Log2 or **B.** relative mRNA abundance. **C.** Kaplan-Meier plot indicating unfavorable prognosis in breast cancer patients with high cyclin D1 in stromal cells. Quantitative immunofluorescence and data-driven dichotomization of nuclear cyclin D1 levels in breast cancer stromal cells is associated with increased risk of disease recurrence (*N* = 914, *p* = 0.03). **D.** Venn diagram of GO terms, or **E.** gene expression comparing cancer-associated fibroblast (CAF) genes (breast cancer-associated fibroblasts compared with normal mammary gland fibroblasts [34] and cyclin D1 induced genes in fibroblasts [62]). **F.** Relative number of fibroblasts or **G.** breast cancer cells determined in co-culture of hTERT fibroblasts (control) or hTERT fibroblasts expressing cyclin D1 (cyclin D1^stroma^) co-incubated with the breast cancer cell line, MDA-MB-231 (*N* = 7 for cyclin D1^stroma^ and *N* = 12 for control at time 72 hour. *N* = 2 for all of other time points).

In view of the finding that the *cyclin D1* gene encodes the regulatory subunit of the holoenzyme that phosphorylates pRB, and RB phosphorylation is increased in human breast cancer-associated fibroblasts [34], we determined the abundance of cyclin D1 in the stroma of human breast cancers. The mRNA for cyclin D1 was increased approximately 32-fold in breast cancer (Figure 1A, 1B).

A cohort of 914 breast cancer specimens with available clinical outcome was stained for stromal cyclin D1 using immunofluorescence-immunohistochemistry. Tissue Studio (Definiens) quantitative analysis was performed on high-resolution digital images obtained using the ScanScope FL line scanner (Leica Biosystems) to determine levels of cyclin D1 expression specifically within the stroma of cancer specimens. A majority of the cyclin D1-positive cells in breast cancer stroma display a fibroblastoid phenotype with their characteristic highly elongated nuclei (Supplementary Figure 1A). Some stromal cell nuclei appear round and may represent other cell types but could also be fibroblastoid cells sectioned transversely. For immunostaining of cyclin D1 in human breast cancer specimens we have used clinical grade DAKO 3642 rabbit monoclonal antibody specific for the 36 kDa human cyclin D1. The antibody has been validated for formalin-fixed paraffin-embedded tissue and is widely used. As expected, the majority of cyclin D1 staining is in the cell nuclei (Supplementary Figure 1A). Data-driven cutpoint analysis using X-tile software [36] identified a sub-population of patients with the highest levels of nuclear cyclin D1 within the stromal cell compartment (Supplementary Figure 1A) to be at a significantly increased risk of breast cancer recurrence (Cox Regression Hazard Ratio = 1.76 (CI: 1.20-2.57), p = 0.004) (Figure 1C). Furthermore the stromal cyclin D1 levels in the tumor were significantly greater than the adjacent normal stromal cyclin D1 in the same patients (Supplementary Figure 1B). Cancer-associated fibroblasts (CAFs) from human breast cancers compared with normal mammary fibroblasts isolated from patients are enriched for gene expression associated with cellular proliferation and pRb/E2F target genes [34]. A comparison of the GO terms evidenced a 64% (9/14) overlap of functional terms induced by cyclin D1 and induced in CAFs compared with normal mammary fibroblasts (Figure 1D). A comparison of cyclin D1-regulated genes in fibroblasts with gene expression induced in CAFs demonstrated 20/126 (16%) of genes were concordant (Figure 1E).

When co-cultured with breast cancer cells, hTERT immotalizd human fibroblasts can acquire a cancer-associated fibroblast phenotype [37]. In order to determine the functional consequence of cyclin D1 expression in the stromal fibroblasts, cyclin D1 was stably integrated into hTERT fibroblasts. Western blot analysis of the hTERT cells overexpressing cyclin D1 (cyclin D1^Stroma^) demonstrated a 2-fold increase in cyclin D1 abundance, which is within the physiological range of changes in cyclin D1 abundance (Figure 1G). Breast cancer cellular growth, and co-culture experiments were conducted as previously described [38]. MDA-MB-231 cells were grown with hTERT fibroblasts expressing cyclin D1 (cyclin D1^Stroma^) or its control GFP vector (control^Stroma^). The proliferation of hTERT fibroblasts with overexpression of cyclin D1 was increased 2-fold (p-value = 0.011), consistent with the known role for cyclin D1 to promote cellular proliferation (Figure 1F). The proliferation of MDA-MB-231 cells was increased approximately 65% at 72 hrs by cyclin D1^Stroma^ suggesting stromal cyclin D1 participates in heterocellular signals to the breast cancer cells (p-value = 0.004) (Figure 1G).

### Stromal cyclin D1 enhances tumor growth, proliferation and reduces apoptosis

**Figure 2:**
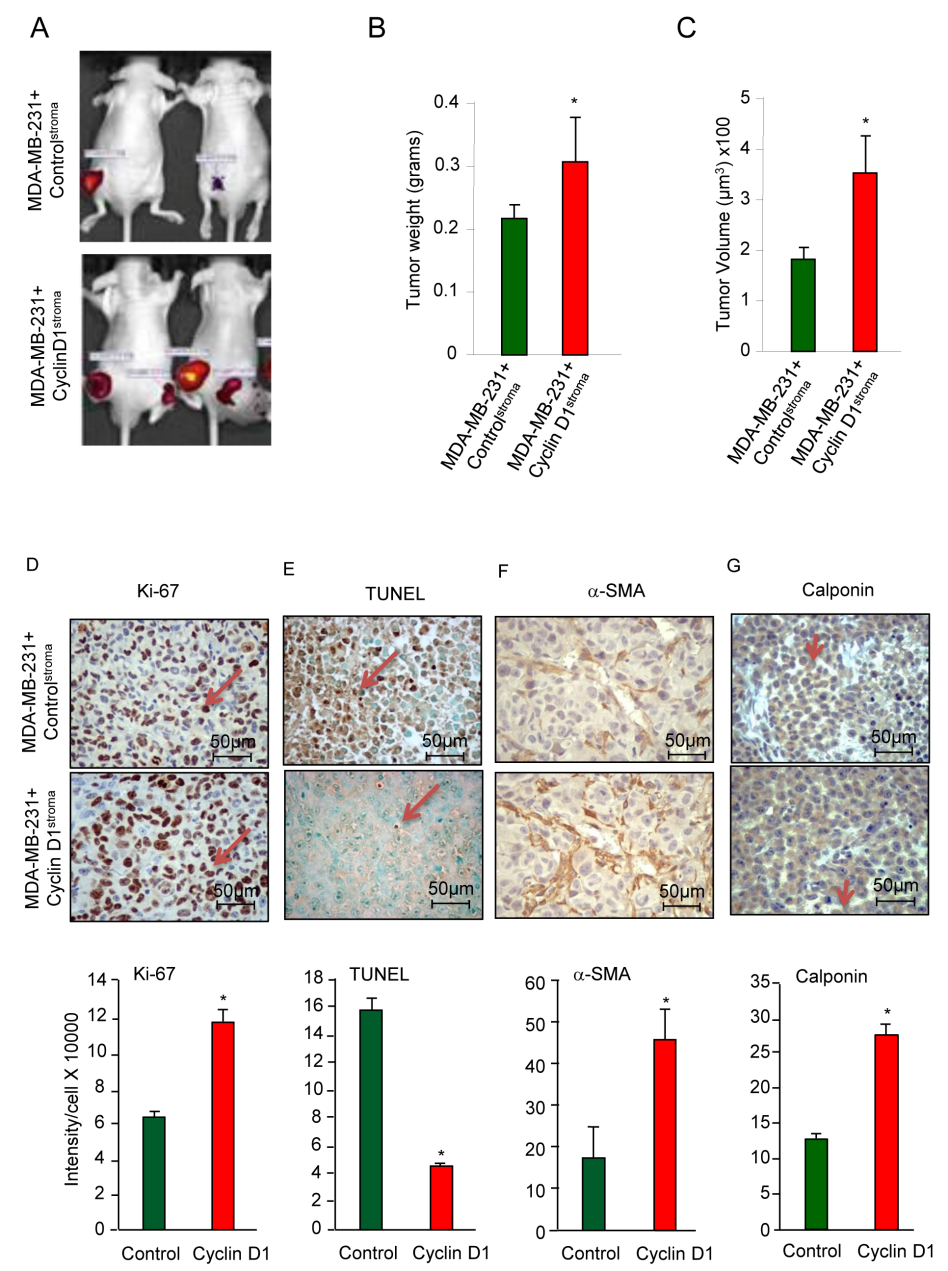
Stromal cyclin D1 expression increases breast tumor growth in mice **A.** Mice were injected with either hTERT control (control^stroma^) or hTERT-cyclin D1 (cyclin D1^stroma^), in equal number together with MDA-MB-231 cells (RFP) and the tumor size assessed with **B.** tumor weight and **C.** tumor volume shown as mean ± SEM for *N* = 6 separate animals in each group. **D.** Immunohistochemical analysis of the tumors for cell proliferation (Ki-67), **E.** apoptosis (TUNEL), **F.** α-SMA and **G.** calponin with quantitation shown as mean ± SEM for *N* = 6 separate animals in each group.

In order to examine the functional significance of an increase in cyclin D1 expression in the tumor-associated fibroblasts, MDA-MB-231 cells were co-injected with hTERT fibroblasts expressing either cyclin D1 (cyclin D1^Stroma^) or control GFP vector (control^Stroma^). Tumors were visualized by the expression of the Luc2 gene (Figure 2A). Tumor weight was increased 40% (0.31 vs 0.22 grams (N = 6)), and tumor volume was increased 93% (351.5 vs 182.3 µM × 100^3^ (N = 6)) (Figure 2B, 2C). Tumors were extirpated and analyzed for markers of proliferation, apoptosis and cancer-associated fibroblast differentiation (Figure 2D-2G). Ki-67, a marker of cellular proliferation, was increased 80% by cyclin D1^Stroma^ in the breast cancer epithelial cells (11.7x10^4^ vs 6.5x10^4^, (N = 4)) (Figure 2D). Terminal deoxynucleotidyl transferase dUTP nick end labeling (TUNEL) staining as a measure of apoptosis was reduced 63% in the breast cancer epithelial cells by cyclin D1^Stroma^ (21x10^4^ vs 7.6x10^4^ for N = 3) (Figure 2E). Induction of the cancer-associated fibroblast phenotype correlates with the expression of calponin and αSMA [37]. The cyclin D1^Stroma^ breast tumors showed increased abundance of αSMA (Figure 2F, 2.6-fold, p = 0.018) and calponin (Figure 2G).

### Stromal cyclin D1 enhances autophagy, features of the cancer-associated fibroblast, neoangiogenesis and tumor inflammation

**Figure 3:**
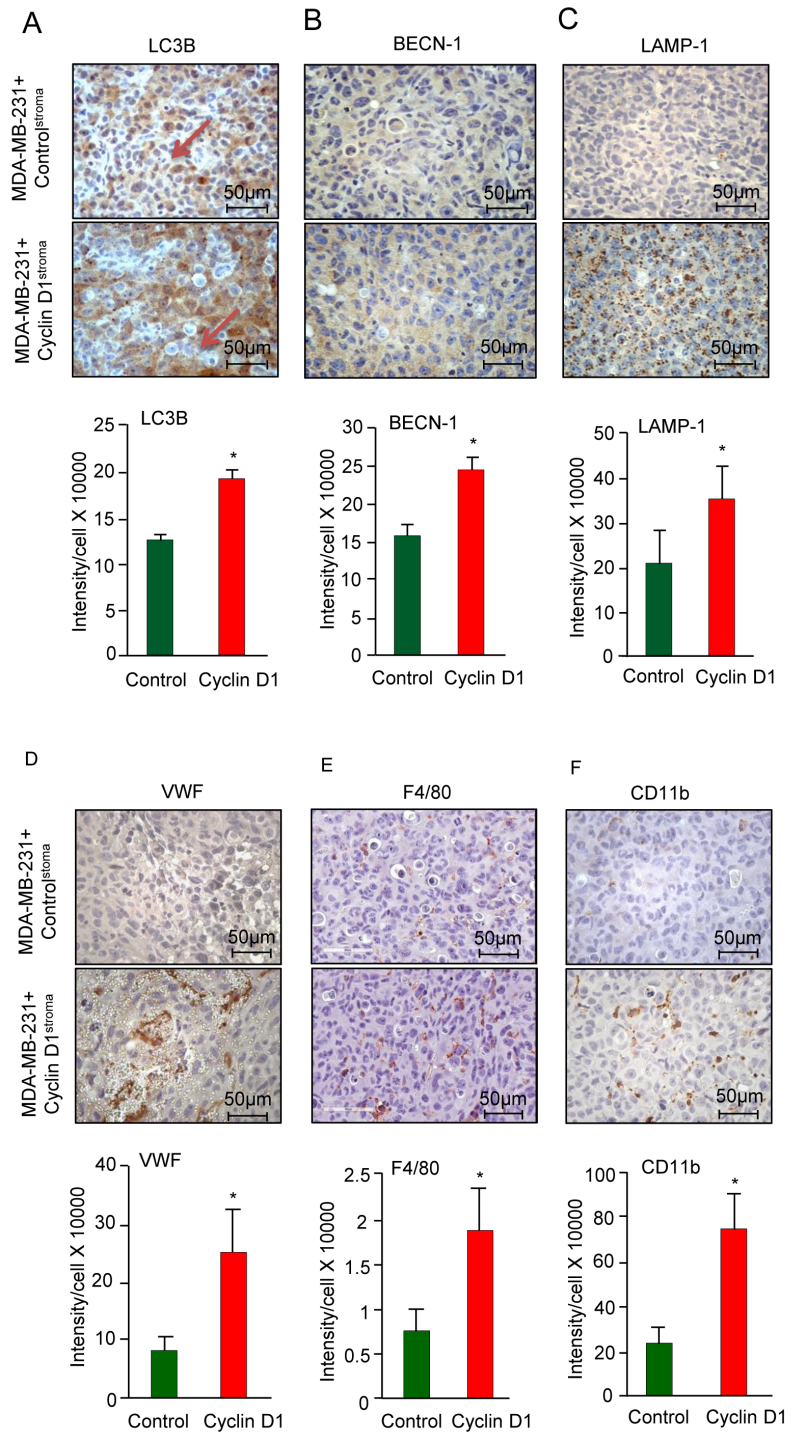
Cyclin D1^stroma^ expression increases breast cancer inflammation **A.** Immunohistochemical analysis of the tumors for **A.**-**C.** mitophagy/autophagy (LC3B, BECN-1, LAMP-1), **D.** angiogenesis (Von Willebrand Factor (VWF), **E.** F4/80^+^ macrophages, **F.** CD11b^+^ macrophages, with quantitation shown as mean ± SEM for *N* = 6 separate animals in each group.

The tumor stroma has the capacity to induce or restrain autophagy in the tumor epithelium [39]. In order to determine whether stromal cyclin D1 induced autophagy in the breast tumor we assessed known markers. LC3B, a marker of autophagy, was induced 40% in the breast cancer epithelial cells (12.4x10^4^ vs 18.9x10^4^ for N = 4) (Figure 3A). Breast cancer epithelial cell abundance of Beclin 1 (BCN1), a novel Bcl-2-interacting mammalian autophagy gene [40], was induced 50% by cyclin D1^Stroma^ (Figure 3B). Chaperone-mediated autophagy (CMA) is a proteolytic system that degrades intracellular proteins in lysosomes. Lysosomal-associated membrane protein 1 (LAMP-1) (also known as lysosome-associated membrane glycoprotein 1 and CD107a (Cluster of Differentiation 107a)), is a marker for chaperone-mediated autophagy [41]. LAMP1 was induced 70% by cyclin D1^Stroma^ (Figure 3C, 2.1x10^5^ vs 3.5x10^5^, N = 4). Collectively these studies demonstrate that increased cyclin D1^Stroma^ promotes breast cancer tumor growth, associated with the induction of cell proliferation, reduction in apoptosis and induction of autophagy in the breast cancer epithelial cell compartment.

As a surrogate for neoangiogenesis we measured the αvβ3 binding ECM protein von Willebrand Factor (vWF), which was increased 4-fold by cyclin D1^Stroma^ (Figure 3D). Tumor-associated macrophages (TAMs) play a critical role in the proliferation, invasion, angiogenesis, and metastasis of human breast carcinomas [42, 43] and increased macrophage infiltration into tumors confers metastatic potential and poor prognosis in breast cancer [44]. We therefore analyzed the presence of F4/80^+^ macrophages, which were increased 2.5-fold by cyclin D1^Stroma^ (Figure 3E). The CD11b^+^ staining macrophages, which also contribute to breast cancer metastasis [45], were increased 3.2-fold by cyclin D1^Stroma^ (Figure 3F).

### Cyclin D1 conditioned medium produces pro-inflammatory cytokines (CCL2, CCL7, CCL11, CXCL1, CXCL5, CXCL9, CXCL12), CSF (CSF, G-CSF) and osteopontin (OPN)

**Figure 4:**
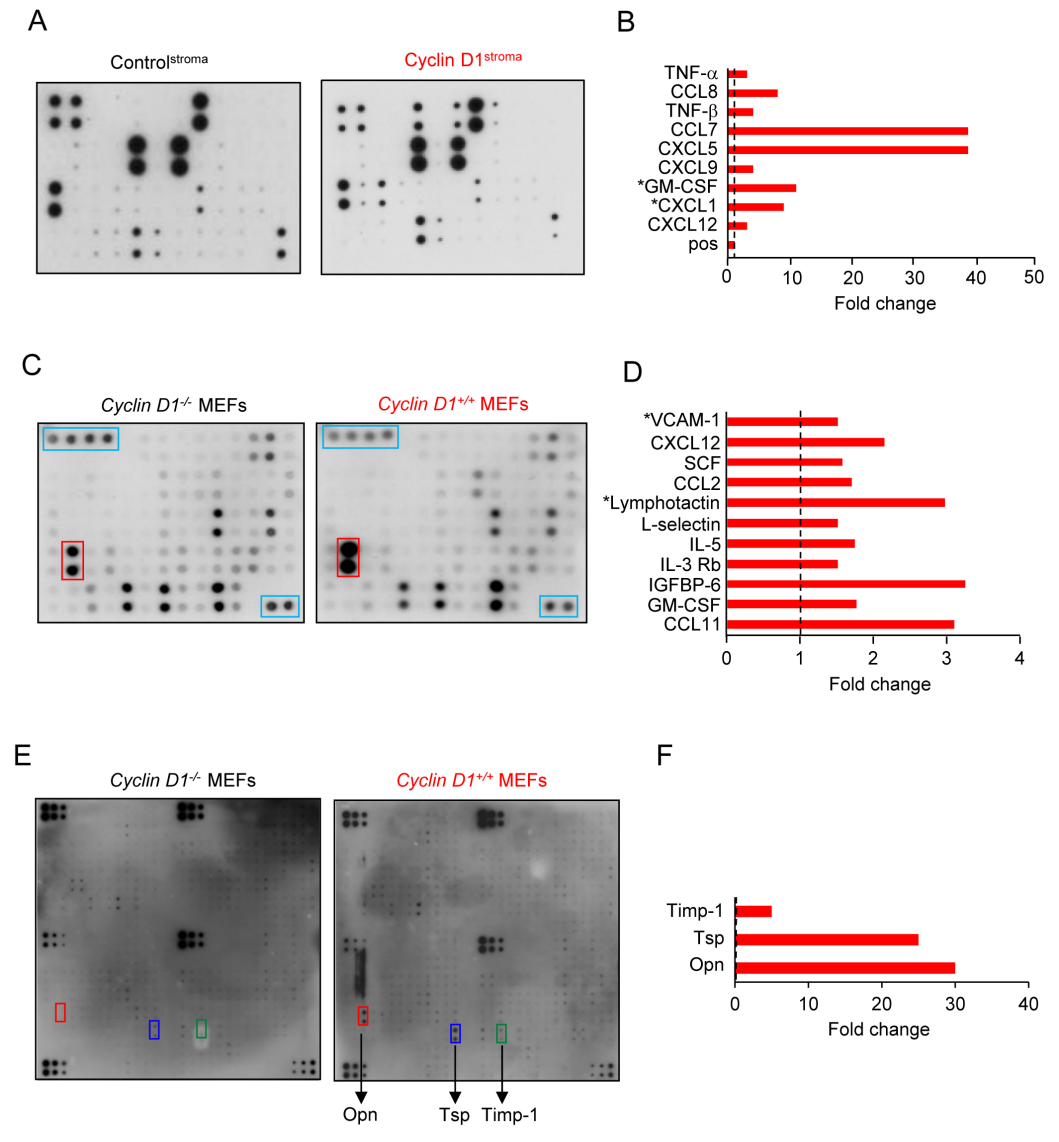
Fibroblast cyclin D1 expression increases secretion of inflammatory cytokines **A.** Soluble growth factor/cytokine/receptor array of conditioned medium from hTERT-control^stroma^ or hTERT-cyclin D1^stroma^ or **C.**-**F.**, from *cyclin D1*^*+/+*^ or *cyclin D1*^*-/-*^ MEFs. Data is shown as fold change.

In order to determine the mechanism by which cyclin D1^Stroma^ promoted MDA-MB-231 tumor inflammation and growth, we analyzed cyclin D1^Stroma^ secreted factors, measuring growth factors, cytokines and receptors (Figure 4A). Chemokines have been grouped into four subfamilies (CXC, CC, CX3C and C) on the basis of a structural cysteine motif found near the amino terminus. Comparing cyclin D1^Stroma^ and control^Stroma^ demonstrated the induction of CCL8 (MCP2, 8-fold), CCL7 (MCP3 39-fold), CXCL5 (39-fold), GM-CSF (10-fold), CXCL1 (9-fold), TNFα, and TNFβ (Figure 4B). In order to determine the role of endogenous cyclin D1 in maintaining production of secreted factors we compared *cyclin D1*^-/-^ with *cyclin D1*^+/+^ MEFs. Endogenous cyclin D1 maintained abundance of CCL11, GM-CSF, IGFBP-6, IL-3Rb, IL-5, XCL1 (lymphotactin), CCL2, SCF, SDF-1α, VCAM (Figure 4C, 4D) and osteopontin (OPN), (30-fold) (Figure 4E, 4F). Collectively these studies demonstrated that increased cyclin D1 in fibroblasts induces the abundance of proinflammatory cytokines and chemokines.

### Cyclin D1 conditioned medium proteomic analysis identified cytokines and pathways governing macrophage and dendritic cell maturation

**Figure 5:**
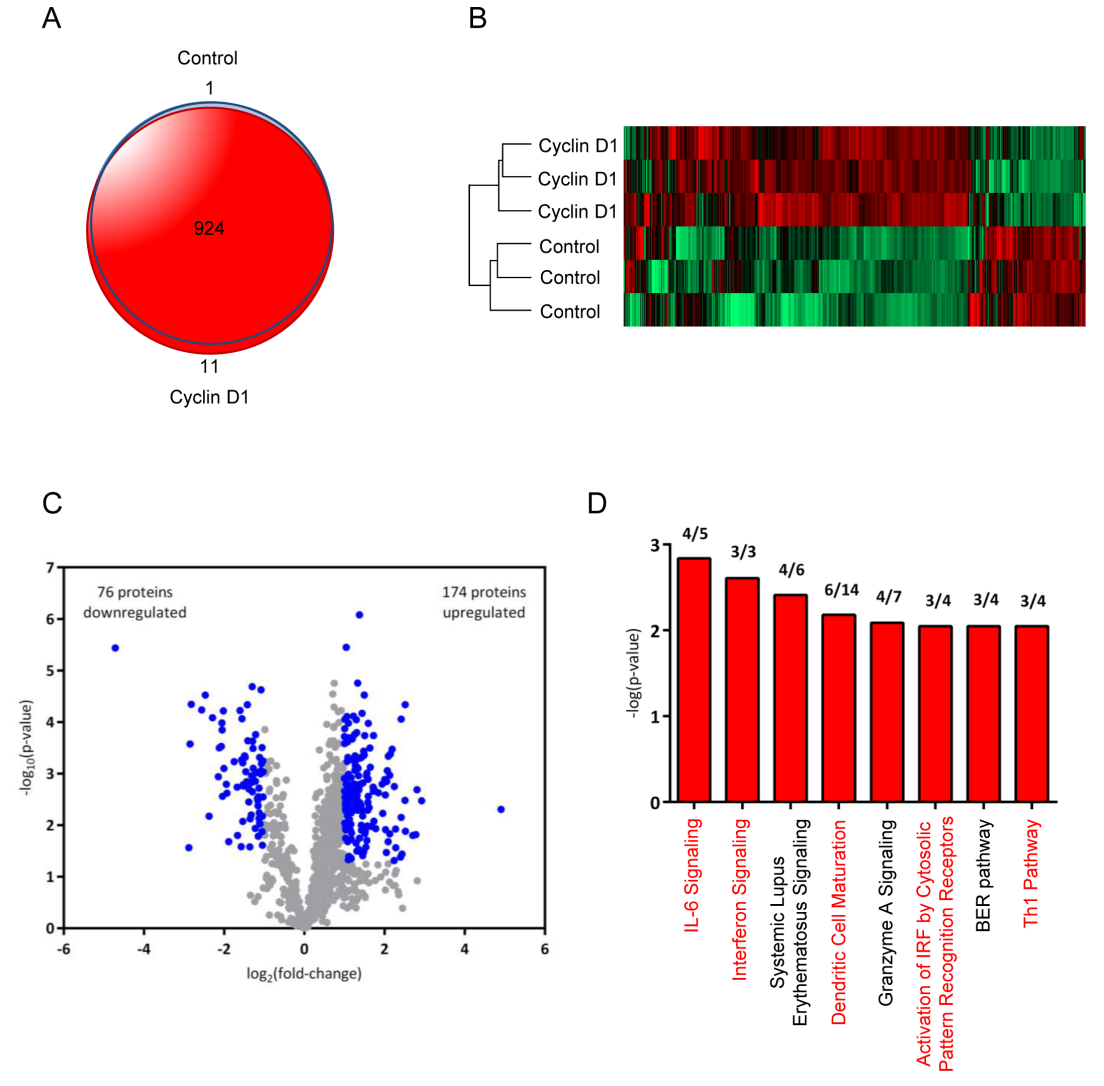
Proteome analysis of cyclin D1 conditioned medium identifies inflammatory cytokines and dendritic cell maturation pathways **A.** Venn diagram comparing the overlap of proteins in secretomes of hTERT-control^stroma^ and hTERT-cyclin D1^stroma^ cells (*n* = 3 for each condition). **B.** Hierarchical clustering heat map of hTERT-control^stroma^ and hTERT-cyclin D1^stroma^ secretomes based on LFQ intensities of identified proteins. **C.** Volcano plot of proteins identified in secretome analysis. Log2 ratios of LFQ intensities in hTERT-cyclin D1^stroma^ *vs*. hTERT-control^stroma^ were plotted against negative log10 Student’s *t*-test p-values. Significantly changed proteins, defined as absolute fold-change > 2 and *p* < 0.05, are shown in blue. **D.** Canonical pathways (Ingenuity Pathway Analysis) associated with secretome changes in hTERT-cyclin D1^stroma^ relative to hTERT-control^stroma^ are shown for a significance threshold of absolute Fold-change > 2.5 and Student’s *t*-test *p* < 0.05. Bars represent negative log10 Fisher’s exact test, right-tailed p-values for each canonical pathway. The ratio of proteins that significantly changed relative to the total identified proteins in this dataset for each pathway is listed above each bar.

To evaluate the extent to which cyclin D1 regulates the tumor microenvironment, we performed an unbiased global secretome analysis of conditioned media from cyclin D1^Stroma^ and control^Stroma^ hTERT cell lines by analyzing biological triplicates for each group (Supplementary Table 1). A total of 935 proteins were identified by two or more peptides (final protein FDR = 0.5%) with 924 proteins (98.8%) detected in both groups (Figure 5A). Protein levels in the secretomes were determined using label free quantitation. Biological replicates were highly similar, while abundance levels of many secreted proteins were different between the groups (Figure 5B). At a threshold of absolute fold-change greater than 2 and Student’s t-test p-value less than 0.05, 250 proteins (27% of identified proteins) were significantly different between the groups (Figure 5C) with 174 proteins upregulated and 76 downregulated in the cyclin D1^Stroma^ relative to control^Stroma^. Canonical pathways significantly associated with cyclin D1 were primarily associated with cytokines and immune response, including “IL-6 Signaling,” “Interferon Signaling,” “Dendritic Cell Maturation,” “Activation of IRF by Cytosolic Pattern Recognition Receptors,” and “Th1 Pathway” (Figure 5D). IL-6 was among the most upregulated proteins in cyclin D1^Stroma^ exhibiting a 7-fold increase (Supplementary Table 1). These results are consistent with a primary role for cyclin D1-regulated secreted factors in determining tumor cytokine signaling and the immune response. While some cytokines, osteopontin, and other proteins assayed by targeted approaches (Figure 4) were not identified in the proteome analysis, these proteins were most likely present at very low abundance levels that were below the detection threshold of the proteomics method used here.

### Cyclin D1 conditioned medium induces expansion of CD34 positive hematopoietic stem cells (HSCs) and differentiation of CD34 positive hematopoietic stem cells (HSCs) into dendritic cells

**Figure 6:**
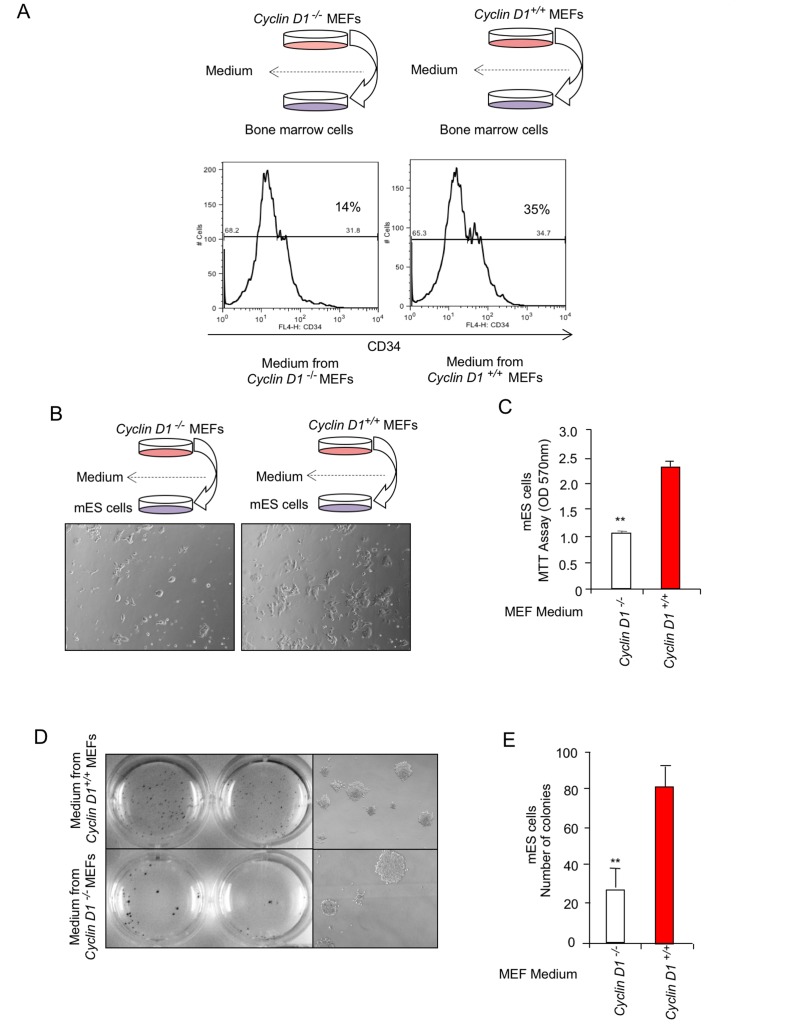
Cyclin D1 conditioned medium induces expansion of CD34 positive hematopoietic stem cells (HSCs) and promotes differentiation of CD34 positive hematopoietic stem cells (HSCs) into dendritic cells **A.** The bone marrow cells were cultured in media derived from *cyclin D1*^*-/-*^ or *cyclin D1*^*+/+*^ MEFs for 9 days and analyzed for CD34 expression by flow cytometry as described in Materials and Methods. Histogram represents the percentage of CD34^+^ cells. The gate was set based on unstained and/or isotype control for each sample. **B.**-**C.** The supernatant from *cyclin D1*^*-/-*^ or *cyclin D1*^*+/+*^ MEFs were incubated with ES cells and assessed for proliferation or **D.**,**E.** colony number. Quantitation of ES cell colony number shown as mean ± SEM for *N* = 3.

CD11b^+^ macrophages are thought to participate in the progression of breast tumorigenesis [46], therefore we sought to examine further the mechanism by which cyclin D1 in the stroma may promote the production of CD11b^+^ cells. Myeloid-derived suppressor cells (MDSC) are a heterogeneous population of cells which strongly inhibit anti-cancer immune responses and in the mouse are characterized by the expression of CD11b. Given the increase in CD11b in the cyclin D1^Stroma^, we examined the potential role of cyclin D1-induced fibroblast heterocellular signals in the expansion of a CD11b^+^ population using murine bone marrow derived cells. CD34 is a marker of hematopoietic stem cells (HSC) [47]. In order to examine the mechanisms governing the induction of the CD11b^+^ from the hematopoietic stem cells, the murine bone marrow cells were cultured with the supernatant from either *cyclin D1*^-/-^ or *cyclin D1*^+/+^ MEFs (Figure 6A). We used the standard HSC protocol with Flt3L media for 9 days and analyzed CD34 expression by flow cytometry. The histogram representing the percentage of CD34^+^ cells demonstrated that cyclin D1 conditioned- supernatant increased the percentage of CD34^+^ cells from 14% to 35% (Figure 6A).

In order to examined further the mechanisms by which cyclin D1-mediated secreted factors increased the proportion of CD11b^+^ cells, we investigated the potential role of the pRB protein. Comparison was therefore made of the secreted signals derived from *cyclin D1*^-/-^, *pRB*^-/-^ and *cyclin D1*/*pRB*^-/-^ MEFs. Western blot demonstrated loss of the cognate protein in the MEFs (Supplementary Figure 2A), maintenance of cellular diameter and reduced proliferation rates in the *cyclin D1*^-/-^ cells (Supplementary Figure 2B, 2C). The supernatant derived from similar numbers of MEFs of each genotype were then used in assays with the murine bone marrow (Supplementary Figure 3). The CD34^+^ population was reduced from 35% to 27% when incubated with *cyclin D1*^+/+^/*pRB*^-/-^ media, however the *cyclin D1*^-/-^/*pRB*^-/-^ media resulted in 23% CD34^+^ cells, which was greater than the proportion shown from the supernatant of *cyclin D1*^-/-^ MEFs. Together these studies suggest that endogenous cyclin D1-mediated secreted factors are the primary driver to expand CD34^+^ cells.

The increase in CD11b^+^ MDSC by media derived from *cyclin D1*^+/+^ vs *cyclin D1*^-/-^ MEFs is consistent with a role for endogenous fibroblast cyclin D1 in producing secreted factors that promote stem cell expansion. Traditional stem cell assays were therefore conducted, quantitating the number of embryonic stem cell colonies to determine the possibility that cyclin D1-mediated secreted factors promote stem cells. The supernatant was incubated with murine ES cells, and colony number determined. The conditioned medium from cyclin D1 expressing cells induced both mES cell proliferation and colony number (Figure 6B-6E).

### Cyclin D1 induces the secretion and cleavage of osteopontin (OPN)

**Figure 7:**
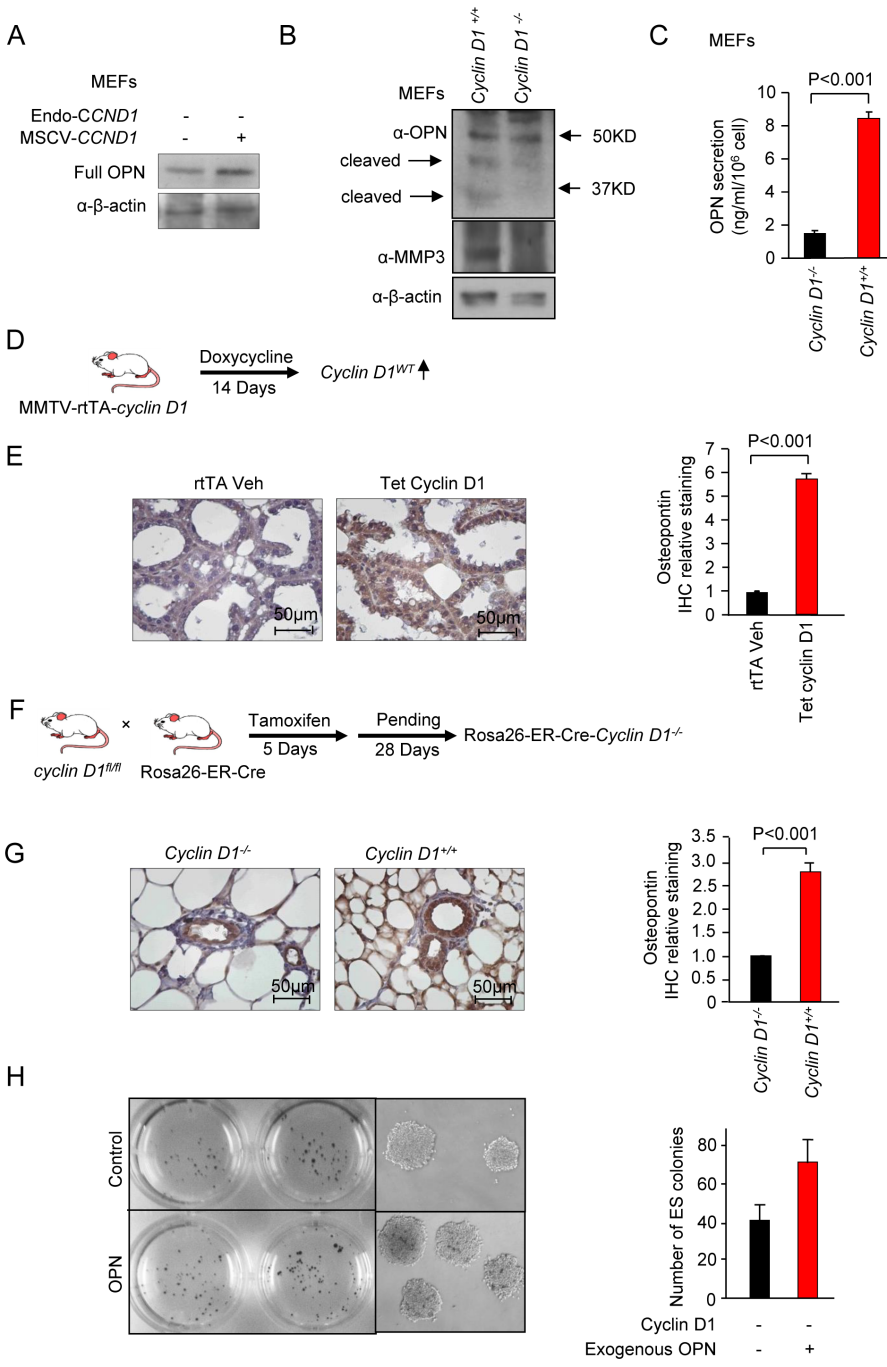
Cyclin D1 induces Osteopontin (OPN) abundance **A.** Western blot analysis for OPN in *cyclin D1*^*-/-*^ MEFs transduced with either control vector or a cyclin D1 expressing vector. β-actin is a loading control. **B.** Western blot analysis of either wild type or *cyclin D1*^*-/-*^ MEFs for the abundance of OPN and its bioactive cleaved forms, MMP3 (cleavge enzyme for OPN) and β-actin loading control. **C.** ELISA of OPN in cell culture media of either wild type or *cyclin D1*^*-/-*^ MEFs. The serum-free cell cuture media were collected after 48 hours. **D.** Schematic representation of transgenic mice in which a cyclin D1 cDNA was induced in the mammary gland upon addition of doxycycline. **E.** Immunohistochemistry for OPN in the mammary gland of transgenic mice with quantitation of IHC shown as mean + SEM for *N* = 3 separate mice. **F.** Schematic representation of transgenic paradigm to delete *cyclin D1* in mice using tamoxifen inducible Cre expression. **G.** Immunohistochemistry for OPN in the mammary gland of transgenic mice with quantitation of IHC shown as mean ± SEM for *N* = 3 separate mice. **H.** Assay of OPN mediated induction of embryonic stem (ES) cells. Image of the plates in which ES cell assays were conducted using either vehicle or OPN, with representative colonies shown at high magnification (200x). Quantitation of ES cell colony number shown as mean ± SEM for *N* = 3.

In our assessment of cyclin D1-mediated soluble factors, OPN was induced 30-fold (Figure 4F). Elevated levels of OPN correlate with poor prognosis in breast cancer [48]. OPN is a soluble pleiotropic cytokine that binds to integrin receptors, including α4β1, α9β1, and α9β4 expressed by leukocytes, to induce cell adhesion, migration, and survival in immune cells (neutrophils, macrophages, T cells, mast cells, and osteoclasts [49, 50]. Because OPN has the capacity to recapitulate many of the cyclin D1-mediated stromal effects, further analysis was conducted of cyclin D1 and OPN using cultured cells and transgenic mice. Cyclin D1 reintroduction into *cyclin D1*^-/-^ MEFs induced OPN abundance (Figure 7A). OPN is cleaved into active forms by thrombin and matrix melloproteases to form a C terminal fragment that is chemotactic to macrophages [51-53]. A comparison of *cyclin D1*^-/-^ and *cyclin D1*^+/+^ MEF showed an increased abundance of the OPN cleaved forms (Figure 7B). As cleavage is conducted by matrix metalloproteases (MMP-3, MMP-7) [54], we assessed the abundance of MMP by Western blot. MMP-3 was induced 7-fold in the *cyclin D1*^+/+^ cells (Figure 7B). This finding is consistent with recent studies in which cyclin D1 was shown to induce MMP gene expression in the mammary gland *in vivo* [1]. OPN abundance was determined by ELISA in the cell culture media of both of *cyclin D1*^+/+^ and *cyclin D1*^-/-^ MEFs. Cyclin D1 increased OPN secretion approximately 6-fold (Figure 7C). In order to determine whether cyclin D1 induces OPN *in vivo*, two transgenic paradigms were deployed. The first in which the abundance of cyclin D1 is induced through a tetracycline inducible operator in the mammary gland of transgenic mice [9] (Figure 7D). 14 days after the induction of the cyclin D1 transgene in the mammary gland, immunohistochemical staining showed a 6-fold induction of OPN by the cyclin D1^WT^ (Figure 7E).

In order to determine whether endogenous cyclin D1 maintained OPN abundance, we assessed transgenic mice in which the *cyclin D1* gene could be excised in the adult mammary gland. The inducible *cyclin D1* genetic deletion mice (ROSA26-Cre-ERT2-cyclin D1^fl/fl^) [55] were treated with tamoxifen for 5 days (Figure 7F), and after a subsequent 28 days immunohistochemistry was conducted for OPN. Mammary gland staining for OPN was reduced 3-fold in the *cyclin D1* genetic deletion mice (Figure 7G). In order to determine whether OPN as sufficient to induce the ES cell expansion demonstrated with cyclin D1 conditioned medium, ES cells assays were conducted in the presence of OPN or control. ES cell number was induced 1.75-fold (Figure 7H, (N = 2, P 0.06).

## DISCUSSION

The current studies demonstrate that cyclin D1 mRNA abundance is increased 30-fold in the stroma of patients with breast cancer. A quantitative analysis of stromal cyclin D1 protein in 914 patients demonstrated that increased nuclear stromal fibroblast expression of cyclin D1 predicted poor outcome. Previous publications did not report the presence of cyclin D1 protein in breast cancer stroma but focused on reporting the more abundant of cyclin D1 in cancer epithelial cells. In part, this could be due to lower sensitivity of the DAB chromogen staining used by these older publications compared to our immunofluorescence based staining. In general, chromogen (brightfield) stains block light and is hypo-luminescent and has a signal range of only one log. In contrast, fluorescent signals emit light and are hyper-luminescent with a signal range of 2-2.5 logs. Consistent with the functional significance of the increased cyclin D1 in the stroma, several target proteins known to be repressed by cyclin D1 (NRF1, mTTFA), were reduced in the stroma (data not shown).

Breast epithelial cell cyclin D1 protein overexpression is found in up to 50% of human breast cancers [56]. Increased cyclin D1 gene copy number is found in up to 20% of breast tumors suggesting that activation of cyclin D1 can occur via additional mechanisms, including transcriptional and post-transcriptional dysregulation. Prior studies had assessed the prognostic significance of nuclear cyclin D1 abundance in the breast cancer epithelial cell. These prior studies demonstrated that increased nuclear epithelial cell cyclin D1 abundance correlates with better prognosis [57]. In these studies cyclin D1 mRNA levels in the cyclin D1 “high group” were induced 1.8-fold [57]. Cyclin D1 amplification or overexpression however identified a subset of ERα+ breast cancers with worse prognosis [58], suggesting additional interactions modify cyclin D1 function. As cyclin D1 has been shown to promote cellular migration it will be of interest in future studies to determine whether stromal cyclin D1 correlates with increased tumor invasiveness. The current studies provide evidence for the independent prognostic significance of stromal cyclin D1 and suggest consideration be given to evaluation of cyclin D1 in the stroma of breast cancer when considering patient prognosis.

We determined the functional significance of stromal cyclin D1 through co-injecting hTERT fibroblasts expressing cyclin D1 (cyclin D1^Stroma^) or control GFP vector (control^Stroma^). Cyclin D1^Stroma^ enhanced breast tumor volume 93%, with a 2-fold increase in breast cancer epithelial cell proliferation and a 63% reduction in breast tumor epithelial cell apoptosis. The finding that stromal cyclin D1 increased tumor growth is consistent with previous findings, in which cyclin D1 was shown to be a marker of senescent stroma [59] and senescent stroma was shown to stimulate tumor growth [60].

In the current co-culture studies, cyclin D1^Stroma^ increased cellular proliferation, consistent with heterocellular signaling driving breast cancer tumor growth. Many of the growth factors and chemokines induced by cyclin D1^Stroma^ are capable of promoting breast cancer epithelial cell proliferation and reducing breast cancer epithelial apoptosis. It is likely that combinatorial interactions of the secreted signals participate in the growth phenotype. Cyclin D1^Stroma^ increased breast cancer epithelial cell autophagy, increasing the abundance of BCN1 and LAMP1. Increased tumor epithelial cell autophagy is thought to participate in tumor cell metabolic heterogeneity by driving epithelial cell catabolism and glycolysis [39]. The mammalian gene encoding Beclin1, a novel Bcl-2-interacting mammalian autophagy gene, can inhibit tumorigenesis and is expressed at decreased levels in human breast carcinoma. Cell surface expression of LAMP1 can serve as a ligand for selectins and help mediate cell-cell adhesion correlating with increased cancer invasiveness. The finding that the stromal cyclin D1 increases breast cancer autophagy contrasts with the cell autonomous function of cyclin D1 in tissue mono culture, wherein cyclin D1 restrains epithelial cells and fibroblast autophagy via phosphorylation of LKB1[61]. Thus cyclin D1 conveys tissue compartment specific effects, with stromal fibroblast cyclin D1 increasing autophagy, and epithelial cell cyclin D1 restraining autophagy.

In the current studies, cyclin D1 expression in fibroblasts determined a gene expression profile that overlapped with the previously described expression pattern associated of cancer-associated fibroblasts [34, 62]. Of the 126 CAF-induced genes, 20 were also induced by cyclin D1. Of the 14 functional terms induced in CAFs, 9 functional terms were also induced by cyclin D1. Cyclin D1^Stroma^ induced an inflammatory infiltration in the breast cancers with increased infiltration of CD11b^+^ macrophages. In mice, MDSCs are defined as being CD11b^+^ and Gr-1^+^. Canonical pathways significantly associated with cyclin D1 mediated secreted proteins were associated with “Dendritic Cell Maturation,” and cyclin D1 conditioned medium induced expansion of CD34 positive hematopoietic stem cells (HSCs) and differentiation of CD34 positive hematopoietic stem cells (HSCs) into dendritic cells. The current studies are consistent with a role for cyclin D1^Stroma^, in the induction of stem cells. Cyclin D1-mediated heterotypic factors increased the number of bone marrow derived stem cells (BMDSC) and increased embryonic stem cell (ESC) colonies 3-fold. We demonstrated that full length OPN enhanced the number of ES colonies approximately 2-fold. Several cyclin D1-mediated secreted factors have the capacity to differentiate MDSC, including granulocyte macrophage-colony stimulating factor (GM-CSF), macrophage-colony stimulating factor (M-CSF), and stem cell factor (SCF) [63, 64]. Cyclin D1 induced GM-CSF 1.75-fold and SCF 1.5-fold. We identified cyclin D1 at the regulatory region of the *Csf1* gene in ChIP-seq (Supplementary Figure 4). As myeloid-derived suppressor cells (MDSCs) accumulate in tumors during malignant progression and in ex vivo CTL assays can suppress T cell responses [65] and block DC maturation at the invasive edge of tumors [65], the current studies suggest a novel mechanism by which stromal cyclin D1 may augment tumor progression.

What might be the functional significance of the cyclin D1-stroma–mediated induction of F4/80^+^ macrophages ? Monocytes and/or macrophages promote the extravasation, survival, and persistent growth of metastatic cells [66]. The role of M1 vs M2 macrophages, and markers used to define these populations remains controversial. However iNOS is produced by macrophages, and increased stromal iNOS correlated with mammary tumor progression [67]. In the current studies increased iNOS staining of macrophages was observed in the cyclin D1 stroma tumors *in vivo* (data not shown), consistent with a protumorigenic role of the cyclin D1 stromal-augmented tumor inflammation.

Increased expression of fibroblast cyclin D1 induced the abundance of inflammatory chemokines/cytokines in fibroblasts, that are known to promote tumorigenesis. Cyclin D1 conditioned medium produced pro-inflammatory cytokines (CCL2, CCL7, CCL11, CXCL1, CXCL5, CXCL9, CXCL12), CSF (CSF, G-CSF) and osteopontin (OPN). Several of the individual components of the secretome, induced by cyclin D1^Stroma^, were previously described as tumor stroma secreted factors. In addition to cytokines/chemokines, proteomic analysis showed increased secretion of 176 proteins in response to cyclin D1 expression, that correlated with functional pathways including “IL-6 signaling”, “interferon signaling”, and “dendritic cell maturation”. Endogenous cyclin D1 maintained abundance of stromal cell-derived factor 1 (SDF-1α), also called CXCL12, which plays a central role in the promotion of tumor growth and angiogenesis. SDF-1α stimulates carcinoma cell growth directly through binding the CXCR4 receptor on tumor epithelial cells and augments neoangiogenesis through recruiting endothelial progenitor cells (EPCs) [68]. Endogenous cyclin D1 also maintained the abundance of CCL2, which is known to enhance the retention of metastasis-associated macrophages [69].

How might cyclin D1 induce the abundance of inflammatory chemokines/cytokines in fibroblasts? Cyclin D1 regulates transcription, both in cultured cells [70] and *in vivo* [1, 71], through interacting in the context of chromatin with target transcription factors [9, 72] recruiting histone regulatory enzymes [70, 73] thereby regulating transcription in a kinase-independent manner [55]. Herein, cyclin D1 was recuited in the context of chromatin to the promoter regulatory regions of several cytokine/chemokine genes that were induced by cyclin D1, including *Csf, Cxcl1, Xcl*, and Vcam1 (Supplementary Figure 4). Therefore cyclin D1 may induce cytokine/chemokine expression via this well established transcriptional mechanism.

The mechanisms governing the induction of cyclin D1 in the stroma of breast cancer patients is not known. Genetic deletion studies have identified components of the aberrant signaling pathway in CAFs. In recent studies endogenous c-Myc was also shown to participate in the development of the inflammatory stromal environment [74]. Deletion of the *JunD* gene in the stroma promoted tumor growth and metastasis, associated with increased ROS production, with consequent accumulation of HIF1α and HIF1α-dependent induction of CXCL12, activating CXCR4, promoting CAF differentiation [75]. Hypoxia-induced cancer cells derived TGFβ also drives CAF differentiation [68, 76]. Stromal Cav-1 is frequently degraded upon hypoxemic induced lysosomal degradation and loss of *Cav-1* is sufficient to drive the CAF genotype. Cav-1 mediates anti-proliferative and survival function via the *cyclin D1* gene, which is transcriptionally repressed by Cav1 [77] via a β-catenin/Tcf pathway [78]. CAF gene expression analysis evidence increased expression of cell-cycle pathways, increased phosphorylation of pRB and loss of Cav1 was highly correlated with poor response to tamoxifen therapy [34]. The possibility the hypoxia induces degradation of Cav-1 and consequently induces cyclin D1 in the breast cancer stroma remains to be investigated.

## MATERIALS AND METHODS

### Gene expression analysis

The abundance of stromal cyclin D1 mRNA in breast tumors was determined by Oncomine, a cancer-profiling database. A cut-off of 1.5-fold enrichment and a p-value of <0.05 were used to interrogate the cancer-profiling database (Oncomine™ Research Edition, https://www.oncomine.org). Comparison of microarray data sets representing cyclin D1-dependent and cancer-associated fibroblast (CAF) upregulated genes was conducted using published data sets [34, 62]. Statistical significance for overlapping cyclin D1 induced and CAF-dependent genes and Gene Ontology (GO) PANTHER terms were calculated using the hypergeometric distance statistical formula. The significance level for P values of gene set overlap and GO functions was established as P<0.01.

### Materials

Antibodies were directed to Ki-67 (AB16667, Abcam), calponin (SC136987, Santa Cruz), BECN1 (sc-11427, Santa Cruz), LC3B (#3868, Cell Signaling), LAMP1 (sc-17768, Santa Cruz), F4/80 (ab6640, Abcam), CD11b (PA5-18727, Invitrogen), Sca1 (ab-51317, Abcam), osteopontin (ab8488, Abcam), LC3A/B (ab58610, Abcam). Secondary antibodies for immunofluorescence were Alexa Green 488 nm and Alexa Orange-Red 546 nm (Invitrogen). 4,6-diamidino-2-phenylindole (DAPI) (D3571), Prolong Gold Anti-fade mounting reagent (P36930), Slow-Fade Anti-fade reagent (S2828). Osteopontin was obtained from SIGMA (Cat. # O2260).

### Cell culture

Human skin fibroblasts, immortalized with telomerase reverse transcriptase protein (hTERT-BJ-1) were originally purchased from Clontech, Inc. and transduced with either MSCV-cyclin D1 or MSCV-GFP as previously described [8]. The breast cancer cell line MDA-MB-231 was purchased from ATCC. The hTERT-BJ-1 and MDA-MB-231 cell lines were obtained in early 2000. Mouse embryonic fibroblasts (MEFs) of distinct genotypes (*cyclin D1*^-/-^*, pRB*^-/-^*, cyclin D1*^-/-^*/pRB*^-/-^) were prepared and cultured as previously described [79]. The MEFs of *cyclin D1*^-/-^*, pRB*^-/-^*, cyclin D1*^-/-^*/pRB*^-/-^ and wildtype control were generated in 2006 and 2011. All cells were maintained in DMEM with 10% Fetal Bovine Serum (FBS) and Penicillin 100 units/mL-Streptomycin 100 µg/mL. Mouse embryonal stem (ES) cells were cultured following the standard protocol [80]. Briefly, tissue culture plates were coated with 0.1% (v/v) porcine gelatin (Sigma-Aldrich Corp, St. Louis, MO) in Dulbecco’s modified Eagle’s medium (DMEM; Invitrogen) in the presence of 15% fetal bovine serum (ES-tested Hyclone, Perbio, Logan, UT), 0.1 mM 2-β-mercaptoethanol, 0.1 mM non-essential amino acids, 2 mM glutamine, 0.1 mM sodium pyruvate, and 1,000 units/ml murine LIF (Chemicon International Inc., Temecula, CA). The cell culture medium was changed daily.

The early passage MEFs were stored in liquid nitrogen. The cells thawed from low passage stocks were used within one month of the initial thaw. During the experiments, the morphology of all cell lines was routinely checked under phase contrast microscopy. All of the newly revived cells were treated with BM-cyclins (Roche) and the presence of possible mycoplasma contamination was routinely excluded with Hoechst 33258 staining under high magnification fluorescent microscope.

### Cell co-culture

hTERT-fibroblasts and MDA-MB-231 cells were plated on glass coverslips in 12-well plates in 1-ml of complete media. MDA-MB-231 cells were plated within 2 hours of fibroblast plating. The total number of cells per well was 1 × 10^5^. Experiments were performed at a 5:1 fibroblast-to epithelial cell ratio. As controls, monocultures of fibroblasts and MDA-MB-231 cells were seeded using the same number of cells as the corresponding co-cultures. The day after plating, media was changed to DMEM with 10% NuSerum (a low protein alternative to FBS; BD Biosciences) and Penicillin-Streptomycin. Cells were maintained at 37°C in a humidified atmosphere containing 5% CO_2_.

### Immunocytochemistry

Immunocytochemistry was performed as previously described [81]. All steps were performed at room temperature. Briefly, after 30 minutes fixation in 2% paraformaldehyde, cells were permeabilized for 10 minutes with immunofluorescence (IF) buffer (PBS, 0.2% BSA, 0.1% TritonX-100). Then, cells were incubated for 10 minutes with NH_4_Cl in PBS to quench free aldehyde groups. Primary antibodies were incubated in IF buffer for 1 hour. After washing with IF buffer (3x, 10 minutes each), cells were incubated for 30 minutes with fluorochrome-conjugated secondary antibodies diluted in IF buffer. Finally, slides were washed with IF buffer (3x, 10 minutes each), incubated with the nuclear stain and mounted.

### Flow cytometric analysis

GFP^+^ MDA-MB-231 cells were plated in co-culture with hTERT-fibroblasts or in mono-culture. The day after, media was changed to DMEM with 10% NuSerum and cells were grown for an additional 48 hours. Then, to isolate the GFP^+^ MDA-MB-231 cell population, co-cultured cells were sorted using a 488 nm laser. As a critical control, mono-cultures of GFP^+^ MDA-MB-231 cells were sorted in parallel. For DNA content analyses, sorted cells were fixed in 70% ethanol overnight at 4°C and stained with PI. DNA cell content was analyzed by flow cytometry. For proliferation analysis, cells were incubated with BrdU (Amersham Pharmacia Biotech) for one hour before sorting. Cells were washed in PBS, fixed in cold 70% ethanol and flow cytometry was employed for analysis of nascent DNA synthesis (BrdU incorporation). Cell cycle analysis was performed using FlowJo 8.8 software. BrdU data was represented as a percentage of the total population.

### CD34^+^ hematopoietic stem cell differentiation into bone marrow-derived dendritic cells

The effect of stromal cyclin D1-mediated secreted factors was tested on the differentiation of CD34^+^ hematopoietic stem cells (HSCs) into bone-marrow derived dendritic cells (BMDCs) as described previously [82, 83]. Briefly, 30 × 10^6^ BM cells were obtained from 6- to 10-week-old C57BL/6J mice (purchased from The Jackson Laboratory, Bar Harbor, ME) cultured in medium obtained from MEF derived from wild type, *cyclin D1*^-/-^ or *pRB*^-/-^, or *cyclin D1*^-/-^/*pRB*^-/-^ animals and supplemented with 20% of Flt3L, (Fms-related tyrosine kinase 3 ligand), containing supernatant, produced from an SP2/0 transfected cell line that secretes murine recombinant Flt3L [84]. These cells were kindly provided by Dr. Robert Rottapel, Ontario Cancer Institute, Toronto Medical Discovery Tower, Toronto, Canada. The cells were maintained undisturbed at 37°C in 5% CO_2_ and 90% relative humidity. On day 9, the cells were collected by gentle trypsinization and phenotyped for marker of stem cells (CD34) as well as myeloid (CD11c^+^/CD11b^+^/B220^-^) and plasmacytoid (CD11c^+^/CD11b^-^/B220^+^) dendritic cell markers, using specific antibodies for flow cytometry as described [83].

### Apoptosis studies

Terminal deoxynucleotidyl transferase dUTP nick end labeling (TUNEL) (TACS2 TdT DAB, Trevigen) staining was used as a measure of apoptosis in tissues as previously described [85]. Apoptosis was quantified by Flow Cytometry using the Annexin V-Cy5 apoptosis detection kit, as per the manufacturer’s instructions (Abcam).

### Western blotting

hTERT-vector and hTERT-cyclin D1 fibroblasts were harvested in lysis buffer (10 mM Tris-HCl pH 7.5, 150 mM NaCl, 1% Triton X-100, 60 mM octylglucoside), containing protease inhibitors (Roche Applied Science) and phosphatase inhibitors (Roche Applied Science) and centrifuged at 13,000x g for 15 min at 4°C to remove insoluble debris. After centrifugation to remove insoluble debris, protein concentration was determined using the Bradford assay (BioRad). 30 µg of proteins were loaded and separated by SDS-PAGE and transferred to a 0.2 µm nitrocellulose membrane (Fisher Scientific). After blocking for 30 min in TBST (10 mM Tris-HCl pH 8.0, 150 mM NaCl, 0.05% Tween-20) with 5% nonfat dry milk, membranes were incubated with the primary antibody for 1 hour, washed and incubated for 30 min with horseradish peroxidase-conjugated secondary antibodies. The membranes were washed and incubated with an enhanced chemi-luminescence substrate (ECL;Thermo Scientific).

### Osteopontin ELISA

1.2 × 106 of cells in 2 ml DMEM with 10% FBS were seeded in 12-well plate and cultured overnight in CO_2_ incubator at 37°C. The media was then changed to FBS-free DMEM media and cultured for another 48 hours. The culture media was collected and centrifuged at 4000 rpm to remove the cell debris. OPN in cultured media was measured by Mouse/Rat Osteopontin (OPN) Quantikine ELISA Kit from R&D Systems based on the manufacture’s protocol.

### Animal studies

The appropriate Thomas Jefferson University institutional committee approved protocols were followed when working with all mice. Animals were housed and maintained in a pathogen-free environment/barrier facility of Thomas Jefferson University under National Institutes of Health (NIH) guidelines. Mice were kept on a 12 hours light/dark cycle with *ad libitum* access to chow and water. Approval for all animal protocols used for this study was reviewed and approved by the Institutional Animal Care and Use Committee. The tetracycline inducible mammary epithelial cell targeted cyclin D1 transgenic mice (MMTV-rtTA-cyclin D1^WT^) and the tamoxifen inducible *cyclin D1* genetic deletion mice (ROSA26-Cre-ERT2-cyclin D1^fl/fl^) were previously described [55].

In brief, transgenic founder lines were backcrossed with wild type FVB mouse for three generations to obtain the stably inherited transgene line, followed by cross mating with MMTV-rtTA line (a kind gift from Dr. Lewis Chodosh’s lab) to obtain cyclin D1^+/+^/rtTA^+/+^ mice. 8-week-old tetracycline-inducible cyclin D1/rtTA bi-transgenic pregnant female mice (12 days postcoitus) were administered doxycycline in the drinking water (2 mg/ml). After 7 days of doxycycline treatment, the mice were sacrificed and mammary glands extracted for analysis. Conditional *cyclin D1*^fl/fl^ mice were a generous gift from Dr. Sicinski. C57BL/6J ROSA26-Cre-ERT2 mice were a kind gift from Dr. Streamson C. Chua Jr. ROSA26-Cre-ERT2-*cyclin D1*^fl/fl^ mice were generated by crossing *cyclin D1*^fl/fl^ and ROSA26-Cre-ERT2 mice. *Ccnd1* knock-out mice were generated by a daily intra-peritoneal injection of Tamoxifen (1 mg/200 µl) for 5 days, followed by a wash out period of four weeks. Mammary gland tissues were then collected. Wild type Cre-ERT2 mice were used as control.

For breast tumor implantation studies, tumor cells (MDA-MB-231 (GFP^+^; 1 × 10^6^ cells) were mixed with hTERT-fibroblasts (3 × 10^5^ cells) in 100 µl of sterile PBS and were co-injected into the flanks of athymic NCr nude mice (NCRNU; Taconic Farms; 6–8 weeks of age). After 4 weeks, mice were sacrificed; tumors were excised and frozen in liquid nitrogen-cooled isopentane.

### Survival studies

For statistical analysis, cyclin D1 expression in stroma, (high vs low), was derived from data-driven optimal cutpoint analysis using X-tile software [36, 86, 87]. Breast cancer recurrence-free survival was analyzed using the Kaplan-Meier survival curve estimator, log-rank test and Cox proportional hazards model. Nuclear-localized cyclin D1 levels were compared in normal or malignant tissue using t-test.

### Paraffin-embedded breast tumor tissues

Breast cancer tissue microarrays were constructed from formalin-fixed, paraffin-embedded unselected tumor specimens from Thomas Jefferson University Hospital pathology archives obtained under IRB approved protocols. Patients were diagnosed between the years 1981 and 2005 with median clinical follow-up data of 8.5 years.

### Quantitative immunofluorescence

Stromal cyclin D1 was detected in clinical breast cancer specimens by immunofluorescence-immunohistochemistry (IF-IHC) performed on an Autostainer Plus (Dako) as previously described [87] with the following specifications: antigen retrieval used the DAKO PT-module with citric acid buffer (pH 6.0) and rabbit monoclonal cyclin D1 antibody (Dako, M3635) was diluted 1:200 and coincubated with mouse monoclonal anti-pancytokeratin (clone AE1/AE3, DAKO, 1:100) for 45 minutes.

Quantitative analysis of cyclin D1 was performed as previously described [86]{Peck, 2016 #603;Peck, 2016 #230} using the ScanScope FL line scanner (Leica Biosystems) to capture high-resolution digital images followed by quantification of cyclin D1 levels in the stroma of tumor specimens using Tissue Studio image analysis software (Definiens). Briefly, user-guided machine learning was performed to generate an analysis solution that identified DAPI-stained cell nuclei within the stroma of each tumor specimen. Mean nuclear cyclin D1 signal intensity was calculated for stromal cells in each tumor core.

### Cytokine arrays

Human cytokine arrays (ab133997) were obtained from Abcam (Cambridge, MA). The medium of hTERT-control^stroma^ and hTERT-cyclin D1^stroma^ was prepared by culturing cells in DMEM with 10% FBS for 48 hrs. Mouse cytokine arrays were purchased from Raybiotech (Norcross, GA). Conditioned medium of *cyclin D1*^+/+^ and *cyclin D1*^−/−^ MEFs was prepared by culturing cells in serum free DMEM for 48 hrs. The experiments were conducted exactly as described in the manufacture’s protocol.

### Proteome analysis of cyclin D1 secretome

Conditioned serum-free media from hTERT-cyclin D1 and control hTERT fibroblasts was centrifuged and filtered through a 0.22 µm membrane to remove cell debris and protease inhibitors were added (1 mM PMSF, 1 µg/ml leupeptin, and 1 µg/ml pepstatin. Samples were concentrated on 3 KDa MWCO Amicon Ultra-4 centrifugal filter units and membranes were washed with 1% SDS, 10 mM Tris, pH 7.4 to extract adsorbed proteins (70-fold final concentration), followed by separation for 0.5 cm on 10% Bis-Tris NuPAGE gels (Thermo Fisher Scientific). In-gel trypsin digestion was performed as described previously [88] with the following changes: trypsin was used at 4 ng/µl and digests were carried out for 4 hr at 37 °C. Digested samples were lyophilized, resuspended in 0.1% formic acid, 4% acetonitrile immediately prior to LC-MS/MS using a Q Exactive Plus mass spectrometer (Thermo Fisher Scientific) as previously described [89]. Briefly, peptides were separated by reverse-phase using a 180 µm × 20 mm nanoACQUITY UPLC Symmetry C18 trap column and a 1.7 µm × 250 mm nanoACQUITY UPLC Peptide BEH C18 column with 1.7 µm particles (Waters) using a 245 min gradient. Data-dependent analysis was performed on the 20 most abundant ions using an isolation width of 1.5 m/z with a 30 sec dynamic exclusion window. Peptide match was preferred, and unassigned and single charge ions were rejected. Data were analyzed by MaxQuant 1.5.1.2 [90] and the built-in Andromeda search engine using the UniProt human sequence database (July 28, 2014; 145,433 sequences) and an in-house database of common contaminants, such as keratins, trypsin, and media components (from UniProt, July 28, 2014; 3,671 sequences). False discovery rates (FDR) for peptides and proteins were set to 1% for the initial output. Matching between runs was performed with a 0.7 min match window and 20 min alignment window. Label-free quantitation (LFQ) was performed with a minimum peptide ratio of 1 required for normalization. MaxQuant results were processed using Perseus version 1.5.0.31 [91] and Microsoft Excel 2016. Contaminants and protein groups identified by a single peptide were removed from the dataset which reduced the protein FDR from 1% to 0.5% in the final dataset. LFQ intensities were log2 transformed, and missing values were imputted from a median downshifted Gaussian distribution with a width of 0.3 and downshift of 1.8. Hierarchical clustering was performed on Z-scored, log2-transformed LFQ intensities using Euclidean distance with k-means preprocessing (300 clusters). Log2 ratios were calculated as the difference in log2 LFQ intensity averages between cyclin D1 and control secretomes. Two-tailed, unpaired, homoscedastic Student’s t-test calculations were performed for pairwise comparisons because the datasets approximated normal distributions. Fold-change values for ratios less than 1 are represented as negative reciprocals of the ratios. Canonical pathway analysis was performed using Ingenuity Pathway Analysis (QIAGEN) core analysis. Proteins were mapped to Ingenuity Knowledgebase identifiers by gene symbol. The entire list of identified proteins was used as the reference in statistical calculations.

## SUPPLEMENTARY MATERIALS FIGURE AND TABLE





## Abbreviations

CAFs: cancer-associated fibroblasts
hTERT: human telomerase reverse transcriptase

## References

[1] Casimiro MC, Wang C, Li Z, Di Sante G, Willmart NE, Addya S, Chen L, Liu Y, Lisanti MP, Pestell RG (2013). Cyclin D1 determines estrogen signaling in the mammary gland in vivo. Mol Endocrinol.

[2] Lee RJ, Albanese C, Fu M, D’Amico M, Lin B, Watanabe G, Haines GK, Siegel PM, Hung MC, Yarden Y, Horowitz JM, Muller WJ, Pestell RG (2000). Cyclin D1 is required for transformation by activated Neu and is induced through an E2F-dependent signaling pathway. Mol Cell Biol.

[3] Yu Q, Geng Y, Sicinski P (2001). Specific protection against breast cancers by cyclin D1 ablation. Nature.

[4] Hulit J, Wang C, Li Z, Albanese C, Rao M, Di Vizio D, Shah S, Byers SW, Mahmood R, Augenlicht LH, Russell R, Pestell RG (2004). Cyclin D1 genetic heterozygosity regulates colonic epithelial cell differentiation and tumor number in ApcMin mice. Mol Cell Biol.

[5] Neumeister P, Pixley FJ, Xiong Y, Xie H, Wu K, Ashton A, Cammer M, Chan A, Symons M, Stanley ER, Pestell RG (2003). Cyclin D1 governs adhesion and motility of macrophages. Mol Biol Cell.

[6] Li Z, Jiao X, Wang C, Shirley LA, Elsaleh H, Dahl O, Wang M, Soutoglou E, Knudsen ES, Pestell RG (2010). Alternative cyclin D1 splice forms differentially regulate the DNA damage response. Cancer Res.

[7] Yu Z, Wang L, Wang C, Ju X, Wang M, Chen K, Loro E, Li Z, Zhang Y, Wu K, Casimiro MC, Gormley M, Ertel A (2013). Cyclin D1 induction of Dicer governs microRNA processing and expression in breast cancer. Nat Commun.

[8] Wang C, Pattabiraman N, Zhou JN, Fu M, Sakamaki T, Albanese C, Li Z, Wu K, Hulit J, Neumeister P, Novikoff PM, Brownlee M, Scherer PE (2003). Cyclin D1 repression of peroxisome proliferator-activated receptor gamma expression and transactivation. Mol Cell Biol.

[9] Casimiro MC, Crosariol M, Loro E, Ertel A, Yu Z, Dampier W, Saria EA, Papanikolaou A, Stanek TJ, Li Z, Wang C, Fortina P, Addya S (2012). ChIP sequencing of cyclin D1 reveals a transcriptional role in chromosomal instability in mice. J Clin Invest.

[10] Hanahan D, Weinberg RA (2011). Hallmarks of cancer: the next generation. Cell.

[11] Basset P, Bellocq JP, Wolf C, Stoll I, Hutin P, Limacher JM, Podhajcer OL, Chenard MP, Rio MC, Chambon P (1990). A novel metalloproteinase gene specifically expressed in stromal cells of breast carcinomas. Nature.

[12] Chiquet-Ehrismann R, Mackie EJ, Pearson CA, Sakakura T (1986). Tenascin: an extracellular matrix protein involved in tissue interactions during fetal development and oncogenesis. Cell.

[13] Singer C, Rasmussen A, Smith HS, Lippman ME, Lynch HT, Cullen KJ (1995). Malignant breast epithelium selects for insulin-like growth factor II expression in breast stroma: evidence for paracrine function. Cancer Res.

[14] Wright JH, McDonnell S, Portella G, Bowden GT, Balmain A, Matrisian LM (1994). A switch from stromal to tumor cell expression of stromelysin-1 mRNA associated with the conversion of squamous to spindle carcinomas during mouse skin tumor progression. Mol Carcinog.

[15] Yee D, Rosen N, Favoni RE, Cullen KJ (1991). The insulin-like growth factors, their receptors, and their binding proteins in human breast cancer. Cancer Treat Res.

[16] Orimo A, Weinberg RA (2006). Stromal fibroblasts in cancer: a novel tumor-promoting cell type. Cell Cycle.

[17] Mishra PJ, Mishra PJ, Humeniuk R, Medina DJ, Alexe G, Mesirov JP, Ganesan S, Glod JW, Banerjee D (2008). Carcinoma-associated fibroblast-like differentiation of human mesenchymal stem cells. Cancer Res.

[18] Mueller L, Goumas FA, Affeldt M, Sandtner S, Gehling UM, Brilloff S, Walter J, Karnatz N, Lamszus K, Rogiers X, Broering DC (2007). Stromal fibroblasts in colorectal liver metastases originate from resident fibroblasts and generate an inflammatory microenvironment. Am J Pathol.

[19] Petersen OW, Nielsen HL, Gudjonsson T, Villadsen R, Rank F, Niebuhr E, Bissell MJ, Rønnov-Jessen L (2003). Epithelial to mesenchymal transition in human breast cancer can provide a nonmalignant stroma. Am J Pathol.

[20] Zeisberg EM, Potenta S, Xie L, Zeisberg M, Kalluri R (2007). Discovery of endothelial to mesenchymal transition as a source for carcinoma-associated fibroblasts. Cancer Res.

[21] Rønnov-Jessen L, Petersen OW, Koteliansky VE, Bissell MJ (1995). The origin of the myofibroblasts in breast cancer. Recapitulation of tumor environment in culture unravels diversity and implicates converted fibroblasts and recruited smooth muscle cells. J Clin Invest.

[22] Schor SL, Schor AM, Grey AM, Rushton G (1988). Foetal and cancer patient fibroblasts produce an autocrine migration-stimulating factor not made by normal adult cells. J Cell Sci.

[23] Ellis MJ, Singer C, Hornby A, Rasmussen A, Cullen KJ (1994). Insulin-like growth factor mediated stromal-epithelial interactions in human breast cancer. Breast Cancer Res Treat.

[24] Frazier KS, Grotendorst GR (1997). Expression of connective tissue growth factor mRNA in the fibrous stroma of mammary tumors. Int J Biochem Cell Biol.

[25] Nakamura T, Matsumoto K, Kiritoshi A, Tano Y, Nakamura T (1997). Induction of hepatocyte growth factor in fibroblasts by tumor-derived factors affects invasive growth of tumor cells: in vitro analysis of tumor-stromal interactions. Cancer Res.

[26] Pontén F, Ren Z, Nistér M, Westermark B, Pontén J (1994). Epithelial-stromal interactions in basal cell cancer: the PDGF system. J Invest Dermatol.

[27] Yan G, Fukabori Y, McBride G, Nikolaropolous S, McKeehan WL (1993). Exon switching and activation of stromal and embryonic fibroblast growth factor (FGF)-FGF receptor genes in prostate epithelial cells accompany stromal independence and malignancy. Mol Cell Biol.

[28] Erez N, Truitt M, Olson P, Arron ST, Hanahan D (2010). Cancer-Associated Fibroblasts Are Activated in Incipient Neoplasia to Orchestrate Tumor-Promoting Inflammation in an NF-kappaB-Dependent Manner. Cancer Cell.

[29] Dong-Le Bourhis X, Berthois Y, Millot G, Degeorges A, Sylvi M, Martin PM, Calvo F (1997). Effect of stromal and epithelial cells derived from normal and tumorous breast tissue on the proliferation of human breast cancer cell lines in co-culture. Int J Cancer.

[30] Skobe M, Fusenig NE (1998). Tumorigenic conversion of immortal human keratinocytes through stromal cell activation. Proc Natl Acad Sci USA.

[31] Noy R, Pollard JW (2014). Tumor-associated macrophages: from mechanisms to therapy. Immunity.

[32] Martinez-Outschoorn UE, Trimmer C, Lin Z, Whitaker-Menezes D, Chiavarina B, Zhou J, Wang C, Pavlides S, Martinez-Cantarin MP, Capozza F, Witkiewicz AK, Flomenberg N, Howell A (2010). Autophagy in cancer associated fibroblasts promotes tumor cell survival: role of hypoxia, HIF1 induction and NFκB activation in the tumor stromal microenvironment. Cell Cycle.

[33] Chiavarina B, Whitaker-Menezes D, Migneco G, Martinez-Outschoorn UE, Pavlides S, Howell A, Tanowitz HB, Casimiro MC, Wang C, Pestell RG, Grieshaber P, Caro J, Sotgia F, Lisanti MP (2010). HIF1-alpha functions as a tumor promoter in cancer associated fibroblasts, and as a tumor suppressor in breast cancer cells: autophagy drives compartment-specific oncogenesis. Cell Cycle.

[34] Mercier I, Casimiro MC, Wang C, Rosenberg AL, Quong J, Minkeu A, Allen KG, Danilo C, Sotgia F, Bonuccelli G, Jasmin JF, Xu H, Bosco E (2008). Human breast cancer-associated fibroblasts (CAFs) show caveolin-1 downregulation and RB tumor suppressor functional inactivation: implications for the response to hormonal therapy. Cancer Biol Ther.

[35] Goetz JG, Minguet S, Navarro-Lérida I, Lazcano JJ, Samaniego R, Calvo E, Tello M, Osteso-Ibáñez T, Pellinen T, Echarri A, Cerezo A, Klein-Szanto AJ, Garcia R (2011). Biomechanical remodeling of the microenvironment by stromal caveolin-1 favors tumor invasion and metastasis. Cell.

[36] Camp RL, Dolled-Filhart M, Rimm DL (2004). X-tile: a new bio-informatics tool for biomarker assessment and outcome-based cut-point optimization. Clin Cancer Res.

[37] Martinez-Outschoorn UE, Pavlides S, Whitaker-Menezes D, Daumer KM, Milliman JN, Chiavarina B, Migneco G, Witkiewicz AK, Martinez-Cantarin MP, Flomenberg N, Howell A, Pestell RG, Lisanti MP, Sotgia F (2010). Tumor cells induce the cancer associated fibroblast phenotype via caveolin-1 degradation: implications for breast cancer and DCIS therapy with autophagy inhibitors. Cell Cycle.

[38] Capparelli C, Guido C, Whitaker-Menezes D, Bonuccelli G, Balliet R, Pestell TG, Goldberg AF, Pestell RG, Howell A, Sneddon S, Birbe R, Tsirigos A, Martinez-Outschoorn U (2012). Autophagy and senescence in cancer-associated fibroblasts metabolically supports tumor growth and metastasis via glycolysis and ketone production. Cell Cycle.

[39] Martinez-Outschoorn UE, Peiris-Pagés M, Pestell RG, Sotgia F, Lisanti MP (2017). Cancer metabolism: a therapeutic perspective. Nat Rev Clin Oncol.

[40] Liang XH, Jackson S, Seaman M, Brown K, Kempkes B, Hibshoosh H, Levine B (1999). Induction of autophagy and inhibition of tumorigenesis by beclin 1. Nature.

[41] Cuervo AM, Wong E (2014). Chaperone-mediated autophagy: roles in disease and aging. Cell Res.

[42] Luo Y, Zhou H, Krueger J, Kaplan C, Lee SH, Dolman C, Markowitz D, Wu W, Liu C, Reisfeld RA, Xiang R (2006). Targeting tumor-associated macrophages as a novel strategy against breast cancer. J Clin Invest.

[43] Condeelis J, Pollard JW (2006). Macrophages: obligate partners for tumor cell migration, invasion, and metastasis. Cell.

[44] Tang X (2013). Tumor-associated macrophages as potential diagnostic and prognostic biomarkers in breast cancer. Cancer Lett.

[45] Kitamura T, Pollard JW (2015). Therapeutic potential of chemokine signal inhibition for metastatic breast cancer. Pharmacol Res.

[46] Williams CB, Yeh ES, Soloff AC (2016). Tumor-associated macrophages: unwitting accomplices in breast cancer malignancy. NPJ Breast Cancer.

[47] Sidney LE, Branch MJ, Dunphy SE, Dua HS, Hopkinson A (2014). Concise review: evidence for CD34 as a common marker for diverse progenitors. Stem Cells.

[48] Rudland PS, Platt-Higgins A, El-Tanani M, De Silva Rudland S, Barraclough R, Winstanley JH, Howitt R, West CR (2002). Prognostic significance of the metastasis-associated protein osteopontin in human breast cancer. Cancer Res.

[49] Esposito M, Kang Y (2014). Targeting tumor-stromal interactions in bone metastasis. Pharmacol Ther.

[50] Shevde LA, Das S, Clark DW, Samant RS (2010). Osteopontin: an effector and an effect of tumor metastasis. Curr Mol Med.

[51] Shao Z, Morser J, Leung LL (2014). Thrombin cleavage of osteopontin disrupts a pro-chemotactic sequence for dendritic cells, which is compensated by the release of its pro-chemotactic C-terminal fragment. J Biol Chem.

[52] Weber GF, Zawaideh S, Hikita S, Kumar VA, Cantor H, Ashkar S (2002). Phosphorylation-dependent interaction of osteopontin with its receptors regulates macrophage migration and activation. J Leukoc Biol.

[53] Katagiri YU, Sleeman J, Fujii H, Herrlich P, Hotta H, Tanaka K, Chikuma S, Yagita H, Okumura K, Murakami M, Saiki I, Chambers AF, Uede T (1999). CD44 variants but not CD44s cooperate with beta1-containing integrins to permit cells to bind to osteopontin independently of arginine-glycine-aspartic acid, thereby stimulating cell motility and chemotaxis. Cancer Res.

[54] Agnihotri R, Crawford HC, Haro H, Matrisian LM, Havrda MC, Liaw L (2001). Osteopontin, a novel substrate for matrix metalloproteinase-3 (stromelysin-1) and matrix metalloproteinase-7 (matrilysin). J Biol Chem.

[55] Casimiro MC, Di Sante G, Crosariol M, Loro E, Dampier W, Ertel A, Yu Z, Saria EA, Papanikolaou A, Li Z, Wang C, Addya S, Lisanti MP (2015). Kinase-independent role of cyclin D1 in chromosomal instability and mammary tumorigenesis. Oncotarget.

[56] Arnold A, Papanikolaou A (2005). Cyclin D1 in breast cancer pathogenesis. J Clin Oncol.

[57] Peurala E, Koivunen P, Haapasaari KM, Bloigu R, Jukkola-Vuorinen A (2013). The prognostic significance and value of cyclin D1, CDK4 and p16 in human breast cancer. Breast Cancer Res.

[58] Esteva FJ, Hortobagyi GN (2004). Prognostic molecular markers in early breast cancer. Breast Cancer Res.

[59] Leontieva OV, Demidenko ZN, Blagosklonny MV (2013). MEK drives cyclin D1 hyperelevation during geroconversion. Cell Death Differ.

[60] Mercier I, Camacho J, Titchen K, Gonzales DM, Quann K, Bryant KG, Molchansky A, Milliman JN, Whitaker-Menezes D, Sotgia F, Jasmin JF, Schwarting R, Pestell RG (2012). Caveolin-1 and accelerated host aging in the breast tumor microenvironment: chemoprevention with rapamycin, an mTOR inhibitor and anti-aging drug. Am J Pathol.

[61] Casimiro MC, Di Sante G, Di Rocco A, Loro E, Pupo C, Pestell TG, Bisetto S, Velasco-Velázquez MA, Jiao X, Li Z, Kusminski CM, Seifert EL, Wang C (2017). Cyclin D1 restrains oncogene-induced autophagy by regulating the AMPK-LKB1 signaling axis. Cancer Res.

[62] Li Z, Wang C, Jiao X, Lu Y, Fu M, Quong AA, Dye C, Yang J, Dai M, Ju X, Zhang X, Li A, Burbelo P (2006). Cyclin D1 regulates cellular migration through the inhibition of thrombospondin 1 and ROCK signaling. Mol Cell Biol.

[63] Gabrilovich DI, Nagaraj S (2009). Myeloid-derived suppressor cells as regulators of the immune system. Nat Rev Immunol.

[64] Lechner MG, Liebertz DJ, Epstein AL (2010). Characterization of cytokine-induced myeloid-derived suppressor cells from normal human peripheral blood mononuclear cells. J Immunol.

[65] Gabrilovich DI, Ostrand-Rosenberg S, Bronte V (2012). Coordinated regulation of myeloid cells by tumours. Nat Rev Immunol.

[66] Joyce JA, Pollard JW (2009). Microenvironmental regulation of metastasis. Nat Rev Cancer.

[67] Gauthier N, Lohm S, Touzery C, Chantôme A, Perette B, Reveneau S, Brunotte F, Juillerat-Jeanneret L, Jeannin JF (2004). Tumour-derived and host-derived nitric oxide differentially regulate breast carcinoma metastasis to the lungs. Carcinogenesis.

[68] Kojima Y, Acar A, Eaton EN, Mellody KT, Scheel C, Ben-Porath I, Onder TT, Wang ZC, Richardson AL, Weinberg RA, Orimo A (2010). Autocrine TGF-beta and stromal cell-derived factor-1 (SDF-1) signaling drives the evolution of tumor-promoting mammary stromal myofibroblasts. Proc Natl Acad Sci USA.

[69] Kitamura T, Qian BZ, Soong D, Cassetta L, Noy R, Sugano G, Kato Y, Li J, Pollard JW (2015). CCL2-induced chemokine cascade promotes breast cancer metastasis by enhancing retention of metastasis-associated macrophages. J Exp Med.

[70] Fu M, Rao M, Bouras T, Wang C, Wu K, Zhang X, Li Z, Yao TP, Pestell RG (2005). Cyclin D1 inhibits peroxisome proliferator-activated receptor gamma-mediated adipogenesis through histone deacetylase recruitment. J Biol Chem.

[71] Ju X, Casimiro MC, Gormley M, Meng H, Jiao X, Katiyar S, Crosariol M, Chen K, Wang M, Quong AA, Lisanti MP, Ertel A, Pestell RG (2014). Identification of a cyclin D1 network in prostate cancer that antagonizes epithelial-mesenchymal restraint. Cancer Res.

[72] Bienvenu F, Jirawatnotai S, Elias JE, Meyer CA, Mizeracka K, Marson A, Frampton GM, Cole MF, Odom DT, Odajima J, Geng Y, Zagozdzon A, Jecrois M (2010). Transcriptional role of cyclin D1 in development revealed by a genetic-proteomic screen. Nature.

[73] Reutens AT, Fu M, Wang C, Albanese C, McPhaul MJ, Sun Z, Balk SP, Jänne OA, Palvimo JJ, Pestell RG (2001). Cyclin D1 binds the androgen receptor and regulates hormone-dependent signaling in a p300/CBP-associated factor (P/CAF)-dependent manner. Mol Endocrinol.

[74] Sodir NM, Swigart LB, Karnezis AN, Hanahan D, Evan GI, Soucek L (2011). Endogenous Myc maintains the tumor microenvironment. Genes Dev.

[75] Toullec A, Gerald D, Despouy G, Bourachot B, Cardon M, Lefort S, Richardson M, Rigaill G, Parrini MC, Lucchesi C, Bellanger D, Stern MH, Dubois T (2010). Oxidative stress promotes myofibroblast differentiation and tumour spreading. EMBO Mol Med.

[76] Suzuki A, Kusakai G, Shimojo Y, Chen J, Ogura T, Kobayashi M, Esumi H (2005). Involvement of transforming growth factor-beta 1 signaling in hypoxia-induced tolerance to glucose starvation. J Biol Chem.

[77] Hulit J, Bash T, Fu M, Galbiati F, Albanese C, Sage DR, Schlegel A, Zhurinsky J, Shtutman M, Ben-Ze’ev A, Lisanti MP, Pestell RG (2000). The cyclin D1 gene is transcriptionally repressed by caveolin-1. J Biol Chem.

[78] Shtutman M, Zhurinsky J, Simcha I, Albanese C, D’Amico M, Pestell R, Ben-Ze’ev A (1999). The cyclin D1 gene is a target of the beta-catenin/LEF-1 pathway. Proc Natl Acad Sci USA.

[79] Albanese C, D’Amico M, Reutens AT, Fu M, Watanabe G, Lee RJ, Kitsis RN, Henglein B, Avantaggiati M, Somasundaram K, Thimmapaya B, Pestell RG (1999). Activation of the cyclin D1 gene by the E1A-associated protein p300 through AP-1 inhibits cellular apoptosis. J Biol Chem.

[80] Rubin R, Arzumanyan A, Soliera AR, Ross B, Peruzzi F, Prisco M (2007). Insulin receptor substrate (IRS)-1 regulates murine embryonic stem (mES) cells self-renewal. J Cell Physiol.

[81] Liu M, Sakamaki T, Casimiro MC, Willmarth NE, Quong AA, Ju X, Ojeifo J, Jiao X, Yeow WS, Katiyar S, Shirley LA, Joyce D, Lisanti MP (2010). The canonical NF-kappaB pathway governs mammary tumorigenesis in transgenic mice and tumor stem cell expansion. Cancer Res.

[82] Jain P, Coisne C, Enzmann G, Rottapel R, Engelhardt B (2010). Alpha4beta1 integrin mediates the recruitment of immature dendritic cells across the blood-brain barrier during experimental autoimmune encephalomyelitis. J Immunol.

[83] Rahman S, Khan ZK, Wigdahl B, Jennings SR, Tangy F, Jain P (2011). Murine FLT3 ligand-derived dendritic cell-mediated early immune responses are critical to controlling cell-free human T cell leukemia virus type 1 infection. J Immunol.

[84] Dehlin M, Bokarewa M, Rottapel R, Foster SJ, Magnusson M, Dahlberg LE, Tarkowski A (2008). Intra-articular fms-like tyrosine kinase 3 ligand expression is a driving force in induction and progression of arthritis. PLoS One.

[85] Chen K, Wu K, Cai S, Zhang W, Zhou J, Wang J, Ertel A, Li Z, Rui H, Quong A, Lisanti MP, Tozeren A, Tanes C (2013). Dachshund binds p53 to block the growth of lung adenocarcinoma cells. Cancer Res.

[86] Peck AR, Girondo MA, Liu C, Kovatich AJ, Hooke JA, Shriver CD, Hu H, Mitchell EP, Freydin B, Hyslop T, Chervoneva I, Rui H (2016). Validation of tumor protein marker quantification by two independent automated immunofluorescence image analysis platforms. Mod Pathol.

[87] Peck AR, Witkiewicz AK, Liu C, Stringer GA, Klimowicz AC, Pequignot E, Freydin B, Tran TH, Yang N, Rosenberg AL, Hooke JA, Kovatich AJ, Nevalainen MT (2011). Loss of nuclear localized and tyrosine phosphorylated Stat5 in breast cancer predicts poor clinical outcome and increased risk of antiestrogen therapy failure. J Clin Oncol.

[88] Beer LA, Tang HY, Sriswasdi S, Barnhart KT, Speicher DW (2011). Systematic discovery of ectopic pregnancy serum biomarkers using 3-D protein profiling coupled with label-free quantitation. J Proteome Res.

[89] Goldman AR, Bitler BG, Schug Z, Conejo-Garcia JR, Zhang R, Speicher DW (2016). The Primary Effect on the Proteome of ARID1A-mutated Ovarian Clear Cell Carcinoma is Downregulation of the Mevalonate Pathway at the Post-transcriptional Level. Mol Cell Proteomics.

[90] Cox J, Mann M (2008). MaxQuant enables high peptide identification rates, individualized p.p.b.-range mass accuracies and proteome-wide protein quantification. Nat Biotechnol.

[91] Tyanova S, Temu T, Sinitcyn P, Carlson A, Hein MY, Geiger T, Mann M, Cox J (2016). The Perseus computational platform for comprehensive analysis of (prote)omics data. Nat Methods.

