# Supplementary material for: Stromal cyclin D1 promotes heterotypic immune signaling and breast cancer growth
Source: Oncotarget. 2017 Aug 4;8(47):81754–75. doi: 10.18632/oncotarget.19953 (PMC5669846; doi:10.18632/oncotarget.19953)
Supplement: Supplementary file 1 [file oncotarget-08-81754-s001.pdf]

Stromal cyclin D1 promotes heterotypic immune signaling and breast cancer growth

SUPPLEMENTARY MATERIALS

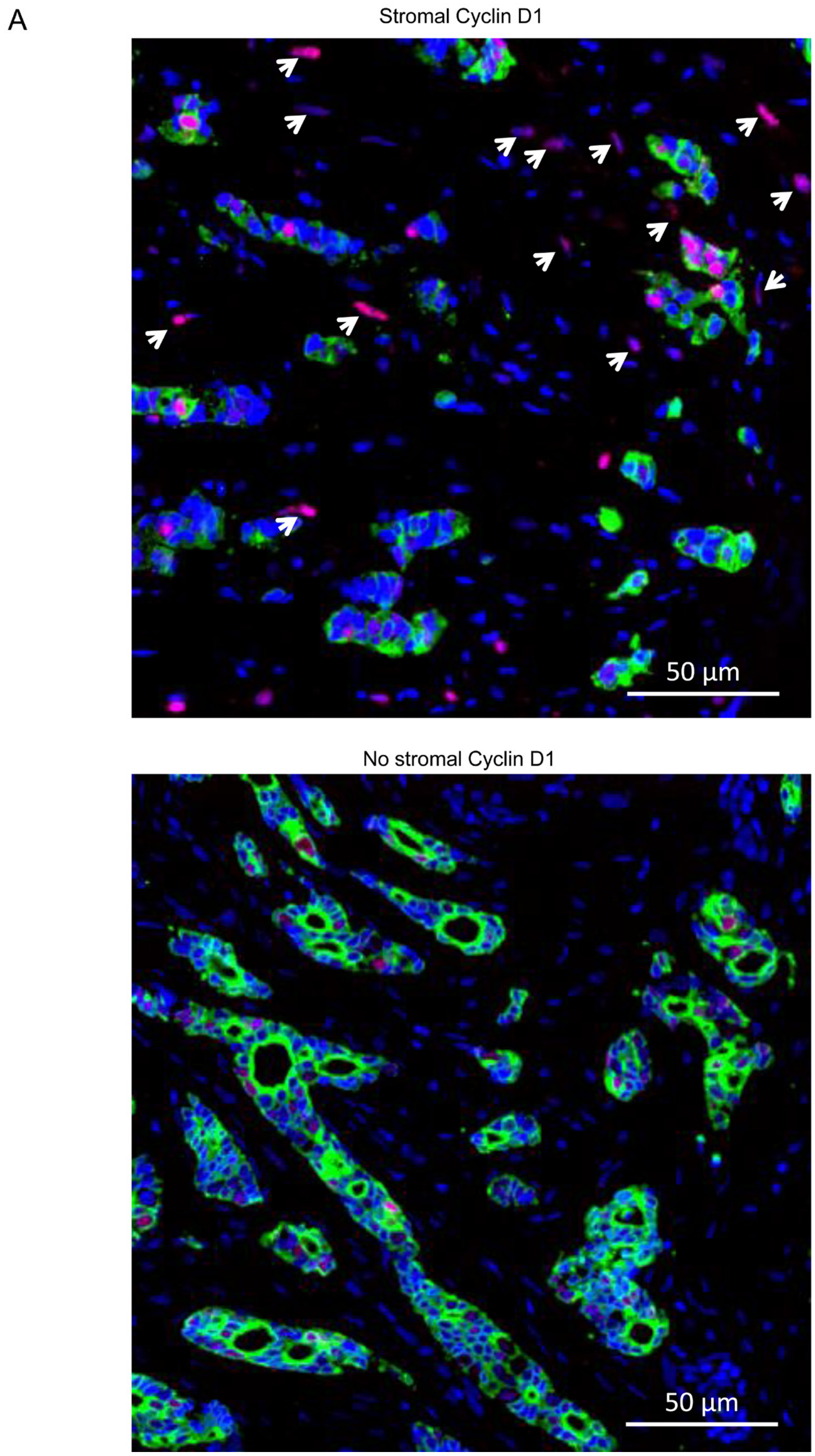

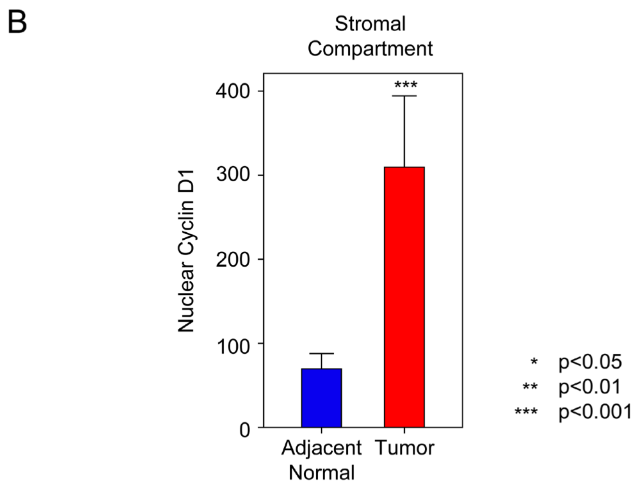

Supplementary Figure 1: Stromal cyclin D1 in human breast cancers correlates with outcome. **A.** Representative examples of immunohistochemistry for nuclear stromal cyclin D1 conducted on human breast cancers showing either high or low cyclin D1 staining. Cyclin D1 in stromal cells was indicated by arrow. **B.** Quantitation of nuclear cyclin D1 in the stroma of breast tumors or adjacent normal tissue.

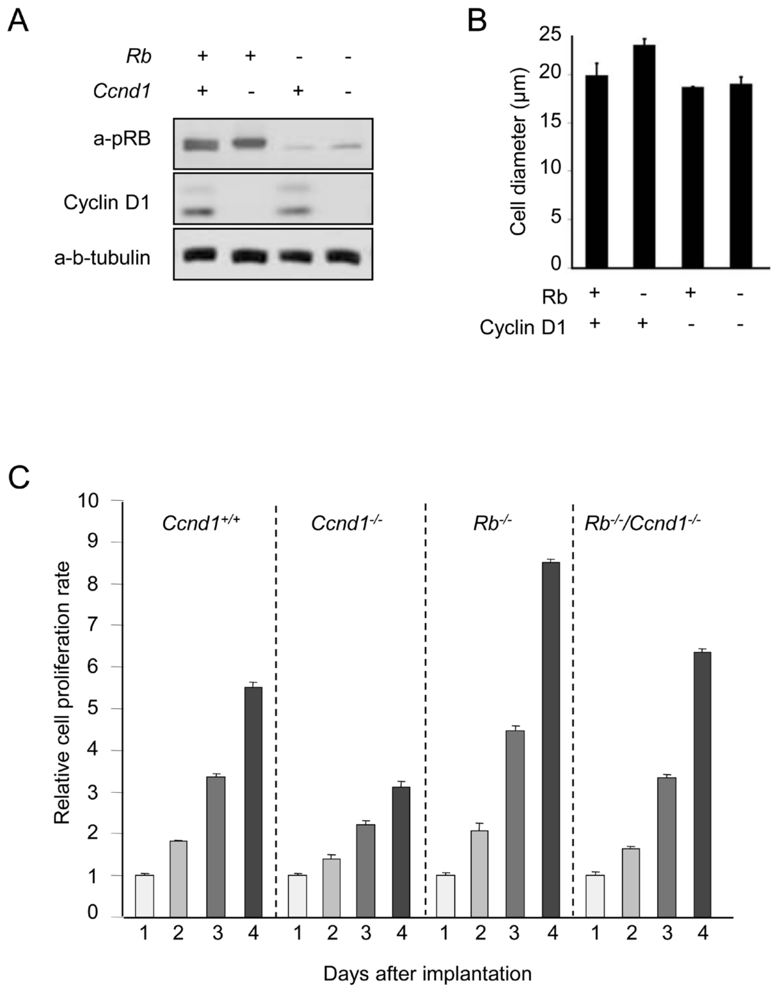

Supplementary Figure 2: Analysis of *cyclin D1*, *pRb* and *cyclin D1/pRb* deletion mouse embryonic fibroblasts. A. Western blot of MEFs derived from gene deletion mice with **B.** cell diameter and **C.** cell proliferation shown as mean $\pm$ SEM for N= 3 separate experiments.

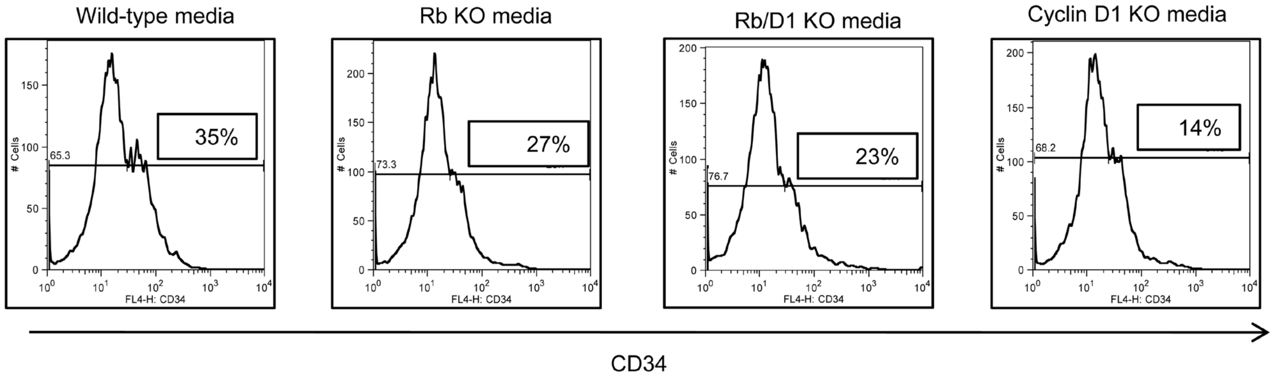

Supplementary Figure 3: Cyclin D1 conditioned medium induces expansion of CD34 positive hematopoietic stem cells (HSCs). Murine bone marrow cells were cultured with the supernatant from MEFs (wild type, *pRB*$^{-/-}$, *pRB*$^{-/-}$*/cyclin D1*$^{-/-}$, *cyclin D1*$^{-/-}$) with Flt3L media for 9 days and analyzed for CD34 expression by flow cytometry (Materials and Methods). Histogram represents the percentage of live CD34^{+} cells. Gate was set based on unstained and/or isotype control for each sample.

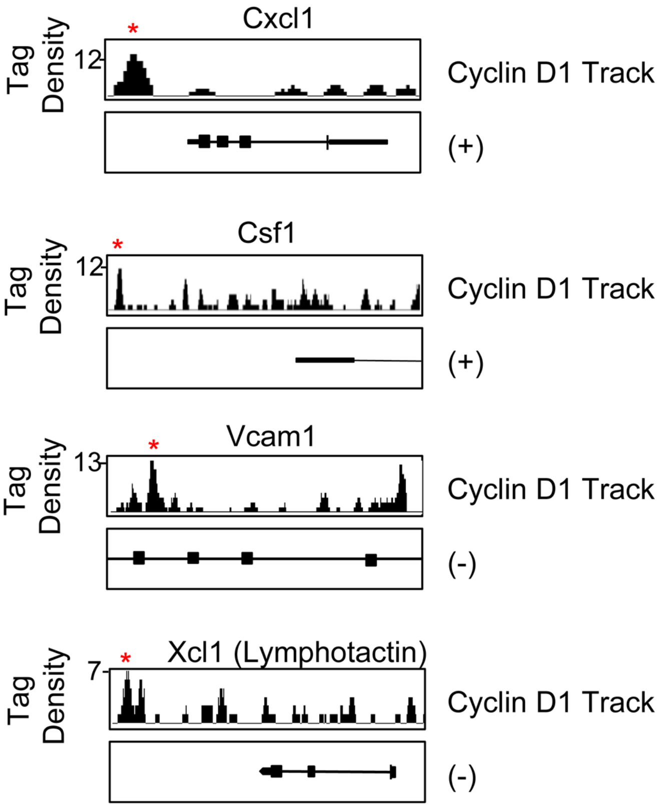

Supplementary Figure 4: Cyclin D1 is associated with inflammatory target gene in the context of chromatin. ChIP-Seq analysis of cyclin D1 binding to *Csf, Ccl2, Cxcl2, Cxcl2* genes.

For Supplementary Tables 1 see in Supplementary Files
