# Supplementary material for: Stromal cyclin D1 promotes heterotypic immune signaling and breast cancer growth
Source: Oncotarget. 2017 Aug 4;8(47):81754–75. doi: 10.18632/oncotarget.19953 (PMC5669846; doi:10.18632/oncotarget.19953)
Supplement: Supplementary file 2 [file oncotarget-08-81754-s002.docx]

**Supplementary Table 1. A global secretome analysis of conditioned media from cyclin D1^Stroma^ and control^Stroma^ hTERT cell lines by analyzing biological triplicates for each condition**

| **Protein IDs** | **Protein names** | **Gene names** | **Razor + unique peptides** | **Unique peptides** | **Sequence coverage [%]** | **Mol. weight [kDa]** | **CyclinD1-1 log2 LFQ Intensity** | **CyclinD1-2 log2 LFQ Intensity** | **CyclinD1-3 log2 LFQ Intensity** | **GFP-1 log2 LFQ Intensity** | **GFP-2 log2 LFQ Intensity** | **GFP-3 log2 LFQ Intensity** | **CyclinD1 vs. GFP LFQ log2 ratio** | **CyclinD1 vs. GFP LFQ fold-change** | **Student's t-test p-value (based on log2 LFQ intensities)** |
| --- | --- | --- | --- | --- | --- | --- | --- | --- | --- | --- | --- | --- | --- | --- | --- |
| B7Z719;B4DVY9;P0DJD9;P0DJD8;P0DJD7;B7ZW16;F5H0H6;F5GWT0;Q658W9;F5GXL4;C9JM59;B7ZW10;B7ZW66;O95577;O95576;B4DVY3 | Pepsin A-5;Pepsin A-3;Pepsin A-4 | PGA5;PGA3;PGA4;DKFZp666J2410 | 3 | 3 | 12.7 | 23.425 | 22.87 | 25.24 | 25.59 | 19.28 | 19.75 | 19.94 | 4.91 | 29.96 | 0.00498 |
| Q8WYJ5;Q9BX68;Q53HJ8 | Histidine triad nucleotide-binding protein 2, mitochondrial | HINT2 | 3 | 3 | 39.8 | 13.918 | 21.74 | 21.77 | 21.19 | 18.43 | 19.46 | 18.02 | 2.93 | 7.61 | 0.00338 |
| B5MCZ3;H9C875;P05231;B5MC21;B5MC14;B4DVM1;B4DNQ5;B4DNV3;C9J5B0 | Interleukin-6 | IL6 | 3 | 3 | 15.9 | 21.494 | 21.78 | 21.52 | 21.88 | 18.31 | 19.61 | 18.82 | 2.81 | 7.03 | 0.00207 |
| Q32MZ4-3;Q32MZ4-2;Q32MZ4;Q05D04 | Leucine-rich repeat flightless-interacting protein 1 | LRRFIP1 | 3 | 3 | 5.9 | 82.688 | 22.12 | 22.46 | 22.62 | 20.65 | 19.84 | 18.37 | 2.78 | 6.87 | 0.01530 |
| B4DLP6;Q9BPW0;H0YDU8;Q53FR0;P53041;B2R6R6;B4DDZ8;A8MU39;Q59F46;B7Z1I1 | Serine/threonine-protein phosphatase;Serine/threonine-protein phosphatase 5 | PPP5C | 6 | 6 | 15.6 | 54.885 | 22.93 | 23.13 | 23.82 | 19.63 | 20.38 | 21.74 | 2.71 | 6.53 | 0.01594 |
| P39748-2;B4DWZ4;P39748;I3L3E9;F5H1Y3 | Flap endonuclease 1 | FEN1 | 2 | 2 | 10.1 | 35.673 | 20.85 | 21.09 | 20.95 | 18.44 | 19.46 | 17.42 | 2.52 | 5.75 | 0.01313 |
| A8KA83;Q9P0L0;Q9P0L0-2;J3QKM9;E5RK64;Q59EZ6;O95292-2;O95292 | Vesicle-associated membrane protein-associated protein A | VAPA | 3 | 3 | 11.6 | 27.317 | 23.07 | 23.33 | 23.53 | 20.80 | 20.75 | 20.82 | 2.52 | 5.73 | 0.00005 |
| Q05D33;Q6PJL0;B4E0E1;P09874;B2R5W3;Q5VX84;Q5VX85;Q96P95 | Poly [ADP-ribose] polymerase 1 | PARP1 | 5 | 5 | 28 | 26.13 | 22.87 | 23.68 | 22.97 | 20.39 | 21.28 | 20.31 | 2.51 | 5.71 | 0.00331 |
| Q14008-2;Q14008;Q14008-3;Q8NAH3;E9PQH5 | Cytoskeleton-associated protein 5 | CKAP5 | 10 | 10 | 5.7 | 218.52 | 23.42 | 23.12 | 23.23 | 19.25 | 21.66 | 21.54 | 2.44 | 5.42 | 0.03640 |
| A5PLK7;Q9P258 | Protein RCC2 | RCC2 | 14 | 14 | 39.4 | 49.678 | 25.60 | 25.12 | 25.43 | 22.87 | 22.96 | 23.07 | 2.42 | 5.34 | 0.00009 |
| P48681;D3DVC4;Q9H6U9;Q2YDX4 | Nestin | NES | 3 | 3 | 3.3 | 177.44 | 22.08 | 22.50 | 22.08 | 20.64 | 19.08 | 19.70 | 2.42 | 5.34 | 0.00708 |
| Q03252;J9JID7 | Lamin-B2 | LMNB2 | 4 | 4 | 8.3 | 67.688 | 23.97 | 24.06 | 23.46 | 19.88 | 21.90 | 22.50 | 2.40 | 5.29 | 0.04200 |
| Q9NZL9;Q9NZL9-4;Q9NZL9-2;A8K7A4;Q9NZL9-3;E5RJR3;Q9NZL9-5;H7C0X7 | Methionine adenosyltransferase 2 subunit beta | MAT2B | 7 | 4 | 28.1 | 37.551 | 23.95 | 23.76 | 24.51 | 20.55 | 22.18 | 22.64 | 2.28 | 4.86 | 0.02753 |
| Q9Y3C8 | Ubiquitin-fold modifier-conjugating enzyme 1 | UFC1 | 3 | 3 | 21.6 | 19.458 | 21.44 | 21.79 | 22.74 | 20.26 | 19.08 | 19.80 | 2.28 | 4.84 | 0.01194 |
| P52926-3;P52926-6;P52926-4;F5H2U8;P52926-5;P52926-2;P52926;F5H2A4;F6LHU9;Q1M183;F5H6H0;D2WHG0;A5Y0M9 | High mobility group protein HMGI-C | HMGA2 | 2 | 2 | 41.9 | 9.2733 | 23.95 | 24.32 | 24.49 | 21.54 | 22.43 | 22.05 | 2.25 | 4.75 | 0.00178 |
| P63010-3;H0UID5;H0UID4;P63010;H0UID3;Q68DI0;P63010-2;A8K916;A8K0G3;K7EJT8;B4DWG4;B4E261;Q96EL6;C9J1E7;Q10567-4;B4DIL5;K7EN71;K7ERB2;K7EJ01 | AP-2 complex subunit beta | AP2B1;DKFZp781K0743 | 10 | 6 | 16.7 | 98.117 | 24.03 | 23.79 | 23.39 | 19.94 | 22.31 | 22.25 | 2.24 | 4.73 | 0.04870 |
| P05161 | Ubiquitin-like protein ISG15 | ISG15 | 5 | 5 | 35.2 | 17.887 | 26.85 | 26.29 | 26.46 | 24.18 | 24.52 | 24.32 | 2.19 | 4.57 | 0.00034 |
| P49321-2;P49321;P49321-3;E9PRH9;H0YF33;Q9P1N1;Q5T624;B4DS57;P49321-4;Q53H03;H0YDS9;O95138;E9PI86;E9PPQ8;Q6PJY7 | Nuclear autoantigenic sperm protein | NASP | 6 | 6 | 16.7 | 48.804 | 25.01 | 24.85 | 24.55 | 22.94 | 22.59 | 22.44 | 2.15 | 4.43 | 0.00042 |
| Q6BCY4;Q6BCY4-2;A8K237;E9PIV9;E9PMI2;E9PQW2;E9PRM4;Q59GV9 | NADH-cytochrome b5 reductase 2 | CYB5R2 | 8 | 8 | 48.2 | 31.458 | 25.35 | 24.70 | 24.08 | 22.62 | 21.92 | 23.19 | 2.13 | 4.39 | 0.01476 |
| A2IRN0 |  |  | 2 | 2 | 10 | 85.015 | 27.02 | 26.53 | 26.27 | 24.34 | 24.73 | 24.36 | 2.13 | 4.38 | 0.00109 |
| Q01970-2;Q01970 | 1-phosphatidylinositol 4,5-bisphosphate phosphodiesterase beta-3 | PLCB3 | 3 | 3 | 3.5 | 131.2 | 21.41 | 21.50 | 21.18 | 19.08 | 19.09 | 19.61 | 2.10 | 4.29 | 0.00046 |
| Q8WXX5 | DnaJ homolog subfamily C member 9 | DNAJC9 | 3 | 3 | 9.6 | 29.909 | 21.24 | 21.05 | 20.99 | 19.25 | 17.92 | 19.82 | 2.09 | 4.27 | 0.02094 |
| Q9Y333 | U6 snRNA-associated Sm-like protein LSm2 | LSM2 | 2 | 2 | 40 | 10.834 | 21.24 | 21.44 | 21.39 | 18.84 | 19.46 | 19.56 | 2.07 | 4.20 | 0.00088 |
| Q02790;B2R9U2;F5H1U3;H0YFG2 | Peptidyl-prolyl cis-trans isomerase FKBP4;Peptidyl-prolyl cis-trans isomerase FKBP4, N-terminally processed | FKBP4 | 9 | 9 | 23.7 | 51.804 | 24.25 | 23.96 | 23.94 | 21.94 | 22.44 | 21.61 | 2.06 | 4.16 | 0.00143 |
| V9HW39;Q9NR45;Q5TBR0;Q5TBR1 | Sialic acid synthase | NANS | 2 | 2 | 7.8 | 40.321 | 21.70 | 21.81 | 21.58 | 18.89 | 20.93 | 19.12 | 2.05 | 4.13 | 0.03393 |
| Q16719;A8K7T0;A8K4D5;Q53F63;Q53SY0;Q53SX6;Q16719-2 | Kynureninase | KYNU | 3 | 3 | 11.2 | 52.351 | 20.66 | 21.63 | 22.07 | 19.00 | 19.64 | 19.65 | 2.03 | 4.08 | 0.01231 |
| E9PQC9;H0YE58;H0YE72;E9PQZ1;P29692-3;E9PK01;P29692-4;P29692;Q9BW34;Q71RH4;Q9H7G6;P29692-2;D3DWK1;B2RAR6;E9PRY8 | Elongation factor 1-delta | EEF1D | 2 | 2 | 61.1 | 3.9417 | 24.47 | 23.82 | 24.77 | 22.43 | 22.45 | 22.10 | 2.02 | 4.06 | 0.00261 |
| Q6FI03;Q32P45;Q13283;E5RIZ6;F5H4D6;B7Z8K4;Q6ZP53;Q53HH4 | Ras GTPase-activating protein-binding protein 1 | G3BP;G3BP1 | 2 | 2 | 4.3 | 52.164 | 22.69 | 22.33 | 22.87 | 20.81 | 20.82 | 20.24 | 2.01 | 4.02 | 0.00131 |
| F5GYN4;Q96FW1;J3KR44;B4DPD5;Q659F9;F5H6Q1;F5GYJ8;F5H3F0;B4E053;Q96FW1-2;B3KUV5 | Ubiquitin thioesterase OTUB1 | OTUB1;DKFZp564E242 | 8 | 8 | 40.7 | 28.05 | 24.83 | 25.22 | 25.18 | 22.64 | 22.91 | 23.79 | 1.96 | 3.89 | 0.00594 |
| P84090;G3V279 | Enhancer of rudimentary homolog | ERH | 3 | 3 | 26 | 12.259 | 24.01 | 23.93 | 24.51 | 22.02 | 22.57 | 22.03 | 1.94 | 3.85 | 0.00161 |
| P42224-2;J3KPM9;P42224 | Signal transducer and activator of transcription 1-alpha/beta | STAT1 | 4 | 4 | 6.6 | 83.042 | 22.68 | 22.22 | 21.94 | 20.13 | 20.51 | 20.67 | 1.85 | 3.60 | 0.00235 |
| C9JQD4;O43447;A6NNE7;Q6FH57;H0YEL5;A6NM32;C9JAN8 | Peptidyl-prolyl cis-trans isomerase;Peptidyl-prolyl cis-trans isomerase H | PPIH | 5 | 5 | 34.7 | 15.8 | 23.91 | 23.89 | 24.94 | 22.73 | 22.62 | 22.04 | 1.78 | 3.44 | 0.01177 |
| F5H8J4;B4DZE1;F5H564;B4DH17;O43432-4;O43432;O43432-3;Q59GJ0 | Eukaryotic translation initiation factor 4 gamma 3 | EIF4G3 | 4 | 4 | 4.7 | 121.59 | 22.61 | 23.00 | 23.33 | 21.03 | 21.46 | 21.24 | 1.74 | 3.33 | 0.00199 |
| P09651-3;F8W6I7;Q6IPF2;P09651-2;A0A024RC46;P09651;F8VTQ5;F8VZ49;Q0VAC0;Q9BSM5;Q32P51;F8W646;H0YH80;B4DP35;B4E0B5;F8VYN5;Q3MI39 | Heterogeneous nuclear ribonucleoprotein A1;Heterogeneous nuclear ribonucleoprotein A1-like 2 | HNRNPA1;HNRPA1;HNRNPA1L2 | 6 | 6 | 27.3 | 29.386 | 23.76 | 23.58 | 23.66 | 21.25 | 22.10 | 22.44 | 1.74 | 3.33 | 0.00839 |
| Q92688-2;Q53F35;Q92688;Q5T6W8 | Acidic leucine-rich nuclear phosphoprotein 32 family member B | ANP32B | 9 | 9 | 37.4 | 22.276 | 27.26 | 27.37 | 27.58 | 25.71 | 25.81 | 25.51 | 1.73 | 3.31 | 0.00018 |
| O43847;O43847-2;B1AKJ5;Q96L67;B4DRI0;Q6UUU9;G3V1R5;F5H7V1;B4DYV0;H0Y5G9 | Nardilysin | NRD1 | 17 | 17 | 15.6 | 131.57 | 24.48 | 24.61 | 25.10 | 22.83 | 23.32 | 22.87 | 1.72 | 3.30 | 0.00220 |
| Q53QE9;Q16851-2;Q16851;B2RAN1;E7EUC7;B4DUP2;C9JTZ5;C9JUW1;C9JWG0;C9JVG3;C9JQU9;C9JNZ1;D6W5E6 | UTP--glucose-1-phosphate uridylyltransferase | UGP2 | 2 | 2 | 5.9 | 49.221 | 22.00 | 21.93 | 22.27 | 20.50 | 20.25 | 20.29 | 1.72 | 3.30 | 0.00018 |
| Q96AG4 | Leucine-rich repeat-containing protein 59 | LRRC59 | 2 | 2 | 8.1 | 34.93 | 21.57 | 21.78 | 22.29 | 20.06 | 20.08 | 20.35 | 1.72 | 3.29 | 0.00183 |
| P46821;A0A024RAM4;Q05BW8;D6RA32;Q86X89;Q32NB7;Q6PJD3;A5D6X1;Q4VBY4;A2BDK6;D6RA40;A5D8X6;D6RCL2;D6RGJ3;Q5H9P1 | Microtubule-associated protein 1B;MAP1B heavy chain;MAP1 light chain LC1 | MAP1B | 4 | 4 | 2.3 | 270.63 | 22.42 | 22.30 | 21.62 | 19.93 | 20.85 | 20.54 | 1.67 | 3.19 | 0.01058 |
| H0YN26;Q6PKH8;Q08AJ6;P39687;Q3KPI8;Q1AHP8;H7BZ09;O95626;O43423 | Acidic leucine-rich nuclear phosphoprotein 32 family member A | ANP32A | 12 | 11 | 68.4 | 19.997 | 26.96 | 26.71 | 27.19 | 25.13 | 25.52 | 25.28 | 1.65 | 3.13 | 0.00076 |
| Q9Y376;Q7Z4X0;B7ZBJ4;Q5TAW7;Q9H9S4;B7ZBJ5 | Calcium-binding protein 39 | CAB39 | 17 | 17 | 41.9 | 39.869 | 26.27 | 26.03 | 25.97 | 24.47 | 24.63 | 24.26 | 1.64 | 3.11 | 0.00032 |
| Q95IE7;A0FIZ4;D0VE44;K7P5A8;K7DWB0;F4YU38;B2YGA1;I6NVP0;F2X5V5;C1KJL2;C9E1F3;C1KJK9 | | HLA-B;HLA-C | 6 | 0 | 34.2 | 34.627 | 24.73 | 24.84 | 24.76 | 22.94 | 23.72 | 22.82 | 1.62 | 3.07 | 0.00458 |
| H3BTL1;H6UMI0;Q9GZQ8;A6NCE7;Q9H492;Q9H492-2 | Microtubule-associated proteins 1A/1B light chain 3B;Microtubule-associated proteins 1A/1B light chain 3 beta 2;Microtubule-associated proteins 1A/1B light chain 3A | MAP1LC3B;MAP1LC3B2;MAP1LC3A | 2 | 2 | 28 | 9.0115 | 24.04 | 23.71 | 24.32 | 22.61 | 22.59 | 22.09 | 1.60 | 3.02 | 0.00278 |
| Q6FHF5;P12004;Q6FI35;B4DUA2;Q7Z6A3;Q7Z6A2;Q7Z6A1 | Proliferating cell nuclear antigen | PCNA | 9 | 9 | 51.3 | 28.705 | 27.03 | 26.97 | 27.08 | 25.24 | 25.56 | 25.49 | 1.60 | 3.02 | 0.00011 |
| Q09028-3;Q09028;Q09028-2;Q09028-4;H0YEU5;B4DRT0;H0YCT5;H0YF10;H0YDK2;E9PNS6;Q5JP02;E9PNS2;C9JPP3;E9PIC4;C9JAJ9;E9PND5 | Histone-binding protein RBBP4 | RBBP4 | 13 | 9 | 54.9 | 46.158 | 26.38 | 26.54 | 26.82 | 24.69 | 25.16 | 25.11 | 1.59 | 3.02 | 0.00129 |
| B4DR94;Q9UES0;O15498;B5BU81;H7C3K7 | Synaptobrevin homolog YKT6 | YKT6 | 3 | 3 | 18.3 | 18.58 | 22.63 | 22.14 | 22.03 | 20.84 | 20.88 | 20.31 | 1.59 | 3.00 | 0.00362 |
| P17096;P17096-3;H7BYM6;B4DWA0;A0A024RCT9 | High mobility group protein HMG-I/HMG-Y | HMGA1 | 4 | 2 | 41.1 | 11.676 | 27.85 | 27.77 | 28.06 | 26.74 | 25.81 | 26.39 | 1.58 | 2.99 | 0.00515 |
| B0YJC4;P08670;B3KRK8;Q53HU8;Q5JVS8;B0YJC5;B4DUI0;Q9H319;Q45VM8;Q45VM7;Q45VM6;P17661;L7RDA5;A5Z217 | Vimentin | VIM | 11 | 11 | 23.7 | 49.653 | 26.35 | 25.70 | 26.26 | 25.07 | 24.40 | 24.12 | 1.57 | 2.97 | 0.01047 |
| Q9H3P7 | Golgi resident protein GCP60 | ACBD3 | 2 | 2 | 4.2 | 60.593 | 21.69 | 22.10 | 22.09 | 19.64 | 20.60 | 20.96 | 1.56 | 2.96 | 0.01984 |
| P32929-3;P32929;P32929-2 | Cystathionine gamma-lyase | CTH | 5 | 5 | 19.8 | 41.26 | 22.12 | 22.14 | 22.55 | 20.49 | 20.87 | 20.76 | 1.56 | 2.95 | 0.00099 |
| P12081-4;P12081;B4DDD8;B3KWE1;P12081-3;P12081-2;Q52NV4;E7ETE2;B4DEA2;B4E1C5;C9JWK3;C9JV49;P49590-2;P49590;B2R7G6;D6RF05;D6RJE6;B4DQ67;B4DSY5;B4DDN8;C9JHI8;E9PG66 | Histidine--tRNA ligase, cytoplasmic | HARS;HRS | 6 | 6 | 12.1 | 54.846 | 23.67 | 23.20 | 23.81 | 21.96 | 22.05 | 22.01 | 1.55 | 2.93 | 0.00112 |
| Q9BWD1;A8K4W5;Q59GW6;B7Z233 | Acetyl-CoA acetyltransferase, cytosolic | ACAT2 | 2 | 2 | 6.8 | 41.35 | 20.22 | 20.93 | 20.33 | 19.45 | 19.23 | 18.19 | 1.54 | 2.90 | 0.02668 |
| K7EML3;Q9NUL5-3;Q9NUL5-2;Q9NUL5-4;Q9NUL5 | UPF0515 protein C19orf66 | C19orf66 | 2 | 2 | 40.8 | 8.1251 | 20.30 | 21.06 | 21.17 | 18.88 | 19.62 | 19.48 | 1.52 | 2.87 | 0.01300 |
| P40306;Q6IB22;J3QQN1;J3QL48 | Proteasome subunit beta type-10;Proteasome subunit beta type | PSMB10 | 8 | 8 | 47.6 | 28.936 | 26.60 | 26.37 | 27.16 | 24.66 | 25.62 | 25.32 | 1.51 | 2.86 | 0.01452 |
| Q9HB71-3;Q9HB71;Q6NVY0;B4DFD3 | Calcyclin-binding protein | CACYBP | 6 | 6 | 40 | 21.228 | 24.74 | 24.64 | 24.81 | 23.34 | 23.02 | 23.31 | 1.51 | 2.84 | 0.00018 |
| Q96A72;P61326;A6NEC0;F5H6P7;B1ARP8;F5H6N1;F5H3U9;F5H124 | Protein mago nashi homolog 2;Protein mago nashi homolog | MAGOHB;MAGOH | 11 | 11 | 86.5 | 17.276 | 25.36 | 25.47 | 25.27 | 24.00 | 23.61 | 23.99 | 1.50 | 2.82 | 0.00043 |
| P33316-2;H0YNJ9 | Deoxyuridine 5-triphosphate nucleotidohydrolase, mitochondrial | DUT | 9 | 2 | 62.2 | 17.748 | 26.48 | 26.42 | 26.64 | 25.00 | 25.06 | 24.99 | 1.50 | 2.82 | 0.00003 |
| O96019-2;Q6FI97;Q53FS0;O96019;H7C5S0;C9JQT2;D3DNS0;O94805 | Actin-like protein 6A | ACTL6A;BAF53A | 5 | 5 | 17.3 | 43.236 | 22.52 | 22.64 | 23.14 | 22.12 | 20.76 | 20.97 | 1.48 | 2.79 | 0.03272 |
| C9JZA4;C9JJN7;C9JJD0;Q6AWB3;Q6AWB1;Q14203-3;E7EX90;C9J1B7;C9JKG6;E9PCY0;Q14203-4;Q14203-6;Q6MZZ3;Q14203;C9JUI8 | Dynactin subunit 1 | DCTN1;DKFZp686E191;DKFZp686E0752;DKFZp686I0746 | 3 | 3 | 44.7 | 8.1231 | 23.34 | 23.27 | 23.20 | 21.96 | 21.54 | 21.87 | 1.48 | 2.79 | 0.00037 |
| A0A024R2V0;Q86Y04;P27816-4;E9PGM5;P27816-6;P27816;E7EVA0;H0Y2V1;H7C4C5;P27816-5;B9ZVR1;F8W9U4;P27816-2;B5MEG9;Q8NDS5;B4DSQ1;B4DM10 | Microtubule-associated protein;Microtubule-associated protein 4 | MAP4 | 5 | 5 | 11 | 60.598 | 24.98 | 25.31 | 25.46 | 23.76 | 23.79 | 23.76 | 1.48 | 2.79 | 0.00045 |
| Q9UHV9 | Prefoldin subunit 2 | PFDN2 | 2 | 2 | 16.9 | 16.648 | 20.64 | 20.38 | 20.49 | 18.69 | 19.22 | 19.20 | 1.46 | 2.75 | 0.00152 |
| Q6IB91;Q16822;H0YML5;B4DW73;Q16822-2;H0YM31 | Phosphoenolpyruvate carboxykinase [GTP], mitochondrial | PCK2 | 5 | 5 | 9.2 | 70.696 | 22.55 | 22.41 | 21.55 | 20.52 | 20.86 | 20.74 | 1.46 | 2.75 | 0.01115 |
| Q5U071;P26583;D6R9A6 | High mobility group protein B2 | HMGB2 | 5 | 5 | 21.6 | 23.904 | 24.19 | 23.71 | 23.72 | 22.85 | 22.04 | 22.35 | 1.46 | 2.75 | 0.00670 |
| Q9UIQ6-3;Q9UIQ6-2;Q9UIQ6;B2RAK1 | Leucyl-cystinyl aminopeptidase;Leucyl-cystinyl aminopeptidase, pregnancy serum form | LNPEP | 6 | 6 | 6.9 | 115.06 | 22.45 | 22.18 | 22.31 | 19.91 | 21.20 | 21.46 | 1.46 | 2.74 | 0.03938 |
| P23381-2;P23381;H0YJP3;G3V3H8;G3V3Y5 | Tryptophan--tRNA ligase, cytoplasmic;T1-TrpRS;T2-TrpRS | WARS | 5 | 5 | 19.8 | 48.851 | 23.24 | 22.52 | 22.93 | 21.23 | 21.36 | 21.76 | 1.45 | 2.73 | 0.00527 |
| P28062-2;X5D2R7;P28062;Q6FHU0;Q5JNW7;B7Z6U7 | Proteasome subunit beta type-8;Proteasome subunit beta type | PSMB8 | 10 | 10 | 41.5 | 29.769 | 27.78 | 27.87 | 27.98 | 26.31 | 26.48 | 26.50 | 1.44 | 2.72 | 0.00007 |
| Q99598;C4P0D6;C4P0D8;Q7Z3N9;Q5VVQ1;C4P0D4 | Translin-associated protein X | TSNAX;DISC1 | 5 | 5 | 27.9 | 33.112 | 23.40 | 23.50 | 22.21 | 21.55 | 21.94 | 21.31 | 1.44 | 2.71 | 0.03420 |
| O95777;F2Z2Y6;C9JIZ0;C9JNV3 | N-alpha-acetyltransferase 38, NatC auxiliary subunit | NAA38 | 3 | 3 | 36.5 | 10.403 | 22.60 | 22.16 | 22.28 | 20.90 | 20.99 | 20.86 | 1.43 | 2.70 | 0.00048 |
| Q15046;Q15046-2;Q6ZTI3;J3KRL2;H3BPV7;H3BVA8 | Lysine--tRNA ligase | KARS | 8 | 8 | 14.4 | 68.047 | 23.86 | 23.74 | 24.37 | 22.02 | 22.76 | 22.89 | 1.43 | 2.69 | 0.01261 |
| P63165;B8ZZN6;B8ZZJ0;B9A032;G2XKQ0;B8ZZ67;P63165-2 | Small ubiquitin-related modifier 1 | SUMO1 | 3 | 3 | 34.7 | 11.557 | 25.34 | 25.41 | 26.19 | 24.40 | 24.34 | 23.94 | 1.42 | 2.68 | 0.01007 |
| P63000;P63000-2;A4D2P2;A0A024R9T5;P60763;P15153;B1AH77;B1AH78;B1AH80;J3KSC4;J3QLK0;F8WET9;B1AH79;Q9UJM0;Q5JYX0;Q9UJM1;Q59FQ0;G3V4H1;G3V476;P84095;P60953-1;P60953;Q9H4E5-2;P17081;Q9H4E5;Q7Z513;Q59G91;B4E1U9 | Ras-related C3 botulinum toxin substrate 1;Ras-related C3 botulinum toxin substrate 3;Ras-related C3 botulinum toxin substrate 2 | RAC1;RAC3;RAC2 | 7 | 7 | 40.6 | 21.45 | 25.30 | 25.39 | 25.29 | 23.45 | 24.27 | 24.00 | 1.42 | 2.67 | 0.00421 |
| P25774-2;P25774;U3KQE7;U3KPS4 | Cathepsin S | CTSS | 4 | 4 | 17.8 | 31.626 | 22.29 | 22.33 | 22.21 | 20.69 | 21.13 | 20.79 | 1.41 | 2.66 | 0.00050 |
| P49247;Q53SB2;Q53R32 | Ribose-5-phosphate isomerase | RPIA | 2 | 2 | 8.7 | 33.269 | 21.49 | 21.82 | 22.01 | 20.37 | 20.69 | 20.10 | 1.39 | 2.62 | 0.00370 |
| B2RCX0;A0A024R895;Q01105-2;Q01105-3;Q01105-4;P0DME0 | Protein SET | SET | 8 | 1 | 38.6 | 32.134 | 27.02 | 26.72 | 26.70 | 25.33 | 25.73 | 25.24 | 1.38 | 2.61 | 0.00162 |
| B7Z920;P29144;Q5VZU9 | Tripeptidyl-peptidase 2 | TPP2 | 8 | 8 | 11.1 | 117.96 | 22.51 | 23.12 | 23.12 | 21.69 | 20.97 | 21.96 | 1.38 | 2.60 | 0.01807 |
| P48739;B2R7P6;P48739-2;P48739-3;B3KYB6;B3KYB7;B3KVT6;I3L471;I3L4U7 | Phosphatidylinositol transfer protein beta isoform | PITPNB | 6 | 5 | 35.4 | 31.54 | 23.18 | 23.01 | 22.86 | 21.34 | 21.70 | 21.88 | 1.38 | 2.60 | 0.00173 |
| C9J0J7;P35080-2;G5E9Q6;B4DNH1;D3DNI2;D3DNI3;C9JQ45;C9J2N0 | Profilin-2;Profilin | PFN2 | 6 | 3 | 51.6 | 9.8402 | 24.64 | 24.67 | 24.72 | 23.32 | 23.30 | 23.28 | 1.38 | 2.60 | 0.00000 |
| P61956-2;P61956;H7BZT4;Q6EEV6 | Small ubiquitin-related modifier 2 | SUMO2 | 3 | 2 | 36.6 | 8.1111 | 27.21 | 27.70 | 27.84 | 26.25 | 26.21 | 26.20 | 1.36 | 2.57 | 0.00207 |
| Q9Y6A4 | UPF0468 protein C16orf80 | C16orf80 | 2 | 2 | 10.4 | 22.774 | 20.49 | 21.01 | 20.55 | 18.62 | 19.91 | 19.44 | 1.36 | 2.56 | 0.02948 |
| O95865;H0Y7N1;Q5SSV3;Q5SRR8 | N(G),N(G)-dimethylarginine dimethylaminohydrolase 2 | DDAH2 | 6 | 6 | 32.6 | 29.644 | 22.50 | 22.91 | 22.99 | 21.52 | 21.48 | 21.35 | 1.35 | 2.55 | 0.00110 |
| Q99584;D3DV53 | Protein S100-A13 | S100A13 | 3 | 3 | 31.6 | 11.471 | 23.21 | 23.24 | 22.83 | 22.10 | 21.55 | 21.62 | 1.34 | 2.54 | 0.00347 |
| P30050;Q59FI9;D3DS95;P30050-2;Q76P68 | 60S ribosomal protein L12 | RPL12;hCG_21173 | 5 | 5 | 43 | 17.818 | 24.78 | 24.45 | 24.42 | 23.22 | 23.05 | 23.36 | 1.34 | 2.53 | 0.00079 |
| Q5TFE4;A8K2Z3;Q5TFE4-2;Q5QPD0;H0Y6C1;H0YDA5 | 5-nucleotidase domain-containing protein 1 | NT5DC1 | 11 | 11 | 37.1 | 51.844 | 25.68 | 25.71 | 25.64 | 24.34 | 24.43 | 24.25 | 1.33 | 2.52 | 0.00002 |
| P16403;Q4VB24;P10412;B2R984;A3R0T7;P16402;P22492;A1L407;Q02539 | Histone H1.2;Histone H1.4;Histone H1.3;Histone H1t;Histone H1.1 | HIST1H1C;HIST1H1E;HIST1H1D;HIST1H1T;HIST1H1A | 4 | 4 | 17.8 | 21.364 | 23.73 | 23.99 | 23.46 | 22.12 | 22.74 | 22.33 | 1.33 | 2.51 | 0.00513 |
| Q15843;F8VSA6;E9PS38;S4R3E9;E9PL57 | NEDD8 | NEDD8;NEDD8-MDP1 | 4 | 4 | 40.7 | 9.0714 | 24.56 | 24.14 | 24.93 | 23.13 | 23.18 | 23.34 | 1.32 | 2.50 | 0.00501 |
| Q9UNM1;P61604;A0A024R3X7;B8ZZL8;B8ZZ54;S4R3N1 | 10 kDa heat shock protein, mitochondrial | EPFP1;HSPE1 | 5 | 5 | 54.6 | 10.295 | 24.92 | 24.67 | 25.11 | 23.85 | 23.33 | 23.56 | 1.32 | 2.50 | 0.00267 |
| P62136;P62136-2;E9PMD7;B3KXM2;P62136-3;F5H1L6;F5H037;C0STL0 | Serine/threonine-protein phosphatase PP1-alpha catalytic subunit;Serine/threonine-protein phosphatase | PPP1CA | 9 | 3 | 33.3 | 37.512 | 23.81 | 23.17 | 24.34 | 22.83 | 22.05 | 22.51 | 1.31 | 2.49 | 0.03185 |
| Q9NWY4;A8MVJ9 | UPF0609 protein C4orf27 | C4orf27 | 7 | 7 | 26.9 | 39.436 | 23.01 | 23.42 | 23.30 | 21.85 | 21.97 | 21.98 | 1.31 | 2.48 | 0.00053 |
| P30040;P30040-2;F8VY02 | Endoplasmic reticulum resident protein 29 | ERP29 | 2 | 2 | 8.4 | 28.993 | 22.43 | 22.43 | 22.21 | 20.95 | 21.16 | 21.04 | 1.31 | 2.48 | 0.00016 |
| Q9Y5B9;Q0VGA3;G3V5A4;G3V401;G3V2X0 | FACT complex subunit SPT16 | SUPT16H | 12 | 12 | 12.9 | 119.91 | 25.42 | 25.46 | 25.38 | 23.84 | 24.23 | 24.28 | 1.30 | 2.46 | 0.00074 |
| C9JBI3;P78330;Q53EY1;A0A024RDL9;C9JEJ7;F8WD74 | Phosphoserine phosphatase | PSPH | 6 | 6 | 40.6 | 20.745 | 24.73 | 24.79 | 24.93 | 23.61 | 23.53 | 23.42 | 1.30 | 2.46 | 0.00009 |
| O60664-2;K7ERZ3;O60664-4;O60664;O60664-3 | Perilipin-3 | PLIN3 | 4 | 4 | 32.3 | 28.157 | 23.00 | 22.99 | 23.53 | 21.66 | 22.13 | 21.83 | 1.30 | 2.46 | 0.00455 |
| Q9H993;F5GZY1;B4DPT6 | UPF0364 protein C6orf211 | C6orf211 | 13 | 13 | 38.1 | 51.172 | 24.67 | 25.00 | 25.28 | 23.15 | 23.70 | 24.23 | 1.29 | 2.45 | 0.02241 |
| Q3B7A7;P22102;Q59HH3;Q15374;P22102-2;C9JZG2;C9JKQ7;C9JBJ1;C9JTV6;F8WD69;O14660;B4DJ93 | Trifunctional purine biosynthetic protein adenosine-3;Phosphoribosylamine--glycine ligase;Phosphoribosylformylglycinamidine cyclo-ligase;Phosphoribosylglycinamide formyltransferase | GART | 9 | 9 | 11.6 | 107.72 | 23.41 | 23.70 | 23.67 | 22.34 | 21.96 | 22.67 | 1.27 | 2.42 | 0.00478 |
| Q9P121-3;Q9P121-2;Q9P121;B7Z1I4;Q9P121-4;B7Z9J9;B7Z1Z5;B7Z1H3;H7BZ62;F8VTR5 | Neurotrimin | NTM | 5 | 5 | 19 | 34.984 | 22.89 | 22.60 | 22.77 | 21.27 | 21.57 | 21.61 | 1.27 | 2.41 | 0.00069 |
| Q07960;B4DPZ4;H0YE29;O15376;E9PNR6 | Rho GTPase-activating protein 1 | ARHGAP1 | 8 | 8 | 26.7 | 50.435 | 22.98 | 22.74 | 22.93 | 21.58 | 21.79 | 21.47 | 1.27 | 2.41 | 0.00046 |
| Q9ULC4-2;Q9ULC4;Q9ULC4-3 | Malignant T-cell-amplified sequence 1 | MCTS1 | 2 | 2 | 9.5 | 19.228 | 21.05 | 20.77 | 21.11 | 19.61 | 19.47 | 20.05 | 1.27 | 2.41 | 0.00333 |
| P36551;B4DSD5;H0YA22;D6RER6 | Coproporphyrinogen-III oxidase, mitochondrial | CPOX | 11 | 11 | 30.6 | 50.151 | 25.62 | 25.32 | 25.69 | 24.12 | 24.33 | 24.40 | 1.26 | 2.40 | 0.00087 |
| B4DP70;B4DME8;B4DPN6;F1T0B3;Q92499 | ATP-dependent RNA helicase DDX1 | DDX1 | 5 | 5 | 10.6 | 59.196 | 23.09 | 22.91 | 22.80 | 21.70 | 21.39 | 21.92 | 1.26 | 2.39 | 0.00198 |
| P06132;Q5T446;H0Y5R6;B4DEM5;Q71UD5;B0AZT7;B4DHV6 | Uroporphyrinogen decarboxylase | UROD | 7 | 7 | 30.8 | 40.786 | 24.85 | 24.74 | 25.14 | 23.93 | 23.59 | 23.45 | 1.25 | 2.38 | 0.00252 |
| P05120;B2R7Y0;H7C004;H7BYS2;E7ERB5;E7EPJ9;E9PDK7;Q6LDR6 | Plasminogen activator inhibitor 2 | SERPINB2 | 22 | 22 | 50.8 | 46.596 | 26.94 | 27.12 | 26.92 | 25.85 | 25.74 | 25.63 | 1.25 | 2.38 | 0.00018 |
| Q86W61;P13611-4;D6RGZ6;E9PF17;P13611-3;P13611-2;P13611-5;P13611;Q59FG9 | Versican core protein | VCAN | 2 | 2 | 6.2 | 39.264 | 21.74 | 22.04 | 22.48 | 20.90 | 20.84 | 20.80 | 1.24 | 2.36 | 0.00463 |
| H7C5G1;Q6NVV8;Q05D21;Q2TAA2;B4DMV3;Q6PCA9;C9JE02;C9JDY4;C9J5J2 | Isoamyl acetate-hydrolyzing esterase 1 homolog | IAH1 | 4 | 4 | 23.2 | 25.085 | 23.52 | 23.14 | 23.10 | 21.87 | 22.19 | 21.99 | 1.24 | 2.36 | 0.00160 |
| B7Z4K8;E7ETZ4;Q9Y6E2;B3KM68;Q05D83;B5MCE7;Q75MG1;B5MCH7;E9PFD4;B7Z6N5;C9JF98;F8WDX8;Q96JW5;E7EMS9;D3DN77;Q7L1Q6-2;Q7L1Q6;Q7L1Q6-4;Q53FN7;Q7L1Q6-3 | Basic leucine zipper and W2 domain-containing protein 2 | BZW2 | 3 | 3 | 8.9 | 45.308 | 21.49 | 21.70 | 21.98 | 20.19 | 20.53 | 20.74 | 1.24 | 2.36 | 0.00431 |
| P43490;Q5SYT8;B7Z8W6;F5H246;Q658Z1;C9JF35;C9JG65 | Nicotinamide phosphoribosyltransferase | NAMPT;NAMPTL | 14 | 14 | 39.9 | 55.52 | 25.41 | 25.20 | 25.36 | 24.02 | 24.10 | 24.17 | 1.23 | 2.34 | 0.00008 |
| A2ACR1;P28065-2;P28065;A2ACR0;B4DZW2;A2ACQ9 | Proteasome subunit beta type;Proteasome subunit beta type-9 | PSMB9 | 6 | 6 | 34.7 | 20.96 | 27.40 | 27.29 | 27.70 | 26.22 | 26.45 | 26.04 | 1.23 | 2.34 | 0.00197 |
| P09429;Q5T7C4;Q5T7C6;B7Z965;Q59GW1;B3KQ05;B2RPK0;Q9NYD7;Q5T7C0;Q75MM1;D3DQY9 | High mobility group protein B1 | HMGB1 | 9 | 7 | 39.5 | 24.893 | 25.60 | 25.18 | 25.51 | 24.48 | 24.31 | 23.88 | 1.21 | 2.31 | 0.00528 |
| C9JTY3;C9JUE0;C9JJP5;K0J5S8;K0J6K2;Q05BK6;Q92734-2;Q7Z426;Q92734;Q8TDJ5;Q7Z427 | Protein TFG;Tyrosine-protein kinase receptor | TFG;TFG/ALK fusion | 4 | 4 | 28.1 | 14.759 | 24.23 | 24.09 | 24.23 | 22.88 | 23.20 | 22.87 | 1.20 | 2.30 | 0.00054 |
| Q9NX46;B4DHV5;B7ZAN4 | Poly(ADP-ribose) glycohydrolase ARH3 | ADPRHL2 | 9 | 9 | 31.4 | 38.946 | 25.34 | 25.11 | 25.39 | 24.03 | 24.06 | 24.15 | 1.20 | 2.30 | 0.00021 |
| Q53EY8;P00568;Q5T9B7;H0Y4J6;H0YID2;Q9Y6K8-2;Q9Y6K8-3;Q9Y6K8 | Adenylate kinase isoenzyme 1 | AK1 | 6 | 6 | 39.7 | 21.619 | 24.00 | 23.70 | 24.21 | 22.67 | 22.77 | 22.88 | 1.20 | 2.30 | 0.00172 |
| Q13907;Q13907-2;C9JKM8;C9JD53;Q9BXS1 | Isopentenyl-diphosphate Delta-isomerase 1 | IDI1 | 9 | 9 | 48.5 | 26.319 | 24.73 | 24.39 | 24.81 | 23.44 | 23.23 | 23.67 | 1.20 | 2.29 | 0.00265 |
| Q6IBS0;D6RG15;B4E0A1;H0Y858;H7C5I2;Q9NR96-4 | Twinfilin-2 | TWF2 | 8 | 7 | 33.2 | 39.548 | 24.60 | 24.24 | 24.18 | 23.38 | 23.17 | 22.93 | 1.18 | 2.27 | 0.00299 |
| P30043;M0R192;M0QZL1 | Flavin reductase (NADPH) | BLVRB | 6 | 6 | 49.5 | 22.119 | 24.92 | 24.62 | 24.57 | 23.28 | 23.43 | 23.86 | 1.18 | 2.26 | 0.00464 |
| P32455;B4DNS2;Q8TCE5;P32456 | Interferon-induced guanylate-binding protein 1 | GBP1 | 15 | 2 | 25 | 67.93 | 25.98 | 25.74 | 25.50 | 24.94 | 24.66 | 24.09 | 1.18 | 2.26 | 0.01428 |
| Q13033-2;A0AV58;Q13033 | Striatin-3 | STRN3 | 4 | 4 | 9 | 77.744 | 21.56 | 21.73 | 21.64 | 21.03 | 20.69 | 19.69 | 1.17 | 2.26 | 0.04460 |
| Q96FQ6 | Protein S100-A16 | S100A16 | 3 | 3 | 33 | 11.801 | 23.07 | 23.21 | 22.81 | 22.42 | 21.61 | 21.54 | 1.17 | 2.26 | 0.01877 |
| Q12904;Q12904-2;B4DNK3 | Aminoacyl tRNA synthase complex-interacting multifunctional protein 1;Endothelial monocyte-activating polypeptide 2 | AIMP1 | 9 | 9 | 41.3 | 34.352 | 25.72 | 25.32 | 25.72 | 24.08 | 24.52 | 24.65 | 1.17 | 2.25 | 0.00578 |
| O95336;M0R261;M0R0U3;M0R1L2 | 6-phosphogluconolactonase | PGLS | 17 | 17 | 92.2 | 27.547 | 27.32 | 27.06 | 27.37 | 25.90 | 26.08 | 26.27 | 1.17 | 2.25 | 0.00119 |
| P58546;C9JL85 | Myotrophin | MTPN | 8 | 8 | 78 | 12.895 | 25.62 | 25.82 | 26.05 | 23.91 | 25.08 | 25.01 | 1.17 | 2.24 | 0.04330 |
| B4DQY1;B4DDF7;P30153;A8K3H8;B3KQV6;Q8NB89;F5H3X9;B4DE69;B4E1Q0;E9PH38;C9J9C1;M0QXG4;M0R0K6;H0YDG7;J3KR29;B7Z1G3;P30154-5;P30154-4;P30154;P30154-3;P30154-2;A8K8B0 | Serine/threonine-protein phosphatase 2A 65 kDa regulatory subunit A alpha isoform | PPP2R1A | 5 | 5 | 14.6 | 61.521 | 22.86 | 22.78 | 22.33 | 21.36 | 21.54 | 21.59 | 1.16 | 2.24 | 0.00289 |
| P50579;B4DUX5;F8VQZ7;F8VRR3;Q8NB11;G3XA91;G3V1U3;B3KWL6;Q96B43;F8VSC4;F8VZX9;F8VY03 | Methionine aminopeptidase 2;Methionine aminopeptidase | METAP2 | 13 | 13 | 36.4 | 52.891 | 24.50 | 24.61 | 24.83 | 23.13 | 23.40 | 23.91 | 1.16 | 2.24 | 0.00965 |
| Q9NQ88 | Fructose-2,6-bisphosphatase TIGAR | TIGAR | 5 | 5 | 21.1 | 30.062 | 22.15 | 22.16 | 22.57 | 21.33 | 21.11 | 20.95 | 1.16 | 2.23 | 0.00276 |
| Q9UHX1-4;Q9UHX1-6;Q9UHX1-3;Q9UHX1-5;Q9UHX1-2;Q9UHX1 | Poly(U)-binding-splicing factor PUF60 | PUF60 | 4 | 4 | 6.2 | 54.024 | 23.44 | 23.32 | 22.65 | 22.18 | 21.78 | 21.96 | 1.16 | 2.23 | 0.01298 |
| P54819-2;F8W1A4;P54819;F8VZG5;P54819-3;F8VY04;P54819-6;P54819-5;B4DLK2;P54819-4;G3V213;F8VPP1 | Adenylate kinase 2, mitochondrial;Adenylate kinase 2, mitochondrial, N-terminally processed | AK2 | 8 | 8 | 39.2 | 25.614 | 25.63 | 25.52 | 25.92 | 24.65 | 24.34 | 24.63 | 1.15 | 2.22 | 0.00172 |
| P49903-3;B4DLS1;P49903;P49903-2;P49903-4 | Selenide, water dikinase 1 | SEPHS1 | 2 | 2 | 9.2 | 35.208 | 21.91 | 21.37 | 21.36 | 20.49 | 20.25 | 20.49 | 1.14 | 2.20 | 0.00464 |
| P21589-2;Q6NZX3;Q53Z63;P21589;B2RBH2;Q96B60;H0Y7R7;H0Y3X5;B3KQK1 | 5-nucleotidase | NT5E | 7 | 7 | 16.2 | 57.948 | 23.18 | 22.87 | 23.27 | 21.82 | 21.70 | 22.42 | 1.13 | 2.19 | 0.01119 |
| Q96GD0 | Pyridoxal phosphate phosphatase | PDXP | 4 | 4 | 21.3 | 31.698 | 21.66 | 21.62 | 21.89 | 20.17 | 20.72 | 20.91 | 1.13 | 2.19 | 0.00909 |
| P50281;K4RH61;B2R6P3;Q7KZY0;Q2PW48;Q86VV6;Q9Y5R2;P51511 | Matrix metalloproteinase-14 | MMP14 | 5 | 5 | 9.5 | 65.893 | 25.03 | 25.00 | 25.51 | 24.00 | 24.35 | 23.81 | 1.13 | 2.18 | 0.00776 |
| P02545-2;Q5TCI8;W8QEH3;B4DFR3;Q3BDU5 | Prelamin-A/C;Lamin-A/C | LMNA | 17 | 1 | 29.7 | 65.134 | 27.69 | 27.40 | 27.35 | 26.35 | 26.17 | 26.55 | 1.12 | 2.18 | 0.00186 |
| Q6NSF2;Q3B7A4;F8VWS0;Q53HW2;Q53HK9;P05388;A8K4Z4;F8VZS0;F8VS58;F8VQY6;F8VRK7;G3V210;F8VU65;F8VWV4;F8VPE8;F8VW21;B4E3D5;Q8NHW5;F8W1K8;Q3MHV2 | 60S acidic ribosomal protein P0;60S acidic ribosomal protein P0-like | RPLP0;RPLP0P6 | 6 | 6 | 38.2 | 27.298 | 22.27 | 21.25 | 21.38 | 20.12 | 20.51 | 20.89 | 1.12 | 2.18 | 0.04464 |
| Q14CX7-2;Q14CX7;A8K8X0;F8VSB9 | N-alpha-acetyltransferase 25, NatB auxiliary subunit | NAA25 | 7 | 7 | 10.1 | 99.268 | 23.33 | 23.42 | 23.44 | 22.00 | 22.49 | 22.34 | 1.12 | 2.17 | 0.00167 |
| Q8TEA8;Q496C9 | D-tyrosyl-tRNA(Tyr) deacylase 1 | DTD1 | 3 | 3 | 20.6 | 23.423 | 24.81 | 24.82 | 25.68 | 24.44 | 23.90 | 23.63 | 1.11 | 2.16 | 0.04040 |
| Q12792;Q12792-3;Q12792-4;F8VS81;F8VRG3 | Twinfilin-1 | TWF1 | 6 | 6 | 22.6 | 40.282 | 23.33 | 23.87 | 23.66 | 22.76 | 22.13 | 22.64 | 1.11 | 2.16 | 0.01094 |
| Q9NTK5;J3KQ32;B4DK14;Q9NTK5-3;Q53SQ6;Q9NTK5-2;Q53SW9;C9JTK6;C9JCJ9 | Obg-like ATPase 1 | OLA1;PTD004 | 15 | 15 | 47.7 | 44.743 | 26.39 | 26.21 | 26.13 | 25.16 | 25.06 | 25.18 | 1.11 | 2.16 | 0.00020 |
| I3L2X8;I3L4C0;I3L459;B4E1U1;I3L3W1;I3L4H1;Q00169;F5GWE5;I3NI10;Q6NUL6 | Phosphatidylinositol transfer protein alpha isoform | PITPNA | 2 | 2 | 28.9 | 9.745 | 21.19 | 21.11 | 20.84 | 20.15 | 19.22 | 20.45 | 1.11 | 2.16 | 0.04496 |
| P07737;K7EJ44;I3L3D5 | Profilin-1 | PFN1 | 15 | 15 | 92.9 | 15.054 | 30.39 | 30.11 | 30.37 | 29.26 | 29.00 | 29.29 | 1.10 | 2.15 | 0.00104 |
| P14174 | Macrophage migration inhibitory factor | MIF | 2 | 2 | 17.4 | 12.476 | 26.92 | 26.29 | 26.81 | 25.73 | 25.30 | 25.68 | 1.10 | 2.14 | 0.00929 |
| Q96AT9-2;E7EW52;Q96AT9;C9J6A7;C9JPQ7;Q96AT9-3;C9J9T0;B3KTW7;Q96AT9-4;C9IZU8;V9GZS1;F8WBT4;C9IYE8;E7ESZ6;C9J8S0;Q2QD12 | Ribulose-phosphate 3-epimerase | RPE | 3 | 3 | 21.4 | 23.924 | 22.63 | 22.62 | 22.80 | 21.50 | 21.64 | 21.61 | 1.10 | 2.14 | 0.00011 |
| P52758;H0YBX3;H0YB34 | Ribonuclease UK114 | HRSP12 | 2 | 2 | 19 | 14.494 | 22.21 | 22.13 | 22.56 | 21.75 | 21.33 | 20.51 | 1.10 | 2.14 | 0.04683 |
| O15511;O15511-2;B1ALC0;B3KTE1;Q9BPX5;B3KPC7 | Actin-related protein 2/3 complex subunit 5 | ARPC5 | 3 | 3 | 25.8 | 16.32 | 24.12 | 23.69 | 24.04 | 22.99 | 22.89 | 22.67 | 1.10 | 2.14 | 0.00258 |
| Q14240;Q14240-2;E7EQG2;Q9NZE6;Q96B07;B4DJX6;J3KSN7 | Eukaryotic initiation factor 4A-II | EIF4A2 | 6 | 6 | 18.4 | 46.402 | 24.23 | 24.03 | 23.83 | 22.73 | 22.83 | 23.22 | 1.10 | 2.14 | 0.00437 |
| H0YJS4;G3V4T5;P05198 | Eukaryotic translation initiation factor 2 subunit 1 | EIF2S1 | 2 | 2 | 19 | 28.865 | 20.52 | 20.75 | 20.41 | 19.46 | 19.73 | 19.20 | 1.10 | 2.14 | 0.00382 |
| Q9NUJ1;B7Z6A8;C9IZX5 | Mycophenolic acid acyl-glucuronide esterase, mitochondrial | ABHD10 | 9 | 9 | 32.4 | 33.932 | 24.89 | 24.70 | 24.99 | 23.51 | 23.83 | 23.96 | 1.09 | 2.13 | 0.00229 |
| P31949;B2R5H0 | Protein S100-A11 | S100A11 | 7 | 7 | 82.9 | 11.74 | 26.17 | 26.28 | 26.35 | 25.64 | 25.07 | 24.83 | 1.09 | 2.12 | 0.01163 |
| Q24JP5-4;Q24JP5;Q24JP5-2 | Transmembrane protein 132A | TMEM132A | 2 | 2 | 4.4 | 83.097 | 21.01 | 21.40 | 21.60 | 19.93 | 20.11 | 20.72 | 1.09 | 2.12 | 0.02095 |
| O43707;B4E337;Q96BG6;O43707-2;B4DSX0;O43707-3;F5GXS2;H7C144;Q08043;B2R8Y4;B4DZQ2;B3W6H4;K7EJH8;K7EP19;B7Z4P8;Q9H254-2;C9JRP8;Q71S06;M0QZQ3;Q9H254-4;Q9H254;E9PDB1;A0A024R0N6 | Alpha-actinin-4 | ACTN4 | 10 | 10 | 15.5 | 104.85 | 24.46 | 24.23 | 24.53 | 23.43 | 23.26 | 23.29 | 1.09 | 2.12 | 0.00049 |
| P61088;F8VQQ8;F8VSD4;F8VV71;Q5JXB2;F8VZ29 | Ubiquitin-conjugating enzyme E2 N;Putative ubiquitin-conjugating enzyme E2 N-like | UBE2N;UBE2NL | 10 | 10 | 77.6 | 17.138 | 26.10 | 25.90 | 26.04 | 25.06 | 24.85 | 24.89 | 1.08 | 2.12 | 0.00024 |
| P54136-2;P54136;F5H3T8;B4DXW6 | Arginine--tRNA ligase, cytoplasmic | RARS | 6 | 6 | 12.1 | 67.14 | 23.44 | 23.21 | 23.20 | 21.93 | 22.10 | 22.58 | 1.08 | 2.11 | 0.00667 |
| C9JFR7;P99999;Q6LER6 | Cytochrome c | CYCS | 11 | 11 | 62.4 | 11.333 | 28.35 | 28.39 | 28.80 | 27.37 | 27.42 | 27.53 | 1.07 | 2.11 | 0.00205 |
| O00410;H0Y8C6;O00410-3;B4E0R6;O00410-2;E7ETV3;C9JZD8;E7EQT5;C9JMV5;B3KWG6;C9J875;C9JQT6;E7ESA1;E7ESZ1;E7EWK4;E7EX05;E7ETV8;E7EV12;H0Y3V4;Q9BVS9 | Importin-5 | IPO5 | 6 | 6 | 8.9 | 123.63 | 22.95 | 22.94 | 22.95 | 21.88 | 21.69 | 22.06 | 1.07 | 2.10 | 0.00058 |
| Q9NUQ9;Q68D08;E5RI16;E5RJL8;Q9NW21;E5RJE1;E5RIR8;Q3ZTR9;E5RGI7;E5RK61;E5RFS4;E5RHU5 | Protein FAM49B | FAM49B;DKFZp686B04128 | 10 | 10 | 42.6 | 36.748 | 24.92 | 24.90 | 24.69 | 23.39 | 23.96 | 23.94 | 1.07 | 2.10 | 0.00586 |
| Q9NQR4;H7C579;B4DFB1;B7Z3F9;F8WF70 | Omega-amidase NIT2 | NIT2 | 11 | 11 | 60.9 | 30.608 | 24.27 | 24.03 | 24.47 | 23.20 | 23.13 | 23.25 | 1.06 | 2.09 | 0.00125 |
| Q6LET3;P00492 | Hypoxanthine-guanine phosphoribosyltransferase | HPRT1 | 11 | 11 | 69.7 | 24.588 | 25.43 | 25.32 | 25.45 | 24.43 | 24.29 | 24.29 | 1.06 | 2.09 | 0.00008 |
| E7EQ12;E7EQA0;Q86YM9;H0Y7F0;P20810-3;Q6AZE3;E9PDE4;B7Z6N0;E7ES10;B7Z5T9;P20810-4;E9PCH5;P20810-8;B7Z574;P20810-2;P20810;P20810-9;B7Z468;P20810-5;E7ESM9;P20810-7;P20810-6;B7Z8S8;B7Z7L4;B2RDU1;A8K8C4;H0YD33;H0Y9H6;B2RCJ7;E7EVY3;B7Z4U9;Q59HE3;A0A024RAN2;Q15786;B7Z5T6;D6RC54 | Calpastatin | CAST | 5 | 5 | 17.3 | 46.133 | 24.01 | 23.83 | 23.75 | 22.86 | 22.56 | 23.00 | 1.06 | 2.08 | 0.00219 |
| P31153;B4DEX8;B4DN45;B4E1K2;B4DN74;B4DFQ8;Q00266;A8K455 | S-adenosylmethionine synthase isoform type-2;S-adenosylmethionine synthase | MAT2A | 12 | 12 | 37.2 | 43.66 | 25.09 | 24.81 | 25.20 | 24.07 | 24.00 | 23.88 | 1.06 | 2.08 | 0.00117 |
| P68036-3;P68036;A8K4W8;P68036-2;I1SRC5 | Ubiquitin-conjugating enzyme E2 L3 | UBE2L3 | 9 | 9 | 54.2 | 24.003 | 26.33 | 26.46 | 26.04 | 25.15 | 25.18 | 25.35 | 1.05 | 2.07 | 0.00160 |
| Q15149-4;Q15149-7;Q15149-8;Q15149-9;Q15149-5;Q15149-6;Q15149-3;Q15149-2;Q15149;E9PMV1;E9PKG0;E9PIA2;E9PQ28;D3DWL0;Q96IE3;Q8WXV6 | Plectin | PLEC | 18 | 18 | 4.5 | 516.19 | 27.32 | 27.31 | 27.32 | 26.27 | 26.22 | 26.32 | 1.05 | 2.07 | 0.00000 |
| Q676U5-5;A0A024R4B6;Q53SV2;B7ZLM5;Q17RG0;Q676U5-2;L7UQD0;A5YM60;Q676U5;E7EVC7;Q8TCI4;Q9NWG8;Q676U5-4;Q676U5-3 | Autophagy-related protein 16-1 | ATG16L1;FLJ10035 | 2 | 2 | 8.1 | 49.485 | 21.39 | 21.54 | 21.86 | 20.32 | 20.42 | 20.95 | 1.04 | 2.05 | 0.01241 |
| O43396;B2R960;Q59G46;K7ER96;G3V1K0;K7EML9;B3KT45;K7EKG2;K7EPB7 | Thioredoxin-like protein 1 | TXNL1 | 9 | 9 | 43.3 | 32.251 | 24.58 | 24.83 | 25.19 | 23.97 | 23.79 | 23.74 | 1.04 | 2.05 | 0.00531 |
| P55263;P55263-2;P55263-3;P55263-4;Q86U79 | Adenosine kinase | ADK | 9 | 9 | 28.5 | 40.545 | 23.75 | 24.05 | 24.39 | 22.75 | 23.13 | 23.20 | 1.03 | 2.05 | 0.01109 |
| B4DZT3;P20700 | Lamin-B1 | LMNB1 | 4 | 4 | 20.2 | 43.033 | 24.12 | 24.28 | 24.51 | 23.35 | 23.47 | 22.99 | 1.03 | 2.05 | 0.00493 |
| E7EMF1;E7ESP4;P17301;E9PB77;Q71V33;D6RG08 | Integrin alpha-2 | ITGA2 | 8 | 8 | 12.5 | 88.561 | 22.87 | 22.51 | 22.56 | 21.38 | 21.37 | 22.10 | 1.03 | 2.04 | 0.01805 |
| B4DQJ8;P52209;F5H7U0;B4E2U0;B4DL86;B4DV68;K7EM49;K7EMN2;K7EPF6;A9Z1X1;K7ELN9 | 6-phosphogluconate dehydrogenase, decarboxylating | PGD | 29 | 29 | 64.7 | 51.872 | 29.22 | 29.42 | 29.66 | 28.37 | 28.40 | 28.43 | 1.03 | 2.04 | 0.00129 |
| P11142;E9PKE3;Q53GZ6;Q96IS6;B3KTV0;Q53HF2;P11142-2;E9PNE6;B4E1Q1;E9PN89;A8K7Q2;B4DTX2;E9PLF4;E9PQQ4;E9PQK7;E9PK54;E9PPY6;E9PN25;E9PI65;E9PS65;Q96BE0;Q96H53;E9PM13;Q9NZ87;Q9NWW3;A4D111 | Heat shock cognate 71 kDa protein | HSPA8 | 35 | 25 | 57.6 | 70.897 | 30.54 | 30.36 | 30.53 | 29.44 | 29.17 | 29.72 | 1.03 | 2.04 | 0.00357 |
| Q9UJ68-2;Q9UJ68-3;Q9UJ68-4;Q9UJ68-5;Q9UJ68;H0YAN3;B7Z694;E5RIA9 | Mitochondrial peptide methionine sulfoxide reductase | MSRA | 3 | 3 | 24.9 | 18.963 | 23.05 | 23.02 | 23.56 | 22.16 | 22.17 | 22.20 | 1.03 | 2.04 | 0.00414 |
| B4DMK0;Q15907;P62491-2;J3KQP6;H3BMH2;H3BSC1;P62491;B4DMN1;M0R2D0;B4DQU5;P57735 | Ras-related protein Rab-11B;Ras-related protein Rab-11A | RAB11B;RAB11A | 6 | 6 | 39.1 | 19.971 | 24.33 | 24.15 | 24.12 | 23.12 | 22.94 | 23.48 | 1.02 | 2.03 | 0.00398 |
| J3QS39;J3QTR3;F5H6Q2;P62987;F5GYU3;F5H2Z3;F5H265;Q5UGI3;B4DV12;F5H388;Q5RKT7;P62979;F5H747;F5GXK7;J3QKN0;Q5PY61;P0CG47;Q96C32;Q96MH4;L8B4I8;L8B196;L8B4R0;F5H041;A8K674;P0CG48;L8B4Z6;L8B4M0;L8B4J3;Q9UFQ0;Q96H31;Q66K58;Q59EM9;M0R1V7;Q49A90;M0R1M6;M0R2S1;F5GZ39;J3QLP7;J3QRK5;J3QSA3;Q8WYN9;K7EMA8;J3KSM4 | Ubiquitin-60S ribosomal protein L40;Ubiquitin;60S ribosomal protein L40;Ubiquitin-40S ribosomal protein S27a;Ubiquitin;40S ribosomal protein S27a;Polyubiquitin-B;Ubiquitin;Polyubiquitin-C;Ubiquitin | UBB;RPS27A;UBC;UBA52;UbC;DKFZp434K0435;UBBP4 | 13 | 13 | 79.6 | 10.469 | 29.84 | 29.81 | 30.23 | 28.78 | 29.03 | 29.01 | 1.02 | 2.02 | 0.00286 |
| P30520;B4E1L0 | Adenylosuccinate synthetase isozyme 2 | ADSS | 12 | 12 | 32.9 | 50.097 | 25.40 | 25.46 | 25.43 | 24.43 | 24.30 | 24.51 | 1.01 | 2.02 | 0.00009 |
| P12814;P12814-3;P12814-2;P12814-4;H9KV75;B7Z565;Q86TX4;B4DFY0;B7Z2W3;B3KUX9;H7C5W8;B4DRP5;G3V2N5;G3V2W4;H0YJ11;A1L0V1;H0YJW3;P35609-2;P35609;G3V2X9;B7Z2N5;G3V5M4;Q5ZEZ4;B7Z4V1;B7Z4P6;Q59FD9;B7Z4K1;G3V2E8;G3V380 | Alpha-actinin-1 | ACTN1 | 39 | 26 | 50.8 | 103.06 | 27.39 | 26.69 | 27.13 | 25.97 | 26.02 | 26.18 | 1.01 | 2.02 | 0.00914 |
| P48444-2;P48444;Q6MZV5;B0YIW6 | Coatomer subunit delta | ARCN1;DKFZp686M09245 | 14 | 14 | 29.1 | 47.204 | 27.44 | 27.52 | 27.63 | 26.42 | 26.63 | 26.50 | 1.01 | 2.01 | 0.00026 |
| P30046;B7Z522;B4DJQ7;J3KQ18;A6NHG4;B5MC82;B4E259;H7C342 | D-dopachrome decarboxylase;D-dopachrome decarboxylase-like protein | DDT;DDTL | 8 | 8 | 63.6 | 12.712 | 25.91 | 26.02 | 26.22 | 24.91 | 24.95 | 25.27 | 1.01 | 2.01 | 0.00220 |
| P62826;B5MDF5;J3KQE5;F5H018;H0YFC6;B4DV51;Q96QB7;Q0EFC9 | GTP-binding nuclear protein Ran | RAN | 10 | 10 | 44.9 | 24.423 | 25.97 | 25.84 | 25.89 | 24.99 | 24.90 | 24.79 | 1.01 | 2.01 | 0.00014 |
| Q15404;Q15404-2;Q32Q10;B0YJ73 | Ras suppressor protein 1 | RSU1 | 9 | 9 | 52.3 | 31.54 | 24.56 | 24.36 | 24.25 | 23.36 | 23.55 | 23.26 | 1.00 | 2.01 | 0.00125 |
| P61086;D6RDM7;B4DIZ2;P61086-3;P61086-2;B3KSH4;D6RFX1 | Ubiquitin-conjugating enzyme E2 K | UBE2K | 6 | 6 | 45.5 | 22.406 | 23.35 | 23.59 | 23.53 | 22.48 | 22.46 | 22.52 | 1.00 | 2.00 | 0.00019 |
| B7Z754 |  |  | 23 | 0 | 36.1 | 108 | 26.91 | 26.92 | 27.10 | 27.81 | 28.06 | 28.14 | -1.02 | -2.03 | 0.00092 |
| B4E1V6;O75503;A0A024R644 | Ceroid-lipofuscinosis neuronal protein 5 | CLN5 | 3 | 3 | 10.6 | 31.982 | 23.36 | 23.56 | 23.67 | 24.44 | 24.82 | 24.43 | -1.03 | -2.04 | 0.00285 |
| P01008;Q8TCE1;Q9UE54;Q8IZZ8 | Antithrombin-III | SERPINC1 | 8 | 8 | 13.4 | 52.602 | 29.52 | 29.59 | 29.70 | 30.46 | 30.73 | 30.72 | -1.03 | -2.05 | 0.00057 |
| B4DQ98;Q01459;B2RBF5;B3KQS3;Q8TC97 | Di-N-acetylchitobiase | CTBS | 13 | 13 | 39.3 | 43.433 | 26.97 | 27.07 | 27.28 | 27.79 | 28.28 | 28.37 | -1.04 | -2.06 | 0.00670 |
| B4E3S3;Q9H6B4 | CXADR-like membrane protein | CLMP | 2 | 2 | 18.1 | 21.068 | 22.83 | 22.34 | 22.05 | 23.07 | 23.63 | 23.66 | -1.04 | -2.06 | 0.02462 |
| P07858;A8K2H4;B4DL49;B4DMY4;B3KUJ8;E9PKQ7;E9PNL5;E9PHZ5;E9PLY3;E9PSG5;E9PQM1;E9PR54;E9PJ67;E9PCB3;E9PS78;R4GMQ5;Q5HYG5;E9PIS1;E9PL32;E9PKX0;Q8TAC7 | Cathepsin B;Cathepsin B light chain;Cathepsin B heavy chain | CTSB | 28 | 21 | 67.6 | 37.821 | 29.87 | 30.00 | 30.15 | 30.99 | 31.04 | 31.14 | -1.05 | -2.07 | 0.00032 |
| Q53GP1;P51688;F5H6A3;B3KTR7;I3NI22;I3L4B7;Q59EB1;I3L2L4;I3L3T3;I3L2I6 | N-sulphoglucosamine sulphohydrolase | SGSH | 15 | 15 | 41.8 | 56.642 | 26.68 | 26.99 | 27.02 | 27.51 | 28.23 | 28.11 | -1.05 | -2.08 | 0.01346 |
| Q9UHL4;Q59EM4;R4GNE8;B4DPD6;R4GMR2;R4GN05;R4GMV4;R4GMU5 | Dipeptidyl peptidase 2 | DPP7 | 14 | 14 | 37 | 54.341 | 26.77 | 27.01 | 26.94 | 27.85 | 28.13 | 27.93 | -1.07 | -2.09 | 0.00064 |
| F8W1Q3;P43251;P43251-3;P43251-2;B7Z7C9;C9JSN9 | Biotinidase | BTD | 11 | 11 | 26.8 | 58.913 | 24.67 | 25.06 | 24.81 | 25.92 | 25.85 | 25.99 | -1.08 | -2.11 | 0.00088 |
| Q99715-4;D6RGG3;Q99715;Q99715-2;B9EJB8;H0Y4P7 | Collagen alpha-1(XII) chain | COL12A1 | 36 | 36 | 13.5 | 324.57 | 27.00 | 26.94 | 26.89 | 28.05 | 27.95 | 28.06 | -1.08 | -2.11 | 0.00002 |
| P10253;I3L3L3;I3L0S5;I3L2V9 | Lysosomal alpha-glucosidase;76 kDa lysosomal alpha-glucosidase;70 kDa lysosomal alpha-glucosidase | GAA | 35 | 1 | 49.7 | 105.32 | 27.92 | 28.00 | 28.14 | 28.90 | 29.27 | 29.14 | -1.09 | -2.12 | 0.00101 |
| O14672;B4DU28;A0AV88;H0YNC5 | Disintegrin and metalloproteinase domain-containing protein 10 | ADAM10 | 9 | 9 | 18.6 | 84.141 | 24.46 | 24.42 | 24.83 | 25.30 | 25.74 | 25.98 | -1.10 | -2.15 | 0.00958 |
| O75882-2;O75882-3;O75882;B4DZ36 | Attractin | ATRN | 32 | 32 | 27.8 | 141.43 | 27.43 | 27.74 | 27.75 | 28.71 | 28.76 | 28.77 | -1.11 | -2.16 | 0.00049 |
| P21781;Q9NSJ0;Q6RK68;H0YNE7;P21781-2 | Fibroblast growth factor 7 | FGF7;PRED3 | 3 | 3 | 15.5 | 22.509 | 22.06 | 21.99 | 22.57 | 23.21 | 23.62 | 23.13 | -1.11 | -2.16 | 0.00912 |
| P09603;A0A024R0A1;Q59H07;P09603-3;E9PJA2;P09603-2;B4DTX0;E9PKP4;F6MF51;E9PQ08;H7BY18 | Macrophage colony-stimulating factor 1;Processed macrophage colony-stimulating factor 1 | CSF1 | 9 | 9 | 17.1 | 60.179 | 26.36 | 26.78 | 26.19 | 27.54 | 27.38 | 27.77 | -1.12 | -2.18 | 0.00579 |
| B4DJQ8;P53634;H0YCY8;I3V9V3;I3V9T0;I3V9V6;P53634-2;P53634-3 | Dipeptidyl peptidase 1;Dipeptidyl peptidase 1 exclusion domain chain;Dipeptidyl peptidase 1 heavy chain;Dipeptidyl peptidase 1 light chain | CTSC | 21 | 21 | 46 | 50.151 | 29.51 | 29.66 | 29.68 | 30.54 | 30.66 | 31.02 | -1.13 | -2.18 | 0.00192 |
| Q99574;C9JQU8;C9JDY5;H7C5T9 | Neuroserpin | SERPINI1 | 8 | 8 | 22.2 | 46.426 | 23.34 | 23.65 | 23.65 | 24.27 | 24.90 | 24.84 | -1.13 | -2.18 | 0.00743 |
| P15289-2;P15289;Q63HL5;A0A024R509;B4DVI5 | Arylsulfatase A;Arylsulfatase A component B;Arylsulfatase A component C | ARSA;DKFZp686G12235 | 12 | 12 | 44 | 44.881 | 25.11 | 25.19 | 25.31 | 26.15 | 26.67 | 26.22 | -1.14 | -2.21 | 0.00282 |
| J3QQU6;J3KSN0;J3QKK2;J3KTR4;Q8WVN6;Q53G63;Q53G27;A8K3U3 | Secreted and transmembrane protein 1 | SECTM1 | 4 | 4 | 46.3 | 15.921 | 26.75 | 26.47 | 26.91 | 27.34 | 28.12 | 28.11 | -1.15 | -2.22 | 0.01654 |
| Q12841;Q12841-2;B4DTZ8;C9J5G4;Q9BZQ0;H7C4W4 | Follistatin-related protein 1 | FSTL1 | 20 | 20 | 58.1 | 34.985 | 29.02 | 28.60 | 29.05 | 30.28 | 29.83 | 30.01 | -1.15 | -2.22 | 0.00424 |
| P98172 | Ephrin-B1 | EFNB1 | 3 | 3 | 15.6 | 38.006 | 23.79 | 23.83 | 24.34 | 25.23 | 25.15 | 25.05 | -1.16 | -2.23 | 0.00332 |
| Q12797-10;Q12797 | Aspartyl/asparaginyl beta-hydroxylase | ASPH | 6 | 6 | 12.3 | 83.267 | 22.12 | 21.78 | 21.87 | 23.00 | 23.39 | 22.89 | -1.17 | -2.24 | 0.00303 |
| Q92484;Q92484-2 | Acid sphingomyelinase-like phosphodiesterase 3a | SMPDL3A | 7 | 7 | 21.9 | 51.26 | 24.75 | 24.70 | 25.09 | 26.16 | 25.95 | 26.02 | -1.20 | -2.29 | 0.00095 |
| A8K5J8;B4DPQ0;Q53HU9;Q53HT9;P00736;H0YFH3;B4E1B0;R4GMN6;F6T9X8;F5GZR1;D3DUT5;F5H2D0;F5H1V0;F5H6Y3;F5H3N3;F5H1N6;F5GWL0;F5H3A3;H0YFL7;Q9H804;Q9NZP8 | Complement C1r subcomponent;Complement C1r subcomponent heavy chain;Complement C1r subcomponent light chain | C1R | 30 | 30 | 53.3 | 80.198 | 27.87 | 27.69 | 27.63 | 29.05 | 28.87 | 28.91 | -1.21 | -2.31 | 0.00018 |
| Q9UBR2;Q5U000 | Cathepsin Z | CTSZ | 15 | 15 | 46.2 | 33.868 | 28.98 | 29.09 | 29.33 | 30.13 | 30.52 | 30.41 | -1.22 | -2.33 | 0.00142 |
| P13497;A5PLK9;P13497-4;P13497-5;P13497-2;Q59F71;P13497-6 | Bone morphogenetic protein 1 | BMP1 | 2 | 2 | 2 | 111.25 | 21.69 | 21.19 | 21.56 | 23.12 | 22.66 | 22.33 | -1.22 | -2.33 | 0.01163 |
| B5MBX2;P20062;P20062-2;C9J6W9 | Transcobalamin-2 | TCN2 | 15 | 15 | 39.7 | 47.142 | 25.81 | 25.64 | 25.57 | 26.78 | 26.88 | 27.18 | -1.27 | -2.42 | 0.00079 |
| P09486;D3DQH8;B4DRV4;B2RDL6;F5GY03;E5RK62;Q6QE20;F5H4E2 | SPARC | SPARC | 21 | 21 | 63.4 | 34.632 | 32.06 | 31.87 | 32.03 | 33.15 | 33.19 | 33.45 | -1.28 | -2.42 | 0.00033 |
| P35475;A8K701;D6REB5;Q56A80;B3KWK6;D3DVN7;D3DVN8;H0Y9B3;D6R9D5;D6RBD5;Q59H58 | Alpha-L-iduronidase | IDUA | 18 | 18 | 34.5 | 72.669 | 25.08 | 25.42 | 25.50 | 26.45 | 26.68 | 26.71 | -1.28 | -2.43 | 0.00111 |
| P08123;B4DTF5 | Collagen alpha-2(I) chain | COL1A2 | 15 | 13 | 13.3 | 129.31 | 28.11 | 27.94 | 28.16 | 29.50 | 29.34 | 29.23 | -1.29 | -2.44 | 0.00024 |
| D6REQ6;D6RHI9;O00584;A6XND5;H0YAE9 | Ribonuclease T2 | RNASET2 | 7 | 7 | 41.7 | 25.308 | 25.74 | 25.67 | 26.02 | 26.80 | 27.28 | 27.23 | -1.29 | -2.45 | 0.00227 |
| P09871;A8K2N0;B3KNX0;F8WCZ6;H0Y5D1;F5H7T4;B5MCV4 | Complement C1s subcomponent;Complement C1s subcomponent heavy chain;Complement C1s subcomponent light chain | C1S | 25 | 24 | 45.2 | 76.684 | 27.69 | 27.65 | 27.75 | 29.07 | 29.01 | 28.90 | -1.30 | -2.46 | 0.00002 |
| C0LQF2 |  |  | 8 | 1 | 25.9 | 38.181 | 27.57 | 27.74 | 27.72 | 28.77 | 29.27 | 28.92 | -1.31 | -2.49 | 0.00112 |
| Q9Y646;B4DJC2;E5RJA8;E5RH35;E5RJZ7;E5RJP8;B4E1W2;H0YBB7 | Carboxypeptidase Q | CPQ | 13 | 13 | 39 | 51.887 | 26.96 | 27.10 | 27.23 | 28.11 | 28.57 | 28.55 | -1.31 | -2.49 | 0.00149 |
| B4E1H2;H9KV48;P05155;B4E1F0;E9PGN7;Q5UGI6;H0YCA1 | Plasma protease C1 inhibitor | SERPING1 | 10 | 10 | 26.8 | 49.757 | 24.89 | 24.99 | 25.06 | 25.89 | 26.28 | 26.74 | -1.32 | -2.50 | 0.00639 |
| O14498;H0YN67;H0YL90 | Immunoglobulin superfamily containing leucine-rich repeat protein | ISLR | 5 | 5 | 15 | 45.997 | 23.24 | 22.61 | 22.40 | 24.16 | 24.24 | 23.91 | -1.35 | -2.55 | 0.00753 |
| P17900;Q14427;H0YBY3;B4DQM5;E5RJD0 | Ganglioside GM2 activator;Ganglioside GM2 activator isoform short | GM2A | 8 | 8 | 45.6 | 20.838 | 24.46 | 23.54 | 23.14 | 25.17 | 25.08 | 24.96 | -1.36 | -2.56 | 0.02641 |
| Q9HAT2;Q9HAT2-2 | Sialate O-acetylesterase | SIAE | 6 | 6 | 14.3 | 58.314 | 23.65 | 23.58 | 24.03 | 25.29 | 25.24 | 24.87 | -1.38 | -2.61 | 0.00200 |
| B4DR21;Q14703;A6NMF3 | Membrane-bound transcription factor site-1 protease | MBTPS1 | 4 | 4 | 5.5 | 100.49 | 22.32 | 21.84 | 21.83 | 23.69 | 23.19 | 23.26 | -1.38 | -2.61 | 0.00358 |
| A0PJG0;P07996;A0A024R9Q1;Q59E99;B4E3J7;A8MZG1 | Thrombospondin-1 | THBS1 | 11 | 11 | 31.9 | 43.943 | 24.47 | 24.46 | 24.68 | 25.90 | 25.83 | 26.11 | -1.41 | -2.65 | 0.00023 |
| O00462;A8K6D3;A7LFP5;E9PFW2;B4DT18;Q59EG5;Q68EA4 | Beta-mannosidase | MANBA | 25 | 24 | 32.3 | 100.89 | 26.19 | 26.23 | 26.41 | 27.67 | 27.66 | 27.76 | -1.42 | -2.68 | 0.00005 |
| Q86UD1;E9PJ29 | Out at first protein homolog | OAF | 6 | 6 | 32.2 | 30.688 | 24.00 | 23.98 | 24.07 | 25.80 | 25.38 | 25.18 | -1.44 | -2.71 | 0.00144 |
| P20908;B2ZZ86;Q59EE7;Q96HC0;H7BY82 | Collagen alpha-1(V) chain | COL5A1 | 11 | 11 | 9 | 183.56 | 26.28 | 26.23 | 26.56 | 27.64 | 28.06 | 27.73 | -1.46 | -2.74 | 0.00092 |
| P01023;H0YFH1;H0YGH6;P20742-2;F8W7L3;Q9BQ22;B7Z7M2 | Alpha-2-macroglobulin | A2M | 70 | 60 | 58.9 | 163.29 | 30.17 | 30.31 | 30.37 | 31.51 | 31.82 | 31.94 | -1.47 | -2.77 | 0.00048 |
| Q9NPR5;Q12913-2;Q12913;E9PPH3 | Receptor-type tyrosine-protein phosphatase eta | PTPRJ | 4 | 4 | 14.6 | 45.078 | 21.06 | 20.53 | 20.69 | 22.06 | 22.46 | 22.22 | -1.49 | -2.80 | 0.00166 |
| Q59GA0;E9PIM6;P04216;E9PNQ8;J3QRJ3 | Thy-1 membrane glycoprotein | THY1 | 4 | 4 | 27.6 | 15.904 | 23.89 | 23.57 | 23.78 | 25.02 | 25.34 | 25.34 | -1.49 | -2.80 | 0.00046 |
| B4E2A0;P15924-2;Q4LE79;P15924-3;P15924;G1UI31;B4DKX6;Q6ZP16;B4DWT7 | Desmoplakin | DSP;DSP variant protein | 4 | 4 | 3.1 | 156.3 | 21.34 | 21.42 | 20.84 | 22.91 | 22.64 | 22.61 | -1.52 | -2.87 | 0.00175 |
| Q3SXP2;Q7Z5L0 | Vitelline membrane outer layer protein 1 homolog | VMO1 | 2 | 2 | 19.2 | 16.104 | 21.68 | 21.64 | 21.92 | 23.20 | 22.99 | 23.61 | -1.52 | -2.87 | 0.00165 |
| P48740-2;P48740-4;Q5HYM1;P48740;Q6MZL2;C9JMA2;Q9NSY8 | Mannan-binding lectin serine protease 1;Mannan-binding lectin serine protease 1 heavy chain;Mannan-binding lectin serine protease 1 light chain | MASP1;DKFZp686O1553 | 21 | 11 | 38.7 | 81.859 | 26.46 | 26.27 | 26.49 | 28.20 | 27.71 | 27.91 | -1.53 | -2.89 | 0.00062 |
| D6RFL4;P08571;F1C4A7;B2R888 | Monocyte differentiation antigen CD14;Monocyte differentiation antigen CD14, urinary form;Monocyte differentiation antigen CD14, membrane-bound form;Monocyte differentiation antigen CD14 | CD14 | 3 | 3 | 17.8 | 23.313 | 21.39 | 21.34 | 21.05 | 22.58 | 22.42 | 23.39 | -1.53 | -2.90 | 0.00851 |
| Q59EG0 |  |  | 14 | 1 | 6.9 | 246.41 | 27.62 | 27.60 | 27.79 | 29.22 | 29.09 | 29.36 | -1.55 | -2.94 | 0.00009 |
| Q13822;E7EUF1;Q13822-3;Q13822-2;E5RIA2;B4DJD3;E5RJ49;E5RIB9 | Ectonucleotide pyrophosphatase/phosphodiesterase family member 2 | ENPP2 | 33 | 33 | 44.6 | 98.993 | 27.07 | 27.25 | 27.35 | 28.77 | 28.57 | 29.02 | -1.56 | -2.96 | 0.00054 |
| B4E1S6;P31431-2;P31431;M1VE83;M1VKI3 | Syndecan;Syndecan-4;Tyrosine-protein kinase receptor | SDC4;SDC4-ROS1_S4;R34;SDC4-ROS1_S4;R32 | 4 | 4 | 28.6 | 13.91 | 24.30 | 23.54 | 23.74 | 25.38 | 26.15 | 24.77 | -1.57 | -2.98 | 0.02629 |
| O75326;O75326-2;F5GYX3;B3KMH6;H3BMF9 | Semaphorin-7A | SEMA7A | 22 | 22 | 39.6 | 74.823 | 25.62 | 25.82 | 25.87 | 27.29 | 27.36 | 27.47 | -1.60 | -3.03 | 0.00006 |
| P24593;C9JXX4 | Insulin-like growth factor-binding protein 5 | IGFBP5 | 6 | 6 | 20.2 | 30.57 | 25.49 | 24.44 | 24.68 | 27.05 | 26.17 | 26.37 | -1.66 | -3.17 | 0.01590 |
| P02461-2;E7ENY8;P02461 | Collagen alpha-1(III) chain | COL3A1 | 13 | 13 | 13 | 111.91 | 26.57 | 26.35 | 27.08 | 28.45 | 28.38 | 28.20 | -1.67 | -3.19 | 0.00184 |
| P51884;Q53FV4 | Lumican | LUM | 22 | 22 | 46.7 | 38.429 | 30.94 | 30.50 | 30.69 | 32.39 | 32.28 | 32.69 | -1.75 | -3.36 | 0.00059 |
| P14923;A0A024R1X8 | Junction plakoglobin | JUP | 2 | 2 | 5.1 | 81.744 | 20.07 | 19.32 | 18.96 | 21.56 | 21.86 | 20.57 | -1.88 | -3.68 | 0.02098 |
| Q08431-2;F5GZN3;B4E396;F5H7N9;B3KTQ2;Q08431;X6R3G6;Q08431-3;H0YKS8 | Lactadherin;Lactadherin short form;Medin | MFGE8 | 5 | 5 | 18.3 | 35.188 | 21.45 | 21.41 | 21.27 | 22.77 | 23.46 | 23.69 | -1.93 | -3.81 | 0.00240 |
| E9PEH6;B4DP78;C9JR67;O43556;O43556-3;O43556-4;B7Z2R4 | Epsilon-sarcoglycan | SGCE | 2 | 2 | 4.8 | 45.068 | 20.22 | 20.08 | 20.04 | 22.42 | 22.18 | 21.58 | -1.94 | -3.85 | 0.00162 |
| Q7Z5L7;Q7Z5L7-2;Q7Z5L7-3;B4DUY6;B2RA94;Q7Z5L7-4 | Podocan | PODN | 5 | 5 | 9.8 | 68.975 | 20.53 | 20.49 | 19.89 | 22.17 | 22.33 | 22.41 | -2.00 | -4.00 | 0.00080 |
| P27487;F8WE17 | Dipeptidyl peptidase 4;Dipeptidyl peptidase 4 membrane form;Dipeptidyl peptidase 4 soluble form | DPP4 | 5 | 5 | 9.4 | 88.278 | 21.74 | 21.92 | 21.90 | 23.97 | 23.97 | 23.67 | -2.02 | -4.05 | 0.00006 |
| Q14393-5;Q14393-4;B4DZY7;Q14393-3;Q14393-2;Q14393;J3KP07;Q658L2 | Growth arrest-specific protein 6 | GAS6;DKFZp666G247 | 6 | 6 | 23.5 | 41.986 | 21.85 | 21.12 | 21.23 | 23.56 | 23.73 | 23.03 | -2.04 | -4.11 | 0.00278 |
| P10124;A0A024QZL1 | Serglycin | SRGN | 4 | 4 | 31.6 | 17.652 | 23.58 | 23.84 | 23.66 | 25.98 | 25.57 | 25.68 | -2.05 | -4.14 | 0.00014 |
| P02452;I3L3H7;Q6LAN8;H9C5C5 | Collagen alpha-1(I) chain | COL1A1 | 12 | 2 | 9.5 | 138.94 | 27.02 | 26.81 | 27.26 | 29.12 | 29.10 | 29.02 | -2.05 | -4.14 | 0.00010 |
| P36222;H0Y3U8;B3KTE6;Q59FK1 | Chitinase-3-like protein 1 | CHI3L1 | 9 | 9 | 31.6 | 42.625 | 23.17 | 23.36 | 23.41 | 25.10 | 25.65 | 25.40 | -2.07 | -4.20 | 0.00030 |
| P49788;P49788-2 | Retinoic acid receptor responder protein 1 | RARRES1 | 8 | 8 | 27.9 | 33.285 | 23.59 | 23.45 | 23.46 | 25.29 | 25.90 | 25.67 | -2.12 | -4.34 | 0.00032 |
| C9JMN2;P12107-4;P12107-3;D3DT71;P12107;P12107-2;D3DT72;H7C381 | Collagen alpha-1(XI) chain | COL11A1 | 5 | 5 | 16 | 61.87 | 22.21 | 21.74 | 22.45 | 24.05 | 24.56 | 24.21 | -2.14 | -4.41 | 0.00116 |
| P35052;A0A024R4D5;H7C410;H7C024;P35052-2;Q59GI7;H7BZL4;H7BZE9;C9J4Y6;Q53EX3 | Glypican-1;Secreted glypican-1 | GPC1 | 25 | 25 | 51.1 | 61.68 | 27.68 | 27.68 | 27.87 | 30.01 | 29.83 | 30.26 | -2.29 | -4.88 | 0.00008 |
| Q86YZ3 | Hornerin | HRNR | 5 | 5 | 8.6 | 282.39 | 23.44 | 23.91 | 22.48 | 25.83 | 25.28 | 25.85 | -2.37 | -5.18 | 0.00673 |
| Q9UI42-2;Q9UI42;B7Z5J4;B7Z577;C9J6N7;C9J3P4;F8WF19 | Carboxypeptidase A4 | CPA4 | 12 | 12 | 41.5 | 43.57 | 23.77 | 23.88 | 24.13 | 26.37 | 26.32 | 26.49 | -2.47 | -5.54 | 0.00003 |
| P36955;I3L4N7;I3L107;I3L4F9;I3L4Z0;I3L2R7;I3L3Z3;I3L425;I3L1U4 | Pigment epithelium-derived factor | SERPINF1 | 18 | 18 | 57.9 | 46.312 | 27.17 | 27.14 | 27.57 | 29.79 | 29.93 | 29.83 | -2.56 | -5.89 | 0.00006 |
| P07585;P07585-4;F8VXZ8;F8VUF6;F8VWU0;F8VX58;P07585-2;P07585-3;H0YI87;F8VNV6;P07585-5;F8VNW0;H0YIH3;F8VU58;F8VSI3 | Decorin | DCN | 12 | 12 | 39.6 | 39.746 | 24.63 | 24.17 | 24.24 | 27.26 | 27.13 | 27.10 | -2.82 | -7.05 | 0.00005 |
| E9PHK0;P05452 | Tetranectin | CLEC3B | 9 | 1 | 59.4 | 17.794 | 23.82 | 24.04 | 24.50 | 27.21 | 26.88 | 26.81 | -2.85 | -7.20 | 0.00027 |
| P55083;P55083-2;K7ES70 | Microfibril-associated glycoprotein 4 | MFAP4 | 2 | 2 | 9.8 | 28.648 | 22.37 | 20.15 | 21.83 | 23.39 | 24.40 | 25.19 | -2.88 | -7.34 | 0.02753 |
| F5GXS0;P0C0L5;P0C0L4-2;P0C0L4;B4DIE5;B4E344;F5H2J9;Q9UNU2;B4DDH0;B7Z1F8;Q6U2E7;Q6U2M2;Q6U2L6;Q6U2K2;Q6U2H4 | Complement C4-B;Complement C4 beta chain;Complement C4-B alpha chain;C4a anaphylatoxin;C4b-B;C4d-B;Complement C4 gamma chain;Complement C4-A;Complement C4 beta chain;Complement C4-A alpha chain;C4a anaphylatoxin;C4b-A;C4d-A;Complement C4 gamma chain | C4B;C4A | 15 | 15 | 15.4 | 187.67 | 20.55 | 20.54 | 20.21 | 25.16 | 25.02 | 25.26 | -4.71 | -26.23 | 0.00000 |
| P07311;G3V2U7;G3V3F8;P07311-2;G3V597 | Acylphosphatase-1;Acylphosphatase | ACYP1 | 5 | 5 | 43.4 | 11.261 | 23.62 | 23.98 | 23.95 | 18.19 | 22.62 | 22.30 | 2.82 | 7.05 | 0.12017 |
| B4DS32;B4DPS6;B4DUC5;B4DM31;P55060-4;P55060-3;P55060 | Exportin-2 | CSE1L | 2 | 2 | 3.9 | 64.382 | 26.13 | 25.90 | 20.23 | 19.19 | 20.46 | 25.25 | 2.45 | 5.47 | 0.41072 |
| P42126-2;P42126;Q96DC0;H3BS70 | Enoyl-CoA delta isomerase 1, mitochondrial | ECI1;DCI | 6 | 6 | 27.4 | 30.895 | 24.16 | 24.26 | 24.33 | 18.60 | 23.46 | 23.61 | 2.36 | 5.13 | 0.22505 |
| Q15819;G3V113;H0YBP9;H0YBX6 | Ubiquitin-conjugating enzyme E2 variant 2 | UBE2V2 | 2 | 2 | 11 | 16.363 | 20.88 | 20.12 | 24.22 | 18.11 | 20.94 | 19.15 | 2.34 | 5.07 | 0.19469 |
| Q9Y5S9-2;Q9Y5S9 | RNA-binding protein 8A | RBM8A | 2 | 2 | 17.3 | 19.76 | 22.74 | 23.11 | 23.10 | 20.05 | 22.57 | 19.45 | 2.29 | 4.89 | 0.07621 |
| P30405;B2R6X6;R4GN99;Q2YDB7;H0Y548 | Peptidyl-prolyl cis-trans isomerase F, mitochondrial;Peptidyl-prolyl cis-trans isomerase | PPIF | 6 | 5 | 50.2 | 22.04 | 25.12 | 25.55 | 25.68 | 20.67 | 24.39 | 24.49 | 2.27 | 4.81 | 0.14877 |
| P55072;Q9NTC4;Q0IIN5;Q96IF9;Q9HAP0;C9JUP7;C9IZA5;Q9HAP1 | Transitional endoplasmic reticulum ATPase | VCP;DKFZp434K0126 | 5 | 5 | 10.2 | 89.321 | 21.87 | 21.06 | 21.19 | 18.24 | 18.04 | 21.06 | 2.26 | 4.79 | 0.08859 |
| P81605;P81605-2 | Dermcidin;Survival-promoting peptide;DCD-1 | DCD | 2 | 2 | 22.7 | 11.284 | 23.29 | 23.18 | 24.04 | 23.67 | 20.41 | 19.79 | 2.21 | 4.64 | 0.14734 |
| O00625 | Pirin | PIR | 3 | 3 | 9.3 | 32.113 | 22.61 | 21.57 | 21.85 | 21.46 | 17.86 | 20.31 | 2.13 | 4.37 | 0.12698 |
| F6WYE2;F6TR96;H0Y5J9;Q9H2D6-6;Q9H2D6-5;Q9H2D6-7;Q9H2D6-3;Q9H2D6-2;Q9H2D6;F6WMF4 | TRIO and F-actin-binding protein | TRIOBP | 2 | 2 | 9.4 | 28.957 | 22.27 | 22.00 | 21.72 | 20.06 | 18.50 | 21.16 | 2.09 | 4.26 | 0.05688 |
| O14933-2;O14933;E9PKW8;E9PQT7 | Ubiquitin/ISG15-conjugating enzyme E2 L6 | UBE2L6 | 2 | 2 | 29.9 | 10.086 | 21.86 | 22.26 | 21.35 | 19.53 | 21.36 | 18.48 | 2.03 | 4.10 | 0.08212 |
| B4DNR3;B4DQI4;Q96IU4;F8W9U3 | Alpha/beta hydrolase domain-containing protein 14B | ABHD14B | 5 | 5 | 31.4 | 19.796 | 24.36 | 24.46 | 24.66 | 20.07 | 24.28 | 23.07 | 2.02 | 4.06 | 0.18237 |
| B4E1K8;B4DVY1;A8MWD3;O15371;B0QYA4;B0QYA5 | Eukaryotic translation initiation factor 3 subunit D | EIF3D | 2 | 2 | 5.3 | 52.827 | 20.90 | 21.35 | 21.78 | 17.72 | 20.14 | 20.45 | 1.91 | 3.75 | 0.10167 |
| Q13185;C9JMM0;B8ZZ43;S4R2Y4 | Chromobox protein homolog 3 | CBX3 | 2 | 2 | 14.2 | 20.811 | 21.84 | 21.76 | 22.40 | 18.82 | 20.88 | 20.80 | 1.84 | 3.57 | 0.05934 |
| P62140;B4DJ75;C9J9S3;C9JP48;B7ZB67;E7ETD8;B4E163;F8WE71;C4TNW6;H0Y3Y6;B4DNE3 | Serine/threonine-protein phosphatase PP1-beta catalytic subunit;Serine/threonine-protein phosphatase | PPP1CB | 4 | 4 | 16.2 | 37.186 | 21.50 | 21.57 | 21.88 | 19.00 | 21.66 | 18.99 | 1.77 | 3.41 | 0.11935 |
| Q8WVY7 | Ubiquitin-like domain-containing CTD phosphatase 1 | UBLCP1 | 5 | 5 | 13.5 | 36.804 | 22.07 | 21.99 | 22.02 | 18.72 | 21.06 | 21.46 | 1.61 | 3.06 | 0.13207 |
| B4DZI8;P35606;B4E2C9;D6R997;D6RBZ7;D6RCL6;D6RBG7;D6RBT6 | Coatomer subunit beta | COPB2 | 5 | 5 | 7.5 | 99.045 | 23.08 | 23.61 | 23.14 | 20.31 | 22.44 | 22.30 | 1.59 | 3.02 | 0.08800 |
| A0A024R172;Q14914-2;Q14914;Q5JVP3;F2Z3J9;Q5JVP2;F6XGT7 | Prostaglandin reductase 1 | PTGR1 | 10 | 10 | 39.2 | 35.885 | 25.05 | 25.37 | 25.42 | 22.71 | 24.67 | 23.84 | 1.54 | 2.90 | 0.05713 |
| E9PHS0;Q6FHH6;O43813;B2R602;A0A024R3Z5;F8WDS9;B4DGM7 | LanC-like protein 1 | LANCL1 | 7 | 7 | 48.5 | 22.042 | 24.47 | 23.85 | 23.83 | 21.17 | 23.08 | 23.31 | 1.53 | 2.89 | 0.09738 |
| P48506;B4E2I4;E1CEI4;H0Y9I7;D6RGF8;H0YAB6;D6R959 | Glutamate--cysteine ligase catalytic subunit | GCLC | 4 | 4 | 7.8 | 72.765 | 21.63 | 21.52 | 21.66 | 20.81 | 21.59 | 17.84 | 1.52 | 2.87 | 0.25383 |
| Q4ZG57;Q14566;B4DRF6 | DNA replication licensing factor MCM6 | MCM6 | 2 | 2 | 3.3 | 88.945 | 21.28 | 21.21 | 21.61 | 19.26 | 21.35 | 18.95 | 1.51 | 2.85 | 0.11885 |
| P19367-4;B4DG62;P19367-2;P19367;A8K7J7;P19367-3;Q59FD4;E7ENR4;B3KXY9;B3KRA9;B1AR63;B4E0J9;Q2TB90;B3KT70;A8K4Q9 | Hexokinase-1 | HK1 | 4 | 4 | 6.2 | 101.08 | 21.40 | 21.57 | 21.67 | 17.91 | 21.43 | 20.79 | 1.51 | 2.84 | 0.23694 |
| E9PC52;Q16576;Q16576-2;A8K6A2;Q5JNZ6;C9J7L0;Q5JP01 | Histone-binding protein RBBP7 | RBBP7 | 3 | 3 | 16.1 | 46.938 | 21.42 | 21.78 | 21.62 | 18.64 | 20.71 | 21.00 | 1.49 | 2.80 | 0.11858 |
| P07305;P07305-2 | Histone H1.0;Histone H1.0, N-terminally processed | H1F0 | 2 | 2 | 11.9 | 20.863 | 22.44 | 22.54 | 21.64 | 20.31 | 21.90 | 20.05 | 1.46 | 2.74 | 0.08686 |
| Q9UBE0;B4DY66;M0QX65;M0QZS6;Q9UBE0-2;Q9UBE0-3;B3KNJ4;M0QYM8;M0R054 | SUMO-activating enzyme subunit 1;SUMO-activating enzyme subunit 1, N-terminally processed | SAE1 | 3 | 3 | 11.8 | 38.449 | 21.28 | 21.34 | 21.18 | 20.78 | 20.78 | 17.90 | 1.45 | 2.72 | 0.20643 |
| P25398 | 40S ribosomal protein S12 | RPS12 | 2 | 2 | 14.4 | 14.515 | 21.36 | 20.82 | 20.21 | 20.01 | 18.18 | 20.07 | 1.38 | 2.60 | 0.12224 |
| Q9Y547;A6NIR2;X6R7Y7 | Heat shock protein beta-11 | HSPB11 | 5 | 5 | 55.6 | 16.297 | 26.12 | 25.83 | 26.01 | 23.50 | 25.06 | 25.30 | 1.36 | 2.58 | 0.07603 |
| Q01469;I6L8B7;A8MUU1 | Fatty acid-binding protein, epidermal | FABP5 | 3 | 3 | 41.5 | 15.164 | 21.69 | 21.73 | 23.44 | 21.32 | 20.75 | 20.72 | 1.36 | 2.56 | 0.08994 |
| O00154-2;B4DUX0;K7EKP8;O00154-3;O00154-6;O00154-4;O00154-5;O00154-7;O00154;F5GWE2;B7Z957 | Cytosolic acyl coenzyme A thioester hydrolase | ACOT7 | 2 | 2 | 11 | 27.041 | 20.97 | 20.98 | 20.92 | 20.28 | 20.97 | 17.62 | 1.33 | 2.52 | 0.26216 |
| B3KRR4;Q32Q83;Q6P392;Q8WUA2 | Peptidyl-prolyl cis-trans isomerase-like 4 | PPIL4 | 2 | 2 | 11.1 | 42.047 | 20.16 | 20.85 | 21.30 | 21.16 | 17.64 | 19.62 | 1.30 | 2.46 | 0.29178 |
| P26599;P26599-2;P26599-3;A6NLN1;K7EK45 | Polypyrimidine tract-binding protein 1 | PTBP1 | 3 | 3 | 8.1 | 57.221 | 22.81 | 22.18 | 20.19 | 20.74 | 20.63 | 19.92 | 1.30 | 2.46 | 0.19246 |
| P49441;B5BU62;B3KSH8;C9J128;C9J2Z6;X5DP77;C9J173;B8ZZF6;E7ET59;E7EUX4;E7ENF2;X5DRF6 | Inositol polyphosphate 1-phosphatase | INPP1 | 3 | 3 | 10.5 | 43.998 | 22.08 | 20.92 | 21.00 | 20.90 | 17.44 | 21.78 | 1.29 | 2.45 | 0.40151 |
| E5RK69;P08133-2;A6NN80;P08133;A8K3Q7;Q6ZP35;E7EMC6;B7Z582;E5RIU8;E5RJR0;E5RJF5;E5RI05;E5RK63;E5RFF0 | Annexin;Annexin A6 | ANXA6 | 3 | 3 | 10.2 | 51.776 | 20.91 | 20.81 | 19.94 | 20.08 | 19.76 | 18.04 | 1.26 | 2.39 | 0.14867 |
| A8K067;B4DI19;Q53HG6;P49589-2;A8MVQ3;B4DKY1;P49589;B4DPV7;P49589-3 | Cysteine--tRNA ligase, cytoplasmic | CARS | 2 | 2 | 2.9 | 73.423 | 22.02 | 21.30 | 19.75 | 19.75 | 19.97 | 19.57 | 1.26 | 2.39 | 0.13824 |
| P20962;F5H7R9;O15256;A2VCM6;F5GXR3 | Parathymosin | PTMS | 2 | 2 | 22.5 | 11.53 | 22.60 | 23.12 | 22.42 | 22.78 | 21.45 | 20.16 | 1.25 | 2.38 | 0.18619 |
| P13500 | C-C motif chemokine 2 | CCL2 | 2 | 2 | 17.2 | 11.025 | 24.34 | 25.11 | 25.79 | 24.38 | 23.12 | 23.99 | 1.25 | 2.38 | 0.08964 |
| B4DL61;Q6MZV4;B4E1Z8;Q8IWE2;B3KSS5 | Protein NOXP20 | DKFZp686F20250;FAM114A1 | 3 | 3 | 16.6 | 39.913 | 22.38 | 22.06 | 21.79 | 19.84 | 21.23 | 21.46 | 1.23 | 2.35 | 0.08231 |
| Q99757;B4DX69;M0QXH0;F8WDN2 | Thioredoxin, mitochondrial | TXN2 | 3 | 3 | 26.5 | 18.383 | 22.23 | 22.02 | 23.05 | 20.61 | 21.91 | 21.13 | 1.22 | 2.33 | 0.06902 |
| Q96FN9;F8W1H4 | Probable D-tyrosyl-tRNA(Tyr) deacylase 2 | DTD2 | 3 | 3 | 31 | 18.66 | 23.02 | 23.04 | 22.55 | 19.96 | 23.04 | 22.03 | 1.19 | 2.29 | 0.26445 |
| F8VR84;Q8WYI2;F8VQQ3;Q96CT6;Q9HB07;Q86UA3 | UPF0160 protein MYG1, mitochondrial | C12orf10;MST024 | 2 | 2 | 12.2 | 24.058 | 21.19 | 21.16 | 20.66 | 18.95 | 20.22 | 20.28 | 1.19 | 2.28 | 0.06305 |
| P30711;C9JA47;G3XAK0;H7BZG9;G5E9I8;G5E9P7;F8WCF3;F6UD80;Q4W251;G5E9K8;Q6IC69;Q4W252 | Glutathione S-transferase theta-1 | GSTT1 | 6 | 6 | 30 | 27.335 | 22.64 | 22.56 | 22.61 | 20.60 | 22.13 | 21.54 | 1.18 | 2.26 | 0.05791 |
| P25311;A0JLQ0;C9JEV0 | Zinc-alpha-2-glycoprotein | AZGP1 | 3 | 3 | 11.7 | 34.258 | 18.02 | 20.80 | 21.55 | 19.52 | 19.17 | 18.16 | 1.17 | 2.26 | 0.36475 |
| P01833 | Polymeric immunoglobulin receptor;Secretory component | PIGR | 2 | 2 | 5.1 | 83.283 | 19.70 | 18.11 | 21.18 | 18.04 | 19.81 | 17.64 | 1.17 | 2.25 | 0.35199 |
| Q9Y3F4;B4DNJ6;B0AZV0 | Serine-threonine kinase receptor-associated protein | STRAP | 3 | 3 | 10.6 | 38.438 | 21.73 | 21.38 | 21.08 | 19.32 | 20.69 | 20.71 | 1.16 | 2.23 | 0.08131 |
| P02792;B1Q3B3;Q6S4P3;B1Q3B4;B1Q387;Q4FCH6 | Ferritin light chain;Ferritin | FTL;FTL variant | 7 | 7 | 46.9 | 20.019 | 26.55 | 26.99 | 26.64 | 24.75 | 26.02 | 25.99 | 1.14 | 2.21 | 0.05961 |
| P54577 | Tyrosine--tRNA ligase, cytoplasmic;Tyrosine--tRNA ligase, cytoplasmic, N-terminally processed | YARS | 3 | 3 | 9.5 | 59.143 | 20.93 | 20.73 | 21.00 | 20.13 | 18.39 | 20.73 | 1.14 | 2.20 | 0.18279 |
| O43815-2;Q3B874;O43815 | Striatin | STRN | 3 | 3 | 8.1 | 80.76 | 20.64 | 20.85 | 20.74 | 18.72 | 20.21 | 19.92 | 1.13 | 2.18 | 0.06984 |
| O75396;I1VE16 | Vesicle-trafficking protein SEC22b | SEC22B | 2 | 2 | 11.2 | 24.593 | 21.42 | 21.22 | 21.05 | 20.61 | 19.01 | 20.73 | 1.11 | 2.16 | 0.12047 |
| P11586;A0A024R652;F5H2F4;B7Z809 | C-1-tetrahydrofolate synthase, cytoplasmic;Methylenetetrahydrofolate dehydrogenase;Methenyltetrahydrofolate cyclohydrolase;Formyltetrahydrofolate synthetase;C-1-tetrahydrofolate synthase, cytoplasmic, N-terminally processed | MTHFD1 | 2 | 2 | 2.7 | 101.56 | 20.35 | 20.27 | 22.38 | 20.12 | 19.70 | 19.88 | 1.10 | 2.14 | 0.19266 |
| F5GXQ0;B7Z953;B7Z9G5;Q5VW32;B7Z1V1;Q5VW33 | BRO1 domain-containing protein BROX | BROX;C1orf58 | 4 | 4 | 23.4 | 38.212 | 22.25 | 22.55 | 21.04 | 20.78 | 21.34 | 20.48 | 1.08 | 2.11 | 0.10971 |
| Q8N440;Q7Z3B1 | Neuronal growth regulator 1 | NEGR1 | 2 | 2 | 15.5 | 24.733 | 21.69 | 21.46 | 21.81 | 18.25 | 21.81 | 21.66 | 1.08 | 2.11 | 0.40807 |
| E9PKT9;O96033 | Molybdopterin synthase sulfur carrier subunit | MOCS2 | 2 | 2 | 22.9 | 9.2145 | 21.39 | 21.56 | 22.09 | 20.98 | 19.84 | 21.03 | 1.06 | 2.08 | 0.07441 |
| D3DPQ2;Q8IWT0;A8K0B5;Q8IWT0-2;H7C3R6 | Protein archease | ZBTB8OS | 2 | 2 | 20 | 12.777 | 21.12 | 22.00 | 22.21 | 20.38 | 20.98 | 20.83 | 1.05 | 2.07 | 0.05058 |
| O00151 | PDZ and LIM domain protein 1 | PDLIM1 | 2 | 2 | 7.6 | 36.071 | 21.71 | 21.40 | 21.93 | 21.23 | 19.93 | 20.76 | 1.04 | 2.06 | 0.06293 |
| J3KS05;P83916;C9JWS9;K7ELA4;Q9Y654;B5MD17 | Chromobox protein homolog 1 | CBX1 | 2 | 2 | 17.3 | 20.03 | 20.91 | 19.62 | 21.17 | 18.68 | 20.09 | 19.83 | 1.03 | 2.05 | 0.18505 |
| F2Z2F3;Q8WV80;Q13126-7;Q05DJ2;J3QSB7;Q13126-4;Q13126;Q13126-6;B4DUC8;Q13126-5;Q13126-3;Q13126-2;F8WES2;Q6FHT1;Q6FHP1 | S-methyl-5-thioadenosine phosphorylase;Purine nucleoside phosphorylase | MTAP | 2 | 2 | 24.5 | 15.645 | 20.35 | 20.15 | 20.68 | 17.93 | 20.45 | 19.72 | 1.02 | 2.03 | 0.25245 |
| Q9BT73 | Proteasome assembly chaperone 3 | PSMG3 | 2 | 2 | 23 | 13.104 | 20.53 | 20.56 | 21.58 | 19.93 | 19.86 | 19.89 | 1.00 | 2.00 | 0.04565 |
| Q53GD8;Q00688 | Peptidyl-prolyl cis-trans isomerase;Peptidyl-prolyl cis-trans isomerase FKBP3 | FKBP3 | 9 | 9 | 45.1 | 25.204 | 24.71 | 24.30 | 24.52 | 23.28 | 23.58 | 23.68 | 1.00 | 2.00 | 0.00410 |
| Q8WZ82 | Ovarian cancer-associated gene 2 protein | OVCA2 | 3 | 3 | 18.1 | 24.418 | 21.15 | 20.84 | 20.89 | 20.02 | 19.88 | 19.99 | 1.00 | 1.99 | 0.00065 |
| Q9Y3C6 | Peptidyl-prolyl cis-trans isomerase-like 1 | PPIL1 | 4 | 4 | 27.1 | 18.237 | 24.80 | 25.90 | 23.69 | 24.46 | 23.90 | 23.06 | 0.99 | 1.99 | 0.25942 |
| P53621;P53621-2 | Coatomer subunit alpha;Xenin;Proxenin | COPA | 13 | 13 | 10.3 | 138.34 | 26.24 | 26.25 | 26.37 | 25.08 | 25.45 | 25.35 | 0.99 | 1.99 | 0.00114 |
| P09417;P09417-2;B7Z415;D6RGG7;H0Y8F7;D6RHJ7 | Dihydropteridine reductase | QDPR | 8 | 8 | 45.1 | 25.789 | 25.84 | 25.80 | 26.12 | 24.89 | 24.92 | 24.97 | 0.99 | 1.99 | 0.00064 |
| Q96C86;Q53G42 | m7GpppX diphosphatase | DCPS | 8 | 8 | 35.6 | 38.608 | 23.84 | 23.53 | 23.45 | 22.72 | 22.57 | 22.55 | 0.99 | 1.99 | 0.00149 |
| P16152;P16152-2;E9PQ63;A8MTM1 | Carbonyl reductase [NADPH] 1 | CBR1 | 15 | 13 | 68.2 | 30.375 | 27.27 | 27.17 | 27.33 | 26.05 | 26.33 | 26.42 | 0.99 | 1.99 | 0.00115 |
| P63241;I3L397;P63241-2;I3L504;Q6IS14;F8WCJ1;C9J7B5;C9J4W5;Q9GZV4 | Eukaryotic translation initiation factor 5A-1;Eukaryotic translation initiation factor 5A-1-like | EIF5A;EIF5AL1 | 16 | 16 | 88.3 | 16.832 | 28.21 | 27.99 | 28.08 | 27.06 | 26.96 | 27.29 | 0.99 | 1.98 | 0.00111 |
| E5RIW3;O75347;E5RJD8;E5RHG6;B4DT30;E5RIX8 | Tubulin-specific chaperone A | TBCA | 7 | 7 | 70.2 | 10.08 | 26.33 | 26.31 | 26.53 | 25.72 | 25.19 | 25.31 | 0.99 | 1.98 | 0.00499 |
| Q8NFH4;B4DKV8;F8VTY2 | Nucleoporin Nup37 | NUP37 | 5 | 5 | 20.6 | 36.707 | 22.39 | 22.62 | 23.12 | 21.70 | 21.79 | 21.70 | 0.98 | 1.97 | 0.01127 |
| H3BRV9;P61970;B4DEQ2 | Nuclear transport factor 2 | NUTF2 | 4 | 4 | 71 | 12.159 | 25.20 | 25.66 | 26.01 | 24.79 | 24.73 | 24.44 | 0.97 | 1.97 | 0.01924 |
| B3KY56;Q9BSJ8;B3KMV5;Q9BSJ8-2 | Extended synaptotagmin-1 | ESYT1 | 9 | 9 | 9 | 117.39 | 24.85 | 24.69 | 24.49 | 23.43 | 23.96 | 23.72 | 0.97 | 1.97 | 0.00622 |
| Q17RE6;F5H1L4;E7ENA2;Q9NNW7-2;F5H2V0;D3YTF9;Q9NNW7;D3YTF8;Q9NNW7-3;B3KUQ5;E7EWK1;A4FTY4;Q9NNW7-4 | Thioredoxin reductase 2, mitochondrial | TXNRD2 | 2 | 2 | 3.3 | 53.394 | 22.31 | 22.13 | 22.05 | 19.28 | 22.10 | 22.19 | 0.97 | 1.96 | 0.36760 |
| P00533-2;P00533-4;Q68GS6;P00533-3;E7BSV0;Q2TTR7;P00533;A9CB80;A8K2T7;Q504U8;E9PFD7;B7Z2I3;F2YGG7;C9JYS6;Q59FL8 | Epidermal growth factor receptor | EGFR | 3 | 3 | 9.1 | 44.664 | 20.41 | 20.33 | 20.70 | 19.86 | 18.21 | 20.46 | 0.97 | 1.96 | 0.22758 |
| Q5TCU6;Q9Y490;Q9Y4G6 | Talin-1 | TLN1 | 16 | 16 | 6.1 | 258.08 | 25.50 | 25.22 | 25.25 | 24.27 | 24.33 | 24.46 | 0.97 | 1.96 | 0.00079 |
| Q96I65;Q04637-6;Q04637-7;Q04637-5;E7EX73;Q04637-4;B4DSI9;E9PGM1;Q04637-3;E7EUU4;B4DGF1;Q04637;Q04637-8;Q04637-9;B2RU10;B2RU06;Q4LE58;H7C0V6 | Eukaryotic translation initiation factor 4 gamma 1 | EIF4G1;EIF4G1 variant protein | 10 | 10 | 23.3 | 72.2 | 26.26 | 26.00 | 25.45 | 24.98 | 24.94 | 24.89 | 0.97 | 1.96 | 0.01560 |
| Q07021;A8K651;I3L3Q7;I3L3B0 | Complement component 1 Q subcomponent-binding protein, mitochondrial | C1QBP | 8 | 8 | 34 | 31.362 | 24.80 | 24.93 | 25.40 | 24.06 | 24.30 | 23.87 | 0.97 | 1.96 | 0.01191 |
| P50897-2;E9PIA8;P50897;Q5T0S4;B4DWU3;E9PMG2;E9PSE5 | Palmitoyl-protein thioesterase 1 | PPT1 | 3 | 3 | 20.2 | 23.093 | 22.10 | 21.82 | 22.17 | 21.26 | 21.10 | 20.83 | 0.97 | 1.95 | 0.00443 |
| P17612;A8K8B9;P17612-2;B7ZA00;P22694-10;P22694-4;P22694-3;P22694;P22694-5;P22694-7;P22694-6;P22694-9;B4E2L0;P22694-2;Q15136;K7ERP6;K7ENJ5;B1APF6;B1APF7;B1APG1;B1APG0;B1APG2;B1APF8;B7Z708;B1APF9;P22694-8;B1APG3 | cAMP-dependent protein kinase catalytic subunit alpha;cAMP-dependent protein kinase catalytic subunit beta | PRKACA;PRKACB | 9 | 9 | 25.9 | 40.589 | 23.86 | 23.36 | 23.61 | 22.61 | 22.51 | 22.81 | 0.97 | 1.95 | 0.00458 |
| M0R389;Q15102 | Platelet-activating factor acetylhydrolase IB subunit gamma | PAFAH1B3 | 2 | 2 | 11 | 18.356 | 21.38 | 20.47 | 21.28 | 19.62 | 19.89 | 20.72 | 0.96 | 1.95 | 0.09343 |
| Q9NRX4;Q6FIE5;Q9NRX4-2;Q9P019 | 14 kDa phosphohistidine phosphatase | PHPT1;PHP14 | 6 | 6 | 63.2 | 13.832 | 25.83 | 25.89 | 26.22 | 24.91 | 25.29 | 24.85 | 0.96 | 1.95 | 0.00622 |
| O14579;M0QXB4;O14579-2;Q53HJ6;O14579-3;Q7Z4Z1;M0R061 | Coatomer subunit epsilon | COPE | 8 | 8 | 47.4 | 34.482 | 24.31 | 24.21 | 24.45 | 23.02 | 23.11 | 23.96 | 0.96 | 1.95 | 0.03588 |
| O75223;O75223-3;M0QZK8;B8ZZK2;O75223-2;H7BZK5;B3KMN7;O75223-4 | Gamma-glutamylcyclotransferase | GGCT | 7 | 7 | 43.1 | 21.007 | 25.08 | 25.25 | 25.34 | 24.41 | 24.30 | 24.08 | 0.96 | 1.95 | 0.00142 |
| B8ZZQ6;V9HVW6;Q15202;Q8TBK9;Q86YS2;Q15204;Q15200;P06454-2;P06454;B8ZZA1;B8ZZW7;H7C2N1;Q15203;Q7KZ52;Q9UMZ1;Q9NYD3;Q15254;V9HVW7;Q7Z4R6 | Prothymosin alpha;Prothymosin alpha, N-terminally processed;Thymosin alpha-1 | PTMA | 2 | 2 | 22.4 | 11.758 | 21.31 | 22.17 | 21.55 | 20.03 | 21.67 | 20.44 | 0.96 | 1.95 | 0.15833 |
| A2VCR0;B4DM33;Q15691 | Microtubule-associated protein RP/EB family member 1 | MAPRE1 | 4 | 4 | 33.5 | 19.408 | 23.00 | 22.52 | 22.20 | 21.49 | 21.69 | 21.65 | 0.96 | 1.95 | 0.01643 |
| Q96GX9;J3KN82;S4R3D6;Q96GX9-3;B4DY17 | Methylthioribulose-1-phosphate dehydratase | APIP | 2 | 2 | 8.3 | 27.125 | 19.65 | 19.98 | 20.71 | 19.31 | 19.08 | 19.08 | 0.96 | 1.94 | 0.04094 |
| P29373;Q5SYZ4;B5MCB5;P29762 | Cellular retinoic acid-binding protein 2 | CRABP2 | 12 | 12 | 75.4 | 15.693 | 28.32 | 28.24 | 28.02 | 27.43 | 27.17 | 27.10 | 0.96 | 1.94 | 0.00204 |
| Q9GZP4-2;Q9GZP4;X6R8S9;B4DKP7;X6RHB9 | PITH domain-containing protein 1 | PITHD1 | 3 | 3 | 12.9 | 24.121 | 21.66 | 21.23 | 21.88 | 20.79 | 20.59 | 20.52 | 0.96 | 1.94 | 0.00978 |
| A8K6Y1;Q6PIN5;Q9UQ80;Q05D08;F8VR77;H0YIN7;F8W0A3;F8VZ69 | Proliferation-associated protein 2G4 | PA2G4 | 23 | 23 | 67.3 | 40.911 | 28.37 | 28.50 | 28.70 | 27.58 | 27.48 | 27.63 | 0.96 | 1.94 | 0.00080 |
| P60983;Q9BS35;G3V4P8;Q59G69;M0QYG8;M0R1D2;M0R0C1;M0QYJ8;Q8TDZ6;O60234 | Glia maturation factor beta | GMFB | 5 | 5 | 35.2 | 16.713 | 24.66 | 24.40 | 24.36 | 23.38 | 23.41 | 23.78 | 0.95 | 1.93 | 0.00396 |
| O95989;S4R435 | Diphosphoinositol polyphosphate phosphohydrolase 1 | NUDT3 | 2 | 2 | 18 | 19.471 | 21.11 | 21.66 | 21.91 | 20.79 | 20.57 | 20.48 | 0.95 | 1.93 | 0.01983 |
| Q86WA6-2;Q86WA6 | Valacyclovir hydrolase | BPHL | 3 | 3 | 12.4 | 31.106 | 21.06 | 21.90 | 21.83 | 21.12 | 20.66 | 20.16 | 0.95 | 1.93 | 0.06915 |
| Q9NWV4;D3DQ38;B1ARP7 | UPF0587 protein C1orf123 | C1orf123 | 7 | 7 | 59.4 | 18.048 | 26.03 | 26.23 | 26.43 | 24.59 | 25.64 | 25.62 | 0.95 | 1.93 | 0.06087 |
| H3BQF7;H3BMU1;H3BUI0;J3KR23;P53990-2;P53990-3;P53990;A8K5S3;P53990-4;P53990-5;F5GXM3;B4DHF3;B4DM80;B4DLP1 | IST1 homolog | IST1 | 3 | 3 | 22.3 | 19.013 | 22.73 | 22.76 | 21.84 | 21.80 | 21.30 | 21.41 | 0.94 | 1.92 | 0.04962 |
| E9PGT1;Q15631;Q53GR3;B3KRM8;H7C1D4;B7Z5D9;Q15631-2 | Translin | TSN | 10 | 10 | 53.8 | 25.572 | 26.08 | 26.11 | 25.95 | 24.84 | 25.13 | 25.34 | 0.94 | 1.92 | 0.00338 |
| Q6P1N4;P46940;A4QPB0 | Ras GTPase-activating-like protein IQGAP1 | IQGAP1 | 4 | 4 | 6.4 | 107.54 | 21.93 | 21.01 | 20.88 | 20.29 | 20.46 | 20.25 | 0.94 | 1.92 | 0.04948 |
| F8W717;B7Z650;O00423;O00423-3;B3KXA3;H0YJK4;H0YJY3;G3V500;G3V3N9 | Echinoderm microtubule-associated protein-like 1 | EML1 | 2 | 2 | 4.9 | 88.716 | 21.09 | 21.08 | 21.65 | 19.46 | 20.76 | 20.79 | 0.94 | 1.92 | 0.12080 |
| F5GX07;H0YG83;H0YGR4;H0YG54;Q9Y3B8-2;Q9Y3B8-3;Q9Y3B8;F5GYG5;H0YH58 | Oligoribonuclease, mitochondrial | REXO2 | 7 | 7 | 50.3 | 17.28 | 23.98 | 23.55 | 23.83 | 23.41 | 22.64 | 22.50 | 0.94 | 1.92 | 0.03824 |
| D6RAN1;Q9NR12-3;D6RF83;Q9NR12-5;H7BYK4;Q9NR12-6;Q9NR12-4;D6RH06;Q9NR12-2;Q9NR12 | PDZ and LIM domain protein 7 | PDLIM7 | 2 | 2 | 24.4 | 9.4587 | 21.33 | 21.98 | 22.02 | 20.78 | 20.88 | 20.87 | 0.94 | 1.91 | 0.01447 |
| Q9UMY4-2;Q9UMY4;Q9UMY4-3;O60493-3;O60493 | Sorting nexin-12 | SNX12 | 6 | 6 | 40.1 | 18.884 | 23.40 | 23.51 | 23.41 | 22.35 | 22.31 | 22.85 | 0.94 | 1.91 | 0.00618 |
| P15121;Q59EL5;E9PCX2;E9PEF9;O15289 | Aldose reductase | AKR1B1 | 25 | 24 | 83.9 | 35.853 | 31.22 | 31.23 | 31.36 | 29.95 | 30.47 | 30.59 | 0.93 | 1.91 | 0.00987 |
| P23528;E9PK25;G3V1A4;E9PP50;E9PQB7;B4E112;E9PS23;E9PLJ3 | Cofilin-1 | CFL1 | 19 | 14 | 74.7 | 18.502 | 30.23 | 30.08 | 30.51 | 29.33 | 29.46 | 29.26 | 0.93 | 1.90 | 0.00258 |
| O95861-4;O95861;O95861-2;A6NF51;O95861-3;B7Z9J7;F8VZG4;F8W1J0;F8VVW8;F8VRY7 | 3(2),5-bisphosphate nucleotidase 1 | BPNT1 | 11 | 11 | 43 | 29.188 | 24.98 | 25.16 | 25.10 | 24.15 | 24.16 | 24.16 | 0.92 | 1.90 | 0.00006 |
| P62857 | 40S ribosomal protein S28 | RPS28 | 3 | 3 | 36.2 | 7.8409 | 23.32 | 23.80 | 23.87 | 22.62 | 22.68 | 22.91 | 0.92 | 1.89 | 0.00889 |
| P26447;Q5Q9Z3 | Protein S100-A4 | S100A4 | 5 | 5 | 44.6 | 11.728 | 27.40 | 27.57 | 27.88 | 27.27 | 26.29 | 26.53 | 0.92 | 1.89 | 0.04705 |
| G3V5E4;G3V4W4;Q96EK6 | Glucosamine 6-phosphate N-acetyltransferase | GNPNAT1 | 2 | 2 | 19.5 | 12.976 | 21.25 | 21.64 | 20.94 | 21.41 | 20.06 | 19.60 | 0.92 | 1.89 | 0.18716 |
| B3KW52;H3BRL3;O14562 | Ubiquitin domain-containing protein UBFD1 | UBFD1 | 2 | 2 | 14.5 | 21.146 | 20.98 | 21.41 | 20.95 | 19.69 | 20.40 | 20.50 | 0.92 | 1.89 | 0.03510 |
| F8W1N5;F8VZJ2;F8VNW4;F8W0W4;H0YHX9;Q13765;E9PAV3-2;E9PAV3;F8VZ58;A8K6N6;B4DDI8;Q9BZK3 | Nascent polypeptide-associated complex subunit alpha;Putative nascent polypeptide-associated complex subunit alpha-like protein | NACA;NACAP1 | 2 | 2 | 38 | 7.813 | 20.44 | 20.61 | 21.21 | 19.41 | 19.89 | 20.21 | 0.92 | 1.89 | 0.04969 |
| H7BYW1;Q9NRW1;Q6FGX3;Q6AZ91;Q53ET8;P20340-2;P20340;C9JU14;J3KR73;B7Z5Z9;P20340-4;B7Z337;H0YGL6;C9JB90;F5H3K7;C9J0I2;F5GX61;P20340-3;Q14964 | Ras-related protein Rab-6B;Ras-related protein Rab-6A | RAB6A;RAB6B | 3 | 3 | 17.2 | 19.745 | 22.35 | 22.47 | 22.31 | 21.43 | 21.11 | 21.83 | 0.92 | 1.89 | 0.01248 |
| Q86VP6;A8K8U1;Q86VP6-2;B3KMG3;Q86VP6-3;H0YH27;B3KM30;F5H6I6;F8WBB8;O75155-2;O75155 | Cullin-associated NEDD8-dissociated protein 1 | CAND1 | 14 | 14 | 14.8 | 136.37 | 23.85 | 23.75 | 23.46 | 22.73 | 22.76 | 22.83 | 0.91 | 1.88 | 0.00164 |
| Q567R6;Q04837;E7EUY5;B7Z268;C9K0U8 | Single-stranded DNA-binding protein;Single-stranded DNA-binding protein, mitochondrial | SSBP1 | 6 | 6 | 47.3 | 17.359 | 25.57 | 25.15 | 25.36 | 24.51 | 24.30 | 24.54 | 0.91 | 1.88 | 0.00315 |
| P84077;P61204;F5H423;B4DEB9;F5H0C7;C9J1Z8;B4DJC8;B4DN70;P84085;B4DLJ3;C9JPM4;B7ZB63;B4E190;P18085;C9JAK5;F5H1V1;F5H6T5;F8WDB3;H0YGG7 | ADP-ribosylation factor 1;ADP-ribosylation factor 3;ADP-ribosylation factor 5;ADP-ribosylation factor 4 | ARF1;ARF3;ARF5;ARF4 | 8 | 8 | 58.6 | 20.697 | 23.92 | 24.01 | 24.01 | 22.89 | 23.31 | 23.01 | 0.91 | 1.88 | 0.00210 |
| B7ZW30;F5H715;B7Z7D5;Q96BJ3;Q96BJ3-2 | Axin interactor, dorsalization-associated protein | AIDA | 2 | 2 | 10.1 | 29.149 | 20.79 | 20.96 | 21.01 | 19.25 | 20.67 | 20.11 | 0.91 | 1.87 | 0.09663 |
| Q9UBT2;B3KMZ6;B2RDF5;K7EPL2;U3KQ55;K7ES38;B3KWB9;B3KNA3 | SUMO-activating enzyme subunit 2 | UBA2 | 4 | 4 | 7.2 | 71.223 | 23.02 | 22.74 | 22.82 | 21.87 | 22.15 | 21.85 | 0.90 | 1.87 | 0.00216 |
| Q7Z7M4;P04179;P04179-4;Q7Z7M6;Q4ZJI1;Q7Z7M7;P04179-2;F5H4R2;F5GYZ5;Q7Z7M5;F5H3C5;P04179-3;G8JLJ2;Q6LE88;Q96AM7;G5E9P6;F5GXZ9 | Superoxide dismutase;Superoxide dismutase [Mn], mitochondrial | SOD2 | 16 | 12 | 88.7 | 23.672 | 30.67 | 30.61 | 30.57 | 29.36 | 30.04 | 29.74 | 0.90 | 1.87 | 0.01062 |
| Q8WW59;B4DUC9 | SPRY domain-containing protein 4 | SPRYD4 | 7 | 7 | 41.1 | 23.128 | 23.78 | 23.60 | 23.36 | 22.92 | 22.63 | 22.50 | 0.90 | 1.86 | 0.00686 |
| O15143;C9J4Z7;C9JEY1;C9JQM8;C9JBJ7;C9K057;C9JTT6;C9JM51;F8WEB3 | Actin-related protein 2/3 complex subunit 1B | ARPC1B | 16 | 15 | 47.6 | 40.949 | 26.69 | 26.78 | 26.92 | 26.05 | 25.98 | 25.68 | 0.89 | 1.86 | 0.00227 |
| A6NDG6;H3BV17 | Phosphoglycolate phosphatase | PGP | 8 | 8 | 34.3 | 34.006 | 24.82 | 25.07 | 25.19 | 24.34 | 24.12 | 23.95 | 0.89 | 1.85 | 0.00479 |
| B4DUP0;P26641;B4DTG2 | Elongation factor 1-gamma | EEF1G | 6 | 6 | 34 | 24.104 | 25.85 | 25.85 | 26.29 | 25.18 | 25.01 | 25.14 | 0.89 | 1.85 | 0.00475 |
| B3KM86;D3DVI5;Q8N5A0;O60841 | Eukaryotic translation initiation factor 5B | EIF5B | 5 | 5 | 15.3 | 71.492 | 21.96 | 22.38 | 22.11 | 20.66 | 21.58 | 21.54 | 0.89 | 1.85 | 0.05201 |
| P10599;P10599-2 | Thioredoxin | TXN | 9 | 9 | 65.7 | 11.737 | 28.45 | 28.34 | 28.56 | 27.70 | 27.35 | 27.64 | 0.89 | 1.85 | 0.00202 |
| P22392-2;Q32Q12;P22392;J3KPD9;O60361;F6XY72 | Nucleoside diphosphate kinase B;Nucleoside diphosphate kinase | NME2;NME1-NME2 | 17 | 8 | 82.8 | 30.137 | 28.61 | 28.55 | 28.53 | 27.48 | 27.52 | 28.02 | 0.89 | 1.85 | 0.00729 |
| Q9UIJ7;Q7Z4Y4;B3KWD9;Q9UIJ7-2;Q9UIJ7-3;Q7Z531 | GTP:AMP phosphotransferase AK3, mitochondrial | AK3 | 5 | 5 | 26.4 | 25.565 | 22.92 | 22.36 | 23.04 | 22.07 | 21.78 | 21.80 | 0.89 | 1.85 | 0.01848 |
| P22626;I6L957;P22626-2;A0A024RA28 | Heterogeneous nuclear ribonucleoproteins A2/B1 | HNRNPA2B1 | 10 | 10 | 26.3 | 37.429 | 25.71 | 25.57 | 25.77 | 24.94 | 24.64 | 24.83 | 0.88 | 1.84 | 0.00112 |
| H3BR27;P38159-3;H3BT71;P38159;H3BNC1;H0Y6E7;P38159-2;Q2VIN3;Q96E39;O75526;A8K8A6;B4E352;Q8N7X1 | RNA-binding motif protein, X chromosome;RNA-binding motif protein, X chromosome, N-terminally processed;RNA binding motif protein, X-linked-like-1;RNA-binding motif protein, X-linked-like-2 | RBMX;RBMXL1;RBMXL2 | 4 | 4 | 55.1 | 8.6098 | 22.15 | 21.74 | 22.50 | 21.37 | 21.25 | 21.13 | 0.88 | 1.84 | 0.01894 |
| P63244;J3KPE3;D6RAC2;H0YAF8;H0Y8W2;D6REE5;D6RHH4;H0YAM7;D6R9Z1;D6R9L0;D6RFX4;B4DVD2;D6RBD0;D6RFZ9;D6RF23;H0Y8R5;D6RAU2;E9PD14;B4E0C3;D6RGK8;H0Y9P0;D6RHJ5;D6R909;B4DWC6;D6RDI0;I3QNU9 | Guanine nucleotide-binding protein subunit beta-2-like 1 | GNB2L1 | 19 | 19 | 78.5 | 35.076 | 26.78 | 26.92 | 27.35 | 26.06 | 26.16 | 26.19 | 0.88 | 1.83 | 0.00745 |
| P51149;C9J592;C9J8S3;B4DPH9;C9J4S4;C9IZZ0;C9J4V0;C9J7D1 | Ras-related protein Rab-7a | RAB7A | 5 | 5 | 30.9 | 23.489 | 23.34 | 23.30 | 23.50 | 22.28 | 22.72 | 22.51 | 0.87 | 1.83 | 0.00347 |
| V9HW62;Q04760-2;Q04760 | Lactoylglutathione lyase | GLO1 | 14 | 14 | 71.7 | 20.719 | 27.38 | 27.19 | 27.35 | 26.44 | 26.51 | 26.35 | 0.87 | 1.83 | 0.00026 |
| P24666-3;P24666-2;P24666;Q59EH3;F2Z2Q9;O75702;G5E9R5;D3YTI2;P24666-4 | Low molecular weight phosphotyrosine protein phosphatase | ACP1 | 2 | 2 | 26.6 | 14.342 | 21.65 | 21.15 | 22.05 | 20.09 | 20.99 | 21.15 | 0.87 | 1.83 | 0.10657 |
| P29218;P29218-3;H0YBL1;E5RIP7;E5RG13;E5RI82;P29218-2;E5RGY4;E5RG94;E5RHE9;E5RIF4 | Inositol monophosphatase 1 | IMPA1 | 11 | 11 | 44 | 30.188 | 25.48 | 25.06 | 25.37 | 24.63 | 24.32 | 24.36 | 0.87 | 1.83 | 0.00549 |
| Q9GZL7;B4DRY7 | Ribosome biogenesis protein WDR12 | WDR12 | 10 | 10 | 30.7 | 47.707 | 25.23 | 25.08 | 25.56 | 24.29 | 24.59 | 24.40 | 0.87 | 1.83 | 0.00630 |
| P51148;P51148-2;K7EIP6;F8WCY6;F8WD79;F8VPW9;F8VSF8;F8VWZ7;F8VWU4;F8VVZ0;F8VUA5;F8VVK3;K7ERI8;P61020-2;B4DJA5;P61020;P20339;K7ERQ8 | Ras-related protein Rab-5C;Ras-related protein Rab-5B;Ras-related protein Rab-5A | RAB5C;RAB5A;RAB5B | 2 | 2 | 10.6 | 23.482 | 20.70 | 20.44 | 20.46 | 18.59 | 20.24 | 20.16 | 0.87 | 1.82 | 0.18559 |
| A8K6Q8;G3V0E5;P02786;F5H6B1;B7Z2I6;H7C3V5;Q9UP52-2;Q9UP52-3;Q9UP52 | Transferrin receptor protein 1;Transferrin receptor protein 1, serum form | TFRC | 31 | 31 | 41.2 | 84.872 | 28.41 | 28.27 | 28.57 | 27.42 | 27.59 | 27.65 | 0.87 | 1.82 | 0.00146 |
| P28838-2;V9HW38;P28838;B4DQG5;H0Y9Q1;H0Y983 | Cytosol aminopeptidase | LAP3 | 31 | 31 | 75.6 | 52.771 | 30.08 | 30.10 | 30.11 | 29.01 | 29.39 | 29.29 | 0.86 | 1.82 | 0.00155 |
| Q15257-3;Q15257-2;A6PVN5;Q15257;B4DZF8;A6PVN9;Q5T949;Q5T948;Q15257-4;B4DLX5;Q68CR8;C9IZ76;B7ZBQ0;A6PVN8;A6PVN6;B7ZBP9;A6PVN7;B7ZBP7;B4DDQ6;H0Y6E5 | Serine/threonine-protein phosphatase 2A activator | PPP2R4 | 9 | 9 | 49.3 | 33.467 | 25.21 | 25.35 | 25.31 | 24.38 | 24.40 | 24.50 | 0.86 | 1.82 | 0.00010 |
| O76054-5;O76054;B2RAW8;B3KRD8;B7Z3Z8;O76054-4;H7C417;Q9UDX4;Q6XCI7;Q6ISB2;F8WEE7;C9JTM4;C9JZI9;B4DNV5;Q9UDX4-3;Q9UDX4-2;Q495V9;B5MC44;B3KYA6 | SEC14-like protein 2 | SEC14L2 | 5 | 5 | 25 | 36.636 | 22.04 | 21.53 | 21.29 | 20.94 | 21.04 | 20.31 | 0.86 | 1.81 | 0.05376 |
| C9JJ47;Q96CW1-2;Q96CW1;E9PFW3;C9JJD3;C9JPV8;C9JTK4;C9JGT8;B4DTI4;B7Z4N2;B4DJB1;B4DFM1;B4E304;B4DNB9 | AP-2 complex subunit mu | AP2M1 | 2 | 2 | 6 | 28.791 | 23.07 | 22.87 | 22.67 | 22.21 | 21.86 | 21.97 | 0.86 | 1.81 | 0.00524 |
| P49720;J3QKR3;J3KSM3;J3KRR2 | Proteasome subunit beta type-3 | PSMB3 | 16 | 16 | 54.6 | 22.949 | 28.18 | 28.28 | 28.16 | 27.11 | 27.41 | 27.54 | 0.86 | 1.81 | 0.00304 |
| Q9BY32-2;Q9BY32;Q9BY32-3;Q5NT82 | Inosine triphosphate pyrophosphatase | ITPA | 3 | 3 | 29.9 | 19.603 | 21.12 | 21.01 | 21.81 | 20.79 | 20.18 | 20.40 | 0.85 | 1.81 | 0.04948 |
| H0YHC3;F8W020;F8W118;F8VY35;F8VV59;B7Z9C2;A0A024RBE6;P55209-2;F8W0J6;F5H4R6;B7Z2V4;H0YIV4;P55209;H0YH88;F8W543;F8VRJ2;B7Z5H0;F8VXI6;F8VUX1;B3KV44;F8VVB5;E9PKT8;C9JZI7;B7ZAC7 | Nucleosome assembly protein 1-like 1 | NAP1L1 | 6 | 5 | 34.8 | 23.417 | 24.30 | 23.90 | 23.61 | 23.30 | 23.10 | 22.86 | 0.85 | 1.81 | 0.02235 |
| P53004;C9J1E1 | Biliverdin reductase A | BLVRA | 12 | 12 | 47.6 | 33.428 | 25.04 | 25.02 | 24.98 | 24.08 | 24.18 | 24.24 | 0.85 | 1.80 | 0.00006 |
| Q1JQ76;P62906;A0A024RCW3 | Ribosomal protein;60S ribosomal protein L10a | RPL10A | 8 | 8 | 39.3 | 23.536 | 26.56 | 26.88 | 27.19 | 26.33 | 25.84 | 25.92 | 0.85 | 1.80 | 0.02373 |
| O75369-5;O75369-4;O75369-6;O75369-3;O75369-2;O75369-9;O75369;A0A024R321;O75369-8;Q68CT4;E7EN95;O75369-7 | Filamin-B | FLNB;DKFZp686A1668 | 12 | 12 | 7.6 | 230.29 | 23.59 | 22.92 | 23.16 | 21.60 | 22.59 | 22.95 | 0.85 | 1.80 | 0.13259 |
| B4DIF8;D6W5C0;Q01082;K9MS24 | Spectrin beta chain, non-erythrocytic 1 | SPTBN1 | 6 | 6 | 25.1 | 39.717 | 24.22 | 24.34 | 24.51 | 23.48 | 23.55 | 23.52 | 0.84 | 1.79 | 0.00069 |
| Q9BRF8;Q9BRF8-2;Q9BRF8-3 | Calcineurin-like phosphoesterase domain-containing protein 1 | CPPED1 | 6 | 3 | 20.4 | 35.548 | 25.00 | 24.90 | 24.26 | 24.23 | 23.21 | 24.21 | 0.84 | 1.79 | 0.11056 |
| P78417;Q5TA02;P78417-3;B2R983;P78417-2;Q5TA01 | Glutathione S-transferase omega-1 | GSTO1 | 21 | 21 | 73 | 27.566 | 28.66 | 28.77 | 28.78 | 27.71 | 28.00 | 27.99 | 0.83 | 1.78 | 0.00122 |
| Q86TX2;B4DV16;P49753;B3KSA0;P49753-2;G3V4F2;B7ZMC1;A1L172 | Acyl-coenzyme A thioesterase 1;Acyl-coenzyme A thioesterase 2, mitochondrial | ACOT1;ACOT2 | 5 | 5 | 18.5 | 46.277 | 22.62 | 22.82 | 22.92 | 21.82 | 22.20 | 21.86 | 0.83 | 1.78 | 0.00510 |
| A8K8N7;O15067;A8K9T9;F5GWT9;B4DJ26;Q6P4B4;Q9BR56;H0YGH1;J3QSG0;J3QSH6;J3KT98;J3KTQ5;J3KTL4 | Phosphoribosylformylglycinamidine synthase | PFAS | 32 | 32 | 31.2 | 144.72 | 27.03 | 26.97 | 27.08 | 26.17 | 26.03 | 26.38 | 0.83 | 1.78 | 0.00148 |
| Q6P1N9;E5RG17;G5EA19;U3KQS5;E5RGF2;Q6P1N9-2;H0YBX2;E5RG62;E5RK70;E5RID7;E5RH86;E5RI69;E5RJY6 | Putative deoxyribonuclease TATDN1 | TATDN1 | 9 | 9 | 35.4 | 33.601 | 24.77 | 24.88 | 24.66 | 23.63 | 24.12 | 24.07 | 0.83 | 1.78 | 0.00798 |
| O95782-2;O95782;Q8N9K4;B4DXJ6;M0R2D9;B3KMI7;E9PR62;Q8N2F8 | AP-2 complex subunit alpha-1 | AP2A1 | 17 | 12 | 24.9 | 105.36 | 25.39 | 25.24 | 25.08 | 24.52 | 24.28 | 24.42 | 0.83 | 1.78 | 0.00178 |
| P63279;H3BPC4;H3BQQ9;A8K503;Q7KZS0;B0QYN7 | SUMO-conjugating enzyme UBC9 | UBE2I | 6 | 6 | 40.5 | 18.007 | 25.66 | 25.66 | 26.11 | 25.31 | 24.76 | 24.86 | 0.83 | 1.78 | 0.02131 |
| P14324-2;P14324;B3KMW3;Q14329 | Farnesyl pyrophosphate synthase | FDPS | 8 | 8 | 23.2 | 40.532 | 26.91 | 26.90 | 27.17 | 26.03 | 26.12 | 26.35 | 0.83 | 1.78 | 0.00311 |
| P04080 | Cystatin-B | CSTB | 6 | 6 | 79.6 | 11.139 | 26.58 | 26.33 | 26.64 | 25.45 | 25.58 | 26.03 | 0.83 | 1.78 | 0.01448 |
| Q9H3K6;Q9H3K6-2;H3BV85;H3BTW0;H3BVE0 | BolA-like protein 2 | BOLA2;BOLA2B;LOC101060252 | 2 | 2 | 40.7 | 10.116 | 21.53 | 22.10 | 21.78 | 21.01 | 21.07 | 20.84 | 0.83 | 1.78 | 0.00993 |
| Q6UXN9;C9JBU3 | WD repeat-containing protein 82 | WDR82 | 3 | 3 | 9.9 | 35.079 | 21.79 | 21.83 | 22.08 | 20.98 | 20.92 | 21.34 | 0.82 | 1.77 | 0.00676 |
| Q9Y617;B4DHQ3;Q9Y617-2;B4DHX7;A9LS35 | Phosphoserine aminotransferase | PSAT1 | 23 | 23 | 66.2 | 40.422 | 29.15 | 29.17 | 29.48 | 28.24 | 28.61 | 28.50 | 0.82 | 1.76 | 0.00589 |
| P00491;Q8N7G1;G3V5M2;G3V2H3;Q9P1G4;G3V393 | Purine nucleoside phosphorylase | PNP | 19 | 19 | 77.5 | 32.118 | 27.27 | 27.24 | 27.56 | 26.37 | 26.58 | 26.66 | 0.82 | 1.76 | 0.00364 |
| P52907;A8K0T9 | F-actin-capping protein subunit alpha-1 | CAPZA1 | 11 | 9 | 57.3 | 32.922 | 26.58 | 26.39 | 26.45 | 25.77 | 25.67 | 25.52 | 0.82 | 1.76 | 0.00087 |
| Q6XQN6-3;Q6XQN6;C9J8U2;C9JC60;Q6XQN6-2;G5E977;Q8WY82 | Nicotinate phosphoribosyltransferase | NAPRT1 | 6 | 6 | 15.4 | 56.099 | 22.42 | 22.28 | 22.12 | 20.96 | 21.69 | 21.73 | 0.82 | 1.76 | 0.03686 |
| Q5JR08;P61586;P08134;Q9BVT0;Q5JR07;C9JNR4;E9PQH6;B4DKN9;C9JX21;Q5JR05;Q5JR06;E9PN11;P62745;U3KQA9;U3KQV3 | Transforming protein RhoA;Rho-related GTP-binding protein RhoC | RHOC;RHOA;ARHA | 3 | 3 | 21.3 | 21.523 | 20.74 | 21.50 | 20.76 | 19.97 | 20.22 | 20.37 | 0.82 | 1.76 | 0.04063 |
| J3QRJ1;Q96FV2-2;Q96FV2;J3QL71;J3QR84;J3QQM9;J3KS57;J3KT79 | Secernin-2 | SCRN2 | 3 | 3 | 18.5 | 29.592 | 22.86 | 22.59 | 22.45 | 21.80 | 21.64 | 22.01 | 0.81 | 1.76 | 0.00692 |
| P13667;C9JMN9 | Protein disulfide-isomerase A4 | PDIA4 | 45 | 44 | 62.8 | 72.932 | 29.22 | 29.29 | 29.21 | 28.48 | 28.61 | 28.21 | 0.81 | 1.75 | 0.00260 |
| Q96CV8;P52888;K7EP46;Q9BW75;B4DU96;K7EKB6;K7EL02;K7EMU4;B3KSE2;K7EIK4;K7EL32 | Thimet oligopeptidase | THOP1 | 24 | 24 | 40.5 | 78.823 | 27.29 | 27.64 | 27.81 | 26.67 | 26.80 | 26.83 | 0.81 | 1.75 | 0.00722 |
| P04424-2;P04424;P04424-3;B4DU69;F8W943;Q6XYD2;H7C0S8 | Argininosuccinate lyase | ASL | 4 | 4 | 11.5 | 49.509 | 21.70 | 21.69 | 21.59 | 21.67 | 21.17 | 19.72 | 0.81 | 1.75 | 0.24006 |
| Q9HC38-2;B4DX01;A8K8F0;Q9Y3E8;Q9HC38;I3L3Q4;B7Z403;B3KV49;Q9HC38-3;I3L1F4;I3L1I0;I3NI27;I3NI24;I3L2C2;I3L277 | Glyoxalase domain-containing protein 4 | GLOD4;C17orf25 | 16 | 16 | 66.4 | 33.232 | 27.35 | 27.22 | 27.58 | 26.54 | 26.88 | 26.31 | 0.81 | 1.75 | 0.01427 |
| P18206-2;P18206;B3KXA2;B4E3Q9;Q5JQ13;B4DKC9;B4DTM7;P18206-3 | Vinculin | VCL | 50 | 50 | 53.8 | 116.72 | 28.99 | 28.41 | 28.39 | 28.11 | 27.65 | 27.61 | 0.80 | 1.75 | 0.03317 |
| Q92598-2;Q92598;Q92598-3;Q92598-4;B4DF68;B4DZB4;B4DY72;R4GN69;B4DXT2;B4DZP3;Q5TBM3 | Heat shock protein 105 kDa | HSPH1 | 18 | 17 | 30 | 92.115 | 25.20 | 25.06 | 25.14 | 24.17 | 24.36 | 24.47 | 0.80 | 1.74 | 0.00119 |
| P08107;B4DNT8;B4DI39;Q59EJ3;P08107-2;B4DWK5;B4DFN9;B4DVU9;E7EP94;B4E1S9;B4E388;V9GZ37;B3KTT5;B4E1T6;B4DNX1;B4DNV4;Q9UQC1 | Heat shock 70 kDa protein 1A/1B | HSPA1A | 22 | 12 | 38.2 | 70.051 | 27.24 | 27.08 | 27.34 | 26.44 | 26.19 | 26.63 | 0.80 | 1.74 | 0.00567 |
| Q0QF37;Q75MT9;Q6FHZ0;P40926;P40926-2;G3XAL0;B3KTM1 | Malate dehydrogenase;Malate dehydrogenase, mitochondrial | MDH2 | 19 | 19 | 70.5 | 31.969 | 30.04 | 30.02 | 30.26 | 29.15 | 29.32 | 29.46 | 0.80 | 1.74 | 0.00246 |
| Q53GC7;P47755;F8W9N7;C9JUG7;P47755-2;B4DE01 | F-actin-capping protein subunit alpha-2 | CAPZA2 | 9 | 9 | 44.4 | 32.967 | 25.07 | 25.18 | 25.05 | 24.24 | 24.13 | 24.53 | 0.80 | 1.74 | 0.00299 |
| B3KQG6;O95373;B3KNG9;Q59F59;O15397-2;O15397 | Importin-7 | IPO7 | 6 | 6 | 11.6 | 82.515 | 22.49 | 22.35 | 22.49 | 21.75 | 21.64 | 21.53 | 0.80 | 1.74 | 0.00053 |
| P10155-3;P10155-5;P10155-4;P10155;P10155-2;B3KN94;H0Y9N5;G5E9R9 | 60 kDa SS-A/Ro ribonucleoprotein | TROVE2 | 18 | 18 | 35.5 | 58.482 | 26.89 | 26.91 | 27.05 | 25.89 | 26.28 | 26.27 | 0.80 | 1.74 | 0.00430 |
| Q9UBQ0;Q9UBQ0-2;F8VXU5;Q5JPE4;E9RKB4;Q05DG7 | Vacuolar protein sorting-associated protein 29 | VPS29;DKFZp667O202 | 7 | 7 | 51.1 | 20.505 | 23.48 | 23.53 | 23.77 | 22.47 | 22.80 | 23.11 | 0.80 | 1.74 | 0.01755 |
| O76003 | Glutaredoxin-3 | GLRX3 | 9 | 9 | 41.8 | 37.432 | 23.91 | 24.20 | 24.41 | 23.65 | 23.66 | 22.81 | 0.80 | 1.74 | 0.06500 |
| P30041;A4UCS6;B4DUK1 | Peroxiredoxin-6 | PRDX6 | 18 | 18 | 77.2 | 25.035 | 28.01 | 27.82 | 28.00 | 27.04 | 27.09 | 27.31 | 0.80 | 1.74 | 0.00154 |
| Q15435-2;H7C003;Q15435;C9J177;C9JD73;B5MCY6;Q15435-4;B5MBZ8;Q15435-3;C9JRC4;H7C3Q5;H7C118 | Protein phosphatase 1 regulatory subunit 7 | PPP1R7 | 7 | 7 | 28.4 | 36.837 | 23.30 | 23.42 | 23.03 | 22.40 | 22.65 | 22.30 | 0.80 | 1.74 | 0.00688 |
| P46926;A8K3S1;D6RAY7;D6R9P4;B7Z3X4;E7EVU7;D6RFF8;B3KMV2;D6RB13;D6R917;D6RFK5 | Glucosamine-6-phosphate isomerase 1 | GNPDA1 | 14 | 9 | 68.2 | 32.668 | 25.99 | 26.01 | 26.32 | 25.02 | 25.44 | 25.49 | 0.79 | 1.73 | 0.01220 |
| P51858;B2RDE8;A8K8G0;B7Z958;P51858-2;P51858-3;B7Z525;Q8N4N4;M0R0J3;Q05CM9;O75475-3;O75475-2;O75475;Q7Z4V5-2;Q7Z4V5;Q7Z4V5-4;Q7Z4V5-3 | Hepatoma-derived growth factor | HDGF | 15 | 13 | 53.3 | 26.788 | 27.25 | 27.25 | 27.29 | 26.66 | 26.32 | 26.43 | 0.79 | 1.73 | 0.00149 |
| P52565;J3KRE2;J3QW41;J3KTF8;J3QQX2;P52565-2;J3KRY1;J3KS60 | Rho GDP-dissociation inhibitor 1 | ARHGDIA | 11 | 11 | 73 | 23.207 | 28.16 | 28.22 | 28.23 | 27.57 | 27.24 | 27.45 | 0.78 | 1.72 | 0.00134 |
| Q5W0S5;B4DEA3;Q53F10;P54727;Q5W0S4;B7ZA74;B7Z4W4;K7ENJ0;K7ELW1;P54725-2;P54725-3;A8K1J3;P54725 | UV excision repair protein RAD23 homolog B | RAD23B | 7 | 7 | 35.6 | 15.213 | 25.25 | 25.04 | 24.31 | 24.22 | 23.92 | 24.13 | 0.78 | 1.71 | 0.06001 |
| P16401 | Histone H1.5 | HIST1H1B | 3 | 3 | 10.6 | 22.58 | 22.81 | 22.66 | 23.22 | 22.03 | 21.99 | 22.34 | 0.78 | 1.71 | 0.01853 |
| O75828;Q53F60 | Carbonyl reductase [NADPH] 3 | CBR3 | 12 | 12 | 44 | 30.85 | 26.11 | 26.15 | 25.98 | 25.38 | 25.24 | 25.31 | 0.77 | 1.71 | 0.00031 |
| P25788-2;P25788;Q6IB71;G3V4X5;G3V3W4;G3V5N4 | Proteasome subunit alpha type-3;Proteasome subunit alpha type | PSMA3 | 17 | 12 | 62.9 | 27.647 | 29.28 | 29.37 | 29.39 | 28.39 | 28.84 | 28.51 | 0.77 | 1.70 | 0.00540 |
| O95394;O95394-4;B3KN28;O95394-3;J3KN95;H0Y8I3;D6RCQ8;D6RF77;D6RIS6;H0Y987;D6RC77;D6RCD1 | Phosphoacetylglucosamine mutase | PGM3 | 17 | 17 | 39.5 | 59.851 | 25.82 | 25.73 | 25.71 | 25.01 | 24.89 | 25.07 | 0.76 | 1.70 | 0.00026 |
| B4DDC8;B3KXL8;O15355;B2R665;Q96IN7;Q59GB2 | Protein phosphatase 1G | PPM1G | 3 | 3 | 6.6 | 56.134 | 21.35 | 21.03 | 21.39 | 20.48 | 20.77 | 20.25 | 0.76 | 1.69 | 0.01598 |
| Q9Y3A5;F8WE72 | Ribosome maturation protein SBDS | SBDS | 7 | 7 | 32 | 28.763 | 23.84 | 23.45 | 23.65 | 23.09 | 22.97 | 22.61 | 0.75 | 1.69 | 0.01414 |
| V9HW89;Q00796;B7Z3A6;H0YKB3;B4DKI2;H0YLA4 | Sorbitol dehydrogenase | SORD | 3 | 3 | 12.6 | 38.311 | 22.86 | 23.38 | 23.26 | 22.29 | 22.27 | 22.69 | 0.75 | 1.69 | 0.02198 |
| P28066;Q5U0A0;P28066-2 | Proteasome subunit alpha type-5;Proteasome subunit alpha type | PSMA5 | 13 | 13 | 66.4 | 26.411 | 28.55 | 28.71 | 28.87 | 27.62 | 28.23 | 28.01 | 0.75 | 1.69 | 0.02001 |
| Q2VPJ6;P07900;P07900-2;B4DTA5;Q86SX1;Q8TBA7;G3V2J8;Q86U12;Q96HX7;O75322 | Heat shock protein HSP 90-alpha | HSP90AA1 | 3 | 3 | 5.5 | 68.371 | 21.23 | 20.66 | 20.93 | 20.21 | 20.22 | 20.13 | 0.75 | 1.69 | 0.01096 |
| Q15185-3;Q15185-4;Q15185;B3KUY2;Q15185-2 | Prostaglandin E synthase 3 | PTGES3 | 2 | 2 | 23.1 | 14.958 | 21.62 | 21.32 | 22.02 | 20.92 | 20.76 | 21.02 | 0.75 | 1.68 | 0.02584 |
| Q04917;Q9H4N8;A2IDB2;B2R6N6;F8WEB6;A2IDB1 | 14-3-3 protein eta | YWHAH | 15 | 15 | 50.8 | 28.218 | 26.85 | 26.87 | 27.05 | 25.91 | 26.34 | 26.27 | 0.75 | 1.68 | 0.00726 |
| P50395;E7EU23;B4DLV7;P50395-2;Q5SX87;Q8TB95;B3KVE3;Q5SX86;Q5SX91;V9GYF8;Q5SX90;V9GYJ7 | Rab GDP dissociation inhibitor beta | GDI2 | 45 | 34 | 87.6 | 50.663 | 29.57 | 29.55 | 29.57 | 28.82 | 28.86 | 28.76 | 0.75 | 1.68 | 0.00002 |
| P50452;P50452-2;C9JVA8;H7BXK7;P50452-3;C9JTJ8;P50453;Q6N0A8;C9J7N5;O75830;B4DDY9 | Serpin B8 | SERPINB8 | 14 | 1 | 39.3 | 42.766 | 26.00 | 26.06 | 26.37 | 25.38 | 25.43 | 25.37 | 0.75 | 1.68 | 0.00288 |
| B1AK87;P47756-2;B2R7T8;B1AK88;B4DWA6;P47756;F6USW4;B1AK85;Q7L4N0;F6Q0E3 | F-actin-capping protein subunit beta | CAPZB | 18 | 18 | 78.1 | 29.295 | 26.87 | 26.82 | 26.75 | 25.88 | 26.23 | 26.09 | 0.75 | 1.68 | 0.00228 |
| P43155-3;P43155-2;P43155;B7ZBP5;A6PVN3 | Carnitine O-acetyltransferase | CRAT | 8 | 8 | 19.3 | 61.87 | 23.69 | 23.61 | 23.87 | 22.48 | 23.07 | 23.37 | 0.75 | 1.68 | 0.05173 |
| Q53G35;P18669;Q0D2Q6;Q6P6D7;Q6FHK8;B4DKL5;B4DMJ7;B7Z9E5;B4DJA4;P15259;Q8N0Y7;A4D2J6;B7ZW15 | Phosphoglycerate mutase;Phosphoglycerate mutase 1 | PGAM1 | 19 | 19 | 80.7 | 28.832 | 30.04 | 29.94 | 30.04 | 29.22 | 29.30 | 29.27 | 0.74 | 1.67 | 0.00005 |
| Q9H488;B4DUV4;Q9H488-2;B4DZS3 | GDP-fucose protein O-fucosyltransferase 1 | POFUT1 | 3 | 3 | 19.6 | 43.955 | 23.03 | 22.96 | 22.92 | 21.93 | 22.15 | 22.61 | 0.74 | 1.67 | 0.02189 |
| B3KM80;Q6ZS99;B3KTP9;P19338;H7BY16;Q9BQ02 | Nucleolin | NCL | 19 | 19 | 39.7 | 58.554 | 27.33 | 27.18 | 27.20 | 26.49 | 26.54 | 26.46 | 0.74 | 1.67 | 0.00012 |
| B3KW21;Q8WUI6;Q9Y678;H0Y8X7 | Coatomer subunit gamma;Coatomer subunit gamma-1 | COPG;COPG1 | 4 | 4 | 9.2 | 67.804 | 23.36 | 23.19 | 23.28 | 22.83 | 22.34 | 22.46 | 0.74 | 1.67 | 0.00897 |
| P27348;B4DMT8;Q53S41;B4DY04;E9PG15;Q53RR5 | 14-3-3 protein theta | YWHAQ | 12 | 12 | 40 | 27.764 | 26.43 | 26.45 | 26.53 | 25.80 | 25.66 | 25.74 | 0.74 | 1.67 | 0.00013 |
| P28070;B4DFL3 | Proteasome subunit beta type-4 | PSMB4 | 9 | 9 | 50.4 | 29.204 | 28.67 | 28.86 | 28.77 | 27.94 | 28.21 | 27.96 | 0.74 | 1.67 | 0.00199 |
| P55145;A8K878;H7C2D6 | Mesencephalic astrocyte-derived neurotrophic factor | MANF | 9 | 9 | 47.3 | 20.7 | 26.07 | 26.02 | 26.22 | 25.49 | 25.34 | 25.28 | 0.74 | 1.66 | 0.00109 |
| A8MZ77;I3L192;B4DY23;P35613-2;P35613;B4DNE1 | Basigin | BSG | 2 | 2 | 9.1 | 16.527 | 23.25 | 23.00 | 23.23 | 22.03 | 22.38 | 22.86 | 0.73 | 1.66 | 0.04544 |
| B4DJC3;O75367-2;O75367-3;O75367;Q59FH0;D6RCF2;Q5SQT3;Q9HA11;Q9P0M6 | Histone H2A;Core histone macro-H2A.1 | H2AFY | 3 | 3 | 14.5 | 21.548 | 22.32 | 22.27 | 22.24 | 21.66 | 21.42 | 21.54 | 0.73 | 1.66 | 0.00056 |
| B4DWV5;Q9HAV7 | GrpE protein homolog 1, mitochondrial | GRPEL1 | 6 | 6 | 34.2 | 21.9 | 23.54 | 23.27 | 23.31 | 22.78 | 22.40 | 22.74 | 0.73 | 1.66 | 0.00781 |
| B7Z992;P06396-2;B7Z2X4;P06396-4;P06396-3;B7Z9A0;B7Z6N2;P06396;B7Z4U6;Q5T0H9;B3KS49;Q5T0I0;Q5T0H8;Q5T0H7;Q5T0I1;V9H1C1;Q69YR8 | Gelsolin | GSN | 21 | 21 | 34.8 | 78.831 | 27.71 | 27.64 | 27.61 | 26.98 | 26.82 | 26.97 | 0.73 | 1.66 | 0.00023 |
| Q658P9;F8VZW6;Q8TAQ2-2;Q8TAQ2-3;Q59G16;Q8TAQ2;F8VXC8 | SWI/SNF complex subunit SMARCC2 | DKFZp313D0632;SMARCC2 | 2 | 2 | 32.4 | 11.527 | 19.39 | 19.46 | 19.82 | 19.38 | 17.66 | 19.45 | 0.73 | 1.66 | 0.28957 |
| Q6P587;Q6P587-2;Q6P587-3 | Acylpyruvase FAHD1, mitochondrial | FAHD1 | 6 | 6 | 40.2 | 24.843 | 25.35 | 25.40 | 25.93 | 24.14 | 25.09 | 25.27 | 0.73 | 1.65 | 0.14241 |
| H3BTZ6;H3BSV9;Q9Y303;Q9Y303-2;Q9Y303-3;Q59FV8;B4DUQ4;H3BTE3;H3BQ15 | Putative N-acetylglucosamine-6-phosphate deacetylase | AMDHD2 | 3 | 3 | 15.9 | 31.455 | 20.85 | 20.68 | 20.83 | 20.22 | 19.90 | 20.07 | 0.72 | 1.65 | 0.00242 |
| P31947-2;P31947 | 14-3-3 protein sigma | SFN | 2 | 2 | 10.6 | 24.336 | 29.73 | 29.91 | 29.63 | 29.00 | 28.91 | 29.18 | 0.72 | 1.65 | 0.00310 |
| P60900;G3V5Z7;G3V295;P60900-2;G3V3I1;P60900-3;G3V3U4;Q9BZ93;Q59F72;H0YJC4;G3V4S5;G3V2S7 | Proteasome subunit alpha type-6;Proteasome subunit alpha type | PSMA6 | 19 | 19 | 57.3 | 27.399 | 29.85 | 29.92 | 30.09 | 29.09 | 29.31 | 29.29 | 0.72 | 1.65 | 0.00181 |
| M5A959;P48507;D3DT44 | Glutamate--cysteine ligase regulatory subunit | GCLM | 2 | 2 | 10.7 | 28.135 | 20.92 | 19.92 | 20.68 | 18.97 | 20.27 | 20.12 | 0.72 | 1.65 | 0.22779 |
| A0A024RC87;P13489;H0YCR7;E9PIM9;E9PMJ3;E9PLZ3;E9PMN0;E9PIK5;E9PMA9;E9PR82;E9PMI1 | Ribonuclease inhibitor | RNH1 | 14 | 14 | 37.7 | 49.416 | 24.99 | 24.95 | 25.18 | 24.18 | 24.20 | 24.57 | 0.72 | 1.65 | 0.00791 |
| B4DZ08;Q99798;B2RBW5;A2A274;B4DLY4;B4DJW1;B4DW08;B4DEC3;Q71UF1;O75944 | Aconitate hydratase, mitochondrial | ACO2 | 26 | 26 | 45.5 | 83.412 | 26.04 | 26.50 | 26.44 | 25.08 | 25.84 | 25.89 | 0.72 | 1.65 | 0.07389 |
| Q9BTT0-3;Q53F20;Q9BTT0;E9PPH5;Q5TB19;B4E0D5;E9PLC4 | Acidic leucine-rich nuclear phosphoprotein 32 family member E | ANP32E | 3 | 3 | 15 | 25.125 | 21.67 | 21.48 | 22.31 | 21.09 | 21.17 | 21.03 | 0.72 | 1.65 | 0.04694 |
| Q92882;A8K646 | Osteoclast-stimulating factor 1 | OSTF1 | 2 | 2 | 11.2 | 23.787 | 21.66 | 21.22 | 21.25 | 21.03 | 20.49 | 20.44 | 0.72 | 1.65 | 0.03790 |
| P06703;R4GN98;B2R577 | Protein S100-A6 | S100A6 | 4 | 4 | 66.7 | 10.18 | 27.40 | 27.54 | 27.55 | 27.29 | 26.59 | 26.47 | 0.71 | 1.64 | 0.05195 |
| P21399;Q9HBB2;Q59FI0;Q5VZA6;F5H2R8;F5H143;F5H3Z7;O00408-5;E9PEF1;B4DIM1;O00408-4;O00408-3;Q8IW54;O00408;B3KT60 | Cytoplasmic aconitate hydratase | ACO1;IRP1 | 14 | 14 | 23.8 | 98.398 | 24.47 | 24.48 | 24.50 | 23.71 | 23.82 | 23.77 | 0.71 | 1.64 | 0.00003 |
| P60842;A8K7F6;A8K088;J3KT12;P60842-2;Q59F68;J3KTB5;J3QS69;J3QL43;B4DNH2;J3KSZ0;J3QR64;J3QLN6;J3KTN0;J3QKZ9;B4E047;J3KS25;B4DKP9;J3KS93;B4E102;J3KT04;J3QL52;J3QQP0;E7EMV8;Q86WD0;P38919;Q59GT8;Q5JWW0;E9PBH4;F8WE11;I3L3H2;D3DSD6 | Eukaryotic initiation factor 4A-I | EIF4A1 | 17 | 7 | 45.3 | 46.153 | 27.08 | 26.87 | 26.91 | 26.45 | 26.23 | 26.04 | 0.71 | 1.64 | 0.00605 |
| P14550;Q5T621;V9GYG2;V9GYP9 | Alcohol dehydrogenase [NADP(+)] | AKR1A1 | 21 | 21 | 67.7 | 36.573 | 28.51 | 28.57 | 28.83 | 27.88 | 27.93 | 27.97 | 0.71 | 1.64 | 0.00232 |
| P22314;Q5JRR6;B3KUJ2;B4DDE4;B4DL67;Q8WY81;Q5JRS1;Q5JRS3;Q5JRS2;Q5JRR9;Q5JRS0 | Ubiquitin-like modifier-activating enzyme 1 | UBA1 | 6 | 6 | 8.4 | 117.85 | 21.48 | 21.80 | 21.36 | 20.86 | 20.29 | 21.36 | 0.71 | 1.64 | 0.10171 |
| Q9Y696;B4DWC4;Q9NVF8;B3KTR3;A6PVS0;Q7Z2R8;Q86YM0;Q9NZA1-3;O15247;Q9NZA1-2;B2RAZ0;Q9NZA1;Q96NY7-2;Q96NY7 | Chloride intracellular channel protein 4 | CLIC4 | 16 | 16 | 78.3 | 28.772 | 28.39 | 28.31 | 28.32 | 27.59 | 27.84 | 27.47 | 0.71 | 1.63 | 0.00309 |
| P36871;B4DFP1;P36871-2;B7Z6C2;P36871-3;B4DDQ8;Q9H1D2 | Phosphoglucomutase-1 | PGM1 | 21 | 21 | 53.9 | 61.448 | 27.05 | 27.01 | 26.95 | 26.46 | 26.31 | 26.12 | 0.70 | 1.63 | 0.00239 |
| A6NJU6;Q9UKK9;A6NFX8;H0YEY4;A6NCQ0 | ADP-sugar pyrophosphatase | NUDT5 | 2 | 2 | 17.2 | 20.629 | 20.05 | 19.82 | 20.33 | 18.47 | 20.00 | 19.64 | 0.70 | 1.63 | 0.22275 |
| Q9H3H3;Q9H3H3-2;Q9H3H3-3 | UPF0696 protein C11orf68 | C11orf68 | 2 | 2 | 9.2 | 27.354 | 20.97 | 21.65 | 20.71 | 20.51 | 20.39 | 20.33 | 0.70 | 1.63 | 0.06998 |
| F8VNS3;C9J2G3;C9JQQ2;Q9NWU1-2;Q9NWU1 | 3-oxoacyl-[acyl-carrier-protein] synthase, mitochondrial | OXSM | 2 | 2 | 28.4 | 11.184 | 19.97 | 20.14 | 19.78 | 19.21 | 19.46 | 19.13 | 0.70 | 1.62 | 0.00819 |
| P61964;V9GZ59;V9GYQ5;Q86VZ2 | WD repeat-containing protein 5 | WDR5 | 14 | 14 | 64.4 | 36.588 | 25.44 | 25.84 | 25.82 | 24.83 | 25.37 | 24.82 | 0.69 | 1.62 | 0.03668 |
| P67936;B4DVY2;P67936-2;B4DTB1;K7ENT6;K7ERG3;K7EMU5;K7EPV9;K7EPB9;Q2TAC2-2;Q2TAC2 | Tropomyosin alpha-4 chain | TPM4 | 31 | 19 | 73 | 28.521 | 30.16 | 30.13 | 30.37 | 29.83 | 29.49 | 29.27 | 0.69 | 1.62 | 0.01900 |
| P62937;A8K486;B2RE56;B4DM82;Q71V99;F8WE65;C9J5S7;Q567Q0;E5RIZ5;Q9Y536;F5H284;A2BFH1 | Peptidyl-prolyl cis-trans isomerase A;Peptidyl-prolyl cis-trans isomerase A, N-terminally processed;Peptidyl-prolyl cis-trans isomerase | PPIA | 16 | 14 | 86.1 | 18.012 | 31.26 | 31.16 | 31.42 | 30.52 | 30.71 | 30.53 | 0.69 | 1.61 | 0.00199 |
| Q8TCD5;J3KSY6;J3KRC4;J3KSX6;Q8TCD5-2 | 5(3)-deoxyribonucleotidase, cytosolic type | NT5C | 6 | 6 | 40.3 | 23.382 | 23.28 | 23.50 | 23.75 | 22.23 | 23.02 | 23.22 | 0.69 | 1.61 | 0.10734 |
| Q53GX7;P26639;P26639-2;Q5M7Z9;B2RDX5;G3XAN9;B3KTN2;E7ERI3;B4DKZ9;D6R9F8;D6RCA5;D6RDJ6;A2RTX5-2;A2RTX5;D6RBR8;D6RJ97;D6RHV7;D6RCS6;B7ZLP8 | Threonine--tRNA ligase, cytoplasmic | TARS | 26 | 26 | 40.1 | 83.444 | 26.52 | 26.41 | 26.47 | 25.67 | 25.87 | 25.81 | 0.69 | 1.61 | 0.00054 |
| Q96QK1;Q5HYM2;Q53FR4 | Vacuolar protein sorting-associated protein 35 | VPS35;DKFZp686O2462 | 14 | 14 | 23.6 | 91.706 | 23.66 | 24.05 | 23.84 | 22.91 | 23.02 | 23.57 | 0.69 | 1.61 | 0.04251 |
| P28072;I3L3X7 | Proteasome subunit beta type-6 | PSMB6 | 6 | 6 | 37.7 | 25.357 | 28.85 | 29.07 | 29.21 | 28.42 | 28.35 | 28.30 | 0.68 | 1.61 | 0.00366 |
| P63167;F8VXL2;F8VRV5;Q96FJ2 | Dynein light chain 1, cytoplasmic;Dynein light chain 2, cytoplasmic | DYNLL1;DYNLL2 | 2 | 2 | 25.8 | 10.366 | 22.84 | 22.59 | 21.80 | 21.38 | 22.61 | 21.20 | 0.68 | 1.60 | 0.27737 |
| Q6NZ44;P02794;B3KXC3;Q6NS36;G3V192;G3V1D1;E9PRK8;Q8TD27;A9JQE1;E9PQR3;E9PPQ4;E9PKM5;E9PK45;D3DPG2 | Ferritin;Ferritin heavy chain | FTH1 | 10 | 5 | 57.4 | 21.241 | 26.73 | 26.89 | 26.65 | 26.19 | 26.35 | 25.70 | 0.68 | 1.60 | 0.03086 |
| P25789;B2RDG0;H0YMZ1;H0YN18;H0YL69;Q7Z474;P25789-2;H0YMA1;H0YKT8;H0YMI6;H0YKS0;H0YLC2;H0YMV3;H0YLS6 | Proteasome subunit alpha type-4;Proteasome subunit alpha type;Proteasome subunit beta type | PSMA4 | 16 | 16 | 75.1 | 29.483 | 29.11 | 29.33 | 29.28 | 28.46 | 28.79 | 28.43 | 0.68 | 1.60 | 0.00724 |
| Q9BRA2;I3L2R6;I3L0K2;I3L3M7 | Thioredoxin domain-containing protein 17 | TXNDC17 | 7 | 7 | 60.2 | 13.941 | 27.06 | 27.14 | 27.68 | 26.37 | 26.66 | 26.81 | 0.68 | 1.60 | 0.04389 |
| P07108;B8ZWD1;P07108-2;P07108-4;B8ZWD9;P07108-5;B8ZWD8;P07108-6 | Acyl-CoA-binding protein | DBI | 4 | 1 | 39.1 | 10.044 | 24.00 | 24.20 | 23.76 | 23.29 | 23.52 | 23.12 | 0.68 | 1.60 | 0.01693 |
| P06737-2;E9PK47;P06737;B2R825;P11217-2;Q6P1L4 | Glycogen phosphorylase, liver form;Phosphorylase | PYGL | 21 | 15 | 28.9 | 93.133 | 26.01 | 25.83 | 26.11 | 25.19 | 25.32 | 25.41 | 0.68 | 1.60 | 0.00300 |
| Q969H8;M0QYN0;M0QXF7 | UPF0556 protein C19orf10 | C19orf10 | 6 | 6 | 39.9 | 18.795 | 27.33 | 27.70 | 27.94 | 26.32 | 27.35 | 27.29 | 0.67 | 1.59 | 0.14926 |
| O14818;Q05DH1;O14818-2;H0Y586;O14818-4;H0UI83;Q8TAA3-2;B0YJ74;Q8TAA3-5;Q8TAA3;F5GY34;Q4G1B8 | Proteasome subunit alpha type-7;Proteasome subunit alpha type | PSMA7 | 19 | 19 | 64.5 | 27.887 | 29.83 | 29.88 | 29.96 | 29.03 | 29.48 | 29.14 | 0.67 | 1.59 | 0.00910 |
| O15144;Q9BXV5;A0A024R408;C9JTV5;G5E9J0;H7C3F9;G5E9S7 | Actin-related protein 2/3 complex subunit 2 | ARPC2 | 19 | 19 | 61.7 | 34.333 | 26.66 | 26.64 | 26.32 | 25.90 | 25.80 | 25.92 | 0.67 | 1.59 | 0.00449 |
| Q9H8S9;Q7L9L4;Q7L9L4-2;D6RCK3;B3KSH6;Q9H8S9-2;H7BZ12;Q4W5E0 | MOB kinase activator 1A;MOB kinase activator 1B | MOB1A;MOB1B;MOBKL1A;MOB4A | 3 | 3 | 17.1 | 25.079 | 22.05 | 22.11 | 21.96 | 21.81 | 21.34 | 20.98 | 0.67 | 1.59 | 0.05212 |
| P36639-4;P36639-3;P36639-2;P36639;C9J361 | 7,8-dihydro-8-oxoguanine triphosphatase | NUDT1 | 5 | 5 | 34 | 17.951 | 25.44 | 25.47 | 25.82 | 24.97 | 25.04 | 24.72 | 0.67 | 1.59 | 0.01277 |
| Q53FA7;B4DMQ7;Q53FA7-2;H7BZH6 | Quinone oxidoreductase PIG3 | TP53I3 | 18 | 18 | 66.3 | 35.536 | 27.79 | 27.91 | 28.09 | 27.24 | 27.38 | 27.19 | 0.67 | 1.59 | 0.00308 |
| Q9BZK7;B7Z475;B4DP68;Q9BQ87 | F-box-like/WD repeat-containing protein TBL1XR1 | TBL1XR1 | 14 | 8 | 34.2 | 55.594 | 26.38 | 26.32 | 26.40 | 25.54 | 25.91 | 25.66 | 0.66 | 1.59 | 0.00393 |
| Q8NCW5-2;Q8NCW5;B4DP80;Q5T3I4;Q6PGN4;Q5T3I3 | NAD(P)H-hydrate epimerase | APOA1BP | 11 | 11 | 73.5 | 20.43 | 26.76 | 27.04 | 27.10 | 26.14 | 26.21 | 26.56 | 0.66 | 1.58 | 0.01739 |
| Q9BR76;F5H390;Q9NSK3;Q59F81;E7EW44;B4DIU2;F5H0D2 | Coronin-1B;Coronin | CORO1B;DKFZp762I166 | 5 | 5 | 14.1 | 54.234 | 22.98 | 23.24 | 22.88 | 22.27 | 22.46 | 22.40 | 0.66 | 1.58 | 0.00561 |
| Q59EZ3;P11717;Q13660 | Cation-independent mannose-6-phosphate receptor | IGF2R | 3 | 3 | 1.8 | 266.11 | 22.53 | 22.40 | 22.48 | 22.09 | 21.83 | 21.52 | 0.66 | 1.58 | 0.01747 |
| P35754;D3DQK5 | Glutaredoxin-1 | GLRX | 6 | 6 | 58.5 | 11.776 | 25.48 | 25.48 | 25.57 | 24.78 | 24.85 | 24.93 | 0.66 | 1.58 | 0.00023 |
| P41250;Q75MN1 | Glycine--tRNA ligase | GARS | 11 | 11 | 21.2 | 83.165 | 24.14 | 23.92 | 23.78 | 23.19 | 23.17 | 23.52 | 0.65 | 1.57 | 0.01386 |
| O95881;V9GY50;V9GYV4 | Thioredoxin domain-containing protein 12 | TXNDC12 | 3 | 3 | 19.2 | 19.206 | 23.87 | 23.67 | 23.13 | 23.09 | 22.83 | 22.80 | 0.65 | 1.57 | 0.05185 |
| H7C585;C9JAX1;Q16595;Q16595-2;Q16595-3 | Frataxin, mitochondrial;Frataxin intermediate form;Frataxin(56-210);Frataxin(78-210);Frataxin mature form | FXN | 2 | 2 | 22.2 | 11.901 | 19.81 | 19.80 | 21.07 | 19.78 | 19.51 | 19.44 | 0.65 | 1.57 | 0.20660 |
| B4DY28;P21291;Q9BTA4;B3KVC9;Q6ZMS3;B4E2T4;E9PS42;E9PND2;Q59EQ5;E9PP21;Q6AI18;Q6AWD1 | Cysteine and glycine-rich protein 1 | CSRP1;DKFZp686M148 | 6 | 6 | 49.7 | 20.042 | 24.08 | 24.18 | 24.21 | 23.86 | 23.33 | 23.32 | 0.65 | 1.57 | 0.02360 |
| H7C386;Q9BW91-2;Q8NG26;Q9BW91;Q96KB3;D6RAW2;D6R8Z6 | ADP-ribose pyrophosphatase, mitochondrial | NUDT9 | 4 | 4 | 15.8 | 31.539 | 22.70 | 22.00 | 22.63 | 21.37 | 21.96 | 22.05 | 0.65 | 1.57 | 0.10470 |
| Q96DG6;B2RD36 | Carboxymethylenebutenolidase homolog | CMBL | 5 | 5 | 22 | 28.048 | 24.52 | 24.99 | 24.29 | 23.70 | 24.65 | 23.52 | 0.64 | 1.56 | 0.18856 |
| Q04828;H0Y804;P52895;A6NHU4;B4DK69;B4E0M1;Q59GU2;S4R3P0;B4DKT3;S4R3D5;P17516;P52895-2 | Aldo-keto reductase family 1 member C1;Aldo-keto reductase family 1 member C2 | AKR1C1;AKR1C2 | 16 | 7 | 64.1 | 36.788 | 28.21 | 28.21 | 28.35 | 27.49 | 27.52 | 27.84 | 0.64 | 1.56 | 0.00636 |
| P11216;Q59GM9;B4DSD8;H0Y4Z6;Q8TDG6;B4DRQ4 | Glycogen phosphorylase, brain form;Phosphorylase | PYGB | 16 | 15 | 22.8 | 96.695 | 24.76 | 24.56 | 24.79 | 24.00 | 23.88 | 24.32 | 0.64 | 1.56 | 0.01381 |
| P09211;A8MX94;C7DJS1;C7DJS2;B2C310;Q5D6A5 | Glutathione S-transferase P | GSTP1 | 12 | 12 | 70.5 | 23.356 | 27.21 | 27.38 | 27.39 | 26.42 | 26.82 | 26.83 | 0.64 | 1.56 | 0.01250 |
| P11387;B9EG90;Q6PK95;Q6NWZ5;D3DWJ7;E5RIC7;B4E061;Q969P6-2;B7Z9E8;B4DYD2;Q969P6;E5KMK6;E5KMK5 | DNA topoisomerase 1 | TOP1 | 3 | 3 | 5.4 | 90.725 | 20.88 | 21.06 | 20.67 | 20.36 | 20.61 | 19.73 | 0.64 | 1.55 | 0.09004 |
| Q08257;Q08257-3;A6NP24;C9JH92;Q08257-2 | Quinone oxidoreductase | CRYZ | 20 | 20 | 70.2 | 35.206 | 28.24 | 28.47 | 28.54 | 27.65 | 27.93 | 27.77 | 0.63 | 1.55 | 0.00638 |
| B4DY09;Q53FG3;Q12905;F4ZW63;X6R6Z1 | Interleukin enhancer-binding factor 2 | ILF2 | 2 | 2 | 6.8 | 38.91 | 21.13 | 21.01 | 21.10 | 20.24 | 20.45 | 20.66 | 0.63 | 1.55 | 0.00728 |
| P34932;B4DH02;V9HW33;Q59GF8;B4DUS3;B4DIZ3;B4DT47;B4E354;O14992;B4E0H6;Q7KYN0;B0AZU6;B4DSE8;B0AZS1;B0AZT0;B4DN48;Q9BUK9 | Heat shock 70 kDa protein 4 | HSPA4;HS24/p52 | 49 | 46 | 59.4 | 94.33 | 28.83 | 28.81 | 28.87 | 28.09 | 28.25 | 28.27 | 0.63 | 1.55 | 0.00044 |
| R9UTP9;R7RV73;H6SSQ0;G8ZKX4;G4V593;C7U2Z0;B6ECH3;A7WPI8;Q0E7X3;B7VF83;F6IQR9;P30443;M4QEH4;C5IWX4;Q59GJ2;M5EE32;A9QUT7;W8SKC0;H6SSQ1;E0WN92;N1NV67;C5J3U2;S0F2I4;J7JHS1;E0WN90;H6SHQ4;F6IQS0;U5YKD6;Q95IG4;U5YMM6;B7ZAP7;Q5MCQ6;Q95HA5;Q5ZGM8;P30455;X5LKD6;Q2A689;I4EPX9;T2MJM6;E0WN84;Q5ND69;X2KUP9;W0NUD2;W0HHM2;S4T750;Q9GIN6;Q5FZM6;Q38HW6;L7PHW3;K9LC03;J7F8K9;I3UI80;G9HW25;G9HRN4;G1EP36;G1EP31;G1END6;F8SKT6;F8R8G6;F8R136;F6KRJ7;F4YU29;E2GJS1;E2GJE8;D7NNS1;D6MLN2;D6ML59;D5M8G0;D5FHR8;D5FHM4;C9WEM3;C9WEM1;C8CH70;C6K4H7;C6K4F8;C5IZR5;C5IZP9;C0M145;A4USG9;A4USG6;O43828;E1Y6U1;A4URH5;D0RB02;R7RVB7;L7UVL0;L8B936;S4T6J6;M9P8T1;H6V076;F8SKV3;C6K4E1;L7UPW6;L0R375;G9HW62;G1EPF9;E0X5Z5;D5L998;C9E1D4;C6K4G9;Q9TQ27;Q9MY40;N0AA25;H6V093;D7NNR6;D6ML36;D5FZI7;A1Z0L7;K9LC09;K7XSD6;I6SJ70;G1ENE0;F6KRJ0;E9LXZ0;E0WN50;E0WBY9;D7GN22;C6K4G5;C6K4F2;C6K4E6;B8YCR8;I2B2W7;G8GIE0;A7X543;A1Z0L4;Q6PW03;D5G1Q4;A6H345;F6KRL6;F2VP63;D7NP79;Q861H1;A7X516;S4T6V4;Q6Q3G5;M9PA18;J7GU98;D7NQT7;D7NNP0;A5YVG8;T1R3B2;M1FUR2;J9PVP6;E0YTH8;D7NNS0;D6MLL3;D5FHH6;D3U476;C5IZP5;A7X549;B3GW70;W6CHX3;J7SBK9;G8FQ53;E1B2D6;C7U1K2;C7E539;C6H0N8;B6ECH6;B6ECH2;A7MAK2;D0AB29;Q5SRN7;Q861B7;U5YMP4;F6IQV7;F6IQV6;B4E2X4;X5MPH3;X5MI21;X5MFE4;Q29840;X5D2K4;P04439;D5H3U2;D5H3U1;C5IWY0;B2R7U3;A9YWM1;Q5SRN5;Q5FZM7;D6MLH7;A5JSG6;A0ZXY8;V6CKU4;I2E8C4;E1Y422;L7X986;G5CJS3;B7VCC4;W8NKP1;W6SIV4;S5U3G8;S0F2B1;R4KXX0;Q9TQP8;Q6F3E3;M5ETC1;M5A8I6;J7I2U3;I3VB21;H2D5G2;G0UE14;G0M6G1;F2XI31;E3WH33;D3YFD2;D1MZT7;D0W032;D0VFI1;C7C6D3;C7C695;B6VA01;B3VE19;B2DFW7;B1Q4X5;B1Q4X2;Q0E7X7;D9UAY2;D5LN74;B1PT16;G9FTK2;G0Z6Y6;Q7YPW4;F6IQV8;U5YMQ3;U5YMI4;U5YME5;U5YKF9;U5YKF4;U5YJQ0;I2GAD6;F6IQZ1;F6IQZ0;F6IQY1;F6IQX7;F6IQX6;F6IQX5;F6IQX1;F6IQW8;F6IQW4;F6IQW0;X5MFD7;A7MAP4;I0J2M4;A1Z1D7;Q2A688;P13746;H2DMX5;F4NCR9;D9UB11;D9UB10;D7GM35;D5H3U4;D5H3U3;D0V0C4;C5IWZ2;P13746-2;H2BE78;A0JHM7;D6CIB2;B7VBV1;R4NLT9;I3TBB9;I0DFI9;L7Y4K8;I7B4Y2;C9E9Y0;V5L1W2;F2XI30;E9ABI3;E1Y6M4;Q6IVJ9;F6IQX3;A7DZQ5;F6IQY9;F6IQY8;F6IQY7;F6IQW3;F6IQW2;F6IQW1;U5YMI0;U5YJM7;F6IQY4;F6IQY3;F6IQX9;F6IQX8;X5MI15;X5MBH2;D9UB05;E0WMV1;F2VNG9;X2L4X5;U5IT50;U5IQK9;S4TZK2;R9R080;R4QGE9;R4L314;R4I3G2;Q9TQF2;Q9MYA8;Q95IH7;Q6UJZ9;Q5JZM7;Q5FYV2;Q2WBP4;Q000J8;M4N7U4;M4MD01;M1FWW1;K9LC23;K9LC01;J7F7A9;J7F4Y9;I6RGW8;I6RGW0;I6QU03;I3QHR2;H9BNV2;G9HW64;G9HRN9;G9HRL2;G9HRL1;G1EPG4;G1EPB3;G1EP93;G1EP75;G1EP22;F8RHD8;F8RHD6;F8R115;F6KRP7;F6KRL8;F6KRK7;F2VYI1;F2VNJ1;F2VNG7;E5DCM1;E5D6K2;E3SWG4;E0WBZ1;D7NSP9;D7NQU0;D7NQT1;D7NPL4;D7NPL2;D7NPA6;D6MLN5;D6MLI3;D6ML63;D6ML13;D6ML12;D6ML07;D6C6A4;D5M8G7;D5FZI6;D5FHR2;D5FHQ8;D4P2Y0;D3U764;D3U477;D2XUR4;D2SSL9;D2DKV2;C9WEL9;C9E848;C8XTP2;C8XTN8;C7E580;C6K4G4;C6K4F7;C6K4E4;C4PFY9;B8Y6A7;B5ATU6;B1PQ33;B1PL02;A8DA04;A7X571;A7E1C1;A4USG8;A2VBX5;F4NC94;Q3BK35;B4DVB9;B4DVC4;Q45NE1;E0WMV3;B2DFW8;X4YT82;U6BZQ8;M9T819;I0DHJ2;G9FTJ8;C0MP56;F6IQW7;Q8MGZ1;D5H3U6;C9EIW0;F2VNJ8;U5YBW6;Q2MCK0;A7L5K9;F2VNJ7;O19603;O19601;D0EP74;B9X247;L7PH07;H6UV64;F2X5Y4;F2VP60;E0X9K3;M4N6H6;E0WBW7;M4SNZ1;M1FXT8;I6NWN3;H9C5F8;C9E846;C9E1D6;C5IZR2;Q208P6;K7P5E7;I6QU20;H2BE91;G1ENE6;G0X8R3;E5G0Y5;D7NSP6;D7NNM8;D5L999;D2JZZ8;D1MEQ1;A5Z1D4;X2KXS7;X2KUQ3;V6BQQ4;S5CJX5;R4QT49;R4QS31;Q9TQG9;Q95HD8;Q6UJZ8;Q6T866;Q5MBP3;Q53ZQ3;Q2L9H3;Q2I0Y9;Q1WAA8;Q1KLJ8;Q1G4P3;Q0ZAX0;Q0KH41;O46853;M4MFB9;M1F363;K7R1R0;K7P5I9;K7P4Y8;J7FR13;I6QQS7;I3UI53;G9HW27;G1EP12;G1EP09;G1ENC2;G0WVA2;F8SWL5;F8SKU1;F8R8I3;F2X5Z6;F2VNI3;F2VNE8;F1C3G8;E5G0Y7;E0YTI9;E0YTH1;E0WN55;D7NPA7;D7NP90;D7NNS6;D6MLK9;D6MJA6;D6MJ98;D6MJ94;D6C6C7;D5FIG3;D5FIF3;D5FHR9;D3Y5Y2;D3VPJ4;D3U478;C6K4D9;B8YCR9;B6VAW9;B3V8R1;A8VYP6;A6N8R8;A4L9U0;Q8MGZ4;Q1XG28;D2U6Y7;A7M780;D3JW64;W6CGW3;W0FBM8;V5V1E3;U3R9Y1;T2BQE4;S6B3R2;S4VA55;Q9BCN8;Q95IG6;Q6L675;Q6F3E2;Q4ZG93;Q4W5U0;Q3YBM1;Q2PP85;Q25BN7;Q1EPW2;Q1EJP6;Q0E7X5;M5EFF5;M4X2U5;M4NUQ2;M4NJM4;M1LMH3;M1KDJ9;L7QJ95;J7HGX9;I7HCZ5;I7H3R1;I6R2W1;I6R2V6;I6M4H5;I2GUK1;H9C3L3;E9ABX3;E7BJ52;E3Q0X4;E0YW13;D9UC12;D5H3W3;D2KZ55;D2KZ53;D1MYY9;C6L836;C3VAL4;C1KBJ6;B3VE20;B3IYE6;B2NJ13;B2DFV0;B2DFH6;B2DFH5;B0FWH0;A4URG8;A4GWZ1;A4GWZ0;A2VC06;A2RQE0;A0SXS8;Q2L4F0;Q14SN2;D9UAY3;D6CIC0;D6CIB9;D5H3V9;D5H3U7;B5A9M9;Q6IVJ8;Q6IVJ7;Q5D1X1;U4PDP4;Q860B5;Q860B4;C0KLT0;U5YMJ4;U5YMF0;U5YKG9;U5YJQ4;U5YJH0;F6IR16;F6IR15;F6IR14;F6IR13;F6IR12;F6IR11;F6IR09;F6IR08;F6IR05;F6IR03;F6IR02;F6IR01;F6IR00;F6IQZ9;F6IQZ8;F6IQZ6;F6IQZ5;F6IQZ2;D9UAY1;A8YQE6;X5MBH7;O15506;L8B934;Q09160;Q9TQ74;Q9MY51;Q708C4;Q29907;Q29689;P30447;P05534;E5FQ47;D5H3V3;D5H3U9;D5H3U8;C5IWY4;C5IWY3;B6ETN7;U5YCF9;U5YCB5;U5YBI5;U5YBG6;U5YBF2;U5YBC7;V5LDZ9;I0BW65;I0BW49;I0BW39;E3SWF9;E2D5M5;D7NNP2;D6ML21;M1L8W8;K7QX28;D6MJC9;T1WF44;R9QZR4;Q6V117;Q5ENH3;Q45FD6;Q09HS6;Q05G01;L7PH16;J7FKW9;I3UI51;H9C5F6;H6V073;F1AQL5;D7NP83;D7NNR7;D7NNR3;C6K4H4;B8Y1X8;A6H583;V6BQN7;M9P9E7;K7RE34;G1ENZ9;E2GJE3;C5IZS0;V6BQN6;Q95J02;Q8WMB6;K9LC14;J9UEL0;I6NS18;H9E8V4;F8R121;F6KRN7;F2X5X0;F2VND9;E5G0Y9;D7NPB2;D6C699;C0M138;B1PL01;D6Q0R2;B9VJL7;R4L6H2;K4ML26;B0LL97;G8ZKX2;E0WN58;E0WN57;S0F2B5;Q5NTA9;L7V338;I1U8N0;F2XI33;S6BEP5;S6B0Q5;S4VAM7;R4L6P2;Q6L625;Q6I6G7;Q5NTA3;Q4ZG94;Q0VJ91;M5EF02;M5A7E7;K4ULX4;K4NRQ4;I7H3R2;I7GSC3;I6Y0B1;I6M4H6;I2GUK2;H2AM05;H2AM00;G9G814;G4VU13;F8WSC7;E3WH35;E2GHW7;D2KZ56;D2KZ33;D1MZT9;C6H0N9;B7XH57;B6ECH4;B3WFC8;B2G3P7;B2DFV8;B2CZA7;A0PBY4;P79556;D7RTU8;Q6ZUK5;D9UB28;A5PHT7;Q19BJ8;F0VRV7;A6YT90;U5YMM3;U5YKD2;U5YJN1;F6IR21;F6IR18;F6IR17;F6IR25;U5YKG4;X4YZB9;G9FTK0;F6IR55;F6IR27;E2FIL8;X5MBG6;M9QZ33;D9UB08;Q9MY52;P30457;P30456;P30450;P18462;I2HA75;D7GM33;C5IWY6;B7VU66;I0BW63;U5YEC8;U5YEB7;U5YCC0;U5YCB1;U5YC91;U5YCL3;V5LDS3;V5LDH8;I0BW67;I0BW54;I0BW53;H9C3K4;H9C3J8;H9C3J2;H9C3J1;H9C3J0;H9C3I4;H9C3I1;H9ZYJ4;Q9UEX6;Q0MSI1;Q95IH3;Q70GH1;A1E125;O78086;Q861Q7;R7RU26;B4DVX9;E7AJF9;R4ZGM9;Q5G0H2;L7PG07;K9L7Y7;H9BNV1;G1EPA0;E0WN56;D6MLP5;D3YN68;C9E847;U5YRJ1;Q9TQG8;Q700J7;Q5ENW1;Q29756;J7FM86;I6RGU8;I6QQT0;G1EPD4;G1EP81;F2X5X6;F2VYI6;F1CK27;E9LXZ2;E1U677;D7R0W2;D6MJ97;D5G235;D5FHN8;D5FHK9;D5FHH2;D3G9K2;C7FDP9;B8Y1X7;C4PIH6;F6IB50;Q75NY8;U6ERM7;Q5NTA2;Q4QZC0;I6S8T6;E0WN61;U5YMR3;G1DUW4;K4UF89;A5CLH5;U5YED9;U5YED5;U5YE88;U5YE82;U5YE78;U5YCD0;U5YCA6;U5YBP0;U5YBJ7;U5YBJ3;Q9TQ84;U5YEJ4;U5YEG3;U5YEE9;U5YBM8;U5YEP2;U5YEK8;U5YEG8;U5YEF3;U5YCI0;U5YBP5;U5YBJ6;V5LDU7;V5LDH2;I0BW56;I0BW46;W6RT42;F1C5F3;E5G6F9;A1E124;E0WMX4;Q8SNB3;Q861A9;Q7YNX9;Q6E5A2;E0WMX5;E0WMX3;I0JGT8;H2DH14;B7UE87;Q2HQJ5;O19689;D5G1Q5;B4DJI3;D5G1Q8;Q4QYZ9;O19776;Q5D5C6;Q9TQK7;O19613;O19610;G8GIE9;X2KYQ5;W9AAG4;W5RED5;W5REC9;W5REB9;W5REB6;W5REB5;W0NU91;W0NTL0;W0NPW3;W0NPV2;W0HIR9;V6BNY6;V5JAE1;V5JAB8;V5J9Y1;U5IT58;U5IQH1;U3RCZ8;U3RCZ0;U3RCR2;U3RCP2;T1WFE4;T1WFC4;T1WEQ8;T1R3D6;T1R358;S5CJY0;S5CJX0;S5CFC8;S4W4Z9;S4T763;S4T6Z9;S4T6R5;S4T6N0;R4S802;R4QIM5;R4QIK0;R4N4E9;R4N0Q9;R4I3H4;Q9UQU5;Q9TQM5;Q9TQF7;Q9TQF6;Q9TPT3;Q9TPR7;Q9TPR1;Q9TPQ4;Q9TP34;Q9TP32;Q9TP27;Q9TP18;Q9MYG9;Q9MYE5;Q9MYD0;Q9MYB2;Q9MY88;Q9GJL9;Q9GJL8;Q9GJ35;Q9GJ29;Q9GJ21;Q9GIK9;Q9BCM4;Q95IZ7;Q95IZ6;Q95IZ2;Q95IG9;Q95IE0;Q95ID8;Q95ID5;Q95ID0;Q95IC9;Q95IC1;Q95378;Q8WMB3;Q8WLT5;Q8WLT4;Q8WLT3;Q8WLT2;Q8TE31;Q8SP54;Q8MHP5;Q8HWS4;Q8HWQ0;Q8HWP9;Q8HWP2;Q8HWN6;Q8HWM1;Q8HWG2;Q861B3;Q7YQ49;Q7YP76;Q7YP60;Q70BE5;Q702P5;Q6ZZX3;Q6ZZC1;Q6ZXX2;Q6TKG6;Q6KBB2;Q6KB21;Q6EZG0;Q5W1J8;Q5S3I7;Q5GMP9;Q5FZP7;Q5F2I5;Q5F2I4;Q56SH7;Q4QZ32;Q4A1C6;Q45QG4;Q38MU6;Q38MU3;Q2L4E8;Q2I0Z2;Q2HWF8;Q2HNR0;Q29739;Q29733;Q29724;Q29710;Q29699;Q27I57;Q27I56;Q209I3;Q1G101;Q19A31;Q0ZB68;Q0ZAX1;Q0ZAK8;Q0VZ44;Q0VKE5;Q0PQ79;Q0PQ41;Q0PQ38;Q0PHV4;Q09K10;Q05FZ4;P79500;O98012;O97997;O78183;O78182;O78141;O78096;O62920;N1JTZ2;N0AA34;N0A0J7;N0A0I9;M9Z6P5;M9XGG6;M9WQI3;M9PNW9;M9PNR1;M9PNN6;M9P9Z6;M9P9J5;M9P9I6;M9P9A2;M9P8K8;M9P8G7;M5DEW2;M4T5A7;M4STE7;M4SNY5;M4QC58;M4N8S5;M4MEW0;M4MAU0;M4M9J1;M4M812;M1FXE9;M1FWX7;M1F5Q1;L7XAA9;L7X807;L7X2Z2;L7UNT4;L7PH10;L7PFY2;L0HS95;L0HNX6;L0BXI1;L0BWY7;L0BW00;L0BVT6;L0BVP9;K9LC31;K9LC28;K9LC20;K7P602;K7P5R7;K7P5R6;K7P5R3;K4RGH3;K4JER4;K4JB02;K0DF06;J9ZY99;J9UMV1;J9UEN0;J9PWN4;J9PWL6;J9PVN6;J7K4Q8;J7K4Q3;J7K3G6;J7JGS9;J7FNZ6;J7FM84;J7FKX1;J7F7C2;J7F578;I7AY89;I7AY85;I7AY72;I7A4C2;I6ZTP9;I6TEY5;I6S3U2;I6RGW3;I6RCY1;I6RCV9;I6R700;I6QQS9;I6NXI5;I6NWH7;I6NN14;I6MHI2;I6MHG4;I6MHF8;I6M534;I3VZ28;I3VZ26;I3VZ06;I3UI68;I3QHR0;I2DAL3;I2B2Y8;I1ZAU6;I1W1M0;H9E8V6;H9E8V3;H9C5I3;H9C5H1;H9C5G1;H6V7R7;H6A2C4;H2DML6;H2DMK9;H2BEA2;H2BE90;H2BE82;H2BE76;H2BDQ7;H2BDP7;H2BDN6;G9I2L3;G9I2L0;G9HW87;G9HW32;G9HRL8;G9HRK6;G9FP30;G8GKQ6;G8GJB7;G8GIE1;G4XPL7;G4XFZ4;G4XFY9;G3DR79;G3DR71;G3D6I9;G1EQ53;G1EQ17;G1EPU8;G1EPS8;G1EPQ8;G1EPP4;G1EPN6;G1EPM9;G1EPL0;G1EPJ9;G1EPC8;G1EPB8;G1EP59;G1EP28;G1EP07;G1ENL2;G1ENL0;G1ENJ9;G1ENF4;G1ENE3;G1END8;G1ENA6;G1EMK2;G0ZMJ2;G0ZMI9;G0ZMI0;G0ZDT7;G0ZDT5;G0Z8C8;G0Z8B9;G0YW72;G0X8Q8;G0X8Q4;G0X8Q1;G0WVA8;G0WVA4;G0KXH8;F8SNU5;F8SKV2;F8RHG2;F8RHF0;F8RHC7;F8RHC6;F8R153;F8R113;F8R112;F6KRT8;F6KRS9;F6KRQ6;F6KRQ5;F6KRQ3;F6KRQ2;F6KRQ0;F6KRM8;F6KRM6;F6KRK5;F6KRK4;F6KRJ4;F5AVE7;F4YZU0;F4YU73;F4YU70;F4YU45;F4YU31;F4YU30;F4YU16;F2X641;F2X611;F2X607;F2X601;F2X5Z2;F2X5X2;F2X5U3;F2VP56;F2VNN3;F2VNM9;F2VNM7;F2VNL2;F2VNH5;F2VNF0;F2VND3;F2VNC9;F1CK26;F1AQP2;F1AQN4;F1AQN1;F1AQM0;E9LY50;E9LY37;E9LY22;E9LY19;E9LY18;E9LY17;E9LXY7;E8ZF54;E7BYB7;E7BYA9;E5G0X3;E5DCM9;E5DCM7;E5D6L5;E5D6K9;E5D6K5;E3T0W1;E3SWJ6;E3SWI2;E3SWH6;E3SWH5;E3SWG3;E3SWG0;E3SWF7;E3SG92;E3SG90;E3SG89;E3SG88;E2IBP1;E2GJM6;E2GJL2;E2GJK2;E2GJE5;E2DH99;E2DH98;E2D5S3;E2D5P4;E2D5P0;E2D5M4;E2D5M2;E2D5M0;E1U680;E0YTJ7;E0YTH9;E0X9K5;E0X602;E0WN67;E0WN65;E0WBZ6;E0WBZ5;E0WBZ0;E0WBU8;E0WBU7;E0WBU6;E0WBT8;E0WBT5;E0WBS8;E0WBS4;E0WBS0;D9U3H1;D7RJ42;D7R0V5;D7R0U7;D7R0U6;D7R0U2;D7NQV6;D7NQV2;D7NQU3;D7NQT4;D7NPP5;D7NP80;D7NNU4;D7NNQ7;D7GN25;D6MLN6;D6ML11;D6ML05;D6ML02;D6MJI7;D6MJH2;D6MJG7;D6MJG5;D6MJG4;D6MJF8;D6MJE6;D6MJC7;D6MJ84;D6C6G9;D6C6D0;D6C6C8;D6C6C2;D6C698;D6C526;D5M8G6;D5M8G5;D5M8D8;D5M8D3;D5M8C9;D5L9E2;D5L9C8;D5L9C5;D5G2L0;D5G2K7;D5FZV0;D5FZU2;D5FZJ8;D5FZJ6;D5FZI2;D5FWB3;D5FUE9;D5FI13;D5FHV9;D5FHV6;D5FHS1;D5FHJ6;D5FHG8;D5FHF2;D4HPL7;D3Y5Z0;D3Y5Y5;D3XDH2;D3XDG8;D3U7V2;D3U763;D3U4B4;D3U491;D3U485;D3U480;D3U407;D3U402;D3U3Z9;D3U3Y9;D3U3W8;D3U3T1;D3U3S7;D3U3R9;D3U3R7;D3U3R4;D3U3N3;D3U3M6;D3U3M2;D3G9J5;D3G9J3;D2XUQ9;D2U730;D2DL36;D2DKS7;D2DKR4;D1MEQ5;D0VE43;D0VE35;D0EP77;C9WEQ8;C9WCS6;C9EIW1;C9E857;C9E853;C9E845;C9E1E2;C9DSL1;C8XTN6;C8CJE2;C8CJC6;C8CJC3;C8CJB8;C8CJB5;C8CH79;C8CH65;C8CH61;C7FDU7;C7FDQ2;C7E593;C7E592;C7E571;C7E560;C6K4J8;C5J020;C5J018;C5IZS1;C5IZP0;C5IZM6;C5IZL3;C4PJK8;C3VIR5;C1KJL3;C1KBJ3;C0M143;C0M109;C0M108;C0LAB2;C0LAB0;C0LAA4;C0L075;C0KLS3;C0KLS0;C0KLR2;C0IY87;B8Y1Y0;B8Y1X9;B8Y1X5;B6VAX0;B6F252;B6DX34;B5MBS1;B5LZ17;B5ASL0;B4Z1E1;B3W6H1;B3V8R2;B2ZCW6;B2ZCW2;B2ZAW2;B2YG90;B2YG89;B2CYA3;B2CYA2;B2CY94;B2CS91;B1PMU8;B1PL03;A9YQA2;A9Y367;A8VYQ2;A8VYP1;A8E1W1;A8D9X9;A7X567;A5YVG4;A5YVG2;A5JSG7;A5JSG3;A5JSG2;A5JSF6;A5HKN6;A4ZW43;A4USH0;A4USG4;A3RKJ7;A3FG64;A3FG63;A3FG57;A3FG56;A2VBX8;A2TH17;A1Z288;A1Z0L5;A1Z0L2;A1YRJ1;A1EC34;A1DZT9;A0T4C1;A0PFV6;A0FKC6;A0FKC4;A0EVK0;Q8MGZ5;P79484;Q9TPS1;Q5D1X2;Q95HM4;Q95HM3;Q95HM2;Q95HM1;O78051;O78050;B6RC64;A9XFY1;Q9MY50;Q5EDD0;Q2HYG4;A7MAP3;H2AM06;A0SY06;X5LRQ8;Q9BCN1;Q29946;Q9MY72;B7Z760;E0WN59;B3KUD8;Q861F0;B4DT81;Q6DU28;Q6DU20;D0EV57;Q6ZUW0;Q54A71;B4DYG3;B4DQ99;Q29843;Q29929;Q29926;U5YEZ6;U5YEX7;U5YET1;U5YEH8;U5YEG4;U5YEF5;U5YED1;U5YEC2;U5YEC1;U5YEB8;U5YE97;U5YE94;U5YE56;U5YD21;U5YD07;U5YCY0;U5YCX5;U5YCF1;U5YCE5;U5YCE0;U5YCA1;U5YC96;U5YC70;U5YC53;U5YBX2;U5YBW4;U5YBM2;U5YBL1;U5YBK1;U5YBI4;U5YBI0;U5YBH7;U5YBG5;U5YBF6;U5YBE0;U5YBD5;U5YBD2;U5YEL0;U5YCQ3;V5NS99;V5NS88;V5NS77;V5NS67;V5NQU5;V5NQR8;V5NQR2;V5NQQ7;V5NQP8;V5NQB4;V5NQ91;V5NQ85;V5NQ80;V5NQ72;V5NQ55;V5NPR0;V5NPQ5;V5NPP7;V5NPP2;V5NPN7;V5NPN1;V5LET0;V5LER4;V5LEQ5;V5LDX8;V5LDU3;V5LDS6;V5LD58;V5LD40;I0BW60;V5LES4;H9C3J9;V5LLY0;V5LKY8;I0BW64;I0BW62;I0BW57;I0BW52;I0BW51;I0BW48;I0BW47;I0BW45;I0BW44;I0BW43;I0BW42;I0BW41;I0BW38;H9BYW0;H9BYV7;H9BYV6;H9BYV3;H9BYV1;H9BYU7;H9BYT7;H9BYT6;H9BYT4;H9BYT3;H9BYS7;H9BYR8;H9BYR6;H9BYR4;H9BYR2;V5LM11;V5LLA6;V5LLA1;V5LL83;V5LL32;V5LL19;V5LL10;V5LKW9;V5LKV1;V5LKI1;V5LKH1;Q30196;U5YEW6;U5YEU5;U5YEU0;U5YET7;U5YER4;U5YER3;U5YEQ2;U5YEP8;U5YCU4;U5YCT9;U5YCT5;U5YCT0;U5YCS5;U5YCQ8;U5YC29;U5YC25;U5YC21;U5YC02;U5YBZ6;U5YBZ2;U5YBY7;U5YBY3;U5YBU5;U5YBT9;U5YBT4;U5YBS1;Q30210;V5L7D7;V5L6C4;V5L695;V5L689;V5L684;V5L5Z3;V5L5Y9;V5L5Y0;V5L5L2;V5L5K8;Q30193;O19525;Q29930;V5L7H3;V5L6C5;V5L5N4;Q9MY64;H9C3K5;H9C3K3;H9C3K2;H9C3K1;H9C3K0;H9C3J6;H9C3J4;H9C3J3;H9C3I9;H9C3I2;V5NXV0;V5NW11;V5NVX8;V5NVQ3;V5NVM6;H9C3I6;S5U2K1;Q9UQU1;Q9UQT4;Q9UBX9;Q9GJ36;Q9BD22;Q8HWP4;Q860R4;Q7YQ97;Q7YP27;Q7YP19;Q70P03;Q70P00;Q2Z271;Q2Z195;Q2MGW4;P79487;O95957;O95732;O19636;O19576;O19559;D1MYV8;C7DRR9;B3G4T8;B2BD81;A8ILK2;A8CKI4;A7XFV8;A7L5M0;A5HC28;A1E126;M9TK49;U6EGC9;U6EFM4;Q9Y4H6;Q9UQT5;Q9UQS7;Q9UM39;Q9BD23;Q861C3;Q860R5;Q7YQ96;Q712M6;Q70T15;Q6H8L7;Q5K2Q5;Q5F312;Q2Z1V9;Q2MGW5;Q19KI9;P79593;O19570;O19516;O19511;H6SG22;F6KTD6;D6R3X5;D1MYW0;D1MYV9;C7FGZ4;B8XRE9;B5AN67;B4XAM4;A8UHA5;A8D714;A7U8S2;A3RLM7;A3FFS7;A3FFS6;A2TEM7;A1E2C1;E9NHA5;Q9UM27;F5A552;F4NAR7;B3GFZ6;D2U546;B3V8R7;A9X166;A8E0Y4;O19682;D0EYG4;B2LRS6;I2GUL0;O78209;Q95IZ0;D5G1Q6;I4EP44;Q5QQ12;C0JFR3;Q4F7G7;B4DV09;B4DEC0;Q95364;O19773;O19771;O19770;Q9TQE7;K7YQA9;W0NT80;W0NT76;W0HE80;U5IQN3;U3RHF3;U3RF87;T1R351;S5CGQ8;S5CFB8;R4ZGN3;R4N2L8;Q9TQH2;Q9TQG4;Q9TQG2;Q9TQF0;Q9MYD4;Q9MYC1;Q9MY44;Q9BD39;Q95J01;Q8WLS8;Q8HWT4;Q8HWP0;Q6H8L9;Q6GYF7;Q5U7L2;Q56GX9;Q29701;Q0ZP94;Q09K12;Q006A9;O97996;O78125;O19623;O19597;N0A0Q9;M9PNV1;M9P8Z5;M4NBV4;M1F5P9;L7X764;L0BXP5;K9LC16;K9L8E4;K9L7Y9;K7WRF3;K7P566;K7P520;J9PWV8;J9PW46;J7K7E6;J7K3F5;J7JXN6;J7JLT0;I6RGX2;I6NS34;I1W1M4;G9HRL7;G1EPT0;G1EPR2;G1EPQ6;G1EPJ7;G1EMK7;G0ZDV2;G0Z8D4;F8R1A1;F8R177;F6KRS3;F6KRS0;F6KRQ8;F6KRP9;F2VP79;F2VNN4;F1CCR9;F1CCR6;F1C3G7;F1AQM7;E9LY32;E9LY26;E5G0Z5;E5DCN4;E2GJN1;E0WBU1;D7R0V8;D7NPN2;D7NPM3;D6MLN7;D5M8D2;D5M8B7;D5G2K5;D5FZT7;D5FZT0;D5FHW6;D5FHU7;D4IH41;D3XDG7;D3XDF8;D3U4A8;D3U3Z1;D3U3T4;D3U3K9;D2SSK7;D2DL35;D1G107;D1G0U0;C9WCV4;C9WCT4;C8CH83;C7E596;C7E590;C7E582;C6K4J9;B6E323;B5AYU6;B2CYA5;B2CY97;A9JNR9;A8YQI3;A7WPI6;A4GX62;A1E2T0;A0A023T4T9;A0A023T393;X5KIQ6;W0HIJ3;V6AW53;U3PPK4;S4TZE5;R9QZS1;Q75ND3;Q2PZA0;N0A2P5;M9XL05;M9P9X7;M1FYE1;M1FXT2;L0BXQ7;K7YQA4;J7F652;I6NWH5;I4EP45;I3QHP5;H6UV78;H6A2F6;G9HWA4;G9HW81;G9HRL6;G1EQ62;G1EQ28;G1ENP9;G1ENH1;G0ZMF8;G0KXH0;F8URE2;F8RHG3;F8R148;F6KS46;F6KS41;F6KRX5;F4YZT6;F4YU14;F2X673;F2VNG1;E9LT29;E9LT17;E7EF84;E5DCN3;E2GJL1;E2DH89;E2D5T3;E0YTH3;E0X9K0;D7NPP2;D5M8C6;D4P2Y1;D4HPC3;D2DKG9;D1MDP2;D1FXH2;D0EP79;C9WCV0;C7FDR0;C0M139;B2ZCW5;A8VYR0;A3FG66;A3FG59;A0A023T4U2;Q6ZZC0;O78079;O78168;B3W4E3;B3W4E2;P79485;B3KQW1;O19633;H0Y842;Q31611;P30511-2;E9PGX5;U3MU75;S5DHZ7;J7RPN4;E0WMT7;P30511;F6IQC9;F6IQC8;Q30171;C7U1K3;P30511-3 | HLA class I histocompatibility antigen, A-1 alpha chain;HLA class I histocompatibility antigen, A-36 alpha chain;HLA class I histocompatibility antigen, A-3 alpha chain;HLA class I histocompatibility antigen, A-11 alpha chain | HLA-A;HLA-A*01;HLA-A null;HLA | 14 | 3 | 57.9 | 31.505 | 26.78 | 26.57 | 26.76 | 25.98 | 26.29 | 25.94 | 0.63 | 1.55 | 0.00834 |
| B3KMP6;O94903;D3DSW3;E5RFX7;A8K520;H0YBG2;E5RG77 | Proline synthase co-transcribed bacterial homolog protein | PROSC | 4 | 4 | 21.7 | 25.937 | 23.58 | 23.40 | 23.49 | 22.88 | 22.65 | 23.07 | 0.63 | 1.55 | 0.00895 |
| C9JG97;Q13685;C9JEH3;H7C0R2;C9JTS3;Q59EM1 | Angio-associated migratory cell protein | AAMP | 4 | 4 | 13.3 | 44.693 | 22.31 | 22.78 | 22.59 | 21.89 | 22.27 | 21.64 | 0.63 | 1.54 | 0.05234 |
| B2R5P6;Q16881-5;E2QRB9;Q16881-2;B7Z2S5;Q16881-4;E9PNQ6;Q16881-3;E7EW10;Q16881-6;A0A024RBK9;Q16881;Q16881-7;E9PIR7;E7ESI6;Q6ZR44;E9PKD3;E9PIZ5;E9PLT3;E9PKI4;E9PQI3;E9PRI8;E9PJU2 | Thioredoxin reductase 1, cytoplasmic | TXNRD1 | 36 | 35 | 75.7 | 54.546 | 30.31 | 30.42 | 30.53 | 29.50 | 30.00 | 29.89 | 0.63 | 1.54 | 0.01920 |
| Q53HF3;P06280 | Alpha-galactosidase A | GLA | 2 | 2 | 6.8 | 48.752 | 21.14 | 21.75 | 21.83 | 20.88 | 20.96 | 21.01 | 0.62 | 1.54 | 0.04814 |
| P35237;A0A024QZX5;Q8IXH2;A0A024QZT8 | Serpin B6 | SERPINB6 | 23 | 22 | 70.7 | 42.621 | 28.98 | 29.03 | 29.08 | 28.33 | 28.43 | 28.47 | 0.62 | 1.54 | 0.00027 |
| Q92747;E9PF58;Q53GB6;B4DLQ7;Q59GQ0;Q75MY0;F8WFD3 | Actin-related protein 2/3 complex subunit 1A | ARPC1A | 5 | 5 | 16.5 | 41.569 | 23.99 | 23.88 | 23.73 | 23.44 | 23.27 | 23.03 | 0.62 | 1.54 | 0.01133 |
| Q658J0;Q9GZT8-2;Q9GZT8;E7EXA3;Q6X734;Q9GZT8-3;B8ZZI0;C9JN42 | NIF3-like protein 1 | DKFZp762L015;NIF3L1 | 9 | 9 | 36.7 | 37.029 | 26.19 | 26.36 | 26.53 | 25.52 | 26.00 | 25.71 | 0.62 | 1.54 | 0.02220 |
| Q9UHY7;D6RA00;Q7Z508;Q9UHY7-2 | Enolase-phosphatase E1 | ENOPH1 | 8 | 8 | 39.1 | 28.932 | 24.83 | 24.79 | 25.10 | 23.72 | 24.40 | 24.73 | 0.62 | 1.54 | 0.11917 |
| O95834;O95834-2;O95834-3;K7EIK7;B7Z2F5;C9JRL6;B7Z872;B7Z918;K7EKG3;K7EKU5;K7EII6;K7ERY9;K7EIM1;K7ELI8;K7EQR0;K7ELB2;K7ERR9;K7ERR2 | Echinoderm microtubule-associated protein-like 2 | EML2 | 20 | 20 | 42.5 | 70.678 | 26.12 | 25.99 | 26.21 | 25.71 | 25.28 | 25.48 | 0.61 | 1.53 | 0.01183 |
| P27695;G3V3M6;G3V5Q1;G3V3C7;G3V5M0;G3V359;G3V5D9;H7C4A8;G3V3Y6;G3V2D9 | DNA-(apurinic or apyrimidinic site) lyase;DNA-(apurinic or apyrimidinic site) lyase, mitochondrial | APEX1 | 26 | 26 | 73.3 | 35.554 | 29.09 | 29.15 | 29.25 | 28.46 | 28.69 | 28.52 | 0.61 | 1.52 | 0.00207 |
| Q6NVY1;Q6NVY1-2;B9A058;B8ZZZ0;H7C126;H7C400;F8W8A6;H7BYI7 | 3-hydroxyisobutyryl-CoA hydrolase, mitochondrial | HIBCH | 9 | 9 | 23.8 | 43.482 | 25.54 | 25.55 | 24.99 | 24.75 | 24.67 | 24.85 | 0.61 | 1.52 | 0.03507 |
| O75436;F5H4L7;B4DLT1;S4R3Q6;O75436-2;G3V1N8;A8K3C1;S4R2Y3 | Vacuolar protein sorting-associated protein 26A | VPS26A | 10 | 9 | 40.7 | 38.169 | 25.47 | 24.89 | 25.40 | 24.55 | 24.78 | 24.62 | 0.60 | 1.52 | 0.03608 |
| O75191;B4DDT2;Q6PIS3;B3KM56 | Xylulose kinase | XYLB | 3 | 3 | 8.8 | 58.382 | 21.95 | 21.81 | 22.35 | 21.39 | 21.35 | 21.55 | 0.60 | 1.52 | 0.02480 |
| P06753-2;P06753-3;B2RDE1;P06753-6;P06753-5;P06753-4;Q5VU59;Q5HYB6;Q5VU61;J3KN67;M1VPF4;D6R904;B4DWT5;P06753;Q8NAG3;Q8NAH0;B4DQ80;K7EP68;P06753-7;Q5VU62;D6RFM2;Q6LDX7;Q8NI98;A0A024R4K5 | Tropomyosin alpha-3 chain;Tyrosine-protein kinase receptor | TPM3;DKFZp686J1372;TPM3-ROS1 | 18 | 18 | 43.1 | 29.032 | 27.62 | 27.54 | 27.63 | 27.40 | 26.77 | 26.81 | 0.60 | 1.52 | 0.04321 |
| Q9H1E3;Q9H1E3-2;B4DTY3;Q6IA16;Q8WYF8 | Nuclear ubiquitous casein and cyclin-dependent kinase substrate 1 | NUCKS1;NUCKS | 6 | 6 | 34.6 | 27.296 | 24.64 | 24.93 | 24.63 | 24.38 | 24.24 | 23.79 | 0.60 | 1.51 | 0.04250 |
| Q16531;F5GY55;B7Z2A1;B4DSA8;B4DZP5;B4DG00;F5H581;F5H2L3;F5H4N9;F5H6C5;F5GWI0;F5GZY8;F5H0Y5;F5H775;F5H238;B7Z859;F5GYG8;F8WF81;F5H7A0;F5GZ34;F5H198 | DNA damage-binding protein 1 | DDB1 | 46 | 46 | 45.5 | 126.97 | 28.62 | 28.68 | 28.89 | 28.09 | 28.12 | 28.21 | 0.60 | 1.51 | 0.00270 |
| O75884;O75884-2 | Putative hydrolase RBBP9 | RBBP9 | 6 | 6 | 45.7 | 21 | 24.38 | 24.93 | 25.10 | 24.30 | 24.19 | 24.14 | 0.59 | 1.51 | 0.05635 |
| P04075;J3KPS3;P04075-2;H3BQN4;H3BPS8;H3BUH7;H3BR04;H3BMQ8;H3BU78;H3BR68;A4UCS9;A4UCT0 | Fructose-bisphosphate aldolase A;Fructose-bisphosphate aldolase | ALDOA | 33 | 30 | 89 | 39.42 | 31.29 | 31.33 | 31.56 | 30.73 | 30.76 | 30.92 | 0.59 | 1.51 | 0.00473 |
| P61160;P61160-2;F5H6T1;Q8IY98;B4DWQ5;B4DHK9 | Actin-related protein 2 | ACTR2 | 12 | 12 | 40.4 | 44.76 | 25.54 | 25.75 | 25.75 | 25.20 | 25.13 | 24.94 | 0.59 | 1.50 | 0.00510 |
| Q99436;Q6IBT1;B2RAQ9;Q86U62;Q5TBG5;B4E0P1 | Proteasome subunit beta type-7;Proteasome subunit beta type | PSMB7 | 12 | 12 | 63.9 | 29.965 | 28.20 | 28.38 | 28.59 | 27.78 | 27.92 | 27.71 | 0.58 | 1.50 | 0.00979 |
| P47813;O14602;X6RAC9;A6NJH9 | Eukaryotic translation initiation factor 1A, X-chromosomal;Eukaryotic translation initiation factor 1A, Y-chromosomal | EIF1AX;EIF1AY | 5 | 5 | 36.8 | 16.46 | 23.49 | 23.35 | 23.54 | 22.75 | 23.04 | 22.84 | 0.58 | 1.50 | 0.00454 |
| Q9P2E9-3;Q9P2E9-2;Q9P2E9;A7BI36;V9GY78;A1A5C4;A2A2S5;Q05DK7;Q32NB1 | Ribosome-binding protein 1 | RRBP1 | 3 | 3 | 2.8 | 108.63 | 21.47 | 21.63 | 21.01 | 21.35 | 21.27 | 19.75 | 0.58 | 1.50 | 0.35400 |
| P53680-2;P53680;M0R0N4;X6R390;M0QYZ2;M0QZ21 | AP-2 complex subunit sigma | AP2S1 | 2 | 2 | 18.3 | 12.417 | 21.44 | 21.72 | 21.46 | 21.01 | 20.82 | 21.05 | 0.58 | 1.49 | 0.00729 |
| P31946;B5BU24;Q4VY20;Q4VY19;Q59EQ2;Q3YBA8 | 14-3-3 protein beta/alpha;14-3-3 protein beta/alpha, N-terminally processed | YWHAB | 11 | 2 | 47.6 | 28.082 | 29.24 | 29.18 | 29.13 | 28.71 | 28.54 | 28.57 | 0.58 | 1.49 | 0.00068 |
| P25786;P25786-2;V9HW55;F5GX11;B4E0X6;B4DEV8;F5H112 | Proteasome subunit alpha type-1 | PSMA1 | 23 | 23 | 83.3 | 29.555 | 30.45 | 30.68 | 30.78 | 30.09 | 30.17 | 29.93 | 0.57 | 1.49 | 0.00864 |
| P01877;P01876;Q9UP60;Q9NPP6;Q7Z379;Q6MZV6;Q6P089;Q6MZX9;Q6ZVX0;Q7Z374;Q8NCL6;Q96K68;Q6ZW64;Q96DK0;Q8WY24;Q6N090;Q6MZW0;Q6N092;Q6N041;Q6N091 | Ig alpha-2 chain C region;Ig alpha-1 chain C region | IGHA2;IGHA1;SNC73;DKFZp686K04218;DKFZp686L19235;IGH@;DKFZp686M08189;DKFZp686C02218;DKFZp686G21220;DKFZp686J11235;DKFZp686K18196;DKFZp686O16217;DKFZp686C02220 | 3 | 3 | 17.4 | 36.526 | 18.20 | 17.92 | 23.58 | 18.86 | 18.97 | 20.17 | 0.57 | 1.48 | 0.77791 |
| Q8NBS9-2;Q86UY0;Q6EHZ3;Q658S9;Q8NBS9 | Thioredoxin domain-containing protein 5 | TXNDC5;STRF8;DKFZp666I134 | 3 | 3 | 13.9 | 36.177 | 20.83 | 20.76 | 20.44 | 20.28 | 19.14 | 20.91 | 0.57 | 1.48 | 0.34560 |
| P13693;J3KPG2;Q5W0H4;P13693-2;B4DKJ4;E9PJF7;Q9NXW1;H0YCX0;Q9UP43;B4DLD6;Q56UQ5;Q86YH5;Q9HAU6 | Translationally-controlled tumor protein | TPT1 | 10 | 10 | 62.8 | 19.595 | 27.99 | 28.06 | 28.12 | 27.57 | 27.37 | 27.52 | 0.57 | 1.48 | 0.00130 |
| Q53FB0;O00299 | Chloride intracellular channel protein 1 | CLIC1 | 16 | 16 | 68.9 | 27.015 | 27.39 | 27.55 | 27.50 | 26.70 | 26.87 | 27.17 | 0.57 | 1.48 | 0.01749 |
| Q96G03;F5H6V2;B4DN40;B4E0G8;E7ENQ8;Q4W5D6;E9PD70;H0Y921 | Phosphoglucomutase-2 | PGM2 | 26 | 26 | 46.7 | 68.283 | 27.02 | 26.99 | 27.06 | 26.51 | 26.41 | 26.46 | 0.56 | 1.48 | 0.00011 |
| P13797;B4DPW9;Q53GY0;B7Z6M1;B4DGB4;F8W8D8;Q96HI1;B4DG31;B4DI60;A7E2S2;U3KQI3;B4DPU6;H7C4N2;Q59GX5;V9HWJ7;Q53FI1;P13796;Q5TBN3;B3KUI1;F2Z2Z9 | Plastin-3 | PLS3 | 38 | 35 | 70 | 70.81 | 28.52 | 28.44 | 28.36 | 27.94 | 27.91 | 27.78 | 0.56 | 1.48 | 0.00112 |
| P46934-4;A0A024R5S9;H0Y8H4;Q96PU5-3;H0Y8X6;Q96PU5-2;Q96PU5-6;Q96PU5-5;Q96PU5-7;Q96PU5;P46934-3;P46934-2;P46934 | E3 ubiquitin-protein ligase NEDD4;E3 ubiquitin-protein ligase NEDD4-like;E3 ubiquitin-protein ligase | NEDD4;NEDD4L | 2 | 2 | 3 | 104.22 | 27.72 | 27.08 | 27.58 | 27.20 | 26.68 | 26.83 | 0.56 | 1.47 | 0.08982 |
| Q9NZM1-4;Q9NZM1-7;Q9NZM1-5;Q9NZM1-6;Q9NZM1-3;C9JCN0;Q9NZM1;F8W8J4;Q9NZM1-8;Q9NZM1-2 | Myoferlin | MYOF | 4 | 4 | 32.5 | 17.135 | 22.85 | 22.72 | 22.81 | 22.10 | 22.87 | 21.74 | 0.56 | 1.47 | 0.17362 |
| Q9UBQ7;U3KQ56;Q5M7Z5;Q5T946 | Glyoxylate reductase/hydroxypyruvate reductase | GRHPR | 5 | 5 | 28 | 35.668 | 23.54 | 23.82 | 24.24 | 23.05 | 23.30 | 23.59 | 0.55 | 1.47 | 0.09745 |
| Q96HC4;A0A024RDE8;Q96HC4-3;F5H0X8;Q96HC4-2;D3YTJ1;Q96HC4-7;B7Z8X5;Q96HC4-4;Q96HC4-6;Q4W5K9;D6RAA1;D6RGG6;H0Y929;Q96HC4-5;H0YBI4 | PDZ and LIM domain protein 5 | PDLIM5;LIM | 4 | 4 | 9.6 | 63.944 | 24.88 | 24.87 | 24.60 | 24.22 | 24.16 | 24.30 | 0.55 | 1.47 | 0.00482 |
| Q53FT8;P20618;Q59GN1 | Proteasome subunit beta type;Proteasome subunit beta type-1 | PSMB1 | 20 | 20 | 73 | 26.491 | 28.99 | 29.10 | 29.20 | 28.44 | 28.71 | 28.48 | 0.55 | 1.47 | 0.00582 |
| O60701-2;O60701;D6RHF4;Q9NY20;E9PBD2;E7ER95;E7ER83;E7ETF4;Q9NQ81;E7EV97;O60701-3 | UDP-glucose 6-dehydrogenase | UGDH;ugd;GDH | 2 | 2 | 5.4 | 47.602 | 20.57 | 21.42 | 19.93 | 20.48 | 19.88 | 19.90 | 0.55 | 1.47 | 0.31049 |
| Q9UBG0 | C-type mannose receptor 2 | MRC2 | 5 | 5 | 4.9 | 166.67 | 21.42 | 21.02 | 21.70 | 20.84 | 20.31 | 21.34 | 0.55 | 1.46 | 0.19884 |
| Q8N1Q1 | Carbonic anhydrase 13 | CA13 | 2 | 2 | 10.7 | 29.443 | 21.26 | 21.96 | 21.28 | 21.08 | 20.84 | 20.93 | 0.55 | 1.46 | 0.08466 |
| Q8TDQ7-3;Q8TDQ7;Q8TDQ7-2;Q8TDQ7-5;Q8TDQ7-4;V9GYK3;V9GYW0 | Glucosamine-6-phosphate isomerase 2 | GNPDA2 | 6 | 6 | 42.9 | 29.173 | 23.88 | 23.77 | 23.59 | 23.47 | 23.26 | 22.87 | 0.54 | 1.46 | 0.04906 |
| B4E1R7;Q59EF6;P17655-2;B4DN77;P17655;Q6ZVC6;B3KUH9 | Calpain-2 catalytic subunit | CAPN2 | 5 | 1 | 8.4 | 60.507 | 22.68 | 22.30 | 22.70 | 22.18 | 21.93 | 21.94 | 0.54 | 1.46 | 0.02500 |
| Q53H82 | Beta-lactamase-like protein 2 | LACTB2 | 10 | 10 | 40.3 | 32.805 | 24.08 | 23.96 | 24.20 | 23.62 | 23.36 | 23.63 | 0.54 | 1.46 | 0.00872 |
| Q9BYT8;A8K9T8;B3KU23;E9PCB6;Q9BQD0;Q96K48;H0YAK4;Q8NDD9;H0YAF7 | Neurolysin, mitochondrial | NLN | 17 | 17 | 25.3 | 80.651 | 25.62 | 25.67 | 25.78 | 24.86 | 25.20 | 25.38 | 0.54 | 1.45 | 0.02649 |
| Q5D1D5;B4E1L5;Q59H47;H3BNS1;Q9H0R5-4;Q9H0R5 |  |  | 2 | 2 | 3.4 | 67.916 | 21.73 | 21.29 | 20.53 | 20.83 | 20.71 | 20.39 | 0.54 | 1.45 | 0.22517 |
| D6R967;H0Y9D8;B4DR66;E2QRM6;Q9H2U2;Q9H2U2-2;B4DFH3;Q9H2U2-6;A8K0P2;Q9H2U2-3;D6RGV9 | Inorganic pyrophosphatase 2, mitochondrial | PPA2 | 3 | 3 | 16.8 | 21.39 | 22.99 | 23.39 | 22.76 | 22.54 | 22.50 | 22.49 | 0.53 | 1.45 | 0.04533 |
| B4DMA2;P08238;Q6PK50;B4DGL0;O14942;Q5T9W8;F8W026;Q58FF7;Q14568;Q9H6X9;A8K3W9;Q58FF6 | Heat shock protein HSP 90-beta | HSP90AB1 | 8 | 4 | 17.5 | 79.194 | 23.65 | 23.72 | 23.47 | 23.18 | 23.25 | 22.81 | 0.53 | 1.44 | 0.02740 |
| Q9BRL5;P62158;B2RDW0;H0Y7A7;E7ETZ0;E7EMB3;G3V361;Q96HY3;A8K1M2;F8WBR5;M0QZ52;G3V479;G3V226;B4DUI9;C9J7T9;P27482;P02585 | Calmodulin | CALM1;CALM2 | 9 | 9 | 46.3 | 16.506 | 27.23 | 26.92 | 27.39 | 27.16 | 26.70 | 26.09 | 0.53 | 1.44 | 0.19400 |
| P37802;P37802-2;X6RJP6;B7Z5A2;C9J5W6;Q9UI15 | Transgelin-2 | TAGLN2 | 13 | 13 | 71.4 | 22.391 | 27.07 | 26.60 | 26.34 | 26.58 | 26.16 | 25.69 | 0.53 | 1.44 | 0.19021 |
| Q9UJ70;Q9UJ70-2;H7C3G9;C9JEV6;H7C286;E9PPU6;H7C1L7;H0YEB7;H0YE82;H0YC94;H0YF44;Q6ZMB4 | N-acetyl-D-glucosamine kinase | NAGK | 10 | 10 | 34.3 | 37.375 | 24.60 | 24.47 | 24.91 | 24.07 | 24.11 | 24.24 | 0.52 | 1.44 | 0.02060 |
| P37837;F2Z393;B4DID5;Q9UMF8;E9PM01;E9PKI8 | Transaldolase | TALDO1 | 29 | 29 | 70.3 | 37.54 | 30.85 | 30.86 | 30.96 | 30.21 | 30.48 | 30.42 | 0.52 | 1.44 | 0.00410 |
| P07195;A8MW50;C9J7H8;F5H793;Q59G78 | L-lactate dehydrogenase B chain;L-lactate dehydrogenase | LDHB | 23 | 23 | 66.5 | 36.638 | 31.34 | 31.28 | 31.63 | 30.73 | 31.00 | 30.97 | 0.52 | 1.44 | 0.01986 |
| P17174;Q2TU84;B7Z1I2;B7Z7E9 | Aspartate aminotransferase, cytoplasmic;Aspartate aminotransferase | GOT1;GIG18 | 30 | 30 | 86.7 | 46.247 | 29.71 | 29.93 | 30.09 | 29.18 | 29.64 | 29.34 | 0.52 | 1.44 | 0.04125 |
| B7Z5S9;O94973;O94973-2;O94973-3;B7Z1Q4;Q9UFK5 | AP-2 complex subunit alpha-2 | AP2A2 | 2 | 2 | 3.5 | 103.06 | 19.93 | 19.76 | 19.64 | 18.13 | 19.71 | 19.96 | 0.51 | 1.43 | 0.42708 |
| H3BPK3;Q16775-2;B4DT01;Q16775;H3BPQ4;H3BQW8;Q16775-3;H3BV79 | Hydroxyacylglutathione hydrolase, mitochondrial | HAGH | 7 | 7 | 38.9 | 26.305 | 26.06 | 26.40 | 26.53 | 26.07 | 25.86 | 25.54 | 0.51 | 1.42 | 0.07035 |
| O75874;B2R5M8;V9HW78;Q6FI37;Q0QER2;Q6FIA4;B4DXS4;C9J4N6;C9JJE5;C9JLU6;Q9H302 | Isocitrate dehydrogenase [NADP] cytoplasmic;Isocitrate dehydrogenase [NADP] | IDH1 | 22 | 21 | 65.2 | 46.659 | 28.38 | 28.51 | 28.71 | 27.86 | 28.08 | 28.13 | 0.51 | 1.42 | 0.01646 |
| P63208;E5RJR5;E5RGM3;E7ERH2;F8W8N3;P63208-2 | S-phase kinase-associated protein 1 | SKP1 | 2 | 2 | 16 | 18.658 | 21.63 | 21.40 | 21.01 | 21.09 | 20.48 | 20.95 | 0.51 | 1.42 | 0.12014 |
| Q6YP21-3;Q6YP21;Q6YP21-2 | Kynurenine--oxoglutarate transaminase 3 | CCBL2 | 12 | 12 | 35 | 47.752 | 24.94 | 24.97 | 25.10 | 24.56 | 24.37 | 24.57 | 0.51 | 1.42 | 0.00352 |
| Q9UN71-2;Q9UN71;Q9Y5G3-2;Q3SY74;Q9Y5G2-2;Q9Y5G1-2;Q9Y5G3;Q9Y5G1;Q9Y5G2 | Protocadherin gamma-B4;Protocadherin gamma-B1;Protocadherin gamma-B2;Protocadherin gamma-B3 | PCDHGB4;PCDHGB1;PCDHGB2;PCDHGB3 | 2 | 2 | 2.6 | 87.446 | 21.77 | 22.01 | 22.47 | 20.39 | 22.06 | 22.28 | 0.50 | 1.42 | 0.46814 |
| P61158;B4DXW1;F5H3P5;Q59FV6;B4DT29;B4DTI0;B4E1U3;Q59GD5;B7Z9W3;Q9P1U1-2;Q9P1U1;B3KM55;Q9P1U1-3;F8WEW2;F8WE84;H7C4J1;Q9C0K3;C9IZN3 | Actin-related protein 3 | ACTR3 | 22 | 22 | 70.6 | 47.371 | 27.23 | 27.22 | 26.99 | 26.72 | 26.69 | 26.53 | 0.50 | 1.42 | 0.00649 |
| P09960;B4DVZ8;P09960-4;P09960-2;P09960-3;Q59ES1;Q49AK0;B4DEH5 | Leukotriene A-4 hydrolase | LTA4H | 31 | 31 | 63.5 | 69.284 | 28.12 | 28.36 | 28.45 | 27.68 | 28.04 | 27.72 | 0.50 | 1.42 | 0.02902 |
| Q14974;B2RBR9;Q14974-2;F5H4R7;B7Z5M1;J3KTM9;B7Z752;J3QR48;J3QKQ5 | Importin subunit beta-1 | KPNB1 | 14 | 14 | 21.5 | 97.169 | 26.09 | 25.92 | 25.67 | 25.63 | 25.00 | 25.55 | 0.50 | 1.41 | 0.09951 |
| B4DNY3;B4DNW7;B4DI38;D3DPU2;Q01518-2;Q01518;B2RDY9;B4DNA3;B4DUZ8;Q5T0R7;Q5T0R6;Q5T0R5;Q5T0R4;Q5T0R3;Q5T0R2;Q5T0R1;Q5T0R9;B7Z214;B7Z385;Q5JPJ8;B7Z1C4;E9PDI2;B7Z4M3;P40123 | Adenylyl cyclase-associated protein;Adenylyl cyclase-associated protein 1 | CAP1 | 7 | 7 | 23.9 | 43.706 | 24.16 | 23.27 | 23.81 | 23.24 | 23.26 | 23.26 | 0.50 | 1.41 | 0.13041 |
| Q15084-3;Q15084;Q15084-4;Q15084-5;Q15084-2 | Protein disulfide-isomerase A6 | PDIA6 | 4 | 4 | 13.7 | 47.837 | 22.40 | 22.54 | 22.34 | 21.99 | 22.04 | 21.75 | 0.50 | 1.41 | 0.01038 |
| Q5T013;F6UJY1;H0YB70;Q5T013-4;Q5T015;J9JIE9;Q5T013-3;E7EWH8;H0YB18;Q5T013-2;F6UJY9;G4XUV3;Q5T017 | Putative hydroxypyruvate isomerase;Hydroxypyruvate isomerase | HYI | 6 | 6 | 36.8 | 30.405 | 23.33 | 23.59 | 23.60 | 22.80 | 23.08 | 23.16 | 0.49 | 1.41 | 0.02475 |
| O00468-6;O00468-3;O00468-2;O00468-5;O00468-4;O00468-7;O00468;H0Y5U1 | Agrin;Agrin N-terminal 110 kDa subunit;Agrin C-terminal 110 kDa subunit;Agrin C-terminal 90 kDa fragment;Agrin C-terminal 22 kDa fragment | AGRN | 7 | 7 | 4.5 | 214.84 | 27.47 | 27.58 | 27.95 | 27.11 | 27.11 | 27.30 | 0.49 | 1.41 | 0.03522 |
| Q13813-3;A0A024R889;Q13813;Q13813-2;A6NG51;Q9UG16;B4DGT1;B4DTV8 | Spectrin alpha chain, non-erythrocytic 1 | SPTAN1;DKFZp564P0562 | 12 | 12 | 6.5 | 282.28 | 25.38 | 24.64 | 24.95 | 24.55 | 24.19 | 24.75 | 0.49 | 1.41 | 0.14349 |
| Q9ULV4;Q53G58;B3KN06;Q9ULV4-2;Q59EA2;Q9ULV4-3;B4DMH3;B4E3S0;B7Z9V0;F8W1H8;F8VUX3;F8VSA4;F8VRE9;H0YHL7;F8VTT6;F8VVB7;F8VV53 | Coronin-1C;Coronin | CORO1C | 13 | 13 | 33.8 | 53.248 | 26.39 | 26.48 | 26.02 | 25.92 | 25.87 | 25.63 | 0.49 | 1.40 | 0.04220 |
| P56537;B7ZBH1;P56537-2 | Eukaryotic translation initiation factor 6 | EIF6 | 4 | 4 | 31.4 | 26.599 | 25.31 | 25.11 | 24.93 | 24.84 | 24.41 | 24.63 | 0.49 | 1.40 | 0.04155 |
| Q6P179-3;Q6P179;B2R769;D6RGW0;H0Y9X9;H0YAL8;Q6P179-4;Q6P179-2 | Endoplasmic reticulum aminopeptidase 2 | ERAP2 | 8 | 8 | 10.9 | 105.52 | 23.36 | 23.48 | 23.34 | 22.92 | 22.84 | 22.95 | 0.49 | 1.40 | 0.00078 |
| P46108-2;P46108;I3L297 | Adapter molecule crk | CRK | 8 | 8 | 64.2 | 22.905 | 24.07 | 24.00 | 24.14 | 23.65 | 23.40 | 23.70 | 0.49 | 1.40 | 0.00822 |
| B7Z4B7;B7Z6U8;Q53FI7;Q13642-1;Q13642-5;Q13642-4;Q5JXI8;B7Z5T3;B7Z5V0;Q13642;Q13642-3;Q5JXH8;Q5JXH7;Q5JXI3;Q5JXI2;B7Z9A1;Q5JXH9;Q5JXI0;B3KRI9 | Four and a half LIM domains protein 1 | FHL1 | 7 | 7 | 35.5 | 28.097 | 24.38 | 23.32 | 23.84 | 23.50 | 23.54 | 23.04 | 0.49 | 1.40 | 0.23326 |
| P59998;F8WCF6;P59998-3;F8WDD7;H7C0A3;P59998-2;Q9H7Z5;R4GN08;P59998-4;F8WDW3;F8WE39 | Actin-related protein 2/3 complex subunit 4 | ARPC4;ARPC4-TTLL3 | 7 | 7 | 53 | 19.667 | 26.44 | 26.12 | 26.08 | 25.64 | 26.02 | 25.54 | 0.48 | 1.40 | 0.06130 |
| P40925;P40925-3;P40925-2;B9A041;C9JF79;B8ZZ51;C9JRL4;C9JLV6;F8WFC2;C9IZI0 | Malate dehydrogenase, cytoplasmic;Malate dehydrogenase | MDH1 | 17 | 17 | 54.5 | 36.426 | 30.05 | 29.97 | 30.09 | 29.39 | 29.54 | 29.76 | 0.48 | 1.39 | 0.01335 |
| Q6QN92;Q6IAT2;Q53XL7;P23434;H3BNV1 | Glycine cleavage system H protein, mitochondrial | GCSH | 3 | 3 | 42.4 | 13.813 | 23.57 | 23.00 | 23.47 | 22.44 | 23.07 | 23.10 | 0.48 | 1.39 | 0.15911 |
| Q5VWZ2-2;Q5VWZ2;B3KVW3 | Lysophospholipase-like protein 1 | LYPLAL1 | 2 | 2 | 11.8 | 24.476 | 20.27 | 19.25 | 19.22 | 19.64 | 18.77 | 18.91 | 0.48 | 1.39 | 0.33601 |
| P09382;F8WEI7 | Galectin-1 | LGALS1 | 10 | 10 | 86.7 | 14.716 | 29.02 | 28.86 | 29.05 | 28.28 | 28.57 | 28.65 | 0.48 | 1.39 | 0.01857 |
| P62805;Q0VAS5;Q6B823 | Histone H4 | HIST1H4A;HIST1H4H | 9 | 9 | 59.2 | 11.367 | 28.21 | 27.57 | 27.57 | 27.05 | 27.36 | 27.50 | 0.48 | 1.39 | 0.13127 |
| P49721;B7Z478;Q59FJ0 | Proteasome subunit beta type-2;Proteasome subunit beta type | PSMB2 | 20 | 20 | 91.5 | 22.836 | 28.96 | 28.83 | 28.86 | 28.35 | 28.60 | 28.28 | 0.48 | 1.39 | 0.00993 |
| P31939;B2R7P8;P31939-2;F5GWY2;B4DP06;H7C1S2;C9JLK0;H7C084;H7BZU3;F8WEF0 | Bifunctional purine biosynthesis protein PURH;Phosphoribosylaminoimidazolecarboxamide formyltransferase;IMP cyclohydrolase | ATIC | 31 | 31 | 62.8 | 64.615 | 26.97 | 27.02 | 27.24 | 26.23 | 26.74 | 26.83 | 0.47 | 1.39 | 0.08147 |
| P00390-2;P00390;P00390-3;P00390-4;P00390-5;H0YBD4;E5RI06;H0YC68;Q03504 | Glutathione reductase, mitochondrial | GSR | 20 | 20 | 60.8 | 51.7 | 28.33 | 28.45 | 28.38 | 27.68 | 28.08 | 27.99 | 0.47 | 1.39 | 0.01977 |
| B3KUZ8;P00505;A0A024R6W0;P00505-2;A8K482;B4DY39 | Aspartate aminotransferase;Aspartate aminotransferase, mitochondrial | GOT2 | 29 | 29 | 86 | 41.326 | 30.10 | 30.17 | 30.38 | 29.42 | 29.90 | 29.91 | 0.47 | 1.39 | 0.06101 |
| Q01995;Q53GC9;Q6FI52;H0YCU9;E9PJ32;Q59FA5;Q96FG7;B0YJ58 | Transgelin | TAGLN | 18 | 18 | 83.6 | 22.611 | 27.96 | 27.72 | 27.95 | 27.80 | 27.34 | 27.09 | 0.47 | 1.38 | 0.10114 |
| P30086;B4DRT4 | Phosphatidylethanolamine-binding protein 1;Hippocampal cholinergic neurostimulating peptide | PEBP1 | 15 | 9 | 82.4 | 21.057 | 30.46 | 30.36 | 30.55 | 29.77 | 30.11 | 30.08 | 0.47 | 1.38 | 0.01884 |
| Q03154;B4DNW0;Q03154-3;Q03154-4;C9JMV9;B4DPC3;Q03154-2;F8WC59;C9JYZ0 | Aminoacylase-1 | ACY1;ABHD14A-ACY1 | 15 | 15 | 40.4 | 45.884 | 26.34 | 26.18 | 26.28 | 25.62 | 25.94 | 25.86 | 0.46 | 1.38 | 0.01158 |
| Q14019;H3BT58 | Coactosin-like protein | COTL1 | 17 | 17 | 80.3 | 15.945 | 29.17 | 29.09 | 29.24 | 28.86 | 28.47 | 28.79 | 0.46 | 1.38 | 0.02203 |
| Q9H0W9-3;Q9H0W9-2;Q9H0W9;A8K718;E9PQS1;E9PIP1;E9PJU8;Q9H0W9-4;E9PR95;E9PSC3;E9PLC5;E9PLB3;E9PPB5 | Ester hydrolase C11orf54 | C11orf54 | 7 | 7 | 35.8 | 29.491 | 24.37 | 24.70 | 24.41 | 23.66 | 24.25 | 24.19 | 0.46 | 1.37 | 0.10071 |
| Q9Y5Z4;Q05DB4;Q9Y5Z4-2;Q5THN1;C9IZA0 | Heme-binding protein 2 | HEBP2 | 6 | 6 | 32.2 | 22.875 | 25.05 | 24.56 | 24.02 | 24.55 | 24.04 | 23.66 | 0.46 | 1.37 | 0.31020 |
| Q99880;Q99879;Q99877;Q93079;Q5QNW6;Q0D2M2;P62807;P58876;P57053;O60814;A8K9J7;Q5QNW6-2;I6L9F7;U3KQK0;B4DR52;Q96A08;L0R4T3 | Histone H2B type 1-L;Histone H2B type 1-M;Histone H2B type 1-N;Histone H2B type 1-H;Histone H2B type 2-F;Histone H2B type 1-C/E/F/G/I;Histone H2B type 1-D;Histone H2B type F-S;Histone H2B type 1-K;Histone H2B;Histone H2B type 1-A | HIST1H2BL;HIST1H2BM;HIST1H2BN;HIST1H2BH;HIST2H2BF;HIST1H2BC;HIST1H2BD;H2BFS;HIST1H2BK;HIST1H2BA | 6 | 2 | 41.3 | 13.952 | 27.99 | 27.31 | 27.17 | 26.61 | 27.15 | 27.33 | 0.46 | 1.37 | 0.24055 |
| Q53EM5;P29401;P29401-2;B4E022;B3KSI4;B4DVU1;B3KPZ8;E9PFF2;F8W888;Q6MZE3;F8WAX4 | Transketolase | TKT | 30 | 30 | 59.7 | 67.905 | 29.19 | 29.35 | 29.28 | 28.67 | 28.84 | 28.94 | 0.46 | 1.37 | 0.00703 |
| P26038;Q6PJT4;B7Z4C7;Q6PKD3;Q05CU6;P35241;P35241-5;V9HW42;Q6NUR7;P15311;B2R6J2;J7M2B1;E7EQR4;P35241-4;B7Z2S7;V9GZ54;Q9UJZ6;Q9UK20;B7Z437;B7Z5V2;Q9UJZ8;Q9UJU1;Q9UJZ2;Q9UJZ7;E9PQ82;E9PNV3;P35241-3;E9PNP4 | Moesin | MSN | 44 | 44 | 70.4 | 67.819 | 30.19 | 29.88 | 29.74 | 29.64 | 29.39 | 29.42 | 0.45 | 1.37 | 0.04310 |
| Q99836-4;B4DT48;B4DU08;Q99836-3;Q99836-2;Q99836-5;Q99836;Q99836-6;H0Y4G9 | Myeloid differentiation primary response protein MyD88 | MYD88 | 2 | 2 | 17.8 | 15.823 | 18.74 | 21.08 | 20.10 | 18.75 | 19.69 | 20.12 | 0.45 | 1.37 | 0.59685 |
| O00203-3;O00203;A8K586;H0YBD0;B4E0B2;Q3MNE1;Q13367-3;Q13367;Q13367-4;Q68CN5 | AP-3 complex subunit beta-1;AP-3 complex subunit beta-2 | AP3B1;AP3B2;DKFZp686D17136 | 2 | 2 | 2.5 | 116.19 | 25.11 | 24.75 | 24.27 | 24.18 | 22.24 | 26.35 | 0.45 | 1.37 | 0.72804 |
| B3KR00;Q96FJ0;B3KS69;Q96FJ0-2 | AMSH-like protease | STAMBPL1 | 3 | 3 | 12.2 | 30.186 | 22.04 | 21.87 | 22.27 | 21.32 | 21.91 | 21.59 | 0.45 | 1.37 | 0.09209 |
| P09936;B2RD14;D6RE83;D6R956;A6NLJ7;D6R974;D6RF53 | Ubiquitin carboxyl-terminal hydrolase isozyme L1 | UCHL1 | 19 | 19 | 92.8 | 24.824 | 30.26 | 30.50 | 30.38 | 29.86 | 29.90 | 30.04 | 0.45 | 1.36 | 0.00722 |
| O75368;D3DTE6;B0AZV6 | SH3 domain-binding glutamic acid-rich-like protein | SH3BGRL | 12 | 12 | 85.1 | 12.774 | 27.02 | 27.03 | 27.20 | 26.53 | 26.77 | 26.61 | 0.45 | 1.36 | 0.00828 |
| B4DN60;O43776 | Asparagine--tRNA ligase, cytoplasmic | NARS | 5 | 5 | 20.1 | 34.921 | 21.81 | 21.98 | 21.90 | 21.85 | 20.87 | 21.66 | 0.44 | 1.35 | 0.22574 |
| Q59ET7;Q6M1B7 |  |  | 13 | 2 | 32.3 | 55.217 | 25.64 | 25.95 | 26.14 | 25.12 | 25.95 | 25.37 | 0.43 | 1.35 | 0.20416 |
| H0YLA2;P37108 | Signal recognition particle 14 kDa protein | SRP14 | 2 | 2 | 23.5 | 13.058 | 21.36 | 21.30 | 20.87 | 20.77 | 20.61 | 20.87 | 0.43 | 1.34 | 0.06747 |
| P06733;E2DRY6;P06733-2;K7EM90;A4UCS8;A4QMW8;L0R849;E5RI09;E5RG95;K7EPM1;K7EKN2;E5RGZ4;K7ERS8;B4DUJ6 | Alpha-enolase;Enolase | ENO1 | 43 | 39 | 84.1 | 47.168 | 32.57 | 32.56 | 32.52 | 31.88 | 32.25 | 32.24 | 0.43 | 1.34 | 0.02441 |
| Q16773;B7Z4W5;Q16773-2;Q5T278;Q16773-3;Q5T277;Q5T276;B3KSW6;Q9UQN7 | Kynurenine--oxoglutarate transaminase 1 | CCBL1 | 6 | 6 | 22.7 | 47.875 | 23.37 | 23.73 | 23.37 | 23.01 | 22.96 | 23.23 | 0.43 | 1.34 | 0.04544 |
| O95831-3;O95831;Q5RZ99;Q5RZA0;O95831-6;O95831-4;O95831-2;O95831-5;E9PMA0 | Apoptosis-inducing factor 1, mitochondrial | AIFM1 | 6 | 6 | 12.8 | 66.294 | 24.16 | 23.77 | 23.45 | 23.58 | 23.24 | 23.30 | 0.42 | 1.34 | 0.14333 |
| P31150;B4DHX4;B4DH24;B4E070;G5E9U5 | Rab GDP dissociation inhibitor alpha | GDI1 | 18 | 18 | 46.1 | 50.582 | 27.48 | 27.54 | 27.72 | 27.14 | 27.21 | 27.13 | 0.42 | 1.33 | 0.00522 |
| H0YC23;B3KQ51;Q8WZ56;Q6I9T8;Q5U0I7;P67775;P62714;B3KRM2;P67775-2 | Serine/threonine-protein phosphatase;Serine/threonine-protein phosphatase 2A catalytic subunit alpha isoform;Serine/threonine-protein phosphatase 2A catalytic subunit beta isoform | PPP2CB;PPP2CA | 2 | 2 | 31.5 | 13.87 | 21.13 | 20.69 | 20.92 | 20.65 | 20.42 | 20.42 | 0.41 | 1.33 | 0.04993 |
| P00441;H7BYH4;X5D5A9;W8Q444;A1YYW4;X5D3G4;X5CHU1 | Superoxide dismutase [Cu-Zn] | SOD1 | 10 | 9 | 86.4 | 15.936 | 28.99 | 29.14 | 29.30 | 28.47 | 28.84 | 28.88 | 0.41 | 1.33 | 0.05700 |
| F5H345;P08397-2;P08397;F5GY90;F5H226;P08397-4;P08397-3;F5H0P4;Q6LER4;F5H4W5;F5H4X2;Q0G847;Q68Y91 | Porphobilinogen deaminase | HMBS | 6 | 6 | 24.5 | 35.761 | 24.27 | 22.97 | 23.32 | 22.83 | 23.22 | 23.27 | 0.41 | 1.33 | 0.37456 |
| Q9H299;Q86Z22;D3DPK5;Q5T123 | SH3 domain-binding glutamic acid-rich-like protein 3 | SH3BGRL3 | 6 | 6 | 67.7 | 10.438 | 29.98 | 29.90 | 30.58 | 29.72 | 30.02 | 29.49 | 0.41 | 1.33 | 0.19169 |
| X5D7K9;Q14764;B4DXN0;B4DDR2;B4DP93;X5D7S8;X5DNU0;H3BQK6;X5DP17;X5D7R7;X5DP24;I3L155;H3BUK7;H3BNF6;H3BRL2 | Major vault protein | MVP | 9 | 9 | 12.1 | 91.958 | 22.64 | 22.61 | 21.66 | 20.96 | 22.07 | 22.68 | 0.40 | 1.32 | 0.53954 |
| Q9H7C9;E9PNP3;E9PR47;Q9H7C9-3;E9PIQ4;E9PJP1;Q9H7C9-2;E9PLK9;K4DI89 | Mth938 domain-containing protein | AAMDC | 3 | 3 | 25.4 | 13.332 | 22.94 | 23.26 | 23.69 | 22.88 | 22.92 | 22.88 | 0.40 | 1.32 | 0.13899 |
| Q16836;E9PF18;B3KTT6;Q16836-3;Q16836-2;B2RB06 | Hydroxyacyl-coenzyme A dehydrogenase, mitochondrial | HADH | 10 | 10 | 41.4 | 34.293 | 26.85 | 26.93 | 26.97 | 26.39 | 26.71 | 26.45 | 0.40 | 1.32 | 0.01892 |
| P30740;B4E3A8;B4DNT0 | Leukocyte elastase inhibitor | SERPINB1 | 18 | 17 | 45.9 | 42.741 | 28.01 | 28.04 | 27.96 | 27.50 | 27.67 | 27.65 | 0.40 | 1.32 | 0.00228 |
| B4DFL1;P09622;B4DT69;E9PEX6;B4DMK9;B4DHG0;F8WDM5;F2Z2E3 | Dihydrolipoyl dehydrogenase;Dihydrolipoyl dehydrogenase, mitochondrial | DLD | 23 | 23 | 61.9 | 48.899 | 28.98 | 29.28 | 29.21 | 28.50 | 28.87 | 28.93 | 0.39 | 1.31 | 0.07431 |
| P25787;Q53GF5;C9JCK5;Q6MZI6;H3BT36;H7C402 | Proteasome subunit alpha type-2;Proteasome subunit alpha type | PSMA2 | 14 | 14 | 60.3 | 25.898 | 29.46 | 29.45 | 28.96 | 28.74 | 29.17 | 28.79 | 0.39 | 1.31 | 0.14205 |
| Q13867;K7ES02;E7EMN3;B4DXF3;K7ESE8;J3KSD8;J3KS79;K7ENH5;K7EMJ3 | Bleomycin hydrolase | BLMH | 13 | 13 | 35.4 | 52.562 | 26.02 | 26.13 | 26.23 | 25.34 | 25.92 | 25.96 | 0.39 | 1.31 | 0.13892 |
| Q96TA1-2;Q96TA1;Q9H8K1;Q2YD88 | Niban-like protein 1 | FAM129B | 5 | 5 | 9.3 | 82.682 | 23.01 | 22.28 | 21.07 | 22.00 | 21.68 | 21.52 | 0.39 | 1.31 | 0.54343 |
| Q16658;J3KNT0;B3KTA3;B3KTM9;C9JFC0;C9JPH9 | Fascin | FSCN1 | 30 | 30 | 73.8 | 54.529 | 30.25 | 30.45 | 30.75 | 29.91 | 30.24 | 30.15 | 0.39 | 1.31 | 0.09290 |
| P62258;G9K389;G9K388;P62258-2;B4DJF2;I3L3T1;B4DJB0;B7ZA86;K7EIT4;K7EM20;I3L0W5;B3KT37 | 14-3-3 protein epsilon | YWHAE;YWHAE/FAM22B fusion;YWHAE/FAM22A fusion | 27 | 25 | 80 | 29.174 | 31.64 | 31.63 | 31.55 | 31.25 | 31.25 | 31.19 | 0.38 | 1.30 | 0.00035 |
| Q5TZP0;Q53G96;Q53FK3;P03956;B4DN15;Q53G75;Q53G97;B4DW26;Q53G95;Q96DZ4;A5GZ69 | Interstitial collagenase;22 kDa interstitial collagenase;27 kDa interstitial collagenase | MMP1 | 31 | 31 | 60.6 | 53.981 | 31.91 | 31.68 | 31.65 | 31.52 | 31.20 | 31.38 | 0.38 | 1.30 | 0.03881 |
| Q96GK7;C9JGM0;C9J5B6;B4DHE4 | Fumarylacetoacetate hydrolase domain-containing protein 2A | FAHD2A | 5 | 3 | 23.9 | 34.596 | 22.48 | 22.22 | 22.74 | 22.00 | 22.17 | 22.13 | 0.38 | 1.30 | 0.07430 |
| Q5CAQ5;P14625;Q59FC6;B4DQX3;Q96GW1;B4DHT9;B4DU71 | Endoplasmin | TRA1;HSP90B1 | 5 | 5 | 9.5 | 92.339 | 22.73 | 22.78 | 22.86 | 22.21 | 22.57 | 22.48 | 0.37 | 1.29 | 0.03259 |
| P06748-2;P06748;P06748-3;A4ZU86;Q9BTI9;E5RI98;Q9NX34;E5RGW4;A0A024R3V9;V9HVU7;A0A024RAP1 | Nucleophosmin | NPM1 | 9 | 9 | 40.8 | 29.464 | 26.33 | 26.54 | 25.86 | 26.44 | 25.24 | 25.93 | 0.37 | 1.29 | 0.41022 |
| P48163;A8K168;B4DZ70;F5H4W0;B4DX99;Q8WVX2 | NADP-dependent malic enzyme;Malic enzyme | ME1 | 17 | 17 | 34.6 | 64.149 | 26.91 | 26.90 | 26.90 | 26.33 | 26.67 | 26.61 | 0.37 | 1.29 | 0.02681 |
| Q13404;Q13404-8;Q13404-7;Q13404-2;Q13404-1;G3V2F7;Q13404-6;E5RIF1;D6RG00 | Ubiquitin-conjugating enzyme E2 variant 1 | UBE2V1;TMEM189 | 6 | 2 | 36.1 | 16.495 | 25.90 | 25.92 | 25.63 | 25.69 | 25.49 | 25.16 | 0.37 | 1.29 | 0.11354 |
| C9JR52;P21246 | Pleiotrophin | PTN | 3 | 3 | 30.1 | 18.877 | 24.12 | 24.07 | 24.75 | 24.22 | 23.64 | 23.97 | 0.36 | 1.29 | 0.25766 |
| P05556;P05556-2;P05556-5;P05556-4;P05556-3;H7C4N8;C9JPK5;Q5T3E6;E9PLR6;E7EUI6;E7EQW5;E7ERX5 | Integrin beta-1 | ITGB1 | 11 | 11 | 15.7 | 88.414 | 24.80 | 24.73 | 24.92 | 24.27 | 24.61 | 24.48 | 0.36 | 1.28 | 0.03410 |
| H7C2G3;P30042;P30042-2;H7C1F6;F2Z2Q0;H7BYH1 | ES1 protein homolog, mitochondrial | C21orf33 | 8 | 8 | 44 | 20.183 | 25.97 | 26.16 | 26.33 | 25.55 | 26.00 | 25.84 | 0.36 | 1.28 | 0.10437 |
| Q08209-2;Q08209;Q08209-4;F5H0F8;B7Z781;E7ETC2;Q08209-5;Q08209-3;Q5F2F8;P16298-2;P16298-3;P16298;P16298-4;E9PPC8;E9PK68;Q9UMM5;Q9UMB2;B7Z6P2;Q5F2G0;H0YC26;P48454-2;P48454;P48454-3 | Serine/threonine-protein phosphatase 2B catalytic subunit alpha isoform;Serine/threonine-protein phosphatase;Serine/threonine-protein phosphatase 2B catalytic subunit beta isoform | PPP3CA;PPP3CB | 4 | 4 | 7.6 | 57.658 | 22.62 | 22.43 | 22.06 | 22.04 | 21.99 | 22.01 | 0.36 | 1.28 | 0.09846 |
| P15374 | Ubiquitin carboxyl-terminal hydrolase isozyme L3 | UCHL3 | 2 | 2 | 15.7 | 26.182 | 22.48 | 22.78 | 22.74 | 22.65 | 22.18 | 22.11 | 0.35 | 1.28 | 0.14370 |
| P01024;B4E216;M0QYC8;M0QXZ3;B4DR57;M0R0Q9;M0R1Q1 | Complement C3;Complement C3 beta chain;Complement C3 alpha chain;C3a anaphylatoxin;Acylation stimulating protein;Complement C3b alpha chain;Complement C3c alpha chain fragment 1;Complement C3dg fragment;Complement C3g fragment;Complement C3d fragment;Complement C3f fragment;Complement C3c alpha chain fragment 2 | C3 | 86 | 77 | 55.5 | 187.15 | 29.55 | 29.39 | 29.44 | 29.14 | 29.03 | 29.15 | 0.35 | 1.27 | 0.00472 |
| Q99497;K7ELW0;K7EN27 | Protein DJ-1 | PARK7 | 22 | 22 | 89.4 | 19.891 | 30.00 | 30.08 | 30.04 | 29.49 | 29.95 | 29.63 | 0.35 | 1.27 | 0.06393 |
| P60981;P60981-2;F6RFD5;B7Z9M9 | Destrin | DSTN | 10 | 10 | 68.5 | 18.506 | 25.50 | 25.53 | 25.66 | 25.40 | 25.18 | 25.06 | 0.35 | 1.27 | 0.03467 |
| P28074;P28074-3;H0YJM8;P28074-2 | Proteasome subunit beta type-5 | PSMB5 | 13 | 13 | 54.8 | 28.48 | 29.08 | 29.31 | 29.53 | 28.94 | 29.17 | 28.77 | 0.35 | 1.27 | 0.11861 |
| P13798;C9JIF9;H7C393;H7C1U0;C9JLK2;F8WEH5;H0YFE5 | Acylamino-acid-releasing enzyme | APEH | 24 | 24 | 49.9 | 81.224 | 27.85 | 28.04 | 28.28 | 27.43 | 27.93 | 27.77 | 0.35 | 1.27 | 0.14836 |
| B4DJV2;O75390;B3KTN4;Q0QEL2;H0YIC4;F8VPA1;B7Z1E1;F8VPF9;F8VRI6;F8W1S4;H0YH82;F8VRP1;F8VX68;F8VR34;F8W4S1;F8VZK9;F8VX07;F8VWQ5;F8VTT8;F8W642;F8W0J2;F8VU34 | Citrate synthase;Citrate synthase, mitochondrial | CS | 19 | 18 | 46.8 | 50.431 | 28.15 | 28.41 | 28.43 | 27.88 | 28.12 | 27.96 | 0.34 | 1.27 | 0.03964 |
| Q53GN4;Q59ER5;O75083;Q53H17;B3KXN4;O75083-3;B4DY05;D6RD66;B4DS71;B4DZX5;Q9BZT4 | WD repeat-containing protein 1 | WDR1 | 36 | 36 | 73.4 | 66.179 | 30.22 | 30.30 | 30.50 | 29.85 | 30.10 | 30.04 | 0.34 | 1.27 | 0.03755 |
| P15144;Q59E93;B4DV63;B4DP01;B4DP96;B4DPH5;H0YKT6;H0YLZ8;H0YMC1;Q8IVL7;Q71E46 | Aminopeptidase N | ANPEP | 45 | 45 | 45.3 | 109.54 | 29.81 | 29.82 | 29.98 | 29.30 | 29.76 | 29.53 | 0.34 | 1.27 | 0.07618 |
| Q9Y281;Q9Y281-3;F8WDN3;G3V2U0 | Cofilin-2 | CFL2 | 7 | 7 | 50.6 | 18.736 | 25.84 | 25.73 | 25.88 | 25.59 | 25.42 | 25.42 | 0.34 | 1.27 | 0.00907 |
| O95302;B3KQQ0;B7Z230;B7Z1U8;O95302-3;C9J4P8;O95302-2 | Peptidyl-prolyl cis-trans isomerase FKBP9 | FKBP9 | 3 | 3 | 6.1 | 63.083 | 21.27 | 21.04 | 21.12 | 21.08 | 20.70 | 20.64 | 0.34 | 1.26 | 0.09367 |
| B7Z9I3;B7Z4S4;O75787;H0Y750;H7C3E1;B7Z1I9;B7Z413;H7C240;B7Z487 | Renin receptor | ATP6AP2 | 4 | 4 | 17 | 35.612 | 23.48 | 23.17 | 23.30 | 22.44 | 23.24 | 23.27 | 0.34 | 1.26 | 0.30519 |
| Q96C23;B8ZZ75;F8WB82;H7C320;Q12915;H7C1B5 | Aldose 1-epimerase | GALM | 11 | 11 | 50.3 | 37.765 | 26.14 | 26.47 | 26.39 | 25.69 | 26.27 | 26.04 | 0.34 | 1.26 | 0.15901 |
| P08254;A5GZ70;H7C139;F5GYX7;P09238 | Stromelysin-1 | MMP3 | 7 | 7 | 14 | 53.977 | 24.25 | 23.94 | 24.34 | 24.39 | 23.63 | 23.53 | 0.32 | 1.25 | 0.33647 |
| P04181;P04181-2;Q59HE2 | Ornithine aminotransferase, mitochondrial;Ornithine aminotransferase, hepatic form;Ornithine aminotransferase, renal form | OAT | 12 | 12 | 33.9 | 48.534 | 24.04 | 24.25 | 24.40 | 23.58 | 24.15 | 23.99 | 0.32 | 1.25 | 0.17954 |
| P07954-2;P07954 | Fumarate hydratase, mitochondrial | FH | 23 | 23 | 62.7 | 50.212 | 28.49 | 28.51 | 28.86 | 28.10 | 28.46 | 28.34 | 0.32 | 1.25 | 0.11463 |
| Q9BV20-2;Q9BV20 | Methylthioribose-1-phosphate isomerase | MRI1 | 3 | 3 | 14 | 34.529 | 22.15 | 22.36 | 21.56 | 21.97 | 21.61 | 21.52 | 0.32 | 1.25 | 0.31366 |
| Q03393;E9PKY8;E9PJM0;E9PNN3 | 6-pyruvoyl tetrahydrobiopterin synthase | PTS | 2 | 2 | 12.4 | 16.386 | 22.00 | 22.12 | 22.34 | 21.63 | 21.90 | 21.96 | 0.32 | 1.25 | 0.09043 |
| A0A024R442;Q9ULA0;Q53SB6;E7ETB3;B7Z7F0;Q9NV55;Q9BSS9;E7EMB6;A0A024R443;Q9HAC6;B9ZVU2;F8WAN0;B7Z822;C9JBE1;E7EPX3;E5RJ35;C9JRG3;C9J1E2 | Aspartyl aminopeptidase | DNPEP | 10 | 10 | 26.3 | 51.998 | 24.91 | 24.99 | 25.04 | 24.69 | 24.69 | 24.62 | 0.32 | 1.25 | 0.00246 |
| Q8IV08;B4DEL6;M0QZI4;M0R1F7;M0R3G9;M0R2W7;E2QRG1 | Phospholipase D3 | PLD3 | 6 | 6 | 15.5 | 54.705 | 23.01 | 23.10 | 22.80 | 22.68 | 22.48 | 22.81 | 0.32 | 1.25 | 0.07145 |
| H0YD13;B4DN59;P16070-18;P16070-12;A8K309;P16070-14;P16070-13;P16070-11;P16070-10;P16070-16;P16070-8;P16070-17;P16070-6;P16070-4;P16070-3;P16070-7;E7EPC6;P16070-5;P16070;H0YDW7;H0YCV9;H0Y2P0;P16070-15;H0Y5E4;P16070-9;E9PKC6;H0YDX6;C1PHC3;Q86UZ1;P16070-19;H0YD17;C1PHC4;H0YE40;J3KN83;B4E2X0 | CD44 antigen | CD44 | 5 | 5 | 25.2 | 22.683 | 29.03 | 29.24 | 29.33 | 28.85 | 28.88 | 28.92 | 0.32 | 1.24 | 0.02704 |
| P63104;E7EX29;B0AZS6;E7ESK7;P63104-2;B7Z2E6;H0YB80;E9PD24;E7EVZ2;E5RIR4;Q6LD62;Q2F831;E5RGE1 | 14-3-3 protein zeta/delta | YWHAZ | 23 | 21 | 78.4 | 27.745 | 31.34 | 31.45 | 31.37 | 31.16 | 31.14 | 30.90 | 0.31 | 1.24 | 0.02439 |
| H7C4F6;O00629 | Importin subunit alpha-3 | KPNA4 | 2 | 2 | 18.2 | 18.881 | 19.89 | 19.24 | 20.25 | 19.69 | 19.69 | 19.06 | 0.31 | 1.24 | 0.43336 |
| B7Z1V7;B7Z4V2;V9HW84;P38646;Q8N1C8;D6RA73;D6RJI2;B7Z4T3;H0YBG6;Q2F839;A1XP52 | Stress-70 protein, mitochondrial | HSPA9 | 6 | 6 | 17.4 | 47.362 | 24.43 | 24.43 | 24.45 | 24.42 | 24.25 | 23.71 | 0.31 | 1.24 | 0.22328 |
| G3V121;B7Z2L8;Q7Z5U5;Q86WR0;Q0VGD4 | Coiled-coil domain-containing protein 25 | CCDC25 | 2 | 2 | 25.6 | 9.9502 | 20.47 | 20.71 | 20.21 | 20.42 | 20.38 | 19.67 | 0.31 | 1.24 | 0.34118 |
| Q9H4A4;Q7RU04;A6NKB8;C9JMZ3;H7C2T3 | Aminopeptidase B | RNPEP | 38 | 38 | 65.4 | 72.595 | 28.55 | 28.71 | 28.82 | 28.24 | 28.46 | 28.47 | 0.30 | 1.23 | 0.04979 |
| B1AKQ8;C9JZN1;F6X3N5;F6UT28;C9JIS1;C9JXA5;E7EP32;P62873-2;P62879;P62873;B2R6K4;F5H8J8;F5H100;F5H0S8;P16520-2;Q59G26;E9PCP0;Q9HAV0;P16520;F1T0G4;C9JD14;A8K3F6 | Guanine nucleotide-binding protein G(I)/G(S)/G(T) subunit beta-1;Guanine nucleotide-binding protein G(I)/G(S)/G(T) subunit beta-2;Guanine nucleotide-binding protein G(I)/G(S)/G(T) subunit beta-3;Guanine nucleotide-binding protein subunit beta-4 | GNB1;GNB2;GNB3;GNB4 | 3 | 3 | 26.2 | 12.198 | 23.51 | 23.04 | 22.37 | 22.58 | 22.80 | 22.65 | 0.30 | 1.23 | 0.42514 |
| P43034;B4DZN3;B4DF38;I3L3N5;P43034-2;I3L495 | Platelet-activating factor acetylhydrolase IB subunit alpha | PAFAH1B1 | 13 | 13 | 34.4 | 46.637 | 25.66 | 25.35 | 25.58 | 25.55 | 25.11 | 25.05 | 0.29 | 1.23 | 0.18184 |
| D6RGE2;Q9Y3B5;Q96CN7 | Isochorismatase domain-containing protein 1 | ISOC1 | 3 | 3 | 21.3 | 20.239 | 23.02 | 22.61 | 22.85 | 22.39 | 22.61 | 22.58 | 0.29 | 1.23 | 0.10101 |
| P16083;A0A024QZT9;Q5TD07;B3KPX6;Q5TD05;Q59EN2;A2A2U4 | Ribosyldihydronicotinamide dehydrogenase [quinone] | NQO2 | 10 | 10 | 59.3 | 25.918 | 26.60 | 26.63 | 26.68 | 26.04 | 26.61 | 26.39 | 0.29 | 1.22 | 0.15592 |
| B4E1G2;Q5HYG8;P34897-3;Q53ET4;P34897;B4DJ63;B4DLV4;B4DW25;B4DP88;Q5BJF5;P34897-2;B4DWA7;G3V2Y4;H0YIZ0;B4DJQ3;G3V5L0;G3V241;G3V3Y8;G3V4T0;G3V4X0;G3V2W0;G3V2E4;G3V540;G3V4W5 | Serine hydroxymethyltransferase;Serine hydroxymethyltransferase, mitochondrial | DKFZp686P09201;SHMT2 | 7 | 7 | 20.1 | 49.261 | 23.88 | 24.43 | 24.71 | 23.78 | 24.12 | 24.24 | 0.29 | 1.22 | 0.35989 |
| P48061-2;P48061-5;P48061;P48061-6;P48061-3;P48061-4 | Stromal cell-derived factor 1;SDF-1-beta(3-72);SDF-1-alpha(3-67) | CXCL12 | 2 | 2 | 24.7 | 10.103 | 22.61 | 22.97 | 23.20 | 22.09 | 22.60 | 23.22 | 0.29 | 1.22 | 0.47589 |
| Q13011;M0R248;M0QZW4;B4DVS4;M0QXS7;M0R1A2;M0QYT0 | Delta(3,5)-Delta(2,4)-dienoyl-CoA isomerase, mitochondrial | ECH1 | 4 | 4 | 14 | 35.816 | 23.91 | 23.69 | 23.85 | 23.54 | 23.51 | 23.53 | 0.29 | 1.22 | 0.01258 |
| O43924;B8ZZK5;C9IZ52 | Retinal rod rhodopsin-sensitive cGMP 3,5-cyclic phosphodiesterase subunit delta | PDE6D | 3 | 3 | 28.7 | 17.42 | 23.57 | 23.86 | 23.38 | 23.40 | 23.17 | 23.38 | 0.29 | 1.22 | 0.14332 |
| A0A024RAR8;A8K6H1 |  |  | 26 | 1 | 33.7 | 107.2 | 26.11 | 26.03 | 26.10 | 25.73 | 25.79 | 25.86 | 0.28 | 1.22 | 0.00294 |
| P53999;Q59G24;Q6E433;B7Z1Z0 | Activated RNA polymerase II transcriptional coactivator p15 | SUB1 | 3 | 3 | 18.9 | 14.395 | 22.15 | 22.21 | 22.24 | 21.97 | 21.99 | 21.78 | 0.28 | 1.22 | 0.01710 |
| P48637;B7Z1C5;B7Z514;P48637-2 | Glutathione synthetase | GSS | 26 | 26 | 62.4 | 52.384 | 28.05 | 28.17 | 28.19 | 27.84 | 28.01 | 27.73 | 0.28 | 1.22 | 0.03800 |
| Q9NY33;G3V1D3;G3V180;Q9NY33-4;Q5JPB8;Q53HL4;Q53GT4;B4E357;Q9NY33-2;E9PQ14;E9PPK9;E9PKK8;E9PQF2;E9PNX5 | Dipeptidyl peptidase 3 | DPP3;DKFZp686O1117 | 40 | 40 | 72.5 | 82.588 | 29.37 | 29.45 | 29.12 | 28.88 | 29.15 | 29.08 | 0.28 | 1.21 | 0.09920 |
| P26885;F5H0N4 | Peptidyl-prolyl cis-trans isomerase FKBP2 | FKBP2 | 4 | 4 | 39.4 | 15.649 | 24.67 | 24.43 | 24.72 | 24.32 | 24.43 | 24.24 | 0.28 | 1.21 | 0.05921 |
| E9PLK3;P55786;B7Z899;B7Z4B2;B7Z463;B3KU93;B3KTP2;B7Z1H4;B7Z1V9;E5RJ24;E7EWZ2;I3L083;A6NEC2;B7Z6T6;F5GZY4;H0YAQ6;E9PP11;H0YDG0;A6NEC2-2;E9PJY4;E9PPD4;H0YCQ5;E9PJ74;A6NEC2-3;B4DGH6;E9PRQ5;E9PJF9;E9PPZ2;E9PI82 | Puromycin-sensitive aminopeptidase | NPEPPS | 46 | 46 | 59.9 | 102.99 | 29.96 | 30.01 | 30.10 | 29.45 | 29.87 | 29.93 | 0.27 | 1.21 | 0.15441 |
| B4DV28;Q96KP4;Q96KP4-2;A0A024R382;B4DPF1;J3QKT2;Q9NW02;J3KSV5;J3QQN6;J3QR27;J3QLU1;J3QKQ0;J3QRD0;J3QL02;Q9H7K8;J3QRA8;Q9NUV1;J3KSS4;J3KRD5;J3QRH4;J3QRP4;J3KRJ8 | Cytosolic non-specific dipeptidase | CNDP2 | 21 | 21 | 55.3 | 51.501 | 26.80 | 26.73 | 26.85 | 26.48 | 26.60 | 26.50 | 0.27 | 1.21 | 0.00661 |
| Q53G71;P27797;B4DHR1;K7EJB9;B4E2Y9;K7EL50 | Calreticulin | CALR | 28 | 28 | 80.8 | 46.918 | 31.70 | 31.96 | 32.07 | 31.48 | 31.76 | 31.70 | 0.27 | 1.20 | 0.12683 |
| P61981;B3KNB4;B4DHC4;B4DE78 | 14-3-3 protein gamma;14-3-3 protein gamma, N-terminally processed | YWHAG | 17 | 15 | 72.1 | 28.302 | 29.32 | 29.34 | 29.16 | 28.98 | 29.14 | 28.91 | 0.26 | 1.20 | 0.03928 |
| P23526;Q1RMG2;P23526-2 | Adenosylhomocysteinase | AHCY | 8 | 8 | 20.1 | 47.716 | 25.00 | 25.06 | 24.89 | 24.26 | 24.76 | 25.13 | 0.26 | 1.20 | 0.36398 |
| F6SBX2;A8K5W7;Q9NSE4 | Isoleucine--tRNA ligase, mitochondrial | IARS2 | 19 | 19 | 23.6 | 106.04 | 24.82 | 25.01 | 25.19 | 24.71 | 24.61 | 24.92 | 0.26 | 1.20 | 0.13957 |
| P07237;B4DNL5;B4DUA5;H7BZ94;F5H8J2;B4DLN6;I3L312;I3L398;H0Y3Z3;B4DJS0;Q96C96;B3KTQ9;I3L4M2;I3L0S0;I3NI03;I3L3P5;I3L3U6;B4DNP9;I3L514;I3L1Y5 | Protein disulfide-isomerase | P4HB | 42 | 42 | 70.7 | 57.116 | 30.57 | 30.81 | 30.80 | 30.46 | 30.54 | 30.40 | 0.26 | 1.20 | 0.04214 |
| H3BLV0;P08174-4;E9PSH2;P08174;P08174-3;P08174-5;P08174-2;B1AP13;P08174-6;P08174-7;Q8TD12;B2R658;Q8TD11;Q8TD13;B1AP15;B4DUF2;H7BY55;B4DPY4;Q8TD14 | Complement decay-accelerating factor | CD55 | 4 | 4 | 14.7 | 35.695 | 22.15 | 22.23 | 22.43 | 21.87 | 22.17 | 21.99 | 0.26 | 1.20 | 0.10170 |
| B3KQT9;G5EA52;P30101;B3KQT2;B4DJ98;B4DDM1 | Protein disulfide-isomerase A3 | PDIA3 | 43 | 36 | 73.1 | 54.102 | 30.80 | 30.95 | 30.86 | 30.47 | 30.68 | 30.69 | 0.26 | 1.19 | 0.04031 |
| H3BPM5;H3BNY8;H3BMZ9;Q8NHZ6;H3BPP3;H3BP57;H3BT48;H3BUZ9;B4DFC4;F5GX71;H3BPB8;P34949;H3BPU7;H3BN01;H3BQX0;H3BT46;B4DYB8;P34949-2;B4DW50;H3BU66 | Mannose-6-phosphate isomerase | MPI | 3 | 3 | 27.7 | 19.145 | 22.52 | 22.21 | 22.57 | 21.36 | 22.05 | 23.12 | 0.26 | 1.19 | 0.65150 |
| P31937;H7BZL2 | 3-hydroxyisobutyrate dehydrogenase, mitochondrial | HIBADH | 12 | 12 | 45.8 | 35.329 | 25.56 | 25.58 | 25.55 | 25.26 | 25.50 | 25.16 | 0.25 | 1.19 | 0.06580 |
| Q13641 | Trophoblast glycoprotein | TPBG | 4 | 4 | 10.2 | 46.031 | 22.81 | 22.55 | 22.70 | 22.06 | 22.96 | 22.29 | 0.25 | 1.19 | 0.41692 |
| P16930;Q53XA7;H0YLC7;B7Z4W2;H3BNP8 | Fumarylacetoacetase | FAH;DKFZp686F13224 | 19 | 19 | 62.1 | 46.374 | 28.29 | 28.22 | 28.14 | 27.78 | 28.18 | 27.96 | 0.24 | 1.18 | 0.12202 |
| P14618;P14618-3;B4DNK4;Q8WUW7;Q9UKK4;Q9NYI7;Q9UK31;Q9UN47 | Pyruvate kinase PKM;Pyruvate kinase | PKM;PKM2 | 48 | 5 | 79.5 | 57.936 | 31.08 | 31.12 | 31.24 | 30.67 | 30.94 | 31.11 | 0.24 | 1.18 | 0.15974 |
| P13639;Q8TA90;B4DPU3;Q6PK56;B4DRE8;Q6W6M8;B4DMC6;K7EJ74;K7EP67;Q8IXJ3;B4DZB1;Q15029-2;Q15029-3;B3KX19;Q6IBM8;Q15029;A8KAP3 | Elongation factor 2 | EEF2 | 21 | 21 | 29.8 | 95.337 | 25.39 | 25.31 | 25.31 | 24.78 | 24.77 | 25.75 | 0.24 | 1.18 | 0.50691 |
| P60174-1;P60174;Q53HE2;B4DUI5;P60174-4;U3KPZ0;U3KQF3;U3KPS5 | Triosephosphate isomerase | TPI1 | 29 | 11 | 94 | 26.669 | 33.63 | 33.68 | 33.88 | 33.22 | 33.75 | 33.51 | 0.23 | 1.18 | 0.24193 |
| Q60FE6;Q60FE5;P21333-2;P21333;Q5HY54;A6NDY9;Q8TES4;F8WE98;Q6NXF2;E9PHF0;B4E2F9;Q96C61;Q86TQ3;H0Y5C6;B4DTD5;H0Y5F3;Q2VP91 | Filamin-A | FLNA | 54 | 49 | 28.9 | 277.5 | 28.30 | 28.25 | 28.40 | 27.99 | 28.10 | 28.17 | 0.23 | 1.17 | 0.03183 |
| P10768;X6RA14;H7BZT7;U3KQT1 | S-formylglutathione hydrolase | ESD | 11 | 11 | 54.3 | 31.462 | 26.08 | 26.08 | 25.89 | 25.55 | 25.87 | 25.95 | 0.23 | 1.17 | 0.17445 |
| P35579;Q86XU5;P35579-2;Q6ZNL4;Q99529;B4E3S1;Q7Z7R0;REV__B4DQZ7;REV__S4R3H4;REV__E7EQT4;REV__Q9UKV3-5;REV__Q9UKV3;Q7Z406-5;Q7Z406-4;P35749-4;P35749-3;P35749;P35749-2;Q7Z406;Q7Z406-6;F2Z2U8;B1PS43;Q7Z406-2;G8JLL9 | Myosin-9 | MYH9 | 28 | 28 | 16.7 | 226.53 | 25.49 | 24.84 | 25.30 | 24.98 | 25.08 | 24.89 | 0.23 | 1.17 | 0.31903 |
| Q04446;E9PGM4 | 1,4-alpha-glucan-branching enzyme | GBE1 | 22 | 1 | 42 | 80.473 | 27.49 | 27.03 | 27.12 | 27.11 | 26.93 | 26.91 | 0.23 | 1.17 | 0.21710 |
| A6NMA8;O95825;H7C3S0;H7C338;C9K0F7;F8WF64;C9JZK8;B3KQ77;H7C3I5;A6NND8;A6NHJ8 | Quinone oxidoreductase-like protein 1 | CRYZL1 | 2 | 2 | 10.8 | 37.061 | 22.33 | 22.92 | 22.14 | 22.15 | 21.82 | 22.75 | 0.22 | 1.17 | 0.56789 |
| Q07955;J3KTL2;Q07955-3;Q59FA2;Q07955-2;A8K1L8;J3QQV5;J3KSW7;J3KSR8;B7Z570 | Serine/arginine-rich splicing factor 1 | SRSF1 | 9 | 9 | 34.3 | 27.744 | 25.21 | 24.95 | 25.04 | 24.68 | 25.17 | 24.69 | 0.22 | 1.17 | 0.27749 |
| Q13630;E9PKL9;B3KU96;E9PP14;E9PLH9;B4DZW9;E9PP60;H0YE90;H0YCP7 | GDP-L-fucose synthase | TSTA3 | 10 | 10 | 39.9 | 35.892 | 25.88 | 26.13 | 26.36 | 25.58 | 26.10 | 26.02 | 0.22 | 1.16 | 0.35799 |
| P14618-2;B4DRT3;H3BTN5;Q504U3;A0A024R609;H3BQ34;H3BUW1;H3BTJ2;H3BT25;H3BU13;H3BN34;H3BQZ3;B4DPM0;P30613-2;Q16716;P30613;Q16715;O75758 | Pyruvate kinase PKM;Pyruvate kinase | PKM;PKM2 | 4 | 4 | 5.8 | 58.061 | 25.45 | 25.72 | 25.62 | 25.29 | 25.31 | 25.53 | 0.22 | 1.16 | 0.11323 |
| Q9UM02;P48147;B2RAH7 | Prolyl endopeptidase | PREP | 12 | 12 | 22.5 | 80.733 | 23.89 | 24.21 | 24.20 | 23.43 | 24.12 | 24.10 | 0.22 | 1.16 | 0.43512 |
| P10809;B7Z597;B3GQS7;B7Z5E7;B7Z4F6;B7Z532;Q53SE2;E7ESH4;B7Z712;B9VPB4;B9VP19;E7EXB4;Q53QD5;C9JL25;C9JL19;C9JCQ4;B9VP24 | 60 kDa heat shock protein, mitochondrial | HSPD1 | 16 | 16 | 39.3 | 61.054 | 25.44 | 25.59 | 25.60 | 25.31 | 25.66 | 25.03 | 0.21 | 1.16 | 0.32614 |
| G3V5X4;D4YW74;Q8WXH0;Q8WXH0-2 | Nesprin-2 | SYNE2;TROPH | 2 | 2 | 0.3 | 787.71 | 23.13 | 22.70 | 23.50 | 22.64 | 22.31 | 23.76 | 0.21 | 1.16 | 0.69666 |
| Q2LE71;O15145;B2R4D5;B4DM63;F8VR50;C9JZD1 | Actin-related protein 2/3 complex subunit 3 | ARPC3 | 6 | 6 | 30.9 | 20.564 | 25.10 | 24.71 | 24.59 | 24.71 | 24.63 | 24.44 | 0.21 | 1.15 | 0.30375 |
| P26022 | Pentraxin-related protein PTX3 | PTX3 | 16 | 16 | 45.9 | 41.975 | 27.98 | 27.37 | 27.34 | 27.11 | 27.30 | 27.66 | 0.21 | 1.15 | 0.47461 |
| P02751-12;P02751-4;P02751-10;P02751-6;Q6MZM7;P02751-5;F8W7G7;P02751-9;B7ZLE5;P02751-14;P02751-13;P02751-8;P02751-17;P02751-3;A0A024R462;P02751-11;P02751;P02751-7;P02751-15;Q9UQS6;O95617;A6YID4;A6YID5;A6YID3;A6YID2;A6YID6;Q5CZ99;B4DU16;Q59G22;B4DTK1;Q6N084;H0Y7Z1;Q68CX6;H0Y4K8;Q6MZF4 | Fibronectin;Anastellin;Ugl-Y1;Ugl-Y2;Ugl-Y3 | FN1;DKFZp686O12165;DKFZp686I1370;DKFZp686L11144;DKFZp686O13149 | 6 | 6 | 3.9 | 221.29 | 23.15 | 23.26 | 23.29 | 22.71 | 23.06 | 23.34 | 0.20 | 1.15 | 0.34919 |
| Q16222-2;Q16222-3;Q16222;Q3KQV9 | UDP-N-acetylhexosamine pyrophosphorylase;UDP-N-acetylgalactosamine pyrophosphorylase;UDP-N-acetylglucosamine pyrophosphorylase | UAP1 | 9 | 9 | 22.8 | 57.028 | 24.25 | 24.35 | 24.02 | 24.20 | 23.86 | 24.00 | 0.19 | 1.14 | 0.23740 |
| Q16777;Q6FI13;Q99878;Q96KK5;Q9BTM1;P20671;P0C0S8;B2R5B3;H0YFX9;B4E0B3;B2R5B6;Q9BTM1-2;Q93077;Q7L7L0;P04908;Q96QV6;P16104;V9GZN0;Q71UI9-5;C9J386;Q71UI9-3;Q8IUE6 | Histone H2A type 2-C;Histone H2A type 2-A;Histone H2A type 1-J;Histone H2A type 1-H;Histone H2A.J;Histone H2A type 1-D;Histone H2A type 1;Histone H2A;Histone H2A type 1-C;Histone H2A type 3;Histone H2A type 1-B/E | HIST2H2AC;HIST2H2AA3;HIST1H2AJ;HIST1H2AH;H2AFJ;HIST1H2AD;HIST1H2AG;HIST1H2AC;HIST3H2A;HIST1H2AB | 7 | 5 | 58.1 | 13.988 | 27.57 | 26.79 | 26.53 | 26.82 | 26.84 | 26.66 | 0.19 | 1.14 | 0.58108 |
| P04406;P04406-2;Q2TSD0;E7EUT5;Q0QET7;B4DRV9;A4UCT1;Q5ZEY3;Q16768;O14556 | Glyceraldehyde-3-phosphate dehydrogenase | GAPDH | 27 | 27 | 84.2 | 36.053 | 31.78 | 32.07 | 32.22 | 31.29 | 32.12 | 32.09 | 0.19 | 1.14 | 0.56573 |
| O00764;F2Z2Y4;O00764-3;O00764-2;G1UI32;A8MV33 | Pyridoxal kinase | PDXK | 11 | 11 | 41.3 | 35.102 | 24.48 | 24.48 | 24.31 | 24.04 | 24.31 | 24.35 | 0.19 | 1.14 | 0.17320 |
| P00558;B7Z7A9;B4E1H9;E7ERH5;B4DHM5;B4DHB3;B4DWQ3;Q16444 | Phosphoglycerate kinase 1;Phosphoglycerate kinase | PGK1 | 44 | 36 | 89 | 44.614 | 32.18 | 32.23 | 32.38 | 31.88 | 32.17 | 32.18 | 0.19 | 1.14 | 0.18718 |
| P30085;B2R6S5;B4DDL4;Q5T0D2;P30085-2;B4DFW6 | UMP-CMP kinase | CMPK1;CMPK | 10 | 10 | 57.7 | 22.222 | 25.92 | 25.83 | 25.65 | 25.57 | 25.76 | 25.52 | 0.18 | 1.14 | 0.16092 |
| P11021;B4DEF7 | 78 kDa glucose-regulated protein | HSPA5 | 43 | 42 | 56.4 | 72.332 | 30.85 | 30.95 | 31.12 | 30.67 | 30.80 | 30.91 | 0.18 | 1.13 | 0.15819 |
| B4DWH5;A0A024R5A3;A0A024R580;P07384;B2RDI5;E9PL37;B4DS85 | Calpain-1 catalytic subunit | CAPN1 | 2 | 2 | 3 | 75.835 | 20.35 | 20.32 | 20.29 | 20.27 | 19.89 | 20.26 | 0.17 | 1.13 | 0.23656 |
| P68402;P68402-3;P68402-2;J3KNE3;P68402-4 | Platelet-activating factor acetylhydrolase IB subunit beta | PAFAH1B2 | 2 | 2 | 12.2 | 25.569 | 21.73 | 21.83 | 20.87 | 20.92 | 21.04 | 21.95 | 0.17 | 1.13 | 0.71695 |
| P12273 | Prolactin-inducible protein | PIP | 2 | 2 | 20.5 | 16.572 | 19.37 | 19.43 | 20.53 | 19.79 | 19.43 | 19.59 | 0.17 | 1.13 | 0.68020 |
| B7Z3I9;P13716;A0A024R877;Q6ZMU0;P13716-2;B7ZBK6 | Delta-aminolevulinic acid dehydratase | ALAD | 5 | 5 | 21.1 | 34.52 | 24.02 | 23.79 | 24.19 | 23.72 | 24.03 | 23.75 | 0.16 | 1.12 | 0.33802 |
| Q9Y316;Q9Y316-3;Q9Y316-2;A8K3Y8;Q2VIM3 | Protein MEMO1 | MEMO1 | 8 | 8 | 33.3 | 33.733 | 23.96 | 23.96 | 24.35 | 23.93 | 23.73 | 24.14 | 0.15 | 1.11 | 0.43235 |
| Q5JRX3-3;B4DRW8;B4DH07;Q5JRX3;Q5JRX3-2;B3KM51;B3KN37;B1APQ1;Q68DT6;B4DEU0;B1APQ0;H0Y4F7;H0Y7L7 | Presequence protease, mitochondrial | PITRM1 | 5 | 5 | 7.7 | 106.06 | 22.11 | 21.91 | 21.96 | 21.96 | 21.55 | 22.03 | 0.15 | 1.11 | 0.41897 |
| A8MZB2;P61599;P61599-2 | N-alpha-acetyltransferase 20 | NAA20 | 2 | 2 | 16.9 | 18.857 | 19.08 | 19.15 | 19.09 | 18.83 | 19.22 | 18.83 | 0.15 | 1.11 | 0.32516 |
| P45877;A8K2Q6;B4E200 | Peptidyl-prolyl cis-trans isomerase C;Peptidyl-prolyl cis-trans isomerase | PPIC | 3 | 3 | 14.6 | 22.763 | 24.05 | 22.98 | 22.88 | 23.45 | 23.18 | 22.93 | 0.12 | 1.09 | 0.78206 |
| P06744;B4DE36;P06744-2;K7EQ48;K7EP41;K7EPY4;K7ELR7;K7ERC6;K7ENA0;K7ERK8;Q59F85 | Glucose-6-phosphate isomerase | GPI | 38 | 2 | 72 | 63.146 | 31.32 | 31.42 | 31.37 | 30.99 | 31.49 | 31.27 | 0.12 | 1.09 | 0.46396 |
| P07339;C9JH19;H7C469;F8WD96;H7C1V0;F8W787 | Cathepsin D;Cathepsin D light chain;Cathepsin D heavy chain | CTSD | 19 | 19 | 55.3 | 44.552 | 27.35 | 27.39 | 27.26 | 27.15 | 27.14 | 27.37 | 0.11 | 1.08 | 0.25072 |
| P04040;B4DWK8;Q8TAK2 | Catalase | CAT | 12 | 12 | 26 | 59.755 | 24.24 | 23.98 | 24.11 | 23.97 | 23.97 | 24.07 | 0.11 | 1.08 | 0.25823 |
| B4E2Z3;P08195-2;P08195-3;F5GZS6;P08195;J3KPF3;P08195-4;A0A024R599;F5GZI0;H0YFS2;H0YFX4;F5H0E2 | 4F2 cell-surface antigen heavy chain | SLC3A2 | 8 | 8 | 23.9 | 55.939 | 24.27 | 24.17 | 24.40 | 24.29 | 24.07 | 24.15 | 0.11 | 1.08 | 0.30421 |
| Q71DI3 | Histone H3.2 | HIST2H3A | 5 | 1 | 43.4 | 15.388 | 27.45 | 27.09 | 26.70 | 27.19 | 26.71 | 27.03 | 0.11 | 1.08 | 0.70368 |
| P09104;A8K3B0;B7Z2X9;F5H0C8;P13929-2;P13929;F5H1C3;D3DTL4;P13929-3;U3KQP4;U3KQQ1;Q9NPL4 | Gamma-enolase;Enolase | ENO2 | 15 | 14 | 57.1 | 47.268 | 27.00 | 26.89 | 26.49 | 26.46 | 26.88 | 26.73 | 0.10 | 1.08 | 0.62444 |
| C9J813;Q9NYG1;Q05682-5;E9PGZ1;Q05682-4;F5H1Z9;B7Z6G4;Q6PJM5;B4DPW5;E7EX44;Q05682-3;Q05682-6;Q05682-2;Q05682;Q6P707;B4E3I0 | Caldesmon | CALD1 | 4 | 4 | 10.5 | 54.158 | 23.21 | 23.18 | 23.37 | 23.58 | 22.83 | 23.05 | 0.10 | 1.07 | 0.68186 |
| P62942;Q5W0X3;Q0VDC6;Q1JUQ5;Q1JUQ3;Q0VDC5;Q1JUQ4;A8MSS1;Q5VVH2 | Peptidyl-prolyl cis-trans isomerase FKBP1A;Peptidyl-prolyl cis-trans isomerase | FKBP1A;FKBP12-Exip2 | 4 | 4 | 73.1 | 11.951 | 25.99 | 25.54 | 23.05 | 23.44 | 25.63 | 25.23 | 0.09 | 1.07 | 0.93823 |
| V9HWC6;P23284 | Peptidyl-prolyl cis-trans isomerase B | PPIB | 23 | 23 | 76.9 | 22.742 | 31.77 | 31.95 | 32.04 | 31.65 | 31.99 | 31.84 | 0.09 | 1.07 | 0.50291 |
| B3KVN2;P49354-2;P49354;D3DSY9;E9PPM9;H0YCW1;H0YE66;E9PQP6 | Protein farnesyltransferase/geranylgeranyltransferase type-1 subunit alpha | FNTA | 2 | 2 | 12.3 | 27.443 | 18.88 | 19.62 | 19.07 | 19.97 | 18.74 | 18.63 | 0.08 | 1.06 | 0.87746 |
| Q5TFQ8;B4DP97;P78324-4;P78324;D3DVW9;P78324-2;H3BQ21;H3BUA5;H3BRP9;Q9P1W8-3;H3BV43;H3BML4;H9KV29;O00241-2;A2RRP5;Q9P1W8-4;Q9P1W8-2;D3DVW8;A8K9N0;Q9P1W8;O00241;Q2QD20;H3BTT9;H3BU43;H3BSK5 | Signal-regulatory protein beta-1 isoform 3;Tyrosine-protein phosphatase non-receptor type substrate 1 | SIRPB1;SIRPA;PTPNS1 | 7 | 7 | 22.6 | 43.359 | 26.01 | 26.10 | 26.38 | 25.94 | 26.13 | 26.17 | 0.08 | 1.06 | 0.58545 |
| Q14118;A0A024R2W4;B4DN78;C9JYS1;C9J196;C9JQL4 | Dystroglycan;Alpha-dystroglycan;Beta-dystroglycan | DAG1 | 9 | 9 | 14.4 | 97.44 | 25.46 | 25.16 | 25.65 | 25.54 | 25.20 | 25.32 | 0.07 | 1.05 | 0.70563 |
| P50583 | Bis(5-nucleosyl)-tetraphosphatase [asymmetrical] | NUDT2 | 6 | 6 | 60.5 | 16.829 | 22.50 | 22.83 | 22.65 | 22.55 | 22.39 | 22.83 | 0.07 | 1.05 | 0.68561 |
| P12236;Q59EI9;I7HJJ0;V9GYG0;Q59EP7;P12235 | ADP/ATP translocase 3;ADP/ATP translocase 1 | SLC25A6;SLC25A4 | 2 | 2 | 14.4 | 32.866 | 22.63 | 19.52 | 18.96 | 21.05 | 20.55 | 19.32 | 0.06 | 1.05 | 0.96177 |
| Q14315-2;Q14315;Q59H94;B3KM41;A4D1J9 | Filamin-C | FLNC | 33 | 33 | 17.3 | 287.28 | 27.25 | 27.18 | 27.44 | 27.07 | 27.24 | 27.37 | 0.06 | 1.04 | 0.62822 |
| E9PKY5;B3KSZ1;B4E3F2;E9PEQ6;Q9UNP9-2;Q9UNP9;A8KAM9;Q9UNP9-3 | Peptidyl-prolyl cis-trans isomerase;Peptidyl-prolyl cis-trans isomerase E | PPIE | 3 | 2 | 11.7 | 23.833 | 21.18 | 20.72 | 21.28 | 20.98 | 20.99 | 21.05 | 0.06 | 1.04 | 0.76075 |
| O00560;O00560-3 | Syntenin-1 | SDCBP | 8 | 1 | 49.3 | 32.444 | 24.43 | 24.01 | 24.21 | 24.04 | 24.21 | 24.23 | 0.06 | 1.04 | 0.70210 |
| P62837;D6RFM0;P62837-2;D6RAH7;H9KV45;P61077;P61077-2;P61077-3;D6RAW0;D6RA11;Q9UQL0;C9J9H9;Q9Y2X8;P51668 | Ubiquitin-conjugating enzyme E2 D2;Ubiquitin-conjugating enzyme E2 D3 | UBE2D2;UBE2D3 | 3 | 3 | 33.3 | 16.735 | 24.73 | 25.26 | 24.79 | 26.22 | 24.35 | 24.04 | 0.05 | 1.04 | 0.94303 |
| P55809;Q6IAV5;E9PDW2;B7Z609;A1E286;B7Z528 | Succinyl-CoA:3-ketoacid coenzyme A transferase 1, mitochondrial;Succinyl-CoA:3-ketoacid-coenzyme A transferase | OXCT1;OXCT | 16 | 16 | 44.4 | 56.157 | 25.87 | 25.88 | 25.88 | 25.70 | 26.10 | 25.70 | 0.04 | 1.03 | 0.76329 |
| P54687-4;P54687;P54687-5;P54687-2;P54687-3;F5H2F2 | Branched-chain-amino-acid aminotransferase, cytosolic | BCAT1 | 10 | 10 | 35.3 | 42.837 | 24.69 | 24.76 | 24.88 | 24.19 | 25.14 | 24.88 | 0.04 | 1.03 | 0.90053 |
| P49189;B4DE91;B9EKV4;B4DX14;B4DXY7;B4DYY1 | 4-trimethylaminobutyraldehyde dehydrogenase | ALDH9A1 | 9 | 9 | 21.7 | 53.801 | 25.03 | 25.10 | 25.29 | 24.93 | 24.95 | 25.45 | 0.03 | 1.02 | 0.86203 |
| P14314-2;A0A024R7F1;P14314;K7ELL7;B4DJQ5;A2VCQ4;K7EPW7;K7EKX1;K7EJ70 | Glucosidase 2 subunit beta | PRKCSH | 18 | 18 | 37.5 | 59.177 | 27.43 | 27.52 | 27.52 | 27.52 | 27.39 | 27.45 | 0.03 | 1.02 | 0.52076 |
| O60888-3;O60888;O60888-2;C9IZG4;C9IZQ5 | Protein CutA | CUTA | 5 | 5 | 55.8 | 16.832 | 24.72 | 24.84 | 24.69 | 24.83 | 25.03 | 24.30 | 0.03 | 1.02 | 0.89026 |
| D3DSM4;P39060-2;D3DSM5;P39060-1;P39060;Q8NG19;H7BXV5;H7C457 | Collagen alpha-1(XVIII) chain;Endostatin | COL18A1 | 7 | 7 | 7.2 | 135.51 | 23.78 | 23.43 | 24.04 | 23.76 | 23.61 | 23.78 | 0.03 | 1.02 | 0.87028 |
| Q9BWS9-3;Q9BWS9;J3KNL3;B7Z705;B4DN31;A0A024RCB5;Q9BWS9-2;E9PI70;E9PIP0;E9PRL3;E9PPH0;E9PPA0;E9PKF1;H0YDL6;H0YCC4;H0YC89 | Chitinase domain-containing protein 1 | CHID1 | 9 | 9 | 27.3 | 41.677 | 23.82 | 23.54 | 23.99 | 23.48 | 23.75 | 24.05 | 0.02 | 1.02 | 0.92338 |
| P09972;B7Z3K9;B7Z1Y2;B7Z3K7;B7Z1N6;A8MVZ9;J3KSV6;J3QKP5;C9J8F3;B7Z1Z9;J3QKK1;B7Z1H6;K7EKH5;B7Z1L5 | Fructose-bisphosphate aldolase C;Fructose-bisphosphate aldolase | ALDOC | 13 | 13 | 44.5 | 39.455 | 25.94 | 25.68 | 25.87 | 25.72 | 25.96 | 25.76 | 0.02 | 1.01 | 0.88412 |
| K7ER15;Q9H0R4;K7EMY7;Q9H0R4-2;K7EQD2;K7EJQ8;K7ENF3 | Haloacid dehalogenase-like hydrolase domain-containing protein 2 | HDHD2 | 6 | 6 | 49.5 | 22.49 | 24.18 | 24.49 | 24.72 | 24.29 | 24.73 | 24.32 | 0.02 | 1.01 | 0.94406 |
| B3KT06;B3KPS3;P68363;Q9BQE3;Q53GA7;A8JZY9;F5H5D3;Q8WU19;Q71U36-2;Q71U36;B7Z1K5;F8VVB9;Q13748-2;P68366-2;P68366;Q13748;B4DDU2;Q8N532;Q6PEY2;F8VQQ4;Q9NY65-2;B4DQK4;Q9NY65;C9J2C0;Q7Z3M3;F8VRZ4;F8VS66;Q9UQM3;F8VX09;F8VWV9;C9JDS9;B4DN58;C9K0S6;F8VRK0;Q6QMJ5;C9JDL2;C9JEV8;C9JQ00;C9JJQ8;F8VXZ7;F8W0F6;Q9H853;V9GZ17 | Tubulin alpha-1B chain;Tubulin alpha-1C chain;Tubulin alpha-1A chain;Tubulin alpha-3C/D chain;Tubulin alpha-4A chain;Tubulin alpha-3E chain;Tubulin alpha-8 chain | TUBA1B;TUBA1C;TUBA1A;TUBA3C;TUBA4A;TUBA3E;TUBA8;DKFZp686L04275 | 12 | 12 | 41.1 | 46.341 | 24.90 | 25.06 | 24.74 | 24.51 | 24.74 | 25.40 | 0.01 | 1.01 | 0.97081 |
| Q6FHL9;Q15121;B1AKZ3;B1AKZ5 | Astrocytic phosphoprotein PEA-15 | PEA15 | 2 | 2 | 23.8 | 15.068 | 19.27 | 20.46 | 20.61 | 19.87 | 20.07 | 20.37 | 0.01 | 1.01 | 0.98644 |
| B3KSS4;P15151-3;P15151-2;P15151-4;P15151;A8K4I1 | Poliovirus receptor | PVR | 3 | 3 | 8 | 39.579 | 24.04 | 24.15 | 24.47 | 24.33 | 24.39 | 23.94 | 0.00 | 1.00 | 0.98494 |
| P12955;J3K000;P12955-3;P12955-2;K7ES25;V9GY89;V9GYL0;V9GYE4 | Xaa-Pro dipeptidase | PEPD | 24 | 24 | 63.3 | 54.548 | 28.27 | 28.59 | 28.54 | 28.26 | 28.75 | 28.38 | 0.00 | 1.00 | 0.98411 |
| Q9BS40;H7C5A4 | Latexin | LXN | 4 | 4 | 22.1 | 25.75 | 26.97 | 27.00 | 27.17 | 27.34 | 26.97 | 26.82 | 0.00 | 1.00 | 0.99379 |
| B7Z7Y3;Q96PD2;Q96PD2-2;Q14089;C9JIW6 | Discoidin, CUB and LCCL domain-containing protein 2 | DCBLD2 | 5 | 5 | 10.5 | 73.743 | 23.69 | 23.81 | 23.90 | 23.75 | 23.88 | 23.78 | 0.00 | -1.00 | 0.97212 |
| O94760;O94760-2;B4DYP1;B4DGT0;B4E3V1 | N(G),N(G)-dimethylarginine dimethylaminohydrolase 1 | DDAH1 | 18 | 17 | 56.1 | 31.121 | 27.89 | 27.98 | 28.18 | 27.85 | 28.16 | 28.06 | -0.01 | -1.00 | 0.96145 |
| Q9BV57;Q9BV57-2;H7C382 | 1,2-dihydroxy-3-keto-5-methylthiopentene dioxygenase | ADI1 | 6 | 6 | 47.5 | 21.498 | 24.16 | 24.26 | 23.62 | 24.18 | 24.20 | 23.70 | -0.01 | -1.01 | 0.96958 |
| Q9Y2B0;F8VXJ7;H0YI18;F8W1K5;H0YIH9;F8W1U5;Q9Y2B0-2 | Protein canopy homolog 2 | CNPY2 | 9 | 2 | 52.2 | 20.652 | 25.32 | 24.96 | 25.15 | 25.40 | 24.93 | 25.14 | -0.01 | -1.01 | 0.93725 |
| B7Z168;B0FYA8;E3SFN0;B0FYA7;A5YAK8;B0FWZ3;A5YAK7;Q7RTW5;B5BU95;A5YAK6;Q6PK61;Q02297-8;Q02297-4;Q02297-12;Q02297-9;A6MW54;B0FYA9;Q02297-3;E7EX30;A6MW56;A6MW55;Q02297-11;E3SFM9;F8W9E3;Q7RTW3;Q02297-7;Q7RTV8;Q02297;B9EK51;Q7RTW4;Q02297-6;Q02297-2;H7BXY2;H0YBA3 | Pro-neuregulin-1, membrane-bound isoform;Neuregulin-1 | NRG1 | 2 | 2 | 13.8 | 17.983 | 21.72 | 21.70 | 21.85 | 22.02 | 21.73 | 21.57 | -0.02 | -1.01 | 0.90574 |
| B4E3P0;P53396-2;P53396;Q4LE36;Q8N9C4;K7ESG8 | ATP-citrate synthase | ACLY;ACLY variant protein | 8 | 8 | 13.9 | 91.098 | 22.28 | 21.88 | 22.20 | 21.97 | 22.23 | 22.23 | -0.02 | -1.01 | 0.90396 |
| P23470-2;P23470;O60420 | Receptor-type tyrosine-protein phosphatase gamma | PTPRG | 5 | 5 | 6.2 | 158.77 | 21.42 | 21.99 | 22.74 | 20.92 | 22.64 | 22.66 | -0.03 | -1.02 | 0.97140 |
| Q13740-2;Q13740;F5GXJ9;B3KNN9;B4DX43;Q13740-3;H7C543;Q13740-4 | CD166 antigen | ALCAM | 23 | 23 | 43.2 | 63.664 | 28.45 | 28.63 | 28.49 | 28.31 | 28.51 | 28.82 | -0.03 | -1.02 | 0.86870 |
| Q13308;Q13308-6;Q13308-4;Q13308-2;Q59FV9;Q13308-5;Q13308-3;Q86X91;B7Z471;F8WDG7;C9J9E8;H7C5L0;C9JQR6;Q9NSQ6;H0Y8F1;B3KP36 | Inactive tyrosine-protein kinase 7 | PTK7 | 21 | 21 | 25.2 | 118.39 | 27.35 | 27.38 | 27.60 | 27.48 | 27.46 | 27.48 | -0.03 | -1.02 | 0.73007 |
| P05455;B5BUB5;E7ERC4;Q9UMH5;E9PGX9;E9PFL9;Q14730;Q2F832 | Lupus La protein | SSB | 5 | 5 | 15.9 | 46.836 | 22.72 | 23.00 | 23.01 | 22.98 | 22.78 | 23.08 | -0.03 | -1.02 | 0.81516 |
| B4E0K9;Q59E90;O00754-2;O00754;A8K6A7;Q93093;M0R2P5;M0R174;M0QZG6;M0QZ24 | Lysosomal alpha-mannosidase;Lysosomal alpha-mannosidase A peptide;Lysosomal alpha-mannosidase B peptide;Lysosomal alpha-mannosidase C peptide;Lysosomal alpha-mannosidase D peptide;Lysosomal alpha-mannosidase E peptide | MAN2B1 | 17 | 17 | 24.9 | 107.46 | 25.59 | 25.84 | 26.04 | 25.66 | 26.09 | 25.82 | -0.03 | -1.02 | 0.86783 |
| Q6FI45;Q5U043;P11766;Q2VIM7;H0YAG8;D6RFE4;D6RAY0;D6R9G2 | Alcohol dehydrogenase class-3 | ADH5 | 16 | 16 | 44.1 | 39.69 | 27.76 | 27.86 | 28.13 | 27.61 | 28.17 | 28.07 | -0.03 | -1.02 | 0.87266 |
| Q5JPJ9;O95274;A8K4E0 | Ly6/PLAUR domain-containing protein 3 | DKFZp686D0114;LYPD3 | 3 | 3 | 15.6 | 30.497 | 22.17 | 22.03 | 21.63 | 20.35 | 22.81 | 22.78 | -0.04 | -1.03 | 0.96677 |
| Q6UVK1 | Chondroitin sulfate proteoglycan 4 | CSPG4 | 4 | 4 | 2.4 | 250.53 | 21.42 | 21.38 | 21.74 | 21.37 | 21.85 | 21.43 | -0.04 | -1.03 | 0.85409 |
| Q14697;Q14697-2;B4DJ30;F5H6X6;B4DSM6;A0A024R592;E9PKU7;B4DZ53;B4DIW2;E9PNH1;Q9BS14 | Neutral alpha-glucosidase AB | GANAB | 4 | 4 | 6 | 106.87 | 21.17 | 21.24 | 21.25 | 21.10 | 21.22 | 21.46 | -0.04 | -1.03 | 0.72532 |
| P61769;F5H6I0;A6XMH4;A6XND9;B4E0X1;H0YLF3;A6XMH5 | Beta-2-microglobulin;Beta-2-microglobulin form pI 5.3 | B2M | 10 | 10 | 77.3 | 13.714 | 31.33 | 30.65 | 30.46 | 30.74 | 30.47 | 31.36 | -0.04 | -1.03 | 0.91754 |
| Q2XPP3 |  |  | 3 | 0 | 16.1 | 36.768 | 22.47 | 22.99 | 22.26 | 23.01 | 22.52 | 22.31 | -0.04 | -1.03 | 0.89707 |
| Q4J6C6-4;Q4J6C6-2;Q4J6C6;Q4J6C6-3 | Prolyl endopeptidase-like | PREPL | 2 | 2 | 5.2 | 73.351 | 19.25 | 19.98 | 19.86 | 19.59 | 20.20 | 19.47 | -0.06 | -1.04 | 0.86526 |
| P55287-2;B4DMA7;Q96CZ9;P55287;Q59EQ1;H3BUU9;B7Z2M1;H3BQH2 | Cadherin-11 | CDH11 | 3 | 3 | 5.1 | 76.456 | 21.78 | 21.37 | 21.52 | 21.71 | 21.46 | 21.68 | -0.06 | -1.04 | 0.69566 |
| Q0D2I8;P28161;A8HT81;P28161-2;E9PHN6;F6XZQ7;E9PHN7;E9PLF1;X5DNN9;B9ZVX7;P09488-2;P09488;E7EWW9;H3BQT3;E9PGV1;Q03013-3;Q03013-2;Q03013;X5D7U4;Q5T8Q9;P46439;Q5T8R1;Q9UE37;Q6ZSP8;A4UJ43;P21266;Q59EJ5 | Glutathione S-transferase Mu 2 | GSTM2 | 10 | 10 | 41.3 | 25.684 | 24.94 | 25.02 | 25.28 | 24.85 | 25.36 | 25.22 | -0.06 | -1.05 | 0.73930 |
| B4DL56;Q8NCL4;Q24JS2;D7UNW5;B3KRZ8;S4R3S5 | Polypeptide N-acetylgalactosaminyltransferase 6 | GALNT6 | 4 | 4 | 10.3 | 68.877 | 23.05 | 22.58 | 22.83 | 22.66 | 22.81 | 23.22 | -0.08 | -1.06 | 0.73905 |
| H0YCC7;E9PRA1;E9PP81;Q17R31-5;Q17R31-2;Q17R31;Q17R31-3;B7Z2Z9;Q17R31-4;H0YD82;E9PPF0;E9PQP8 | Putative deoxyribonuclease TATDN3 | TATDN3 | 3 | 3 | 31.2 | 15.822 | 20.51 | 20.42 | 20.66 | 20.62 | 20.93 | 20.28 | -0.08 | -1.06 | 0.71738 |
| Q12765;Q12765-2;Q12765-3;B4DH22;C9K052;B4DIV7;B8ZZP4;C9J7U9;B4DE46 | Secernin-1 | SCRN1 | 11 | 11 | 30.4 | 46.382 | 25.81 | 25.81 | 26.14 | 26.17 | 26.09 | 25.74 | -0.08 | -1.06 | 0.66511 |
| Q9NQW7;Q9NQW7-3;Q5T6H7;B3KSI7;Q9NQW7-2;G8JLB2;B4E2P4;Q5T6H2;B4DIS4;Q5T6H3 | Xaa-Pro aminopeptidase 1 | XPNPEP1 | 15 | 15 | 36.8 | 69.917 | 25.43 | 25.33 | 25.34 | 25.12 | 25.47 | 25.78 | -0.09 | -1.07 | 0.65545 |
| P49915-2;B4DUT7;Q53F90;P49915 | GMP synthase [glutamine-hydrolyzing] | GMPS | 2 | 2 | 4 | 65.928 | 19.73 | 19.71 | 19.19 | 19.96 | 19.75 | 19.20 | -0.09 | -1.07 | 0.76273 |
| Q9H008;Q9H008-2;Q5T1Z0 | Phospholysine phosphohistidine inorganic pyrophosphate phosphatase | LHPP | 4 | 4 | 23.7 | 29.165 | 22.43 | 22.72 | 22.80 | 22.97 | 22.71 | 22.54 | -0.09 | -1.07 | 0.60870 |
| B4DVE1;Q08380;B3KP88;B4DDG4;B4DWA8;B4DI70;K7EP36;K7ESM3;K7EKQ5;K7ES75;K7EN99;K7ERZ6;K7EJY8;K7EJD3;K7EQT9 | Galectin-3-binding protein | LGALS3BP | 20 | 20 | 36.8 | 64.096 | 29.07 | 29.02 | 29.17 | 29.03 | 29.16 | 29.35 | -0.10 | -1.07 | 0.41194 |
| P17050 | Alpha-N-acetylgalactosaminidase | NAGA | 4 | 4 | 12.9 | 46.564 | 22.30 | 22.28 | 22.48 | 22.26 | 22.43 | 22.68 | -0.11 | -1.08 | 0.48114 |
| P00338;P00338-3;B4DJI1;P00338-4;P00338-5;P00338-2;F5GYU2;F5GXH2;F5H5J4;F5H6W8;F5GZQ4;F5H8H6;F5GXC7;F5GWW2;F5GXU1;F5H5G7;F5H155;Q96L19;G3XAP5;F5H245;Q6ZMR3;P07864;G9BCY8;G9BCY7;D6NKH9 | L-lactate dehydrogenase A chain;L-lactate dehydrogenase | LDHA | 37 | 16 | 91.3 | 36.688 | 33.02 | 32.99 | 33.02 | 33.17 | 33.29 | 32.93 | -0.12 | -1.09 | 0.30632 |
| B7ZAF0;Q5JP53;Q6LC01;P07437;Q5ST81;Q8N6N5;Q8IZ29;Q8IWP6;P68371;B4DMJ5;E9PBJ4;B7ZAK1;A0A024QZU2;Q9BVA1;Q13885;B2R6L0;B4DY90;O43209;B4DJ43;Q13509-2;Q9BV28;B4DE77;P04350;Q2NKY5;Q13509;B2RBD5;A1L195;Q96HX0;B4DFH6;A5D907;A5D906;Q96B85;B4DXZ5;Q3ZCR3;B4DMU8;Q9BUU9;B4E052;B4DQN9;B3KML9;Q53G92;A4UCT2;G3V2A3;Q1KSF8;B4E386;Q9UGA2;K7ESM5;B7Z4N1;B3KS31;B4DP54;Q9BUF5;M0R1I1;M0QY85;Q6P602;M0QZL7;M0R278;F5H0I4;Q5SQY0;I3L2F9;Q3ZCM7;A6NNZ2;Q9H4B7 | Tubulin beta chain;Tubulin beta-4B chain;Tubulin beta-2B chain;Tubulin beta-2A chain;Tubulin beta-3 chain;Tubulin beta-4A chain | TUBB;TUBB2C;TUBB4B;TUBB2B;TUBB2A;TUBB3;TUBB4A;TUBB6 | 7 | 7 | 21.7 | 46.566 | 23.12 | 23.49 | 22.52 | 22.96 | 23.10 | 23.47 | -0.13 | -1.10 | 0.70111 |
| P08253;P08253-3;P08253-2;H3BR66;H3BS34;H3BV48;Q2EF79;Q53GF1;P09237 | 72 kDa type IV collagenase;PEX | MMP2 | 28 | 28 | 56.8 | 73.881 | 27.74 | 27.20 | 27.58 | 27.92 | 27.48 | 27.56 | -0.15 | -1.11 | 0.51225 |
| Q9UNN8 | Endothelial protein C receptor | PROCR | 5 | 5 | 26.1 | 26.671 | 26.74 | 27.12 | 27.26 | 26.93 | 27.38 | 27.26 | -0.15 | -1.11 | 0.49521 |
| P15586;A8K6V6;B4DTT0;Q7Z3X3;H7C3P4;B4DYH8;F6S8M0;B4E284;H0YFA9;F5H4C6;B4DKX8 | N-acetylglucosamine-6-sulfatase | GNS;DKFZp686E12166 | 16 | 16 | 32.4 | 62.081 | 28.22 | 28.37 | 28.48 | 28.31 | 28.57 | 28.67 | -0.16 | -1.12 | 0.29696 |
| P07686;Q5URX0;H0YA83;H0Y9B6;H0Y9M3;D6REQ8 | Beta-hexosaminidase subunit beta;Beta-hexosaminidase subunit beta chain B;Beta-hexosaminidase subunit beta chain A | HEXB | 26 | 26 | 49.5 | 63.111 | 28.36 | 28.16 | 28.49 | 28.28 | 28.66 | 28.58 | -0.17 | -1.12 | 0.31480 |
| B3KTR9;O60565-2;O60565;Q9H772 | Gremlin-1;Gremlin-2 | GREM1;GREM2 | 2 | 2 | 24.6 | 13.413 | 22.78 | 22.70 | 23.06 | 23.92 | 22.15 | 23.00 | -0.18 | -1.13 | 0.75156 |
| O00622;Q9UID7;B4DI61;Q53FA4 | Protein CYR61 | CYR61 | 5 | 5 | 16.3 | 42.026 | 25.06 | 24.72 | 25.03 | 25.00 | 25.26 | 25.08 | -0.18 | -1.13 | 0.24861 |
| Q15293;B7Z1M1;Q5J7V8 | Reticulocalbin-1 | RCN1 | 5 | 5 | 15.4 | 38.89 | 24.50 | 24.30 | 24.25 | 24.73 | 24.66 | 24.21 | -0.18 | -1.13 | 0.37298 |
| P07093-2;P07093-3;B4DMR3;C9JN98;C9K031 | Glia-derived nexin | SERPINE2 | 23 | 1 | 61.2 | 44 | 30.71 | 30.54 | 30.30 | 30.51 | 30.73 | 30.86 | -0.18 | -1.14 | 0.30642 |
| O60568;B3KQQ3;H7C2V1;Q9UG85;H7C2S8;B4DMP0;B4DPX5;H7C0B8;C9JIX5;C9JU11 | Procollagen-lysine,2-oxoglutarate 5-dioxygenase 3 | PLOD3 | 15 | 15 | 30.4 | 84.784 | 24.62 | 24.03 | 24.32 | 24.41 | 24.48 | 24.64 | -0.19 | -1.14 | 0.36524 |
| P04083;B5BU38;B4DL19;Q5T3N1;Q5T3N0;Q05BR2 | Annexin A1;Annexin | ANXA1 | 20 | 20 | 60.1 | 38.714 | 26.36 | 26.21 | 26.46 | 26.56 | 26.48 | 26.57 | -0.19 | -1.14 | 0.07514 |
| H0YK49;H0YNX6;P13804-2;P13804;H0YM12;H0YL83;H0YL12;H0YLU7 | Electron transfer flavoprotein subunit alpha, mitochondrial | ETFA | 2 | 2 | 17.5 | 24.16 | 21.73 | 18.65 | 18.58 | 19.70 | 18.18 | 21.66 | -0.19 | -1.14 | 0.90173 |
| B4DFW1;P48745 | Protein NOV homolog | NOV | 11 | 11 | 40.4 | 37.652 | 27.80 | 27.56 | 28.05 | 28.26 | 27.77 | 27.98 | -0.20 | -1.15 | 0.38489 |
| Q86U75;Q9HCN8 |  |  | 5 | 2 | 15.8 | 67.017 | 22.37 | 22.32 | 22.31 | 22.55 | 22.31 | 22.72 | -0.20 | -1.15 | 0.18286 |
| P01034 | Cystatin-C | CST3 | 7 | 7 | 57.5 | 15.799 | 27.34 | 27.17 | 27.07 | 27.57 | 27.32 | 27.27 | -0.20 | -1.15 | 0.18373 |
| P42765;B2RB23;B3KNP8;K7EME0;K7ER88;K7EJB1;K7EJ68 | 3-ketoacyl-CoA thiolase, mitochondrial | ACAA2 | 12 | 12 | 37.3 | 41.924 | 25.89 | 26.01 | 26.10 | 26.09 | 26.38 | 26.15 | -0.21 | -1.15 | 0.12601 |
| Q8TE01 |  | derp12 | 10 | 10 | 31.2 | 38.192 | 25.22 | 24.84 | 25.32 | 25.14 | 25.53 | 25.35 | -0.21 | -1.15 | 0.32575 |
| Q8WVB5;Q8TDC4;Q8N339;P80297;P13640-2;P04732;P02795;A0A024R6T4;P13640 | Metallothionein;Metallothionein-1M;Metallothionein-1X;Metallothionein-1G;Metallothionein-1E;Metallothionein-2 | MT1X;MT1M;MT1G;MT1E;MT2A | 2 | 2 | 21.3 | 6.0542 | 26.79 | 27.29 | 26.73 | 28.02 | 26.77 | 26.64 | -0.21 | -1.16 | 0.68236 |
| P08236-3;P08236;P08236-2;B2R6X2;B4DTH9;F8WBK6;F2Z3L6 | Beta-glucuronidase | GUSB | 8 | 8 | 16 | 58.345 | 23.43 | 23.42 | 23.68 | 23.56 | 23.81 | 23.81 | -0.21 | -1.16 | 0.14781 |
| E2PU09;A0PK02;O15031;A6QRH1 | Plexin-B2 | PLXNB2 | 3 | 3 | 9.4 | 51.754 | 20.50 | 20.69 | 21.37 | 19.99 | 21.61 | 21.60 | -0.21 | -1.16 | 0.74073 |
| O00391-2;A8K4C2;A8K477;O00391;Q13876;A8MXT8 | Sulfhydryl oxidase 1 | QSOX1;QSCN6 | 8 | 8 | 18.2 | 66.86 | 23.64 | 23.42 | 23.65 | 24.18 | 23.49 | 23.69 | -0.22 | -1.16 | 0.37004 |
| P63261;B4E3A4;B4DVQ0;I3L1U9;I3L4N8;K7EM38;I3L3R2;J3KT65 | Actin, cytoplasmic 2;Actin, cytoplasmic 2, N-terminally processed | ACTG1 | 20 | 1 | 58.9 | 41.792 | 29.77 | 29.69 | 29.39 | 29.68 | 29.69 | 30.14 | -0.22 | -1.16 | 0.31250 |
| Q13219;A0A024R842;F5GZ19;B4DTA8;B4DTA7;Q5QFB7 | Pappalysin-1 | PAPPA | 16 | 16 | 13.7 | 180.97 | 24.88 | 25.06 | 24.85 | 25.30 | 24.89 | 25.28 | -0.23 | -1.17 | 0.20793 |
| Q96I99;H0Y852;Q9Y436;Q3ZCW5;E9PDQ8;Q96I99-2 | Succinyl-CoA ligase [GDP-forming] subunit beta, mitochondrial | SUCLG2;DKFZp586M2023 | 2 | 2 | 6.2 | 46.51 | 20.80 | 20.65 | 21.18 | 21.08 | 21.35 | 20.88 | -0.23 | -1.17 | 0.34022 |
| Q58F15;E9PG71;P54764;C9JIX8;F5GZZ5;Q584H6;Q53TA0;B7Z6Q8 | Ephrin type-A receptor 4 | EPHA4 | 2 | 2 | 2.7 | 105.74 | 20.10 | 20.03 | 20.20 | 18.30 | 21.01 | 21.70 | -0.23 | -1.17 | 0.83677 |
| Q8NBJ7;J3KQJ1;C9JL30;C9J660;Q8NBJ7-2;H7C3B2;A8MXB9;Q75LP3;Q8NBJ7-3;F8WA42;B4DLK7;E9PBT8;B4DQ27;E9PG02;B4DN71;Q8NBJ7-5;J3QT17;F8WEX5;F8WEV7;F8WES7;Q8NBJ7-4 | Sulfatase-modifying factor 2 | SUMF2;DKFZP566I1024 | 10 | 10 | 37.9 | 33.843 | 24.56 | 24.47 | 24.67 | 24.75 | 25.06 | 24.60 | -0.24 | -1.18 | 0.18034 |
| Q96RE1;Q6IPT9;Q6IPN6;Q5VTE0;Q53HR5;Q53HQ7;Q53HM9;Q53G85;P68104;A8K9C4;B4DV42;Q9NZS6;Q6IQ15;Q53GE9;Q53GA1;Q8IUB0;Q53HR1;B4DNE0;Q53G89;A9X7H1;Q6P4C9;Q504Z0;B4E2C5;Q16577;Q9H2I7;Q05639;Q96C29;Q8TBL1;Q6P082;Q96CD8;Q59GP5;Q14222;Q5JR01;Q2F837;A6PW80;Q96EB3 | Elongation factor 1-alpha;Putative elongation factor 1-alpha-like 3;Elongation factor 1-alpha 1;Elongation factor 1-alpha 2 | EEF1A1L14;EEF1A1;EEF1A1P5;PTI-1;EEF1A2 | 8 | 8 | 28.1 | 43.024 | 24.62 | 24.75 | 24.84 | 25.13 | 24.73 | 25.06 | -0.24 | -1.18 | 0.16223 |
| O95479;R4GMU1 | GDH/6PGL endoplasmic bifunctional protein;Glucose 1-dehydrogenase;6-phosphogluconolactonase | H6PD | 2 | 2 | 2.8 | 88.892 | 20.25 | 19.98 | 20.24 | 20.55 | 20.64 | 20.01 | -0.24 | -1.18 | 0.32522 |
| Q92820;A8K335;B4DVI2 | Gamma-glutamyl hydrolase | GGH | 19 | 19 | 46.9 | 35.964 | 30.10 | 30.06 | 30.38 | 30.18 | 30.63 | 30.48 | -0.25 | -1.19 | 0.20848 |
| Q53FK2;P48723;F5GWS8;B3KY30;B4DQX6 | Heat shock 70 kDa protein 13 | HSPA13 | 8 | 8 | 18.3 | 51.867 | 23.77 | 23.91 | 23.60 | 23.79 | 24.00 | 24.25 | -0.25 | -1.19 | 0.19861 |
| A0A024R972;P11047 | Laminin subunit gamma-1 | LAMC1 | 4 | 4 | 3.6 | 174.01 | 26.10 | 25.50 | 25.85 | 26.40 | 25.92 | 25.88 | -0.25 | -1.19 | 0.35108 |
| B7Z5V6 |  |  | 2 | 2 | 4.8 | 71.795 | 23.52 | 24.22 | 23.74 | 25.29 | 21.76 | 25.21 | -0.26 | -1.20 | 0.83724 |
| B4DHC3;Q86WJ2;Q68DT8;Q15262-2;Q15262-3;F5GXI4;Q5TG12;Q15262-4;E9PGC5;Q59EZ1;Q15262;H0YDP9;E9PRW2;H0Y6P5 | Receptor-type tyrosine-protein phosphatase kappa | PTPRK;DKFZp686C2268 | 20 | 20 | 27.3 | 97.449 | 26.98 | 27.00 | 27.19 | 27.26 | 27.30 | 27.40 | -0.26 | -1.20 | 0.03173 |
| Q16610;Q16610-4;C8CHS3;Q16610-2;B7ZAS5;Q16610-3 | Extracellular matrix protein 1 | ECM1 | 5 | 5 | 12.8 | 60.673 | 23.35 | 22.70 | 23.23 | 23.44 | 23.06 | 23.57 | -0.26 | -1.20 | 0.35068 |
| E9PJK1;E9PRJ8;H0YDL9;H0YDJ9;P60033;E9PIF1;A6NMH8;E9PM31;H0YEE2 | CD81 antigen | CD81 | 2 | 2 | 26.1 | 17.963 | 22.17 | 21.74 | 21.87 | 21.98 | 22.20 | 22.41 | -0.27 | -1.20 | 0.20884 |
| A8KAM5;Q8N474;Q05DS5;Q6ZSL4;Q9BZZ6 | Secreted frizzled-related protein 1 | SFRP1 | 7 | 7 | 31.6 | 35.314 | 24.22 | 23.90 | 24.40 | 23.93 | 24.66 | 24.76 | -0.28 | -1.21 | 0.40680 |
| P30084 | Enoyl-CoA hydratase, mitochondrial | ECHS1 | 2 | 2 | 8.3 | 31.387 | 22.08 | 22.04 | 21.27 | 22.14 | 22.16 | 21.96 | -0.29 | -1.23 | 0.33683 |
| C9JEG3;F5GZK1;B4DNZ2;Q8N8F1;Q9UBQ6;Q8IYF4;Q49A43;Q05DH5;C9IYF5;O00245 | Exostosin-like 2;Processed exostosin-like 2 | EXTL2 | 4 | 4 | 18.8 | 29.877 | 23.51 | 23.37 | 23.69 | 23.66 | 23.88 | 23.91 | -0.30 | -1.23 | 0.07254 |
| Q5H9A7;P01033;B3KQF4;Q58P21;H0Y789;Q5H9B5;Q5H9B4 | Metalloproteinase inhibitor 1 | TIMP1 | 7 | 7 | 65.7 | 16.057 | 27.15 | 26.68 | 27.29 | 27.78 | 27.06 | 27.17 | -0.30 | -1.23 | 0.36326 |
| Q9UKU6;H0YHU0 | Thyrotropin-releasing hormone-degrading ectoenzyme | TRHDE | 31 | 31 | 31.2 | 117 | 27.36 | 27.35 | 27.64 | 27.47 | 27.69 | 28.09 | -0.30 | -1.23 | 0.21997 |
| Q15113;A4D2D2;B4DPJ4 | Procollagen C-endopeptidase enhancer 1 | PCOLCE | 16 | 16 | 44.3 | 47.972 | 26.41 | 26.01 | 26.59 | 26.98 | 26.28 | 26.65 | -0.30 | -1.23 | 0.32117 |
| O00469;O00469-2;E7ETU9;B4DHG3;C9JXZ0;B3KWS3;F8WEW3 | Procollagen-lysine,2-oxoglutarate 5-dioxygenase 2 | PLOD2 | 6 | 6 | 9.8 | 84.685 | 22.69 | 22.42 | 22.63 | 22.86 | 22.64 | 23.16 | -0.31 | -1.24 | 0.14601 |
| B2RBY8;P22413;Q7Z3P5;E9PE72 | Ectonucleotide pyrophosphatase/phosphodiesterase family member 1;Alkaline phosphodiesterase I;Nucleotide pyrophosphatase | ENPP1 | 9 | 9 | 17.8 | 99.869 | 23.92 | 23.84 | 24.13 | 24.27 | 24.13 | 24.42 | -0.31 | -1.24 | 0.06165 |
| H7BYD9;P19021-2;F8WE90;B4DKE0;P19021-4;P19021-3;P19021-6;P19021;P19021-5;A6NMH0 | Peptidyl-glycine alpha-amidating monooxygenase;Peptidylglycine alpha-hydroxylating monooxygenase;Peptidyl-alpha-hydroxyglycine alpha-amidating lyase | PAM | 2 | 2 | 3.5 | 75.563 | 22.29 | 21.90 | 22.28 | 22.50 | 22.68 | 22.23 | -0.31 | -1.24 | 0.16405 |
| F5H1S8;Q14165;H0YG07;F5GX14 | Malectin | MLEC | 2 | 2 | 17.8 | 16.729 | 20.30 | 20.57 | 20.41 | 20.66 | 20.83 | 20.74 | -0.32 | -1.25 | 0.02735 |
| Q6YHK3;Q6YHK3-4;Q6YHK3-2;Q6YHK3-3;Q5XUM6 | CD109 antigen | CD109 | 21 | 21 | 18.8 | 161.69 | 25.80 | 25.39 | 25.63 | 25.84 | 25.88 | 26.05 | -0.32 | -1.25 | 0.07442 |
| Q5TCU3;P07951;Q5TCU8;B4DGC2;P07951-2;A7XZE4;P07951-3;B4E3P1;H0YL52;P09493-9;P09493;Q6ZN40;H0YKP3;B4DTK3;H0YL80;C9IZA2;H0YNC7;P09493-5;H0YK48;B7Z722;P09493-7;P09493-6;Q59GR8;U3KQK2 | Tropomyosin beta chain;Tropomyosin alpha-1 chain | TPM2;TPM2b;TPM1 | 9 | 5 | 25 | 32.814 | 25.58 | 25.78 | 25.50 | 26.28 | 26.15 | 25.41 | -0.33 | -1.25 | 0.31341 |
| Q03167-2;Q03167;E9PKY4 | Transforming growth factor beta receptor type 3 | TGFBR3 | 3 | 3 | 4.4 | 93.427 | 20.94 | 20.71 | 20.74 | 21.03 | 21.02 | 21.33 | -0.33 | -1.26 | 0.05833 |
| P24821;A0A024R884;Q4LE33;P24821-3;Q6MZI1 | Tenascin | TNC;TNC variant protein | 88 | 2 | 51.9 | 240.85 | 30.68 | 30.59 | 30.52 | 31.08 | 30.78 | 30.92 | -0.33 | -1.26 | 0.02868 |
| Q6NVI1;P29966;Q05C82 | Myristoylated alanine-rich C-kinase substrate | MARCKS | 4 | 4 | 42.7 | 14.859 | 23.13 | 23.89 | 24.92 | 25.13 | 24.07 | 23.75 | -0.33 | -1.26 | 0.64313 |
| Q9H173;D6REA1 | Nucleotide exchange factor SIL1 | SIL1 | 3 | 3 | 7.4 | 52.084 | 21.38 | 20.94 | 21.26 | 21.45 | 21.17 | 21.97 | -0.34 | -1.26 | 0.27808 |
| Q13228;Q13228-4;Q13228-2;Q13228-3;B4DPI7;A6PVX1;H0Y532;Q9H8A8;F2Z2W8;F8WCR4;C9JVL0 | Selenium-binding protein 1 | SELENBP1 | 22 | 22 | 55.5 | 52.39 | 25.88 | 25.89 | 26.23 | 26.00 | 26.58 | 26.44 | -0.34 | -1.26 | 0.18148 |
| Q99650-2;Q99650 | Oncostatin-M-specific receptor subunit beta | OSMR | 5 | 5 | 20.5 | 39.503 | 22.58 | 22.54 | 22.88 | 22.90 | 22.74 | 23.42 | -0.35 | -1.28 | 0.20186 |
| A6NLG9;B4DDQ2;Q53HU6;Q53FI4;P21810;A8K7E0;B3KS75;Q8NAB7;B4DNL4;B4DQD6;B4DDN7;C9JKG1 | Biglycan | BGN | 8 | 8 | 34.9 | 34.875 | 24.78 | 23.95 | 24.40 | 24.80 | 24.47 | 24.94 | -0.36 | -1.28 | 0.26588 |
| Q6GMV3 | Putative peptidyl-tRNA hydrolase PTRHD1 | PTRHD1 | 3 | 3 | 25 | 15.805 | 20.07 | 20.13 | 20.32 | 20.20 | 20.07 | 21.34 | -0.37 | -1.29 | 0.42255 |
| B3KNF2;O76061;H0YB13;E5RG57 | Stanniocalcin-2 | STC2 | 6 | 6 | 25.6 | 32.212 | 25.86 | 25.46 | 25.96 | 26.59 | 26.02 | 25.79 | -0.37 | -1.29 | 0.25856 |
| Q8N8Z6-2;Q8N8Z6;Q5H993;Q68DD5 | Discoidin, CUB and LCCL domain-containing protein 1 | DCBLD1 | 3 | 3 | 9.6 | 59.195 | 20.98 | 21.15 | 21.29 | 21.40 | 21.41 | 21.74 | -0.38 | -1.30 | 0.05873 |
| A0A024R8V7;P16035;B4DFW2;K7EIX4;K7EL90 | Metalloproteinase inhibitor 2 | TIMP2 | 10 | 10 | 52.5 | 20.195 | 26.24 | 25.75 | 25.96 | 26.55 | 26.11 | 26.42 | -0.38 | -1.30 | 0.12553 |
| P15104;B4DWM6;Q9NSP3;Q2TU80;A1L199 | Glutamine synthetase | GLUL | 6 | 6 | 17.2 | 42.064 | 24.54 | 24.46 | 25.09 | 25.37 | 25.18 | 24.67 | -0.38 | -1.30 | 0.26110 |
| Q8WVQ1-3;Q8WVQ1;Q8WVQ1-2;K7EN15 | Soluble calcium-activated nucleotidase 1 | CANT1 | 9 | 9 | 37.1 | 38.945 | 24.05 | 23.57 | 24.05 | 24.42 | 24.24 | 24.16 | -0.39 | -1.31 | 0.09621 |
| H0YNP1;O94923;B3KML4 | D-glucuronyl C5-epimerase | GLCE | 2 | 2 | 4.5 | 62.886 | 20.10 | 20.02 | 19.39 | 20.01 | 19.76 | 20.90 | -0.39 | -1.31 | 0.39871 |
| B4E0H8;P26006;P26006-1;Q59F03;D6R9X8;H0YA49 | Integrin alpha-3;Integrin alpha-3 heavy chain;Integrin alpha-3 light chain | ITGA3 | 3 | 3 | 2.9 | 115.17 | 21.79 | 20.80 | 20.81 | 21.50 | 21.66 | 21.41 | -0.39 | -1.31 | 0.31016 |
| Q16394;T2FFJ4;V5QSK8 | Exostosin-1 | EXT1 | 3 | 3 | 5.9 | 86.254 | 21.04 | 20.84 | 20.94 | 21.27 | 21.18 | 21.53 | -0.39 | -1.31 | 0.03121 |
| B3KVA7;P17540;B2R8A3;D6RHV3;D6R998 | Creatine kinase S-type, mitochondrial | CKMT2 | 2 | 2 | 12.6 | 40.479 | 22.49 | 19.51 | 19.06 | 21.71 | 20.69 | 19.85 | -0.39 | -1.31 | 0.76000 |
| P10619-2;P10619;B4E324;X6R5C5;Q59EV6;X6R8A1;Q5JZH0;Q5JZG9 | Lysosomal protective protein;Lysosomal protective protein 32 kDa chain;Lysosomal protective protein 20 kDa chain | CTSA;PPGB | 6 | 6 | 13.2 | 52.488 | 23.17 | 22.57 | 23.50 | 23.51 | 23.30 | 23.63 | -0.40 | -1.32 | 0.23661 |
| Q07954;H0YJI8;Q6PJ72;Q59FG2 | Prolow-density lipoprotein receptor-related protein 1;Low-density lipoprotein receptor-related protein 1 85 kDa subunit;Low-density lipoprotein receptor-related protein 1 515 kDa subunit;Low-density lipoprotein receptor-related protein 1 intracellular domain | LRP1 | 16 | 16 | 4.4 | 504.6 | 24.88 | 24.73 | 24.68 | 24.90 | 25.17 | 25.43 | -0.40 | -1.32 | 0.07160 |
| Q8NHP8;F5H5E2 | Putative phospholipase B-like 2;Putative phospholipase B-like 2 32 kDa form;Putative phospholipase B-like 2 45 kDa form | PLBD2 | 12 | 12 | 22.4 | 65.471 | 26.15 | 25.99 | 26.49 | 26.64 | 26.69 | 26.55 | -0.41 | -1.33 | 0.05316 |
| Q86TY5;Q6FGL0;P17931;Q59FR8;Q6NVH9;G3V3R6 | Galectin-3 | LGALS3 | 6 | 6 | 58.7 | 13.897 | 24.12 | 23.66 | 23.88 | 24.34 | 24.40 | 24.18 | -0.42 | -1.34 | 0.04606 |
| F5H7J9;O75581;B7ZLD0;H0YGW5;Q59EX8;B3KQG5;B3KQA9;E9PHY1 | Low-density lipoprotein receptor-related protein 6 | LRP6 | 13 | 11 | 8.3 | 175.43 | 23.91 | 23.71 | 24.15 | 24.20 | 24.45 | 24.40 | -0.43 | -1.34 | 0.04353 |
| Q9NZ23 |  | YA61 | 9 | 9 | 84.6 | 14.867 | 28.63 | 28.76 | 29.05 | 28.91 | 29.38 | 29.45 | -0.43 | -1.35 | 0.10767 |
| Q6ZRP7;H0Y430;B3KY64 | Sulfhydryl oxidase 2 | QSOX2 | 2 | 2 | 4.3 | 77.528 | 19.79 | 19.31 | 19.48 | 19.93 | 19.92 | 20.05 | -0.44 | -1.36 | 0.03823 |
| P33908;Q6P052 | Mannosyl-oligosaccharide 1,2-alpha-mannosidase IA | MAN1A1 | 9 | 9 | 16.5 | 72.968 | 24.64 | 24.45 | 24.54 | 24.97 | 25.01 | 25.00 | -0.45 | -1.36 | 0.00134 |
| P12110-3;P12110-2;P12110;C9JH44;Q9BUM6;H7C0M5 | Collagen alpha-2(VI) chain | COL6A2 | 7 | 7 | 12 | 87.279 | 23.76 | 23.52 | 23.94 | 24.38 | 23.86 | 24.36 | -0.46 | -1.38 | 0.09131 |
| Q92520;C9JP35;C9JMN4 | Protein FAM3C | FAM3C | 9 | 9 | 32.6 | 24.68 | 25.58 | 25.72 | 25.93 | 26.19 | 26.18 | 26.27 | -0.47 | -1.39 | 0.01151 |
| B4E2S7;P13473-2;P13473;P13473-3;B7Z2R9;H0YCG2;B4DF49 | Lysosome-associated membrane glycoprotein 2 | LAMP2 | 4 | 4 | 10.5 | 39.811 | 24.46 | 24.58 | 24.49 | 24.91 | 25.35 | 24.68 | -0.47 | -1.39 | 0.07631 |
| P12111-2;P12111;E9PCV6;E7ENL6;P12111-4;P12111-5;Q63HQ4;B4E0A4;P12111-3;C9JNG9;Q8N4Z1;I3L392 | Collagen alpha-3(VI) chain | COL6A3;DKFZp686K04147 | 28 | 6 | 13.3 | 321.35 | 25.77 | 25.45 | 25.54 | 26.17 | 26.07 | 26.01 | -0.49 | -1.40 | 0.00954 |
| P17948-4;P17948-2;P17948-3;P17948 | Vascular endothelial growth factor receptor 1 | FLT1 | 2 | 2 | 3.3 | 60.916 | 20.97 | 20.07 | 21.20 | 21.14 | 21.20 | 21.41 | -0.50 | -1.42 | 0.22776 |
| J3QS03;Q9GZX9;Q9GZX9-2 | Twisted gastrulation protein homolog 1 | TWSG1 | 2 | 2 | 13 | 20.365 | 22.06 | 21.88 | 22.87 | 22.67 | 22.80 | 22.85 | -0.51 | -1.42 | 0.17551 |
| P35555 | Fibrillin-1 | FBN1 | 4 | 4 | 2.5 | 312.24 | 24.08 | 23.38 | 23.64 | 24.13 | 24.09 | 24.41 | -0.51 | -1.43 | 0.08721 |
| Q6EMK4 | Vasorin | VASN | 10 | 10 | 16 | 71.712 | 27.84 | 27.32 | 27.39 | 28.26 | 27.85 | 27.99 | -0.51 | -1.43 | 0.06343 |
| P16278-3;Q53G40;P16278;B7Z6Q5;Q53H18;P16278-2;E7EQ29;B7Z5H9;F8WF40;C9JF15;C9JWX1;C9J4G9;C9J539 | Beta-galactosidase | GLB1 | 14 | 14 | 31.1 | 72.75 | 25.22 | 25.20 | 25.38 | 25.63 | 25.81 | 25.92 | -0.52 | -1.43 | 0.00692 |
| P34096 | Ribonuclease 4 | RNASE4 | 5 | 5 | 29.3 | 16.84 | 24.04 | 23.87 | 24.47 | 24.98 | 24.13 | 24.83 | -0.52 | -1.44 | 0.17543 |
| P32004-3;P32004-2;Q7Z3Z9;F5H1H0;P32004;A8K139;F5H025;Q86SE4;E9PHJ4;E7EMY4;E7EVM4;E7EPI4;Q7Z2J8;Q7Z2I3;H0Y5C3;Q7Z2J0 | Neural cell adhesion molecule L1 | L1CAM | 24 | 24 | 26.4 | 138.91 | 26.92 | 26.90 | 26.98 | 27.44 | 27.34 | 27.59 | -0.52 | -1.44 | 0.00257 |
| Q6FHN2;P43235;Q5QP40;H7CEH5;O60911;B2R717 | Cathepsin K | CTSK | 13 | 13 | 43.2 | 36.932 | 26.99 | 26.89 | 27.19 | 27.95 | 27.40 | 27.35 | -0.54 | -1.46 | 0.06276 |
| P05121;P05121-2;B7ZAB0;B7Z4X6;B7Z1D9 | Plasminogen activator inhibitor 1 | SERPINE1 | 19 | 19 | 63.7 | 45.059 | 28.66 | 28.58 | 28.47 | 29.24 | 29.05 | 29.05 | -0.55 | -1.46 | 0.00291 |
| B3KRY3;P11279;B4DWL3;Q59FZ0 | Lysosome-associated membrane glycoprotein 1 | LAMP1 | 7 | 7 | 16.2 | 42.585 | 25.96 | 26.01 | 25.45 | 26.25 | 26.63 | 26.20 | -0.55 | -1.47 | 0.06979 |
| F6KPG5;P02768;B2RBS8;B4DPP6;A8K9P0;B4DPR2;Q8IUK7;H0YA55;B7WNR0;P02768-2;C9JKR2;D6RHD5;H7C013 | Serum albumin | ALB | 7 | 1 | 13.3 | 66.531 | 23.50 | 22.99 | 26.45 | 26.34 | 23.88 | 24.41 | -0.56 | -1.48 | 0.68951 |
| Q9UN70-2;Q9UN70 | Protocadherin gamma-C3 | PCDHGC3 | 5 | 5 | 9.2 | 94.457 | 24.23 | 23.93 | 24.20 | 24.80 | 24.83 | 24.44 | -0.57 | -1.49 | 0.02232 |
| O00300;E5RFV7 | Tumor necrosis factor receptor superfamily member 11B | TNFRSF11B | 14 | 14 | 39.4 | 46.026 | 27.59 | 27.10 | 27.24 | 28.18 | 27.63 | 27.84 | -0.57 | -1.49 | 0.05584 |
| E9PPJ5;P21741;E9PLM6;P21741-2 | Midkine | MDK | 4 | 4 | 41.2 | 14.374 | 23.83 | 23.56 | 24.01 | 24.50 | 24.29 | 24.34 | -0.58 | -1.49 | 0.01670 |
| P14210-5;P14210-2;P14210-4;P14210-3;P14210;A8K6K9;P14210-6;Q59H59;C9JJ35;C9JDP4;C9WSJ4;C9WSJ3 | Hepatocyte growth factor;Hepatocyte growth factor alpha chain;Hepatocyte growth factor beta chain | HGF | 4 | 4 | 20.7 | 33.234 | 22.05 | 21.56 | 21.80 | 22.61 | 22.42 | 22.11 | -0.58 | -1.49 | 0.04631 |
| Q9Y240;Q5U0B9;V9H0X6;M0R081;A0AUH1 | C-type lectin domain family 11 member A | CLEC11A | 5 | 5 | 18.3 | 35.694 | 23.30 | 22.22 | 22.85 | 24.17 | 23.11 | 22.84 | -0.58 | -1.50 | 0.32156 |
| X5D767;F5GWI4;P00813 | Adenosine deaminase | ADA | 2 | 2 | 5 | 40.763 | 20.90 | 21.29 | 20.93 | 19.71 | 22.05 | 23.10 | -0.58 | -1.50 | 0.59547 |
| P08294;B2R9V7;Q16867;M0R1V4 | Extracellular superoxide dismutase [Cu-Zn];Superoxide dismutase [Cu-Zn] | SOD3 | 8 | 8 | 43.3 | 25.851 | 25.65 | 25.84 | 26.23 | 26.50 | 26.67 | 26.32 | -0.59 | -1.51 | 0.03972 |
| H3BS10;P06865;H3BP20;E9PGL4;H3BTD4;H3BU85;B4DKE7;B4DMX7;H3BVH8;H3BT62;H3BQ04;G3XL83;G3XL79;Q16017;V9H0E1;G3XL81;G3XL78;H3BRP6 | Beta-hexosaminidase;Beta-hexosaminidase subunit alpha | HEXA | 20 | 3 | 43.2 | 58.44 | 27.06 | 26.83 | 26.84 | 27.39 | 27.63 | 27.50 | -0.60 | -1.51 | 0.00425 |
| B4DWN1;D6RBV2;Q12907;A8K7T4;D6RIU4;D6RDX1;D6RBH1 | Vesicular integral-membrane protein VIP36 | LMAN2 | 10 | 10 | 43.2 | 32.577 | 25.77 | 25.55 | 25.72 | 26.09 | 26.30 | 26.49 | -0.61 | -1.53 | 0.01011 |
| P27824;B4DGP8;B4E2T8;D6RHJ3;D6RD16;D6RAQ8;D6RAU8;D6RB85;D6RDP7;H0Y9Q7;Q16094 | Calnexin | CANX | 3 | 3 | 7.6 | 67.567 | 21.49 | 20.81 | 19.89 | 21.73 | 20.85 | 21.47 | -0.62 | -1.54 | 0.30768 |
| Q02809;B4DR87;B2R5M9;B4DGN8;B4DHT1;Q5JXB7;Q5JXB9;Q5JXB8 | Procollagen-lysine,2-oxoglutarate 5-dioxygenase 1 | PLOD1 | 17 | 17 | 28.6 | 83.549 | 25.62 | 24.98 | 25.29 | 25.96 | 25.82 | 26.00 | -0.63 | -1.55 | 0.02992 |
| P54802;Q14769;Q59FD0;K7ENX5;K7EQH9 | Alpha-N-acetylglucosaminidase;Alpha-N-acetylglucosaminidase 82 kDa form;Alpha-N-acetylglucosaminidase 77 kDa form | NAGLU;ufHSD2 | 22 | 22 | 39.3 | 82.265 | 26.32 | 26.22 | 26.56 | 26.76 | 27.06 | 27.16 | -0.63 | -1.55 | 0.01610 |
| Q9BRK3-3;Q9BRK3;Q9BRK3-2;A0A024R0A0;B4E385;Q9BRK3-4 | Matrix-remodeling-associated protein 8 | MXRA8 | 13 | 13 | 34.2 | 47.971 | 25.68 | 25.48 | 25.88 | 26.44 | 26.15 | 26.35 | -0.63 | -1.55 | 0.01177 |
| P15848;P15848-2;A8K4A0 | Arylsulfatase B | ARSB | 7 | 7 | 18.9 | 59.687 | 23.28 | 23.50 | 23.57 | 23.99 | 24.25 | 24.03 | -0.64 | -1.56 | 0.00560 |
| P68032;P63267;P62736;A8K3K1;P68133;B7Z6P1;B3KW67;Q5T8M8;A6NL76;B4DUI8;P63267-2;Q5T8M7;Q7Z7J6;B3KUD3;Q13707;F6UVQ4;F8WB63;B8ZZJ2;C9JFL5;B7Z6I1;B3KPP5;Q562S2 | Actin, alpha cardiac muscle 1;Actin, gamma-enteric smooth muscle;Actin, aortic smooth muscle;Actin, alpha skeletal muscle | ACTC1;ACTG2;ACTA2;ACTA1 | 3 | 3 | 18 | 42.019 | 24.28 | 22.80 | 22.87 | 25.60 | 23.90 | 22.45 | -0.66 | -1.59 | 0.55455 |
| B7ZAH9;B4DLB8;W6MEN3;P15291-2;P15291;W6MEN4;Q86XA6 | Beta-1,4-galactosyltransferase 1;Lactose synthase A protein;N-acetyllactosamine synthase;Beta-N-acetylglucosaminylglycopeptide beta-1,4-galactosyltransferase;Beta-N-acetylglucosaminyl-glycolipid beta-1,4-galactosyltransferase;Processed beta-1,4-galactosyltransferase 1 | B4GALT1 | 2 | 2 | 12.4 | 29.976 | 20.21 | 20.33 | 19.93 | 20.77 | 20.91 | 20.80 | -0.67 | -1.59 | 0.00605 |
| P04066;B7Z362;B5MDC5 | Tissue alpha-L-fucosidase | FUCA1 | 2 | 2 | 7.1 | 53.688 | 19.70 | 19.94 | 19.70 | 19.97 | 20.71 | 20.73 | -0.69 | -1.61 | 0.05887 |
| P42785;P42785-2;B7Z7Q6;B3KR26;E9PIG4;E9PNF7;E9PQB5;E9PL85;E9PQN3;E9PKN6;E9PNJ1;E9PLY4;E9PL49 | Lysosomal Pro-X carboxypeptidase | PRCP | 15 | 15 | 33.7 | 55.799 | 26.99 | 26.84 | 27.11 | 27.60 | 27.65 | 27.75 | -0.69 | -1.61 | 0.00155 |
| P30530-2;P30530;M0R0W6 | Tyrosine-protein kinase receptor UFO | AXL | 6 | 6 | 8 | 97.376 | 25.92 | 26.21 | 25.58 | 26.38 | 26.52 | 26.88 | -0.69 | -1.61 | 0.04252 |
| B7Z4S8;Q99538;Q6I9U9;A8K669;B7Z4A4;Q86TV3;Q96CY7;Q86TV2;G3V4E4;G3V2T4;G3V4P5;H0YJN9;G3V3Z4 | Legumain | LGMN | 13 | 13 | 39.8 | 47.071 | 26.70 | 26.86 | 26.80 | 27.59 | 27.34 | 27.49 | -0.69 | -1.62 | 0.00138 |
| O94985-2;B4E3Q1;O94985;Q5SR54;B3KMD3 | Calsyntenin-1;Soluble Alc-alpha;CTF1-alpha | CLSTN1 | 38 | 38 | 40.4 | 108.64 | 29.63 | 29.74 | 29.75 | 30.30 | 30.39 | 30.51 | -0.69 | -1.62 | 0.00070 |
| P20933;H0Y9C7;H0Y8L9;B4E0G5 | N(4)-(beta-N-acetylglucosaminyl)-L-asparaginase;Glycosylasparaginase alpha chain;Glycosylasparaginase beta chain | AGA | 11 | 11 | 41.6 | 37.208 | 27.45 | 27.65 | 27.71 | 28.18 | 28.45 | 28.29 | -0.70 | -1.63 | 0.00296 |
| V9HW65;P07355;P07355-2;H0YN42;H0YMD0;H0YMU9;A6NMY6;B4DNH8;H0YM50;H0YKS4;B3KRQ1;H0YMM1;H0YNP5;H0YN28;H0YL33;H0YKZ7;H0YLV6;H0YMT9;H0YKX9;H0YKL9;H0YMW4;H0YKV8;H0YMD9;H0YNA0;H0YNB8;H0YKN4;H0YN52;H0YLE2 | Annexin A2;Annexin;Putative annexin A2-like protein | ANXA2;ANXA2P2 | 26 | 26 | 72.6 | 38.576 | 27.93 | 27.67 | 28.03 | 28.80 | 28.41 | 28.55 | -0.71 | -1.63 | 0.01055 |
| Q9Y680-3;Q9Y680-2;Q9Y680;H7BZJ4;F8WE12;B4DRE2;D3DPF8 | Peptidyl-prolyl cis-trans isomerase FKBP7;Peptidyl-prolyl cis-trans isomerase | FKBP7 | 2 | 2 | 9 | 25.723 | 21.04 | 20.81 | 21.12 | 21.60 | 21.41 | 22.08 | -0.71 | -1.64 | 0.03219 |
| Q9BTY2;Q7Z6V1;Q7Z6V2 | Plasma alpha-L-fucosidase | FUCA2 | 23 | 23 | 41.1 | 54.066 | 28.66 | 28.89 | 29.14 | 29.40 | 29.73 | 29.72 | -0.72 | -1.65 | 0.01511 |
| Q8NCC3;H3BMU8;B4DUD1;H3BM47;B4DJW4;H3BPT3;B4DPU0 | Group XV phospholipase A2 | PLA2G15 | 8 | 8 | 23.1 | 46.657 | 24.42 | 24.23 | 24.48 | 24.94 | 25.05 | 25.32 | -0.73 | -1.66 | 0.00558 |
| Q16270;Q16270-2;A0A024RDA6 | Insulin-like growth factor-binding protein 7 | IGFBP7 | 20 | 20 | 58.5 | 29.13 | 31.35 | 31.12 | 31.37 | 31.99 | 31.84 | 32.21 | -0.73 | -1.66 | 0.00556 |
| Q9BRK5;Q9BRK5-6;H0Y3T6;Q9BRK5-4;Q9BRK5-3;G3V1E2;Q9BRK5-2;Q9BRK5-5 | 45 kDa calcium-binding protein | SDF4 | 8 | 8 | 30.1 | 41.806 | 24.54 | 24.14 | 24.32 | 25.30 | 24.94 | 25.03 | -0.75 | -1.69 | 0.00896 |
| B4DEN5;O43505;B3KQQ7;B4DGI0 | N-acetyllactosaminide beta-1,3-N-acetylglucosaminyltransferase | B3GNT1 | 5 | 5 | 17.6 | 46.38 | 23.33 | 23.41 | 23.15 | 24.37 | 23.76 | 24.06 | -0.77 | -1.71 | 0.01621 |
| E9PNW4;P13987;E9PR17;H0YET2 | CD59 glycoprotein | CD59 | 4 | 4 | 30.6 | 11.985 | 26.06 | 26.00 | 25.50 | 26.58 | 26.71 | 26.64 | -0.79 | -1.72 | 0.01204 |
| P01130-2;P01130-3;Q9UH51;P01130-5;P01130;J3KMZ9;H0YMD1;P01130-4;P01130-6;E2J5K4;H0YM92 | Low-density lipoprotein receptor | LDLR | 6 | 6 | 9.1 | 75.854 | 24.48 | 25.04 | 25.54 | 26.31 | 25.63 | 25.48 | -0.79 | -1.73 | 0.12021 |
| P50454;B4DN87;A8K259;E9PPV6;E9PR70;E9PKH2;E9PK86;E9PMI5;E9PNX1;Q9NPA9;E9PJH8;E9PIG2;E9PRS3;E9PQ34;E9PLA6;H0YEP8 | Serpin H1 | SERPINH1 | 20 | 20 | 53.6 | 46.44 | 28.31 | 28.41 | 27.98 | 28.68 | 29.08 | 29.30 | -0.79 | -1.73 | 0.02371 |
| Q5D862 | Filaggrin-2 | FLG2 | 2 | 2 | 1 | 248.07 | 21.21 | 21.54 | 21.32 | 22.92 | 21.83 | 21.72 | -0.80 | -1.74 | 0.11099 |
| B4DQV1;B4DRR8;D3DSM7;P12109;Q05BT9 | Collagen alpha-1(VI) chain | COL6A1 | 6 | 6 | 19.8 | 43.606 | 23.59 | 23.70 | 23.49 | 24.35 | 24.04 | 24.79 | -0.80 | -1.74 | 0.02348 |
| Q99470 | Stromal cell-derived factor 2 | SDF2 | 2 | 2 | 18 | 23.026 | 21.52 | 21.15 | 21.51 | 22.11 | 21.77 | 22.71 | -0.80 | -1.74 | 0.05616 |
| B4DSS7;Q9BZM5;Q8IZX9;J7HHH7;J7HBC9;J7HHG3;J7HEN7;J7HEM2;J7HBD2;J7HBB6;Q6H3X3-2;Q4V9S8;C9JAK3;Q5VY80;Q6H3X3 | NKG2D ligand 2 | ULBP2;RAET1L;RAET1H | 5 | 5 | 24.3 | 25.611 | 23.97 | 23.43 | 24.08 | 24.31 | 24.88 | 24.70 | -0.80 | -1.75 | 0.03728 |
| P07711;B3KQK4;A5PLM9;Q5T8F0;Q9HBQ7;Q5K630;Q6LAF7;Q5NE16 | Cathepsin L1;Cathepsin L1 heavy chain;Cathepsin L1 light chain | CTSL1 | 15 | 14 | 45 | 37.564 | 29.06 | 28.86 | 29.11 | 29.90 | 29.86 | 29.78 | -0.83 | -1.78 | 0.00058 |
| P34059;B2R6P1;Q6ZNJ9;F5H325;B7Z267;Q6YL38;Q9HAL1;Q96I49;H3BP66 | N-acetylgalactosamine-6-sulfatase | GALNS;FLJ00319 | 20 | 20 | 44.3 | 58.025 | 27.05 | 27.02 | 27.09 | 27.54 | 28.14 | 28.01 | -0.84 | -1.79 | 0.01029 |
| P08758;E9PHT9;B4DNG6;D6RBE9;D6RBL5;D6RCN3 | Annexin A5;Annexin | ANXA5 | 6 | 6 | 20.9 | 35.936 | 23.85 | 24.00 | 24.11 | 24.87 | 25.02 | 24.61 | -0.84 | -1.80 | 0.00411 |
| Q6X907;Q68DN3;Q71SW6;Q9H2E1;Q59F20;E9PEP6;A8K9V7;O14786;O14786-3;E7EX60;O14786-2;Q5T7F0;Q6AWA9;Q5JWQ4;Q5JWQ6;H0Y4A0;Q9H2D9;Q9H2E0 | Neuropilin-1 | NRP1;DKFZp781F1414;DKFZp686A03134 | 24 | 24 | 36.1 | 101.29 | 26.67 | 26.51 | 26.85 | 27.61 | 27.28 | 27.68 | -0.85 | -1.80 | 0.00605 |
| E9PMI0;E9PQU7;Q6UX15-2;Q6UX15;E9PR90;B4DP26;E9PQY8;E9PK64;B4DDS5 | Layilin | LAYN | 7 | 7 | 24.4 | 29.584 | 23.92 | 23.63 | 23.26 | 24.15 | 24.71 | 24.51 | -0.85 | -1.81 | 0.02821 |
| P13686;K7EJD9;K7EIP0;K7ESF2 | Tartrate-resistant acid phosphatase type 5 | ACP5 | 7 | 7 | 26.5 | 36.598 | 23.90 | 23.80 | 23.93 | 23.79 | 25.26 | 25.15 | -0.85 | -1.81 | 0.14665 |
| Q53HP2;O14773;D3DQU2;B2R608;B4DIV8;B4DE89;B4E0C7;B4DVA5;B4DEQ3;O14773-2;B4DSE2;E7EV34 | Tripeptidyl-peptidase 1 | TPP1 | 13 | 13 | 35.5 | 61.212 | 25.73 | 25.42 | 25.54 | 26.30 | 26.38 | 26.57 | -0.86 | -1.81 | 0.00200 |
| P03950 | Angiogenin | ANG | 3 | 3 | 30.6 | 16.55 | 23.93 | 23.83 | 23.59 | 24.92 | 24.35 | 24.66 | -0.86 | -1.81 | 0.01135 |
| P05109 | Protein S100-A8;Protein S100-A8, N-terminally processed | S100A8 | 2 | 2 | 19.4 | 10.834 | 19.58 | 20.81 | 19.92 | 23.59 | 19.70 | 19.63 | -0.87 | -1.83 | 0.55693 |
| E9PIF4;Q99519;A8K4K1;Q6IBT5;Q6Q4G7;Q6Q4G9 | Sialidase-1 | NEU1 | 4 | 4 | 18.7 | 29 | 22.10 | 22.07 | 22.04 | 22.93 | 22.74 | 23.17 | -0.87 | -1.83 | 0.00224 |
| Q6UVY6;Q6UVY6-2;A6PVS1 | DBH-like monooxygenase protein 1 | MOXD1 | 11 | 11 | 15.8 | 69.652 | 27.09 | 26.99 | 27.09 | 27.77 | 28.08 | 27.96 | -0.88 | -1.83 | 0.00082 |
| P24592;F8VYK9 | Insulin-like growth factor-binding protein 6 | IGFBP6 | 2 | 2 | 10.4 | 25.322 | 22.57 | 21.20 | 22.39 | 23.06 | 22.58 | 23.18 | -0.88 | -1.85 | 0.13130 |
| O75635-2;O75635;A8K3Q8;A8K4B1;Q6MZG5;C9JM00;C9JA68;C9JZR8 | Serpin B7 | SERPINB7 | 20 | 20 | 49.3 | 41.174 | 26.44 | 26.63 | 26.67 | 27.22 | 27.53 | 27.70 | -0.91 | -1.87 | 0.00448 |
| P24752;E9PRQ6;Q96FG8;G3XAB4;H0YEL7 | Acetyl-CoA acetyltransferase, mitochondrial | ACAT1 | 3 | 3 | 8 | 45.199 | 21.16 | 21.06 | 21.52 | 21.96 | 22.26 | 22.24 | -0.91 | -1.88 | 0.00571 |
| Q9NSD0;Q16519;P07225;Q8IXD4;Q8IXD1;Q8IXC3;H7BXT0;B4E1L6;G5E9F8 | Vitamin K-dependent protein S | PROS1 | 2 | 2 | 3.4 | 72.48 | 19.76 | 20.25 | 19.74 | 21.00 | 20.89 | 20.62 | -0.92 | -1.89 | 0.01065 |
| Q6UXH9-3;B7Z6E5;E9PIV1;B7Z4A8;Q6UXH9;Q6UXH9-2;E9PMN5;E9PQ70 | Inactive serine protease PAMR1 | PAMR1 | 2 | 2 | 3.4 | 67.541 | 20.25 | 20.25 | 20.20 | 21.30 | 20.98 | 21.20 | -0.93 | -1.90 | 0.00059 |
| B4DSZ6;B4DFL2;Q53GL5;P48735;Q13584;H0YL11 | Isocitrate dehydrogenase [NADP];Isocitrate dehydrogenase [NADP], mitochondrial | IDH2 | 4 | 4 | 22.7 | 36.171 | 21.91 | 22.47 | 21.25 | 23.66 | 22.37 | 22.38 | -0.93 | -1.90 | 0.16993 |
| B4DTC0;D3DPH5;Q5PR22;P05997 | Collagen alpha-2(V) chain | COL5A2 | 4 | 4 | 8.8 | 39.993 | 22.25 | 21.89 | 21.89 | 23.58 | 23.08 | 22.18 | -0.93 | -1.91 | 0.09356 |
| Q9BVJ8;B4DVA7;B4DVL8;Q6PJ76 | Beta-hexosaminidase | HEXA | 2 | 2 | 6.1 | 47.094 | 23.15 | 22.22 | 22.78 | 23.41 | 23.59 | 23.98 | -0.94 | -1.92 | 0.04072 |
| P28799-3;K7EQ05;B4E1G5;P28799-2;B4DJI2;P28799;K7EKL3;K7EQI0 | Granulins;Acrogranin;Paragranulin;Granulin-1;Granulin-2;Granulin-3;Granulin-4;Granulin-5;Granulin-6;Granulin-7 | GRN | 3 | 3 | 7.7 | 44.132 | 22.28 | 22.39 | 22.01 | 23.46 | 23.22 | 22.83 | -0.95 | -1.93 | 0.01178 |
| J3KMY5;Q53HV6;P61916;G3V3E8;E7EMS2;B4DV10;G3V3D1;G3V2V8;B4DQV7;B2R4S5;H0YIZ1 | Epididymal secretory protein E1 | NPC2 | 11 | 11 | 52.7 | 16.23 | 28.36 | 28.15 | 28.02 | 29.15 | 29.12 | 29.11 | -0.95 | -1.93 | 0.00066 |
| Q9UNW1;B2R7D2;Q9UNW1-3;Q9UNW1-2;B4E394;Q9UNW1-4 | Multiple inositol polyphosphate phosphatase 1 | MINPP1 | 3 | 3 | 7 | 55.051 | 21.33 | 21.78 | 20.28 | 22.25 | 22.14 | 21.85 | -0.95 | -1.93 | 0.10716 |
| B4DGD7;P22304;B3KWA1;P22304-2;B4DIX1;P22304-3;Q14603;H0YB91;O60597 | Iduronate 2-sulfatase;Iduronate 2-sulfatase 42 kDa chain;Iduronate 2-sulfatase 14 kDa chain | IDS | 13 | 13 | 35.4 | 52.421 | 24.89 | 24.83 | 24.87 | 25.60 | 25.96 | 25.88 | -0.95 | -1.94 | 0.00099 |
| O75063;X6RH03 | Glycosaminoglycan xylosylkinase | FAM20B | 4 | 4 | 11.2 | 46.432 | 21.81 | 21.63 | 21.29 | 23.05 | 22.65 | 21.97 | -0.98 | -1.97 | 0.04833 |
| P09619;A8KAM8;Q59F04;E5RJ14;E5RH16;B5A957;E5RII0 | Platelet-derived growth factor receptor beta;Tyrosine-protein kinase receptor | PDGFRB | 6 | 6 | 7.7 | 123.97 | 22.79 | 23.01 | 22.77 | 24.27 | 23.48 | 23.76 | -0.98 | -1.97 | 0.01570 |
| P19022;A8MWK3;C9J8J8;C9J126;C9JMH2 | Cadherin-2 | CDH2 | 16 | 16 | 23.6 | 99.808 | 26.32 | 26.39 | 26.39 | 27.23 | 27.45 | 27.36 | -0.98 | -1.98 | 0.00014 |
| O43852;B3KQF5;O43852-3;O43852-5;O43852-6;O43852-9;O43852-2;B3KNG6;O43852-4;O43852-13;O43852-14;O43852-11;O43852-10;H0Y875;O43852-15;B3KQK3;O43852-8;O43852-12;O43852-7 | Calumenin | CALU | 11 | 11 | 38.7 | 37.106 | 26.38 | 25.97 | 25.91 | 27.29 | 27.14 | 26.78 | -0.98 | -1.98 | 0.00972 |
| I0B0K3;I0B0K4;Q05331;I0B0K5;I0B0K6;I0B0K7;I0B0K8;P20930;Q4JFL9 | Filaggrin | FLG | 2 | 2 | 2.9 | 91.099 | 20.58 | 20.50 | 20.79 | 22.84 | 21.04 | 21.05 | -1.02 | -2.03 | 0.16495 |
| Q0QEN7;P06576;H0YH81;F8VPV9;F8W079;F8W0P7;F8VQY0;CON__tr\|N9TUW5\|N9TUW5_MYCAR;CON__tr\|D1J7G6\|D1J7G6_MYCHP;CON__tr\|C4XEK2\|C4XEK2_MYCFP | ATP synthase subunit beta;ATP synthase subunit beta, mitochondrial | ATP5B | 6 | 6 | 24.9 | 48.113 | 24.75 | 22.66 | 23.39 | 25.67 | 24.91 | 23.68 | -1.15 | -2.22 | 0.24355 |
| Q96L73-2;B2RWP5;Q96L73;D6RBV9;D6RA58;A4QPE5;Q9H6H8;Q658U6;Q9H6B5;Q96L73-3 | Histone-lysine N-methyltransferase, H3 lysine-36 and H4 lysine-20 specific | NSD1;DKFZp666C163 | 2 | 2 | 0.9 | 267.34 | 21.71 | 21.24 | 22.61 | 24.97 | 22.54 | 21.75 | -1.23 | -2.35 | 0.30486 |
| Q5T749 | Keratinocyte proline-rich protein | KPRP | 3 | 3 | 7.4 | 64.135 | 21.98 | 22.40 | 21.77 | 25.21 | 22.83 | 22.15 | -1.34 | -2.54 | 0.22834 |
